# Mendelian randomization analysis for attention deficit/hyperactivity disorder: studying a broad range of exposures and outcomes

**DOI:** 10.1093/ije/dyac128

**Published:** 2022-06-12

**Authors:** María Soler Artigas, Cristina Sánchez-Mora, Paula Rovira, Laura Vilar-Ribó, Josep Antoni Ramos-Quiroga, Marta Ribasés

**Affiliations:** Psychiatric Genetics Unit, Group of Psychiatry, Mental Health and Addiction, Vall d'Hebron Research Institute (VHIR), Universitat Autònoma de Barcelona, Barcelona, Spain; Department of Mental Health, Hospital Universitari Vall d'Hebron, Barcelona, Spain; Biomedical Network Research Centre on Mental Health (CIBERSAM), Instituto de Salud Carlos III, Madrid, Spain; Department of Genetics, Microbiology, and Statistics, Faculty of Biology, Universitat de Barcelona, Barcelona, Spain; Psychiatric Genetics Unit, Group of Psychiatry, Mental Health and Addiction, Vall d'Hebron Research Institute (VHIR), Universitat Autònoma de Barcelona, Barcelona, Spain; Department of Mental Health, Hospital Universitari Vall d'Hebron, Barcelona, Spain; Biomedical Network Research Centre on Mental Health (CIBERSAM), Instituto de Salud Carlos III, Madrid, Spain; Department of Genetics, Microbiology, and Statistics, Faculty of Biology, Universitat de Barcelona, Barcelona, Spain; Psychiatric Genetics Unit, Group of Psychiatry, Mental Health and Addiction, Vall d'Hebron Research Institute (VHIR), Universitat Autònoma de Barcelona, Barcelona, Spain; Department of Mental Health, Hospital Universitari Vall d'Hebron, Barcelona, Spain; Vicerectorat de Recerca, postdoctoral researcher Margarita Salas, Universitat de Barcelona, Barcelona, Spain; Departament of Psychiatry, Faculty of Medicine, Universidad de Granada, Spain; Psychiatric Genetics Unit, Group of Psychiatry, Mental Health and Addiction, Vall d'Hebron Research Institute (VHIR), Universitat Autònoma de Barcelona, Barcelona, Spain; Department of Mental Health, Hospital Universitari Vall d'Hebron, Barcelona, Spain; Biomedical Network Research Centre on Mental Health (CIBERSAM), Instituto de Salud Carlos III, Madrid, Spain; Psychiatric Genetics Unit, Group of Psychiatry, Mental Health and Addiction, Vall d'Hebron Research Institute (VHIR), Universitat Autònoma de Barcelona, Barcelona, Spain; Department of Mental Health, Hospital Universitari Vall d'Hebron, Barcelona, Spain; Biomedical Network Research Centre on Mental Health (CIBERSAM), Instituto de Salud Carlos III, Madrid, Spain; Department of Psychiatry and Forensic Medicine, Universitat Autònoma de Barcelona, Barcelona, Spain; Psychiatric Genetics Unit, Group of Psychiatry, Mental Health and Addiction, Vall d'Hebron Research Institute (VHIR), Universitat Autònoma de Barcelona, Barcelona, Spain; Department of Mental Health, Hospital Universitari Vall d'Hebron, Barcelona, Spain; Biomedical Network Research Centre on Mental Health (CIBERSAM), Instituto de Salud Carlos III, Madrid, Spain; Department of Genetics, Microbiology, and Statistics, Faculty of Biology, Universitat de Barcelona, Barcelona, Spain

**Keywords:** ADHD, Mendelian randomization, causal analysis using summary effect estimates

## Abstract

**Background:**

Attention deficit/hyperactivity disorder (ADHD) is a highly prevalent neurodevelopmental disorder caused by a combination of genetic and environmental factors and is often thought as an entry point into a negative life trajectory, including risk for comorbid disorders, poor educational achievement or low income. In the present study, we aimed to clarify the causal relationship between ADHD and a comprehensive range of related traits.

**Methods:**

We used genome-wide association study (GWAS) summary statistics for ADHD (*n* = 53 293) and 124 traits related to anthropometry, cognitive function and intelligence, early life exposures, education and employment, lifestyle and environment, longevity, neurological, and psychiatric and mental health or personality and psychosocial factors available in the MR-Base database (16 067 ≤*n* ≤766 345). To investigate their causal relationship with ADHD, we used two-sample Mendelian randomization (MR) with a range of sensitivity analyses, and validated MR findings using causal analysis using summary effect estimates (CAUSE), aiming to avoid potential false-positive results.

**Results:**

Our findings strengthen previous evidence of a causal effect of ADHD liability on smoking and major depression, and are consistent with a causal effect on odds of decreased average total household income [odds ratio (OR) = 0.966, 95% credible interval (CrI) = (0.954, 0.979)] and increased lifetime number of sexual partners [OR = 1.023, 95% CrI = (1.013, 1.033)]. We also found evidence for a causal effect on ADHD for liability of arm predicted mass and weight [OR = 1.452, 95% CrI = (1.307, 1.614) and OR = 1.430, 95% CrI = (1.326, 1.539), respectively] and time spent watching television [OR = 1.862, 95% CrI = (1.545, 2.246)], and evidence for a bidirectional effect for age of first sexual intercourse [beta = −0.058, 95% CrI = (−0.072, −0.044) and OR = 0.413, 95% CrI = (0.372, 0.457), respectively], odds of decreased age completed full-time education [OR = 0.972, 95% CrI = (0.962, 0.981) and OR = 0.435, 95% CrI = (0.356, 0.533), respectively] and years of schooling [beta = -0.036, 95% CrI = (−0.048, −0.024) and OR = 0.458, 95% CrI = (0.411, 0.511), respectively].

**Conclusions:**

Our results may contribute to explain part of the widespread co-occurring traits and comorbid disorders across the lifespan of individuals with ADHD and may open new opportunities for developing preventive strategies for ADHD and for negative ADHD trajectories.

Key Messages

- Our results are consistent with a causal effect of attention deficit/hyperactivity disorder (ADHD) genetic liability decreasing average total household income and increasing lifetime number of sexual partners.
- We detect a positive effect of the liability of anthropometric traits (arm predicted mass and weight) and of time spent watching television on ADHD.
- We show evidence for a bidirectional negative effect between liability of ADHD and of education outcomes (years of schooling and age completed full-time education), age of first sexual intercourse and past tobacco smoking for non-heavy smokers.

## Introduction

Attention deficit/hyperactivity disorder (ADHD) is a neurodevelopmental disorder with a prevalence of around 5.3% in childhood and 2.8% in adulthood.^1^^,^^2^ The aetiology of ADHD involves a combination of genetic and environmental factors, with an estimated heritability of ∼70–80%.^3^^,^^4^ Potential environmental risk factors include pre- and perinatal risk factors (maternal smoking or alcohol consumption, low birthweight, prematurity), exposure to environmental toxins, unfavourable psychosocial conditions (severe early childhood deprivation, maternal hostility) or low socioeconomic status.^5^^,^^6^

ADHD is characterized by a persistent pattern of inattentive, hyperactive and impulsive behaviour; however, its clinical presentation is heterogeneous, with a wide spectrum of severity and symptoms that often overlap with other conditions.^7^ There are a number of traits that are not part of the core diagnostic criteria for ADHD, which can nevertheless influence severity, persistence and treatment decisions. For instance, individuals with ADHD often have a poor cognitive performance in executive functions, such as response inhibition, vigilance, working memory or planning, personality profiles with low effortful control and high neuroticism,^8–11^ or emotion dysregulation problems such as irritability or temper outbursts.^12^ In addition, up to 70–80% of ADHD patients suffer from comorbid disorders across their lifespan.^13^ These include other psychiatric conditions, such as major depressive, oppositional defiant, bipolar or substance use disorders, but also somatic diseases such as obesity, sleep disorders or migraine.^7^ The presence of comorbidities in ADHD worsens symptom progression, disorder course and outcome, and also increases mortality rates.^8^^,^^14^ In this context ADHD can be thought of as an entry point into a negative life trajectory with higher risks for poor educational achievement, unemployment or criminality, among others.^7^

Most studies undertaken to date have reported association between ADHD and comorbid traits, but inferring causality can be more challenging due to the potential effect of confounding factors or reverse causality. Different strategies have been developed to overcome these inference problems, and the causality for ADHD and a number of traits have been tested using: (i) longitudinal analyses, some of them undertaken in twins; (ii) Mendelian randomization (MR), which uses genetic variants as proxies for an exposure (instrumental variables) to test for a causal effect on an outcome^15^; and (iii) the latent causal variable (LCV) model, based on a latent variable that mediates the genetic correlation between two traits and quantifies the degree of causality between them.^16^ When using only one approach, longitudinal analyses have reported a causal role for low family income in early childhood on ADHD^6^ and of ADHD on lower educational achievement.^17^ In addition, MR studies have reported an effect of the liability of low birthweight increasing the risk for ADHD,^18^ an effect of higher intelligence lowering the risk for ADHD^19^ and an effect of the genetic liability to ADHD increasing the risk for asthma^20^ and coronary artery disease, as well as a positive bidirectional effect for childhood obesity.^21^ When more than one approach was used, consistent results were found for an effect for ADHD liability on major depression^22^ and inconsistent results, which require further investigation, were identified for body mass index (BMI),^23–25^ phone use,^26^ smoking, cannabis and alcohol use.^27–30^

Overall, the evidence from causal inference analyses undertaken for ADHD is hard to interpret, in some cases due to the limited number of strategies applied^19^^,^^20^ or because inconsistent findings are found when using different approaches.^29^^,^^30^ In the present study we aim to clarify the relationship between ADHD and a comprehensive range of related traits, using genome-wide association studies (GWAS) datasets available,^31^^,^^32^ following current guidelines on two-sample MR analyses^33^ and a range of sensitivity analyses. In addition, we validated MR findings using causal analysis using summary effect estimates (CAUSE), a recently developed method to account for horizontal pleiotropy that acts through a common shared heritable factor, and avoids potential false-positive results.^34^

## Methods

### GWAS dataset selection

We selected GWAS summary statistics available in the MR-Base database^31^^,^^32^ in May 2020 (*n* = 31 772) which fulfilled the following inclusion criteria: (i) sample size [N effective = 4 × N cases × N controls/(N cases + N controls) for binary traits] >5000; (ii) results available in more than 450 000 genetic variants; (iii) European ancestry; (iv) non sex-specific; and (v) more than three independent genome-wide significant signals (*P *<5.00e-08). This reduced the number of traits to 1259 (Supplementary Table S1, available as Supplementary data at *IJE* online). At this point we removed traits related to diet (*n *= 52), to metabolites’ levels (*n *= 132), to procedural metrics or biological samples (*n *= 76) and to clinical traits (*n *= 600), except those related to neurological, psychiatric and mental health; and selected traits related to anthropometry, cognitive function and intelligence, early life exposures, education and employment, lifestyle and environment, longevity, neurological, psychiatric and mental health or personality and psychosocial factors. We then removed duplicated and unordered categorical traits (*n* = 150, Supplementary Table S1). Finally, in order to reduce further the number of traits analysed to those with causal relationships most likely to be identified by MR methods, we removed traits with a heritability Z score ≤4 and an ADHD genetic correlation Z score ≤2, obtained using LD score regression,^35^^,^^36^ ending up with a total of 124 traits included in the analysis (Supplementary Tables S1 and S2, available as Supplementary data at *IJE* online). The MR-Base summary statistics used for these 124 traits were obtained from different sources: European Bioinformatics Institute (EBI) database,^37^ Neale Lab and Integrative Epidemiology Unit (IEU) analyses of UK Biobank phenotypes [http://www.nealelab.is/uk-biobank]^38^ and manually collected and curated data from different consortia for MR-Base.^19^^,^^39–47^

### Mendelian randomization

#### Main analysis

We ran two-sample MR in both directions to assess the potential causal relationship between ADHD and every selected trait, using the multiplicative random effects inverse variance weighted (IVW) method as the main analysis.^48^ MR analyses only included independent single nucleotide polymorphisms (SNPs) (r^2^ <0.001 or distance >10 000 kb) with *P *<5.00e-08 in the exposure, and variants meeting the threshold for both the exposure and the outcome were removed. For exposure variants not found in the outcome, GWAS proxies were used instead (r^2^ ≥0.8, obtained using 1000 Genomes European sample). ADHD liability genetic instruments are provided in Supplementary Table S3 (available as Supplementary data at *IJE* online). False-discovery rate (FDR) across all tests considered was used to correct for multiple testing.

#### MR sensitivity analyses

For IVW results with FDR corrected *P *<0.05, we undertook sensitivity analyses to assess the robustness of the findings under weaker assumptions, given that the genetic variants used as instruments must meet three assumptions for IVW results to be valid: (i) robust association with the exposure; (ii) no horizontal pleiotropy, or association with the outcome through a pathway independent of the exposure; and (iii) independence of confounders that influence the exposure and the outcome.

We used weighted median and weighed mode methods, which only require a subset of variants to be valid instruments and are robust to outliers.^49^^,^^50^ Under equal weights, the weighted median requires at least half of the variants to be valid instruments, and the weighted mode requires more variants to estimate the true causal effect than any other quantity.^49^^,^^50^ We used MR-PRESSO, which performs a test to detect horizontal pleiotropy (MR-PRESSO global test), and if detected, it removes horizontal pleiotropic outliers and then performs the IVW method using the remaining instruments.^51^ We also applied MR-Egger, which is affected by outlying data points but allows all genetic variants to have pleiotropic effects, assuming that these effects are independent of the variant-exposure associations.^52^^,^^53^ MR-Egger also implements a pleiotropy test, however, when the assumption of no measurement error in the SNP-exposure effect estimates (NOME assumption) is violated ($I_{\text{GX}}^{2}$ <0.9). MR-Egger causal estimates are biased towards the null, and the pleiotropy test type I error can be inflated.^54^ For this reason, when 0.6 <I^2^_GX_< 0.9 we implemented the method of simulation extrapolation (SIMEX) to obtain an adjusted estimate of the causal effect.^54^ When $I_{\text{GX}}^{2}$ <0.6, we disregarded MR-Egger results. We also calculated F statistics for continuous exposures to measure the strength of the instruments used; as a ‘rule of thumb’, F statistics >10 indicate strong-enough instruments.^55^ In order to avoid results due to reverse causation, we also applied Steiger filtering, removing instruments from the analysis if they did not explain substantially more of the variance in the exposure trait than in the outcome and undertaking the IVW method on the remaining set of instruments.^56^ Variance explained for binary traits was estimated using Equation 10 from Lee *et al.*,^57^ as implemented by get_r_from_lor, R function within the TwoSampleMR package. In addition, we ran heterogeneity tests and leave-one-out analyses and generated scatter, funnel and forest plots. TwoSampleMR v0.5.5 and MRPRESSO R packages were used for these analyses.^31^^,^^51^

We considered that there was evidence of a causal relationship if: (i) IVW FDR *P *<0.05; (ii) the same direction of effect as the IVW beta estimate and *P *<0.05 was found for the weighted median and mode, MR PRESSO, IVW after Steiger filtering and when there was also evidence of pleiotropy (MR-Egger intercept *P *<0.05), MR-Egger or SIMEX if $I_{\text{GX}}^{2}$ >0.9 or 0.6<$I_{\text{GX}}^{2}$<=0.9, respectively; and (iii) F statistic >10 for continuous exposures.

#### Causal analysis using summary effect estimates

For those traits that met the MR sensitivity criteria, we also ran causal analysis using summary effect estimates (CAUSE).^34^ Uncorrelated horizontal pleiotropy occurs when the effects on the exposure and the outcome are uncorrelated and it can be accounted for by methods such as MR-Egger or MR-PRESSO. Correlated horizontal pleiotropy takes place when the effects on the exposure and the outcome act through a shared heritable factor, and is harder to account for by current MR methods. CAUSE can deal with both kinds of horizontal pleiotropy, avoiding therefore potential false-positive findings. This method uses expected log pointwise posterior density (ELPD), a Bayesian approach, to compare two nested models: a sharing model where the causal effect (γ) is zero but allows for horizontal pleiotropic effects; and a causal model where γ is a free parameter.^34^ Independent variants (r^2^ <0.01; distance >250 kb) associated to the exposure with *P *<1.00e-03 were included, and the cause v1.2.0 R package was used for these analyses.^34^ A Bonferroni correction for the number of tests undertaken was used to correct for multiple testing at this stage (*P *<1.21e-03).

## Results

### Anthropometric measures

**Figure 1:**
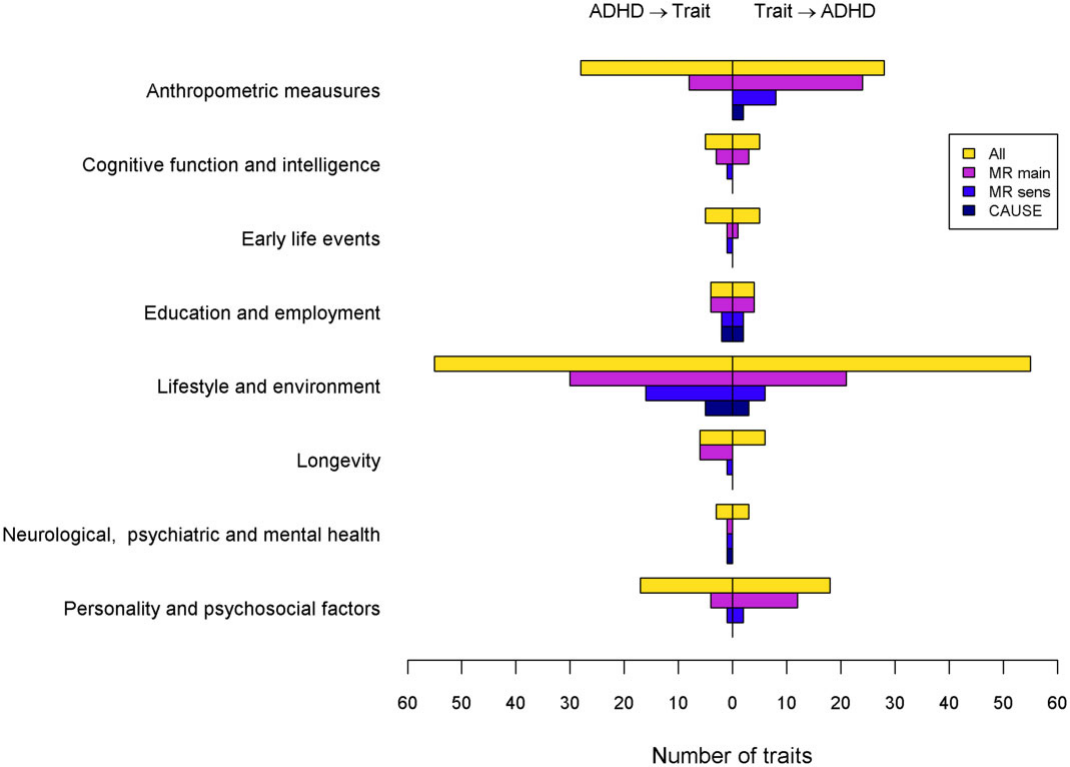
Number of traits included in each stage of the analysis for each direction and category. Total number of traits analysed (‘All’) are presented as well as number of traits meeting the significance criteria in the MR main analysis (‘MR main’), in the MR sensitivity analyses (‘MR sens’) and in CAUSE (‘CAUSE’). ADHD, attention deficit/hyperactivity disorder; CAUSE, causal analysis using summary effect estimates; MR, Mendelian randomization

**Figure 2:**
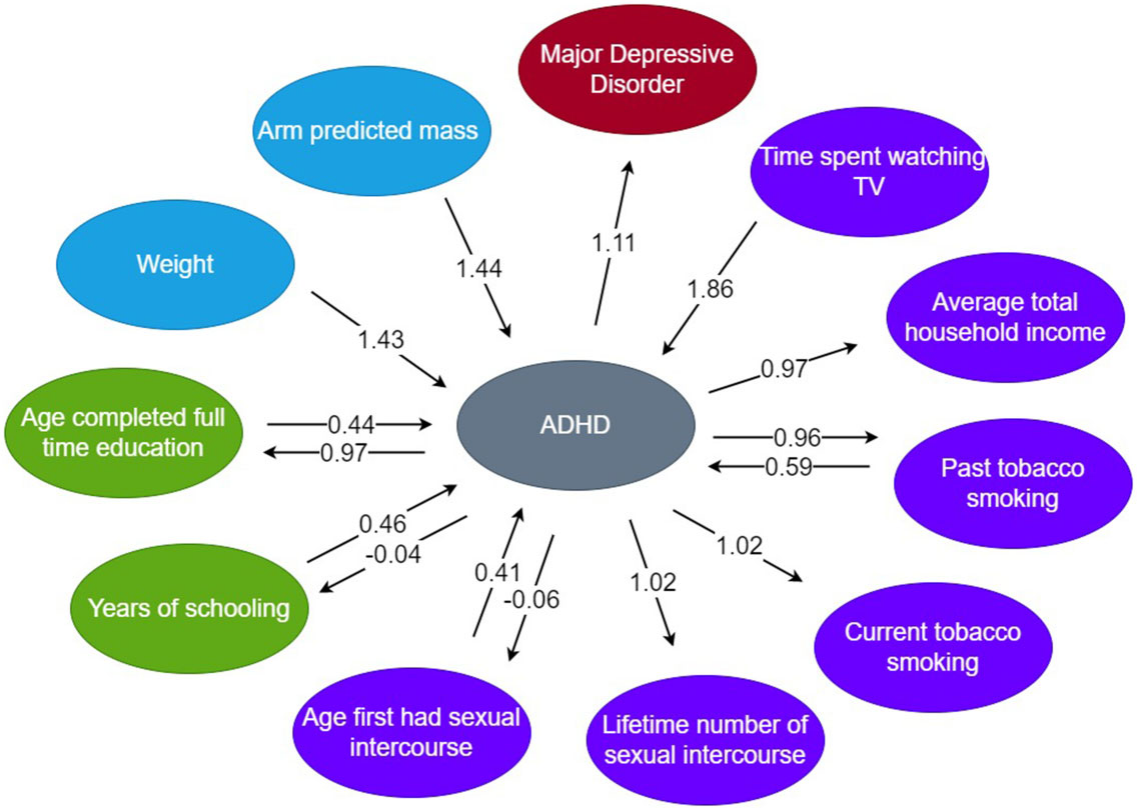
Diagram of the relationships found with consistent evidence across methods. Traits are coloured by category (education and employment in green; anthropometric measures in blue; neurological, psychiatric and mental health-related traits in red; and lifestyle and environment in purple) and CAUSE effect sizes are presented. Details on effect size units can be found in Table 2. ADHD, attention deficit/hyperactivity disorder; CAUSE, causal analysis using summary effect estimates; TV, television

The IVW analyses showed findings with FDR *P *<0.05 for one anthropometric trait when ADHD was the exposure, 17 when ADHD was considered as the outcome and seven in both directions (Figure 1; Supplementary Table S4, available as Supplementary data at *IJE* online). After sensitivity analyses there was evidence of a positive effect of the genetic liability of eight traits (arm, leg, whole body and trunk fat-free mass, arm and trunk predicted mass, whole body water mass and weight) on ADHD (Table 1), showing moderate heterogeneity (40.128%<I^2^<46.114%, Supplementary Table S5a, Figure S1, available as Supplementary data at *IJE* online). Also, evidence meeting the multiple comparison correction was found with CAUSE for a causal effect of arm predicted mass and weight on ADHD [odds ratio (OR)= 1.452, 95% CrI = (1.307, 1.614), ΔELPD *P *=* *5.32e-04 and OR = 1.430, 95% credible interval (CrI) = (1.326, 1.539), ΔELPD *P *=* *1.01e-06, respectively], with also suggestive evidence (ΔELPD *P *<0.05) of a positive effect for the remaining traits (Figure 2, Table 2).

**Table 1:** Mendelian randomization results

Trait^a^	ADHD → Trait					Trait → ADHD				
	Number of SNPs	Effect size	95% CI	FDR *P*	Meets sensitivity?	Number of SNPs	OR	95% CI	FDR *P*	Meets sensitivity?
**Anthropometric measures**										
Arm fat-free mass (left)	9	0.026	(0.005, 0.046)	3.09E-02	No	479	1.671	(1.449, 1.927)	2.00E-11	Yes
Arm predicted mass (left)	9	0.025	(0.004, 0.046)	4.38E-02	No	471	1.665	(1.438, 1.929)	1.11E-10	Yes
Whole body fat-free mass	9	0.016	(−0.006, 0.038)	2.31E-01	–	509	1.431	(1.251, 1.637)	1.13E-06	Yes
Whole body water mass	9	0.016	(−0.006, 0.038)	2.25E-01	–	518	1.456	(1.27, 1.669)	5.24E-07	Yes
Leg fat-free mass (left)	9	0.012	(−0.016, 0.04)	4.88E-01	–	460	1.461	(1.262, 1.69)	2.12E-06	Yes
Trunk fat-free mass	9	0.017	(−0.003, 0.037)	1.47E-01	–	520	1.397	(1.218, 1.602)	8.25E-06	Yes
Trunk predicted mass	9	0.017	(−0.003, 0.037)	1.58E-01	–	518	1.406	(1.225, 1.613)	6.09E-06	Yes
Weight	9	0.045	(0.002, 0.088)	7.67E-02	–	458	1.662	(1.485, 1.859)	1.54E-17	Yes
**Cognitive function and intelligence**										
Cognitive performance	9	−0.072	(−0.131, −0.013)	3.55E-02	Yes	134	0.600	(0.499, 0.722)	5.24E-07	No
**Early life events**										
Maternal smoking around birth^b^	9	1.037	(1.023, 1.052)	1.89E-06	Yes	15	143.936	(25.34, 817.581)	1.70E-07	No
**Education and employment**										
Age completed full-time education^b^	9	0.930	(0.909, 0.951)	2.52E-09	Yes	36	0.167	(0.102, 0.275)	2.00E-11	Yes
Years of schooling	9	−0.098	(−0.138, −0.059)	5.99E-06	Yes	297	0.298	(0.251, 0.354)	1.20E-41	Yes
**Lifestyle and environment **										
Alcohol intake frequency^b^	9	1.108	(1.049, 1.171)	8.51E-04	Yes	92	1.399	(1.168, 1.676)	8.51E-04	No
Alcohol intake versus 10 years previously^b^	9	1.042	(1.026, 1.058)	1.13E-06	Yes	12	8.389	(2.293, 30.696)	3.62E-03	Yes
Alcohol usually taken with meals^b^	9	0.959	(0.942, 0.976)	2.08E-05	Yes	33	0.085	(0.033, 0.221)	2.19E-06	No
Average weekly champagne plus white wine intake^b^	9	0.955	(0.936, 0.975)	4.74E-05	Yes	4	0.148	(0.041, 0.528)	7.92E-03	No
Frequency of stair-climbing in past 4 weeks^b^	9	0.954	(0.918, 0.991)	3.21E-02	Yes	17	0.383	(0.204, 0.717)	7.00E-03	No
Nitrogen dioxide air pollution 2010	9	0.032	(0.016, 0.049)	5.37E-04	Yes	8	0.610	(0.159, 2.339)	5.43E-01	–
Particulate matter air pollution (pm_2.5_) 2010	9	0.047	(0.029, 0.064)	1.55E-06	Yes	7	1.989	(0.38, 10.401)	5.01E-01	–
Age first had sexual intercourse	9	−0.130	(-0.18, -0.08)	1.74E-06	Yes	184	0.223	(0.187, 0.266)	8.34E-61	Yes
Lifetime number of sexual partners^b^	9	1.063	(1.029, 1.099)	8.61E-04	Yes	60	2.958	(1.76, 4.971)	1.63E-04	No
Current tobacco smoking^b^	9	1.041	(1.028, 1.055)	4.94E-09	Yes	32	12.305	(3.945, 38.38)	6.54E-05	No
Ever smoked^b^	9	1.032	(1.022, 1.043)	3.64E-09	Yes	73	15.251	(7.528, 30.898)	7.20E-13	No
Pack/years adult smoking as proportion of lifespan exposed to smoking	9	0.075	(0.039, 0.111)	1.73E-04	Yes	13	1.335	(0.971, 1.835)	1.28E-01	–
Past tobacco smoking^b^	9	0.905	(0.88, 0.931)	4.14E-11	Yes	87	0.381	(0.296, 0.491)	1.02E-12	Yes
Average total household income before tax^b^	9	0.909	(0.885, 0.933)	2.65E-11	Yes	44	0.329	(0.237, 0.455)	2.37E-10	No
Number of full sisters^b^	9	1.029	(1.016, 1.043)	8.78E-05	Yes	5	9.746	(0.4, 237.152)	2.38E-01	–
Townsend deprivation index at recruitment	9	0.075	(0.055, 0.095)	1.35E-12	Yes	17	5.400	(1.952, 14.941)	3.25E-03	Yes
Length of mobile phone use^b^	9	1.013	(0.975, 1.052)	5.87E-01	–	29	1.983	(1.314, 2.993)	3.14E-03	Yes
Time spent watching television (TV)^b^	9	1.030	(0.997, 1.065)	1.34E-01	–	107	3.495	(2.521, 4.847)	1.00E-12	Yes
**Longevity**										
Parental longevity (combined parental attained age, Martingale residuals)^c^	9	0.050	(0.029, 0.071)	1.07E-05	Yes	10	0.972	(0.462, 2.041)	9.62E-01	–
**Neurological, psychiatric and mental health**										
Major depressive disorder^b^	10	1.200	(1.122, 1.283)	7.12E-07	Yes	5	1.739	(1.048, 2.886)	6.25E-02	–
Personality and psychosocial factors										
Able to confide^b^	9	0.919	(0.881, 0.959)	3.51E-04	No	13	0.635	(0.46, 0.878)	1.39E-02	Yes
Frequency of tiredness/lethargy in past 2 weeks^b^	9	1.029	(1.004, 1.055)	5.16E-02	–	36	5.220	(2.809, 9.698)	1.13E-06	Yes
Frequency of unenthusiasm/disinterest in past 2 weeks^b^	9	1.034	(1.021, 1.047)	1.13E-06	yes	11	8.077	(1.753, 37.221)	1.69E-02	No

For all the traits with inverse variance weighted FDR *P* <0.05 which also met the sensitivity criteria in at least one direction, the number of SNPs included, the inverse variance weighted results and information on whether the MR sensitivity criteria were met are presented for both directions.

MR, Mendelian randomization; ADHD, attention deficit/hyperactivity disorder; SNP, single nucleotide polymorphism; CI, 95% confidence interval; FDR, false-discovery rate; *P*, *P*-value.

a The unit reported for all continuous traits is SD, except for years of schooling, which was provided in years and cognitive performance, which was provided using a cognitive score.^44^

b The causal effect size provided for the comparison with ADHD as the exposure for these traits is odds raio (OR) per ADHD log(OR) unit increase, since they were all analysed as categorical ordered, except for maternal smoking around birth, ever smoked, alcohol usually taken with meals and major depressive disorder, which were analysed as binary.

c For parental longevity (combined parental attained age, Martingale residuals) a positive effect size indicates decreased attained age.^47^

**Table 2:** Causal analysis using summary effect estimates results

**Trait^a^**	ADHD → Trait				Trait → ADHD			
	γ	95% CrI	ΔELPD (SE)	ΔELPD *P*	γ	95% CrI	ΔELPD (SE)	ΔELPD *P*
**Anthropometric measures**								
Arm fat-free mass (left)	–	–	–	–	1.441	(1.287,1.606)	−4.444 (1.555)	2.13E-03
Arm predicted mass (left)	–	–	–	–	1.452	(1.307,1.614)	−4.57 (1.396)	**5.32E-04**
Whole body fat-free mass	–	–	–	–	1.312	(1.175,1.464)	−3.275 (1.405)	9.87E-03
Whole body water mass	–	–	–	–	1.312	(1.176,1.464)	−3.14 (1.346)	9.83E-03
Leg fat-free mass (left)	–	–	–	–	1.371	(1.234,1.523)	−3.79 (1.4)	3.39E-03
Trunk fat-free mass	–	–	–	–	1.232	(1.1,1.381)	−2.51 (1.44)	4.06E-02
Trunk predicted mass	–	–	–	–	1.244	(1.108,1.398)	−2.505 (1.406)	3.74E-02
Weight	–	–	–	–	1.430	(1.326,1.539)	−5.71 (1.202)	**1.01E-06**
**Cognitive function and intelligence **								
Cognitive performance	−0.023	(−0.04,−0.006)	−2.681 (1.81)	6.92E-02	–	–	–	–
**Early life events**								
Maternal smoking around birth^b^	1.012	(1.007,1.017)	−4.886 (1.715)	2.19E-03	–	–	–	–
**Education and employment**								
Age completed full time education^b^	0.972	(0.962,0.981)	−5.655 (1.641)	**2.85E-04**	0.435	(0.356,0.533)	−6.159 (1.349)	**2.51E-06**
Years of schooling	−0.036	(−0.048,-0.024)	−6.5 (1.723)	**8.04E-05**	0.458	(0.411,0.511)	−7.184 (1.131)	**1.08E-10**
**Lifestyle and environment **								
Alcohol intake frequency^b^	1.033	(1.017,1.049)	−5.103 (2.079)	7.04E-03	–	–	–	–
Alcohol intake versus 10 years previously^b^	1.010	(1.003,1.016)	−2.66 (1.813)	7.12E-02	2.099	(1.294,3.384)	−3.324 (1.814)	3.34E-02
Alcohol usually taken with meals^b^	0.986	(0.98,0.993)	−4.543 (1.8)	5.80E-03	–	–	–	–
Average weekly champagne plus white wine intake^b^	0.985	(0.977,0.994)	−3.654 (1.905)	2.75E-02	–	–	–	–
Frequency of stair climbing in last 4 weeks^b^	0.991	(0.979,1.002)	−0.55 (1.288)	3.35E-01	–	–	–	–
Nitrogen dioxide air pollution 2010	0.003	(-0.005,0.012)	0.653 (0.47)	9.18E-01	–	–	–	–
Particulate matter air pollution (pm_2.5_) 2010	0.008	(-0.001,0.016)	−0.936 (1.484)	2.64E-01	–	–	–	–
Age first had sexual intercourse	−0.058	(-0.072,−0.044)	−6.939 (1.562)	**4.47E-06**	0.413	(0.372,0.457)	−7.797 (1.105)	**8.70E-13**
Lifetime number of sexual partners^b^	1.023	(1.013,1.033)	−5.869 (1.911)	**1.06E-03**	–	–	–	–
Current tobacco smoking^b^	1.015	(1.01,1.021)	−5.488 (1.703)	**6.35E-04**	–	–	–	–
Ever smoked^b^	1.011	(1.006,1.016)	−5.368 (1.974)	3.27E-03	–	–	–	–
Pack years adult smoking as proportion of life span exposed to smoking	0.034	(0.019,0.047)	−4.232 (1.751)	7.81E-03	–	–	–	–
Past tobacco smoking^b^	0.963	(0.947,0.978)	−5.747 (1.829)	**8.37E-04**	0.588	(0.523,0.664)	−5.685 (1.399)	**2.41E-05**
Average total household income before tax^b^	0.966	(0.954,0.979)	−5.777 (1.809)	**7.01E-04**	–	–	–	–
Number of full sisters^b^	1.007	(0.999,1.013)	−1.207 (1.546)	2.17E-01	–	–	–	–
Townsend deprivation index at recruitment	0.024	(0.015,0.033)	−4.996 (1.701)	1.65E-03	1.870	(1.38,2.522)	−3.676 (1.546)	8.72E-03
Length of mobile phone use^b^	–	–	–	–	1.281	(1.084,1.516)	−2.874 (1.805)	5.56E-02
Time spent watching television (TV)^b^	–	–	–	–	1.862	(1.545,2.246)	−4.491 (1.475)	**1.16E-03**
**Longevity**								
Parental longevity (combined parental attained age, Martingale residuals)^c^	0.017	(0.007,0.026)	−3.663 (1.873)	2.52E-02	–	–	–	–
**Neurological, psychiatric and mental health**								
Major depressive disorder^b^	1.110	(1.073,1.148)	−5.828 (1.666)	**2.34e-04**	–	–	–	–
**Personality and psychosocial factors**								
Able to confide^b^	–	–	–	–	0.864	(0.739,1.012)	−0.836 (1.416)	2.77E-01
Frequency of tiredness/lethargy in past 2 weeks^b^	–	–	–	–	1.772	(1.388,2.25)	−3.717 (1.523)	7.34E-03
Frequency of unenthusiasm/disinterest in past 2 weeks^b^	1.013	(1.007,1.019)	−5.665 (1.982)	2.13E-03	–	–	–	–

For all analyses that met the MR sensitivity criteria, CAUSE results are presented, including an estimate of the causal effect size (γ) with 95% credible intervals and the difference between the ELPD in the causal and in the sharing models (**ΔELPD**) with its standard error and one-sided *P*-value. *P*-values meeting a Bonferroni corrected threshold are highlighted in bold (0.05/41=1.21E-03).

MR, Mendelian randomization; CAUSE, causal analysis using summary effect estimates; ADHD, attention deficit/hyperactivity disorder; Crl, credible interval; ELPD, expected log pointwise posterior density; SE, standard error; *P*, *P*-value.

a The unit reported for all continuous traits is SD, except for years of schooling, which was provided in years and cognitive performance, which was provided using a cognitive score.^44^

b The causal effect size (γ) provided for these traits is odds ratio (OR) per ADHD log(OR) unit increase when ADHD is the exposure, since they were all analysed as categorical ordered, except for maternal smoking around birth, ever smoked, alcohol usually taken with meals and major depressive disorder, which were analysed as binary.

c For parental longevity (combined parental attained age, Martingale residuals) a positive effect size indicates decreased attained age.^47^

### Cognitive function and intelligence

In the IVW analysis, a negative effect of ADHD genetic liability on mean time to correctly identify matches, a measure of raw processing and reaction speed, was detected as well as a negative effect of intelligence when ADHD was the outcome, and for cognitive performance and fluid intelligence in both directions (IVW FDR *P *<0.05) (Table 1; Supplementary Table S4; Figure 1). Of them, the effect of ADHD as exposure on cognitive performance survived the sensitivity analyses, but showed high heterogeneity (I^2^ = 84.231%, Supplementary Table S5b, Supplementary Figure S1a–h). When outliers were removed in the MR-PRESSO analysis, the magnitude of the causal effect estimate increased slightly (Supplementary Table S5b,) and the heterogeneity went down to an I^2^ of 68.844%. The CAUSE analysis did not show evidence for the effect of ADHD liability on cognitive performance (ΔELPD *P *=* *0.069, Table 2).

### Early life events

Out of all early life exposure traits, a positive effect between maternal smoking around birth and ADHD was identified in both directions in the IVW analysis; but only when ADHD was considered as exposure were the sensitivity criteria met (Figure 1; Supplementary Table S4). There was moderately high heterogeneity for this analysis (I^2^ = 63.896%), and although removing one outlier in the MR-PRESSO analysis reduced the heterogeneity and the causal effect estimate, measured as odds of maternal smoking per unit increase in log odds ratio [log(OR)] of ADHD, it remained significant [from OR = 1.037, 95% confidence interval (CI) = (1.023, 1.052) to OR = 1.03, 95% CI = (1.018, 1.043), I^2^ = 36.476%, Supplementary Table S5c, Supplementary Figure S1am]. The effect of ADHD on maternal smoking around birth, however, did not survive multiple comparisons correction with CAUSE (**Δ**ELPD *P *=* *2.19e-03, Table 2).

### Education and employment

The IVW analysis detected results with FDR *P *<0.05 in both directions for all traits analysed, with a negative effect for age completed full-time education and years of schooling and a positive effect for job involving heavy manual, physical work or mainly walking or standing (Figure 1, Table 1; Supplementary Table S4). Only age completed full-time education and years of schooling met the sensitivity analysis criteria in both directions. However, when ADHD was the outcome, the Steiger analysis provided evidence for reverse causation,with reduced effect sizes after filtering, which suggests that at least part of the observed effect was due to ADHD liability causing the educational outcomes (Supplementary Table S5d). The heterogeneity for these analyses was moderate (I^2^ <50%) except for the effect of ADHD liability on years of schooling, which was considerable (I^2^ = 87.572%, Supplementary Table S5d). After excluding outliers, MR-PRESSO results remained significant (Supplementary Table S5d, Supplementary Figure S1au) and the heterogeneity decreased, although it remained substantial (I^2^ = 67.755%). CAUSE confirmed these results, providing strong evidence for a causal effect per unit increase in the log(OR) of ADHD on odds of decreased age completed full-time education and years of schooling [OR = 0.972, 95% CrI = (0.962, 0.981), **Δ**ELPD *P *=* *2.85e-04 and beta = -0.036, 95% CrI = (-0.048, -0.024), **Δ**ELPD *P *=* *8.04e-05, respectively] (Figure 2, Table 2). Evidence in the opposite direction was also found, with causal effect per unit increase in odds of increased age completed full-time education and years of schooling on ADHD odds [OR = 0.435, 95% CrI = (0.356, 0.533), ΔELPD *P *=* *2.51e-06 and OR = 0.458, 95% CrI = (0.411, 0.511), ΔELPD *P *=* *1.08e-010, respectively] (Figure 2, Table 2).

### Lifestyle and environment

In the IVW analysis 12 lifestyle and environment traits had FDR *P *<0.05 when ADHD was the exposure, three when ADHD was the outcome and 18 in both directions (Figure 1; Supplementary Table S4). After the sensitivity analyses, we found evidence for an effect of ADHD as exposure on traits related to alcohol use (increased alcohol intake frequency, alcohol intake versus 10 years previously, decreased alcohol usually taken with meals and average weekly champagne plus white wine intake), physical exercise (decreased frequency of stair-climbing in past 4 weeks), air pollution (increased nitrogen dioxide and particulate matter), sexual behaviour (decreased age first had sexual intercourse and increased lifetime number of sexual partners), smoking (increased current tobacco smoking, ever smoking and pack/years adult smoking, and decreased past tobacco smoking) and sociodemographic information (decreased average total household income and increased Townsend deprivation index and number of sisters) (Table 1). Alcohol intake versus 10 years previously, age of first sexual intercourse, past tobacco smoking and Townsend deprivation index also had an effect when ADHD was used as outcome, as did length of mobile phone use and time spent watching television (Table 1).

When ADHD was considered as outcome, all analyses that met the sensitivity criteria also showed evidence of reverse causation, with smaller effect sizes after Steiger filtering particularly for Townsend deprivation index, suggesting that at least some the observed effect was due to ADHD liability causing the lifestyle and environmental outcomes (Supplementary Table S5e). Also, high heterogeneity (I^2^ >70%) was detected for alcohol intake frequency, age of first sexual intercourse and lifetime number of sexual partners when ADHD was the exposure and for Townsend deprivation index in the other direction (Supplementary Figure S1s, af, ah, au, Supplementary Table S5e). After removing outliers there was still evidence of a causal effect and the heterogeneity decreased, although it remained around 60% for age of first sexual intercourse, lifetime number of sexual partners and Townsend deprivation index (Supplementary Table S5e).

After multiple comparisons correction, CAUSE analyses provided evidence with ADHD as the exposure for lower odds of increased average total household income [OR = 0.966, 95% CrI = (0.954, 0.979)], higher odds of a increased lifetime number of sexual partners [OR = 1.023, 95% CrI = (1.013, 1.033)] and a negative effect in both directions for age first had sexual intercourse [beta = −0.058, 95% CrI = (−0.072, −0.044) and OR = 0.413, 95% CrI = (0.372, 0.457) for ADHD as exposure and outcome, respectively] (Figure 2, Table 2). Evidence was also found for a positive effect of ADHD liability on current tobacco smoking [OR = 1.015, 95% CrI = (1.01, 1.021)], and a negative effect for past tobacco smoking in both directions [OR = 0.963, 95% CrI = (0.947, 0.978), OR = 0.588, 95% CrI = (0.523, 0.664) for ADHD as exposure and outcome, respectively] (Figure 2, Table 2). In addition, time spent watching television seemed to increase significantly the odds of ADHD [OR = 1.862, 95% CrI = (1.545, 2.246)] (Figure 2, Table 2). Suggestive evidence (ΔELPD *P *<0.05) was shown in the CAUSE analysis for a causal effect of the genetic liability of ADHD on the remaining smoking-related traits and on all alcohol-related traits, except for alcohol intake versus 10 years previously, which showed an effect in the opposite direction, and for Townsend deprivation index, which showed an effect in both directions (Table 2).

### Longevity

In the IVW analysis, there was no evidence of a causal effect when ADHD was considered as outcome, but evidence for an effect of ADHD as exposure was found in all longevity-related traits, decreasing maternal, paternal and combined age of death or attained age and decreasing the odds of both parents being in the top 10% of survival (Figure 1, Table 1; Supplementary Table S4). However, only effects on combined parental attained age met the sensitivity analysis criteria and showed suggestive evidence of a causal relationship in the CAUSE analysis, although it did not meet multiple testing correction (ΔELPD *P *=* *0.025, Table 2).

### Neurological, psychiatric and mental health-related traits

We found evidence of an increase in the odds of major depression per unit increase in the log(OR) of ADHD in the IVW analysis [OR = 1.200, 95% CI=(1.122, 1.283), Table 1], which survived the sensitivity analyses and also met the Bonferroni correction for multiple testing in CAUSE [OR = 1.110, 95% CrI=(1.073, 1.148), ΔELPD *P *=* *2.34e-04] (Figure 2, Table 2). An alternative and more broad definition of depression, however, did not reach statistical significance at any stage, and neither did the other analyses undertaken (Figure 1; Supplementary Table S4).

### Personality and psychosocial factors

In the IVW analysis, we found evidence of an effect for ADHD as exposure on one trait, with ADHD as outcome for nine traits and in both directions for three traits (Supplementary Table S4; Figure 1). The effect of the genetic liability of ADHD on frequency of unenthusiasm/disinterest in past 2 weeks met the sensibility analysis criteria, but was only suggestive after multiple comparison correction in the CAUSE analysis (**Δ**ELPD *P *=* *2.13e-03, Table 2) and evidence of reverse causation was found in the opposite direction (Supplementary Table S5h). The effect of the genetic liability of frequency of tiredness/lethargy in past 2 weeks and of being able to confide on ADHD remained after the sensitivity analyses, but only suggestive evidence was found for being able to confide in the CAUSE analyses (ΔELPD *P *=* *7.34e-03, Table 2).

**Figure 3:**
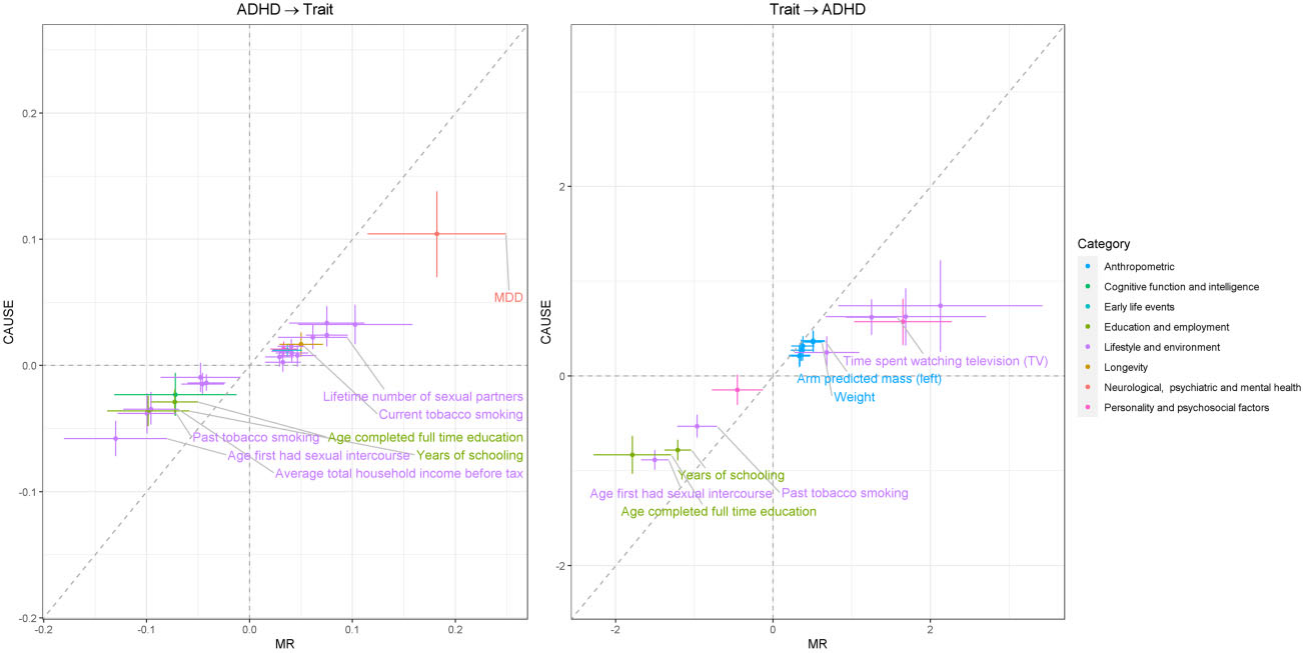
Causal effect estimates obtained by MR, with 95% CI, vs those obtained by CAUSE, with 95% CrI, in both directions. Effect sizes are provided as logOR for binary and categorical ordered traits. Different categories have been plotted in different colours, and traits with results meeting the Bonferroni corrected threshold in CAUSE are labelled. ADHD, attention deficit/hyperactivity disorder; CAUSE, causal analysis using summary effect estimates; CI, confidence interval; CrI, credible interval; MDD, major depressive disorder; MR, Mendelian randomization; TV, television; OR, odds ratio

Overall, the direction of the causal effect size estimates was consistent between IVW MR and CAUSE for all analyses undertaken and, in general, CAUSE effect size estimates were smaller than those estimated by IVW MR (Figure 3, Tables 1 and 2).

## Discussion

In the present study we assessed for the first time the causal relationships between ADHD and a broad range of ADHD-related traits, applying complementary approaches. Through this comprehensive strategy we found consistent evidence across methods for a causal effect of the genetic liability of anthropometric measures and time spent watching television on ADHD, for the genetic liability of ADHD on average household income and major depressive disorder, and a bidirectional relationship with educational achievement, smoking and sexual behaviour.

Our findings give support to the relationship between ADHD and risk-taking behaviours and to existing evidence indicating that ADHD is an entry point into a harmful life trajectory, where ADHD individuals are more likely to engage in behaviours that put them at risk for negative outcomes, including smoking, problematic substance use or unsafe sexual behaviour.^58^^,^^59^ We confirm previous findings for a causal effect of the genetic liability of ADHD on smoking behaviour.^29^ In particular, we found a positive effect of ADHD liability on frequency of current smoking and a negative effect on past tobacco smoking, which indicates lower frequency of smoking in the past for individuals who were not heavy smokers when asked. These results may suggest that those individuals who were heavy smokers in the past carried on being heavy smokers when asked, and support that ADHD genetic liability may increase frequency of smoking and make smokers less likely to give up, which agrees with a reported negative effect of ADHD liability in smoking cessation.^29^ A recent MR study, however, did not find evidence for a causal relationship from liability for ADHD to nicotine dependence, although their sample size was more limited and these results may just reflect lack of statistical power.^60^ We also found suggestive evidence for an effect of the genetic liability of ADHD increasing smoking initiation and pack/years of smoking that support previous findings.^29^^,^^30^ Also, despite not surpassing the strict Bonferroni correction applied, suggestive evidence of a causal effect of the genetic liability of ADHD increasing alcohol intake frequency and making individuals less likely to have their alcohol intake with meals, was found, which suggests increased risk for prejudicial use of alcohol. Although previous studies reported a weak effect of liability to ADHD on alcohol dependence, they failed to find a causal connection between ADHD and alcohol amount or alcohol use disorder.^29^ Differences between the traits considered, sample sizes or the way of measuring alcohol consumption may account for inconsistent findings among studies. With respect to sexual behaviour, we show, for the first time, evidence for a causal role of the genetic liability to ADHD on lower age at first sexual intercourse and on increased lifetime number of sexual partners. In line with all these results, and with additional evidence from observational literature, we also found suggestive evidence for a causal effect of ADHD genetic liability on decreased longevity.^14^

Our results also support a positive effect of time spent watching television on ADHD, which goes in line with a reported association between ADHD and television usage and with evidence from longitudinal studies reporting an effect of increased screen time worsening a child’s development and increasing risk for autism spectrum disorder.^61–63^

In addition, our findings strengthen previous evidence linking ADHD with academic, employment and financial problems.^17^^,^^64^ In fact we report, for the first time, consistent evidence for a negative causal effect of ADHD liability on years of schooling, age completed full-time education and average total household income before tax, and suggestive evidence of a positive effect on Townsend deprivation index at recruitment. In addition, evidence was found in the other direction for the educational traits and suggestive evidence for Townsend deprivation index. Given that ADHD is a neurodevelopmental disorder, the latest relationships are temporally implausible and results may reflect previous studies showing that children in families with lower parental education, family income or socioeconomic status are at higher risk for ADHD or ADHD symptoms.^6^^,^^65^^,^^66^ This is consistent with dynastic effects, when genetic variants in parents may affect the next generation indirectly through their effect on the environment rather than through the inherited DNA, affecting our results.^67^ For instance, it might be that parent’s socioeconomic status could influence parenting skills, social development or stress levels and these, in turn, may impact on children’s mental health.^66^

Along these lines, we also found evidence for liability of lower age at first sexual intercourse and of lower rate of past tobacco smoking in non-heavy smokers increasing the odds of ADHD. Evidence from the literature has linked related traits such as young parental age or maternal smoking with increased risk of ADHD in children,^5^^,^^68^^,^^69^ suggesting that these chronological implausible results may also be driven by dynastic effects.

We also show evidence consistent with a positive effect of anthropometric measures on ADHD, a finding which is likely related to the effect of BMI liability on ADHD, reported in a previous MR study.^23^ Later studies with longitudinal and family designs, however, pointed to this relationship being largely explained by a variety of psychosocial factors and shared genetic and environmental confounders, also including a role for parental education dynastic effects.^24^^,^^25^

Our findings also confirm the effect of ADHD liability on major depression and the lack thereof when using a broader definition,^22^ but no evidence supporting previous results of a causal relationship of ADHD and birthweight or intelligence was found. These discrepancies may be explained by methodological differences between studies, including: (i) the selection of genetic instruments and additional covariates taken into account by authors considering birthweight^18^; or (ii) differences in sensitivity analyses undertaken when the protective effect of intelligence on ADHD was described.^19^

The results of the present study should be interpreted in the light of several limitations, as follows.

- Given that we aimed to give an overview of potential causal relationships between ADHD and a considerable number of related traits, using publicly available summary statistics datasets, it was not feasible to tailor the analytical strategy separately for each trait or to carefully curate each phenotype. This may have prevented us from identifying additional evidence for causal relationships, as may be the case for birthweight mentioned above,^18^ but also may have led to some spurious findings due to instrument mis-specification.
- Aiming to avoid false-positive results, we designed a strict analysis pipeline. We undertook a comprehensive set of sensitivity analyses, including the weighted mode, recently reported to maintain the correct type I error rate in a diverse set of scenarios but also to be too conservative, particularly for large sample sizes.^70^ In addition, we applied a strict multiple testing correction, despite the presence of correlated traits.
- Despite undertaking this range of different analyses, each one under a different set of assumptions, and selecting only results that were consistent across methods, we still identified some temporally implausible relationships. These associations could be explained statistically because the instruments were used as measures of the liability for a trait, not necessarily its observed manifestation,^70^ although they may also indicate the invalidity of the genetic instruments. As discussed above, some of them, such the effect of socioeconomic status or smoking behaviour liability on ADHD, are likely driven by dynastic effects. In addition, for those traits with evidence of bidirectional causality, we cannot rule out a scenario where most of the heritable variation of both exposure and outcome is mediated by the same unobserved process, as acknowledged by CAUSE authors.^34^
- Some of the scenarios where MR sensitivity analyses have been carried out may not have been optimal for their performance: for instance, the limited number of genetic instruments available for ADHD (particularly relevant for MR Egger) or the difference in sample size between the exposure and the outcome in some comparisons (relevant for Steiger filtering). It may be that under more suitable circumstances, MR sensitivity analyses would be more efficient in detecting false-positive results.
- In addition, genetic variants could be associated with more than one trait, which would make it difficult to ascertain which one is the true causal exposure. This is particularly relevant when analysing correlated traits, as it is the case in this study. Further sensitivity analyses, which were out of the scope of this study, excluding variants associated with other traits or undertaking mediation analysis would contribute to deepen our understanding and provide more robust evidence for the causal relationships identified. For instance, multivariable MR analyses have been used to detect an effect of educational attainment mediating the relationship between BMI and ADHD.^25^
- We must be cautious when comparing effect sizes between analyses with ADHD as exposure and outcome, since they are often presented in different scales, and there are a number of assumptions that need to hold for reliable interpretation of causal effects for binary exposures.^71^ Also, due to differences in sample size, the power was often different between analyses, which in turn makes it difficult to establish a prevailing direction of causality for traits with a bidirectional effect.
- There are some methodological issues that should also be considered. Given the large resources of GWAS summary statistics currently available and the flourishment of MR-related methods being developed, there is a huge potential for MR analyses to shed light into the causal relationships between many complex traits. We should, however, also bear in mind their limitations when designing studies and interpreting results. In order to avoid the effect of horizontal pleiotropy, which is relevant given that emerging results from GWAS point to a large number of genetic variants being associated with multiple traits,^51^^,^^71^ we followed up MR findings with CAUSE.^34^ Overall we observed consistency in direction of effects between both methods, with smaller effect sizes estimated by CAUSE than by MR and narrower intervals in general. The difference in effect sizes may be due to CAUSE also accounting for horizontal pleiotropy in its model. Although, CAUSE authors’ also acknowledge that in the event of causality and no correlated pleiotropy, their causal estimates tend to shrink towards zero in comparison with other methods, partly due to prior distribution being centred at zero.^34^ Under these circumstances they also report lower mean squared error for CAUSE compared with MR if causal effects are small, and there is also low power on the exposure, which seems to be the case when we consider ADHD as exposure.^34^ Overall, despite the use of a range of complementary approaches in this study and of the evidence provided for causal relationships supported by the literature and by alternative study designs, such as the effect of ADHD on depression,^22^ it seems that some of our results may still have been affected by biases such as dynastic effects. This highlights the caution that must still be exerted when interpreting MR findings and the need for other studies with alternative designs, such as those in families,^72^ to triangulate their findings and confirm MR results.

## Conclusion

Our results are consistent with a causal effect of the genetic liability of ADHD on average household income and major depressive disorder, of the genetic liability of anthropometric measures and time spent watching television on ADHD, and of a bidirectional relationship with educational achievement, smoking and sexual behaviour. Additional analyses with complementary study designs to avoid the effect of potential biases will be required to follow up these findings. However, our results may still contribute to explain part of the widespread co-occurring traits and comorbid disorders across the lifespan of individuals with ADHD, and may open new opportunities for developing preventive strategies for ADHD and for negative ADHD trajectories.

## Ethics approval

Ethics approval was not required for this study, since no new data were generated and only summary statistics were analysed.

## Supplementary Material

dyac128_Supplementary_Data

## Data Availability

The data underlying this article are available in the MR-Base database (doi: 10.1101/2020.08.10.244293 and doi : 10.7554/eLife.34408.001).
