# Supplementary material for: Mendelian randomization analysis for attention deficit/hyperactivity disorder: studying a broad range of exposures and outcomes
Source: Int J Epidemiol. 2022 Jun 12;52(2):386–402. doi: 10.1093/ije/dyac128 (PMC10114062; doi:10.1093/ije/dyac128)

Supplementary Figure S1 Plots for MR sensitivity analyses

For all analyses with IVW FDR P<0.05 this figure presents: scatter plots for the exposure vs. the outcome effect size estimates (using logOR for categorical ordered and binary traits) for the different MR methods used, as well as funnel, forest and leave-one-out plots for the IVW analysis.

a) Arm fat−free mass (left) → ADHD

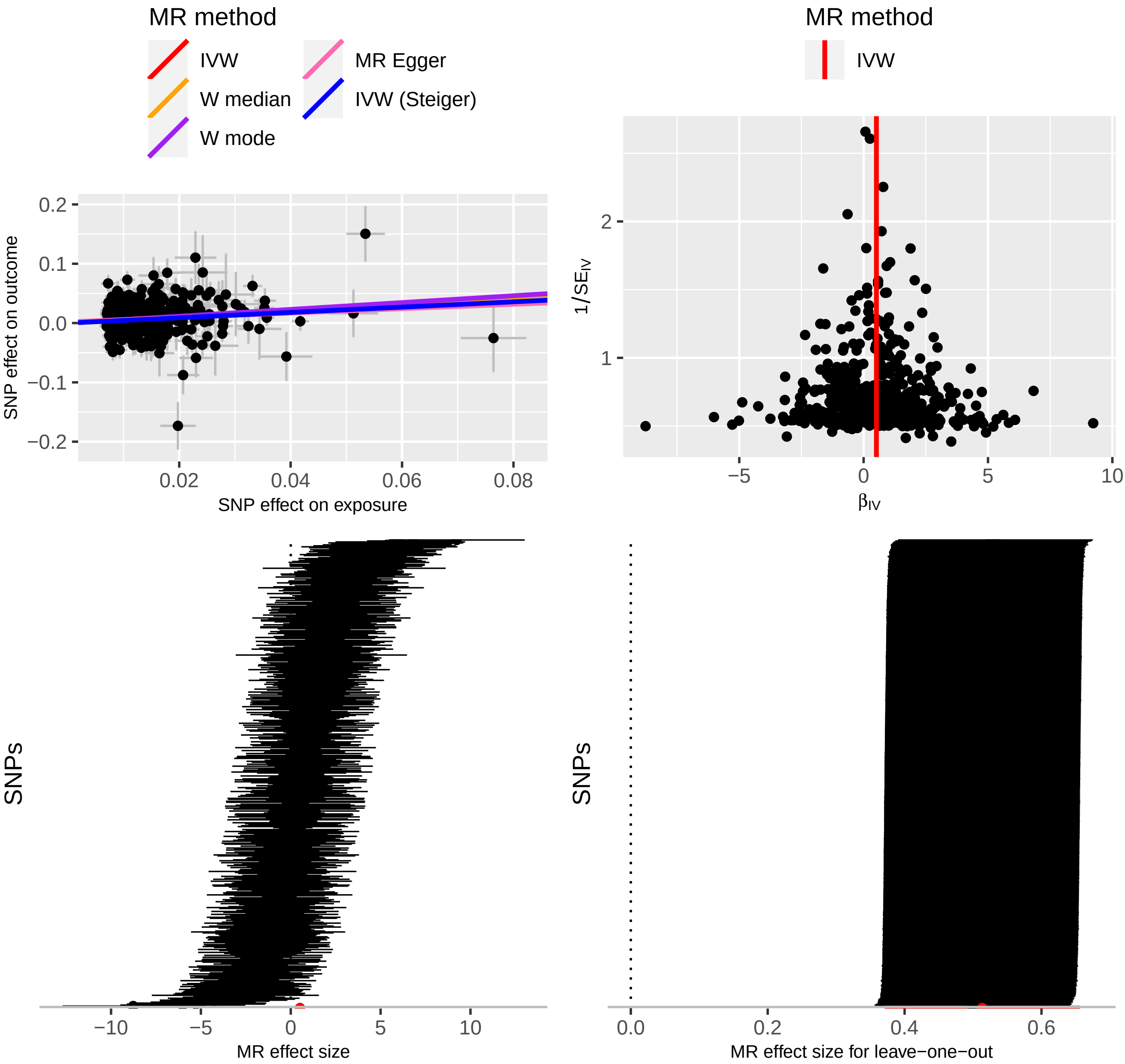

b) ADHD → Arm fat−free mass (left)

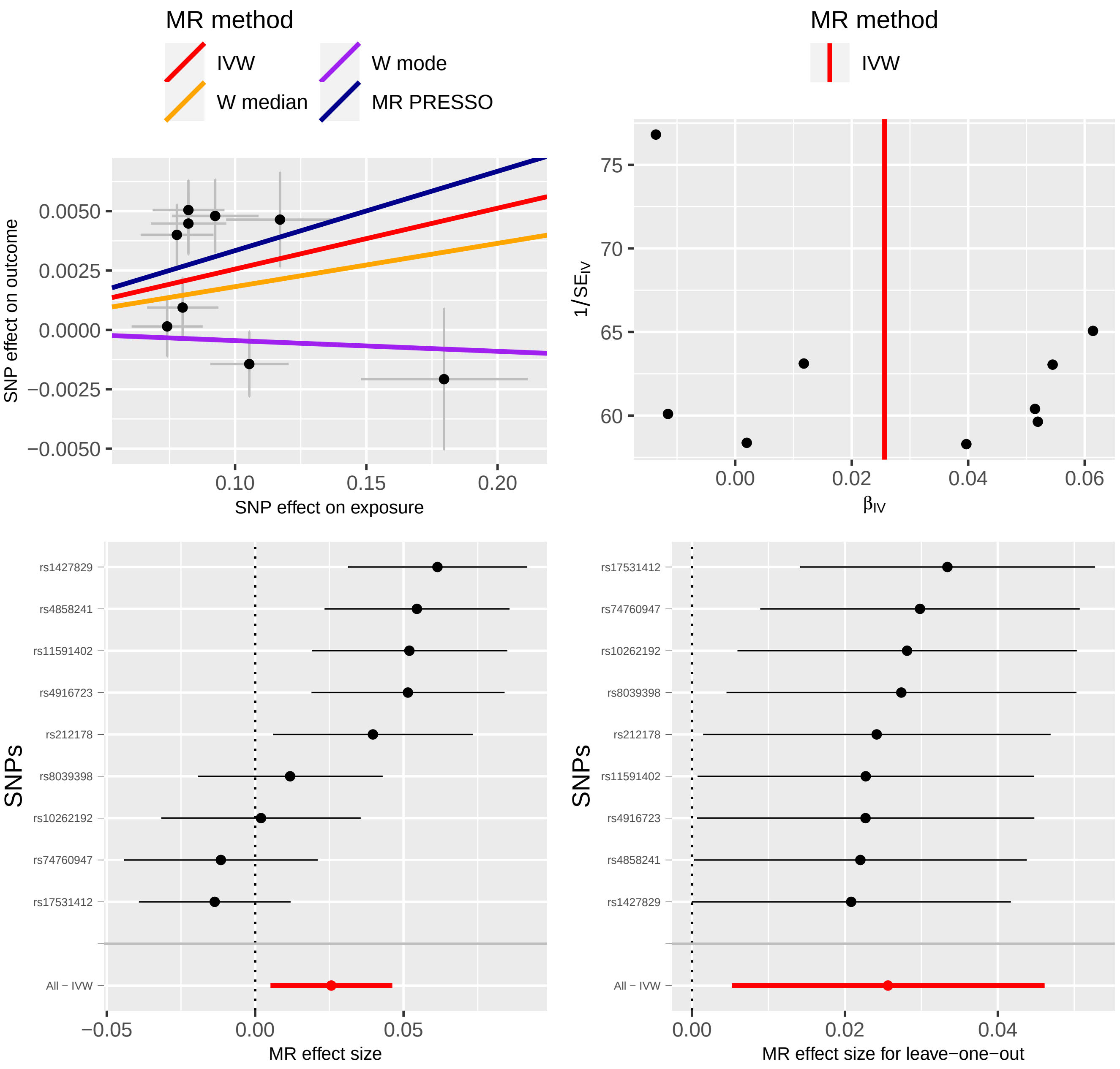

c) Arm fat mass (left) → ADHD

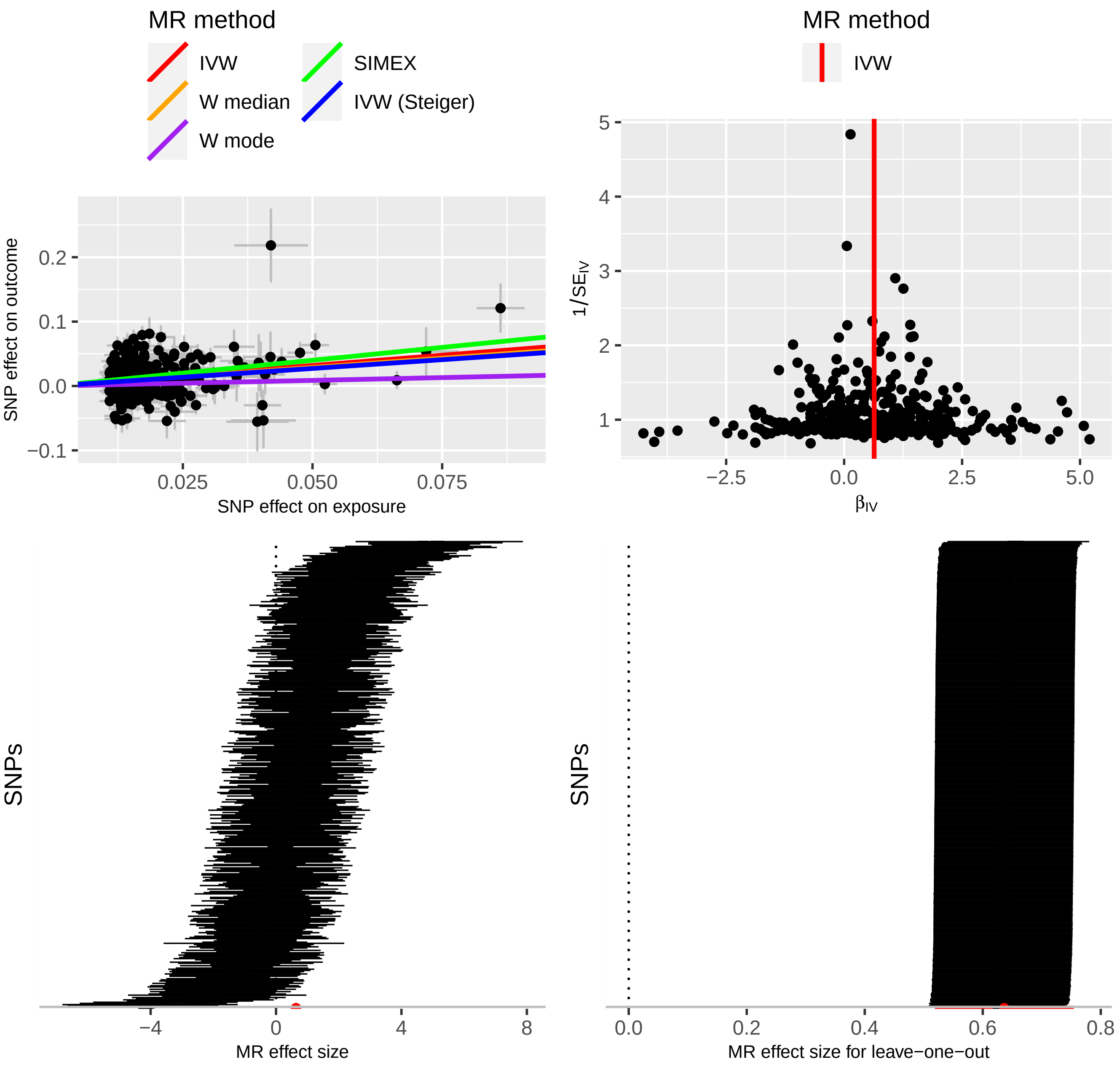

d) Arm fat percentage (left) → ADHD

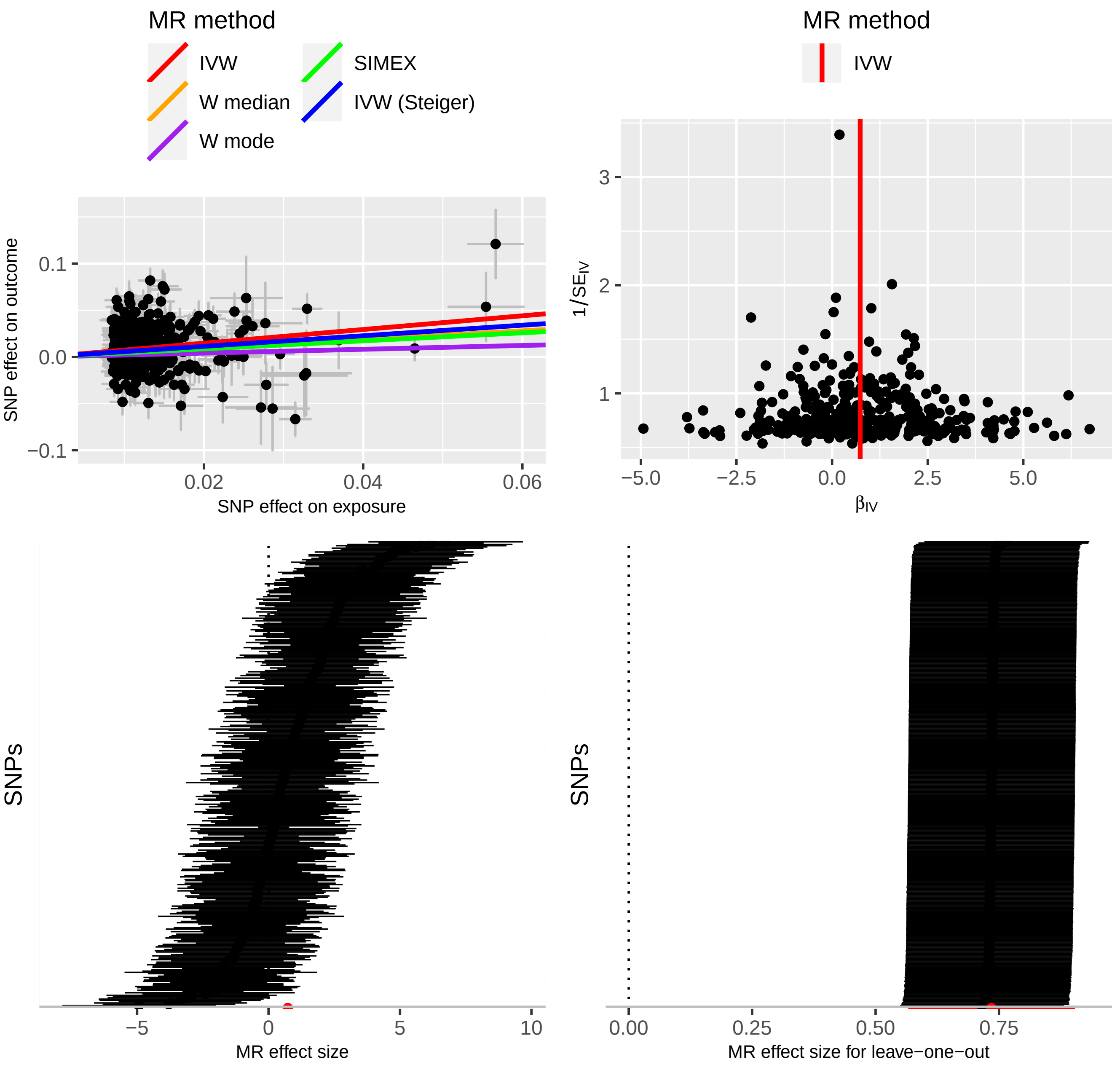

e) Arm predicted mass (left) → ADHD

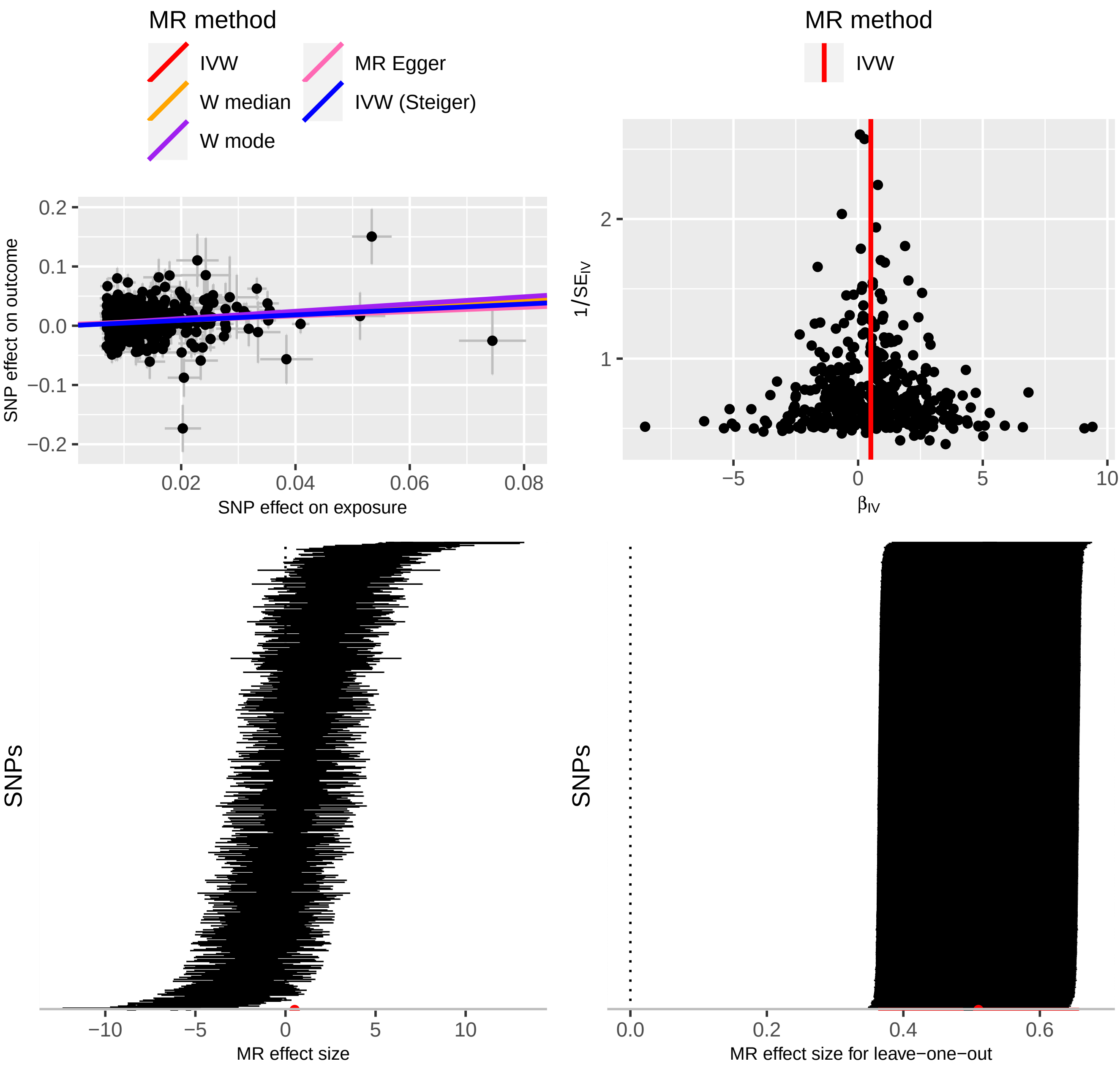

f) ADHD → Arm predicted mass (left)

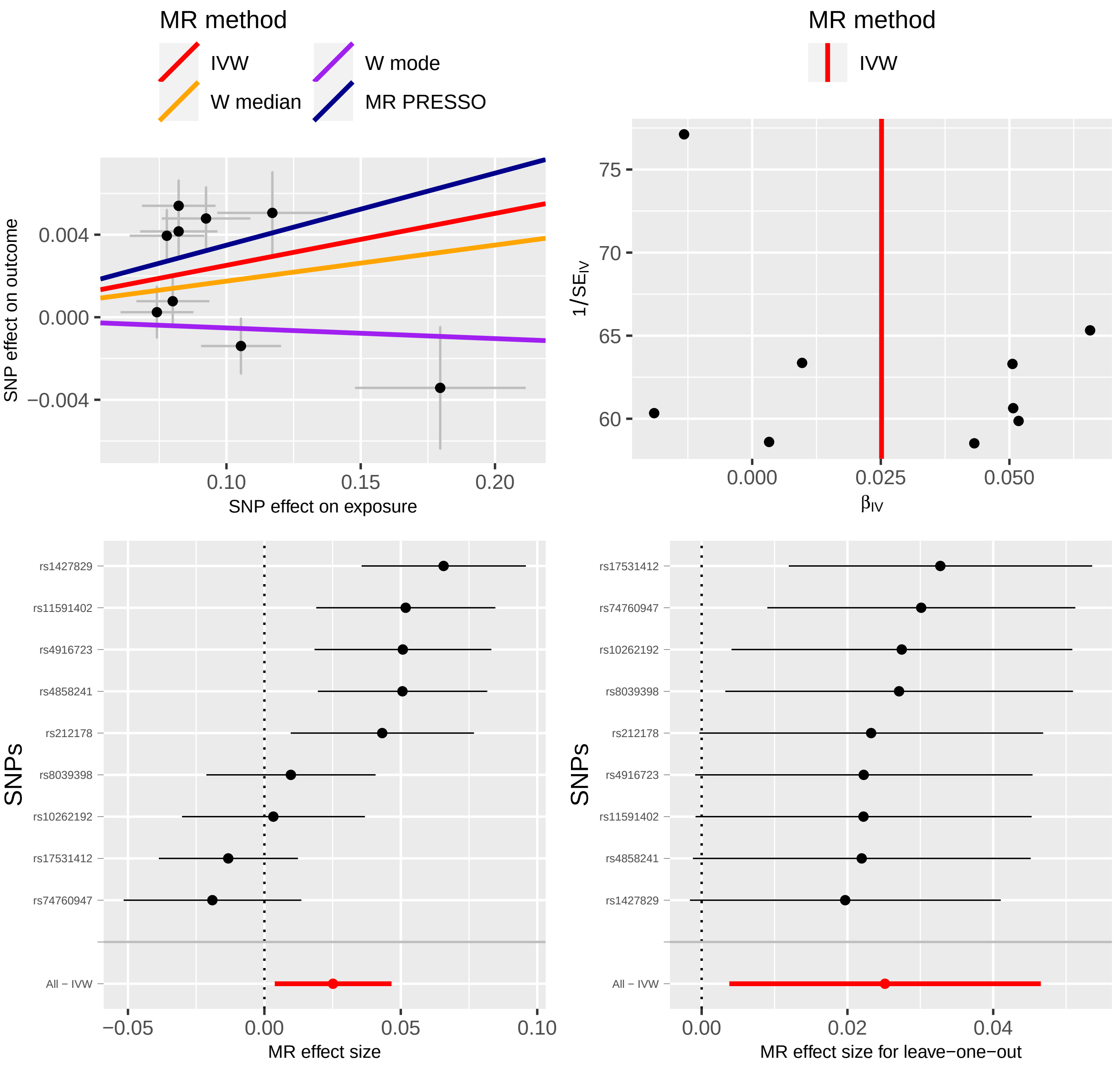

g) Impedance of arm (left) → ADHD

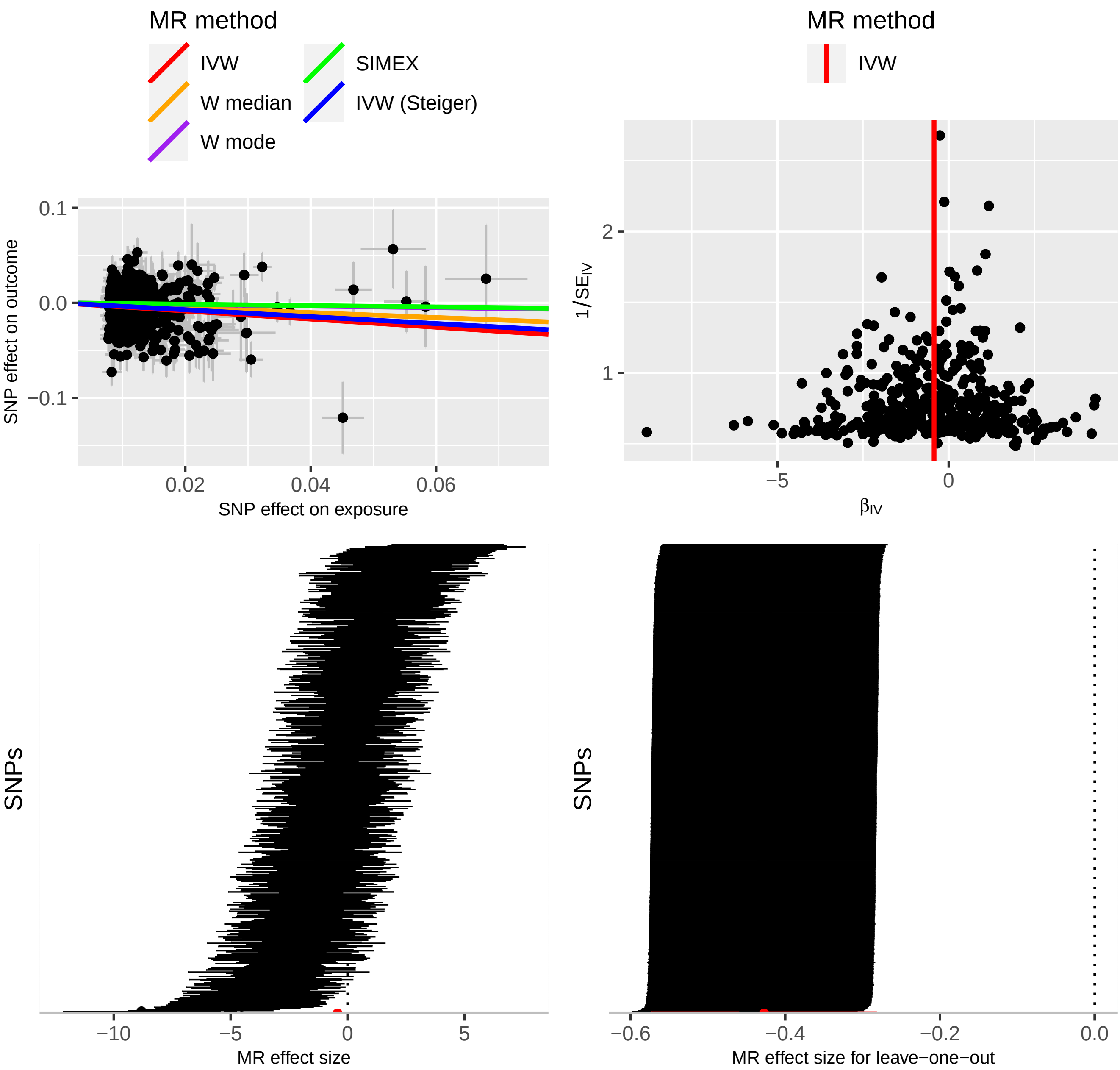

h) ADHD → Impedance of arm (left)

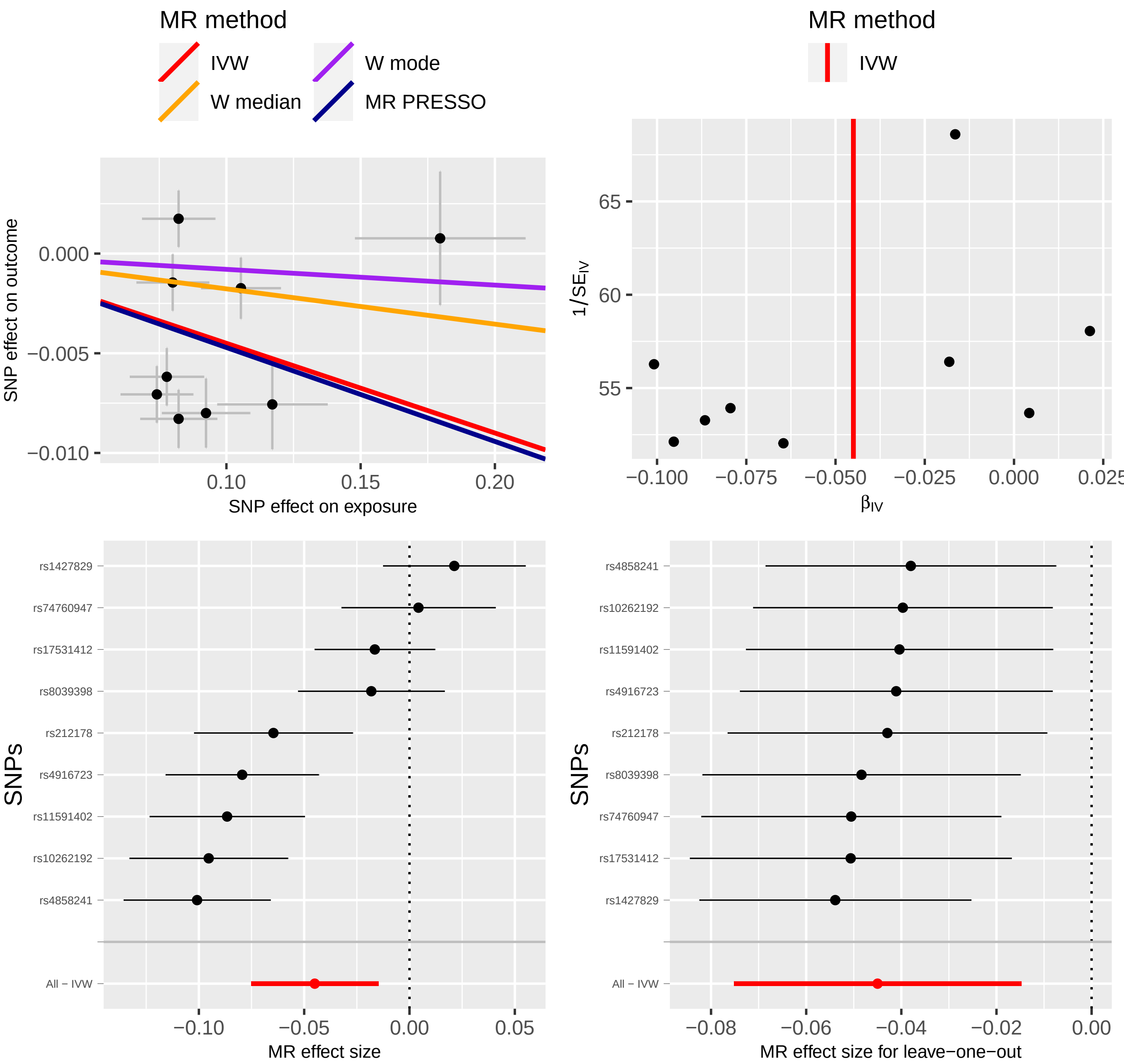

i) Body fat percentage → ADHD

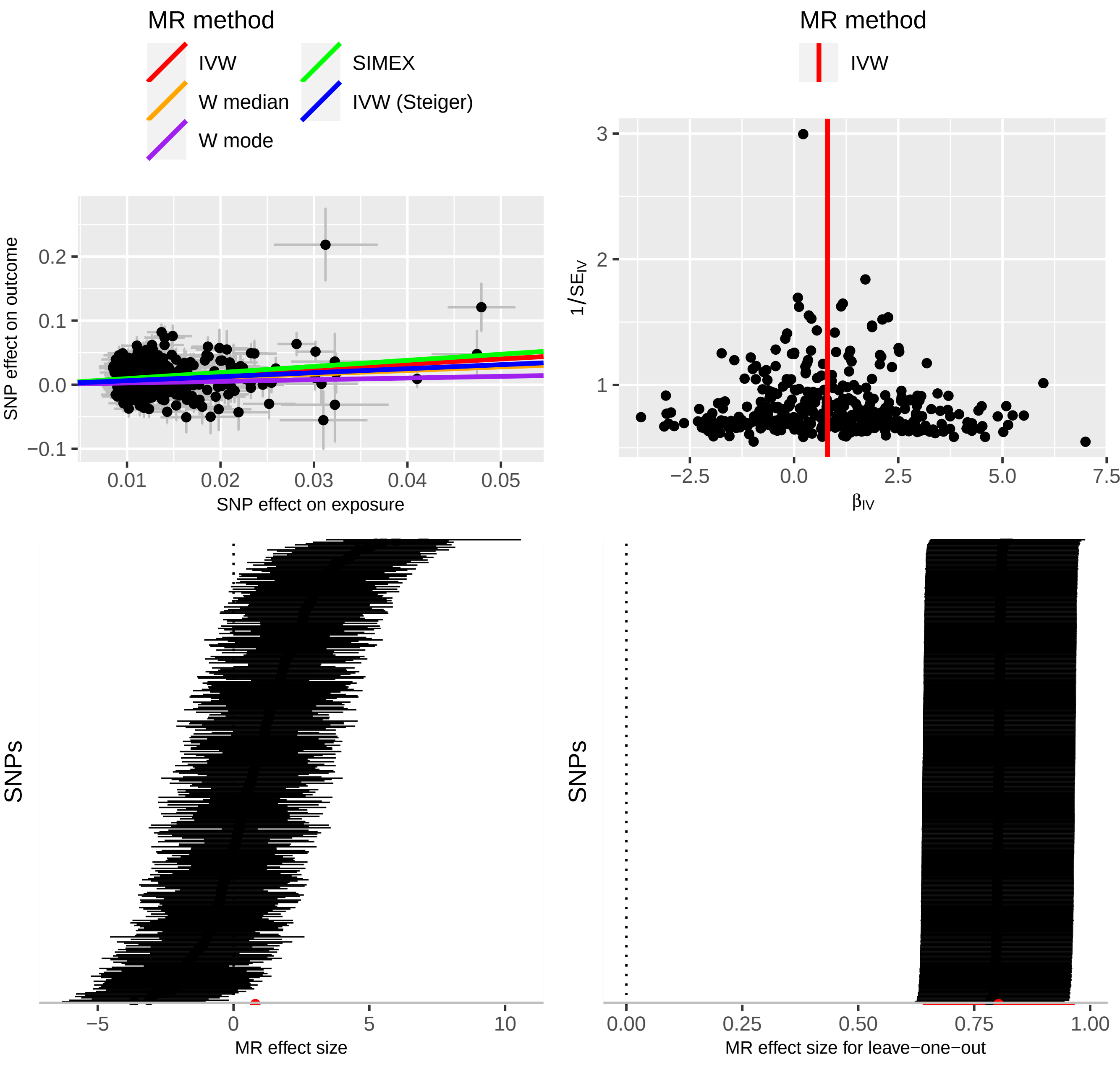

j) Body mass index (BMI) → ADHD

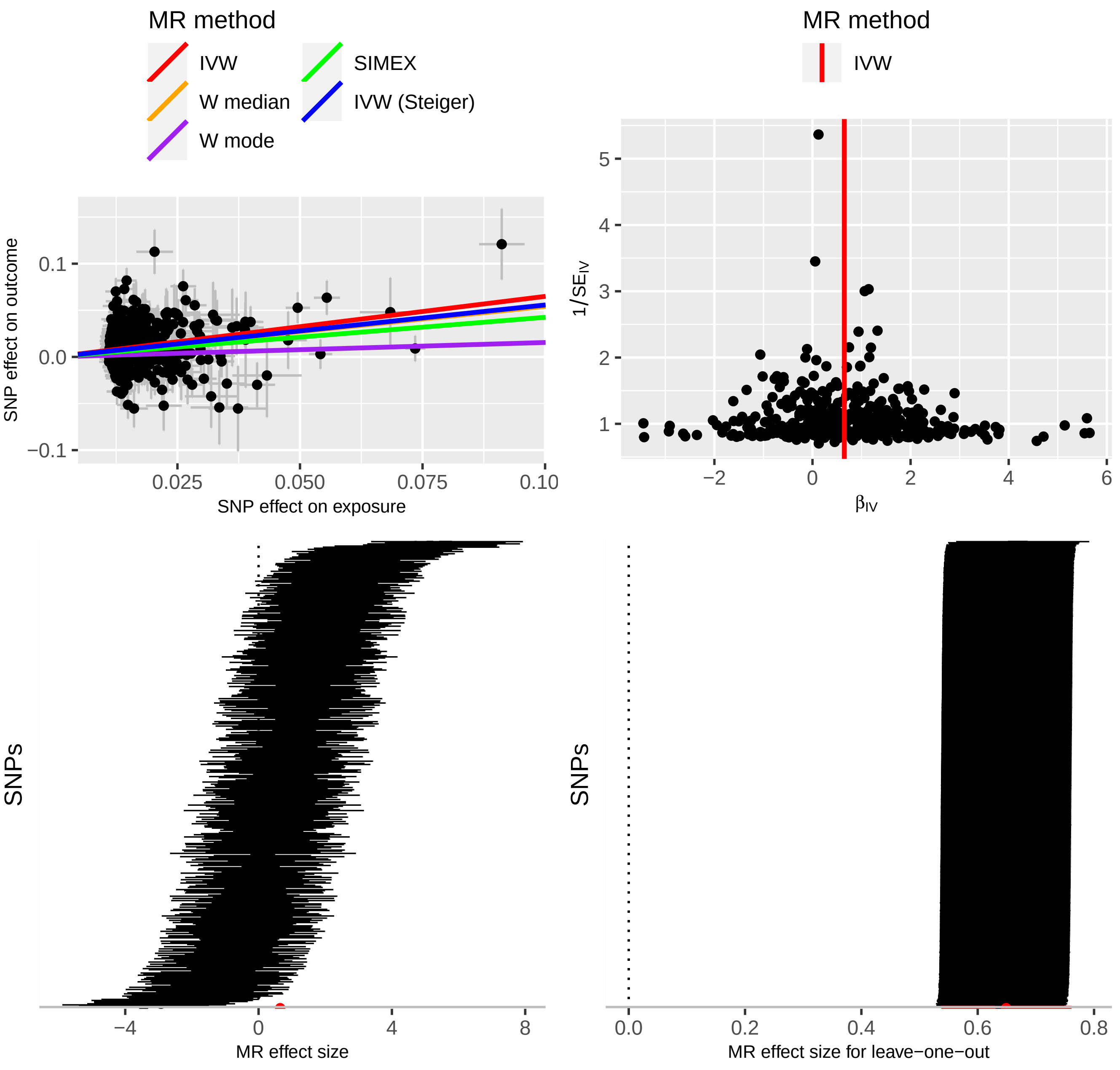

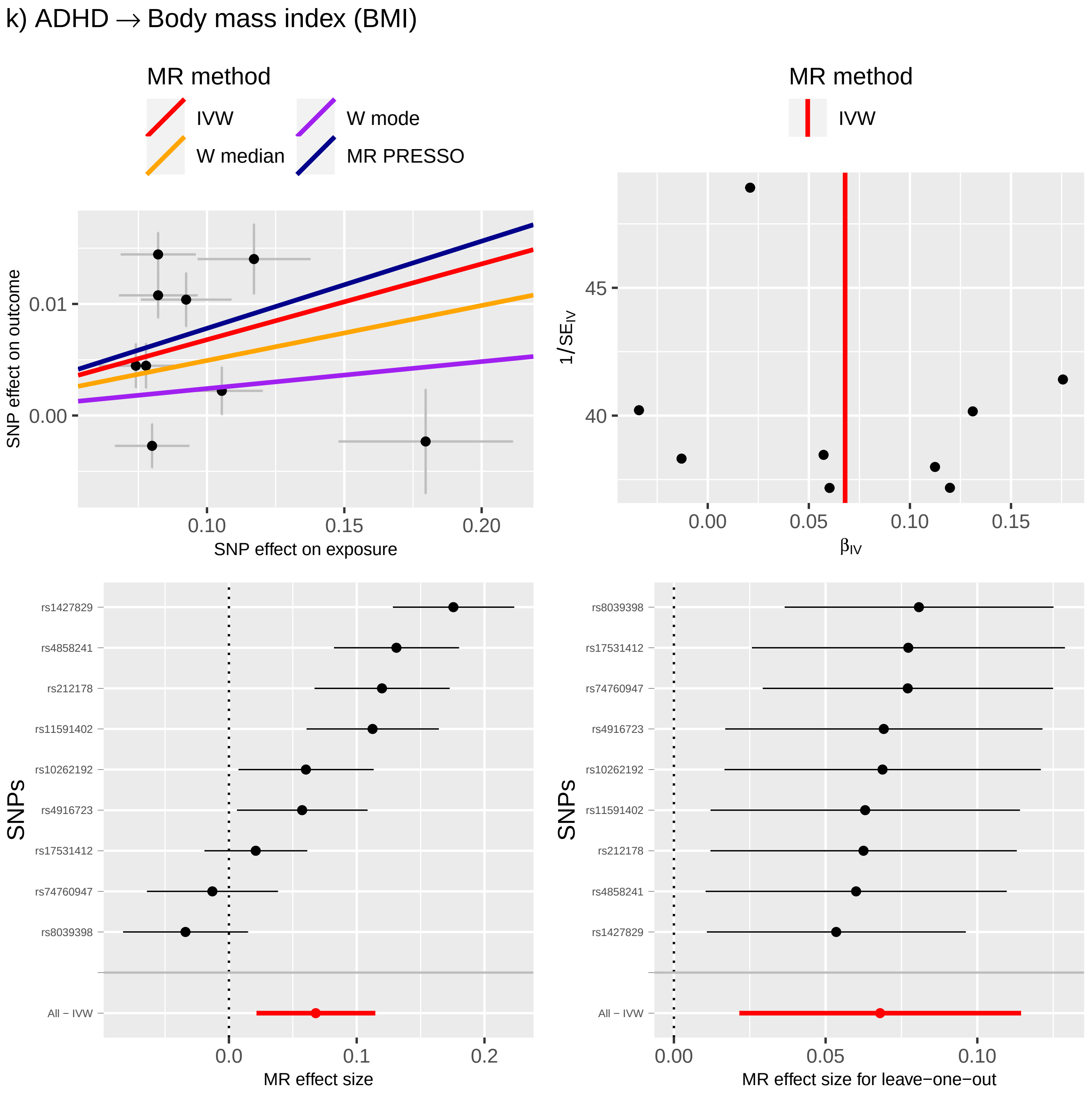

k) ADHD → Body mass index (BMI)
MR method
IVW
W median
W mode
MR PRESSO
SNP effect on outcome
SNP effect on exposure
0.01
0.00
0.10
0.15
0.20
MR method
IVW
1/SE_IV
β_IV
45
40
0.00
0.05
0.10
0.15
SNPs
rs1427829
rs4858241
rs212178
rs11591402
rs10262192
rs4916723
rs17531412
rs74760947
rs8039398
All − IVW
0.0
0.1
0.2
MR effect size
SNPs
rs8039398
rs17531412
rs74760947
rs4916723
rs10262192
rs11591402
rs212178
rs4858241
rs1427829
All − IVW
0.00
0.05
0.10
MR effect size for leave−one−out

l) Impedance of whole body → ADHD

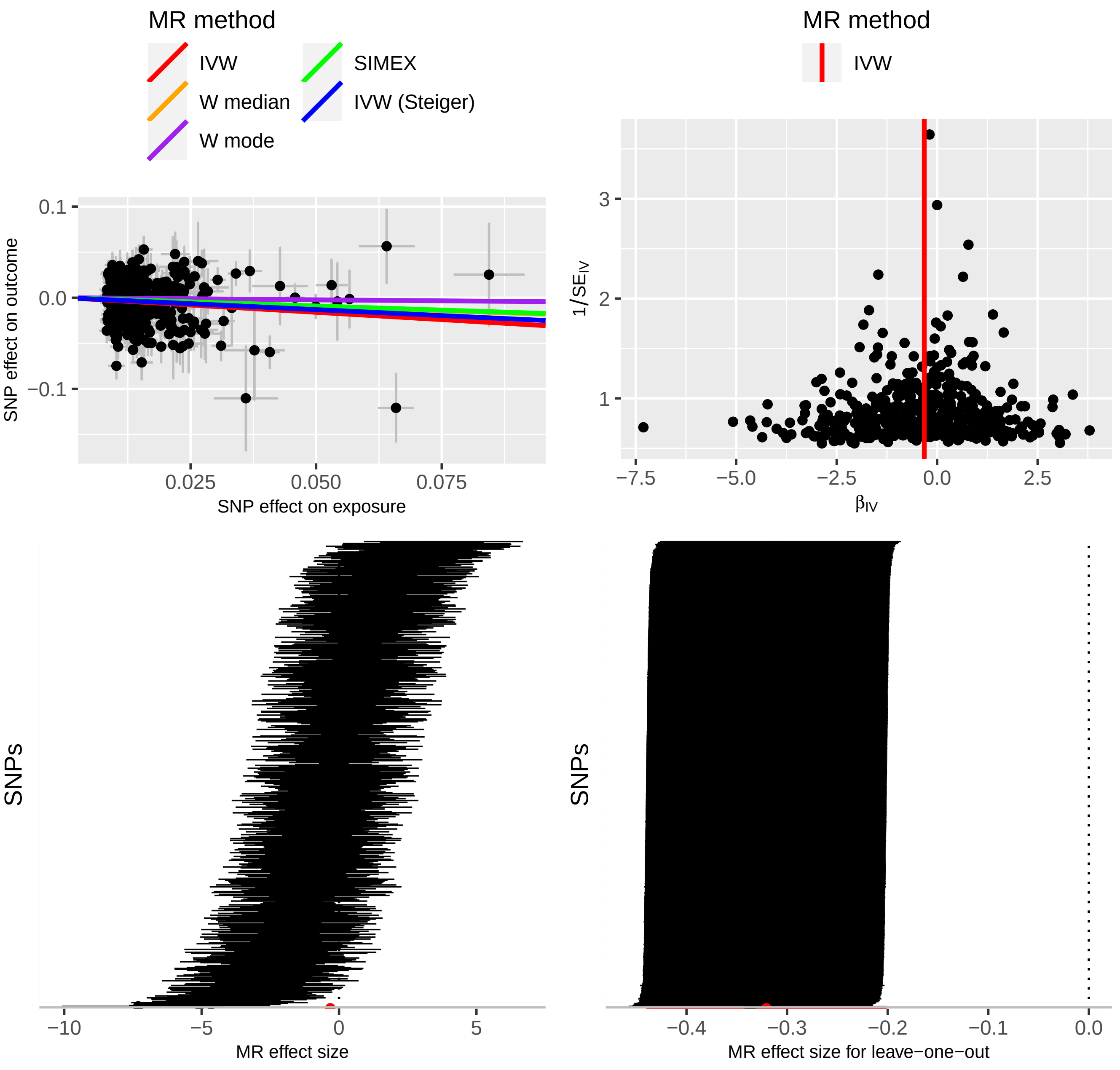

m) Whole body fat−free mass → ADHD

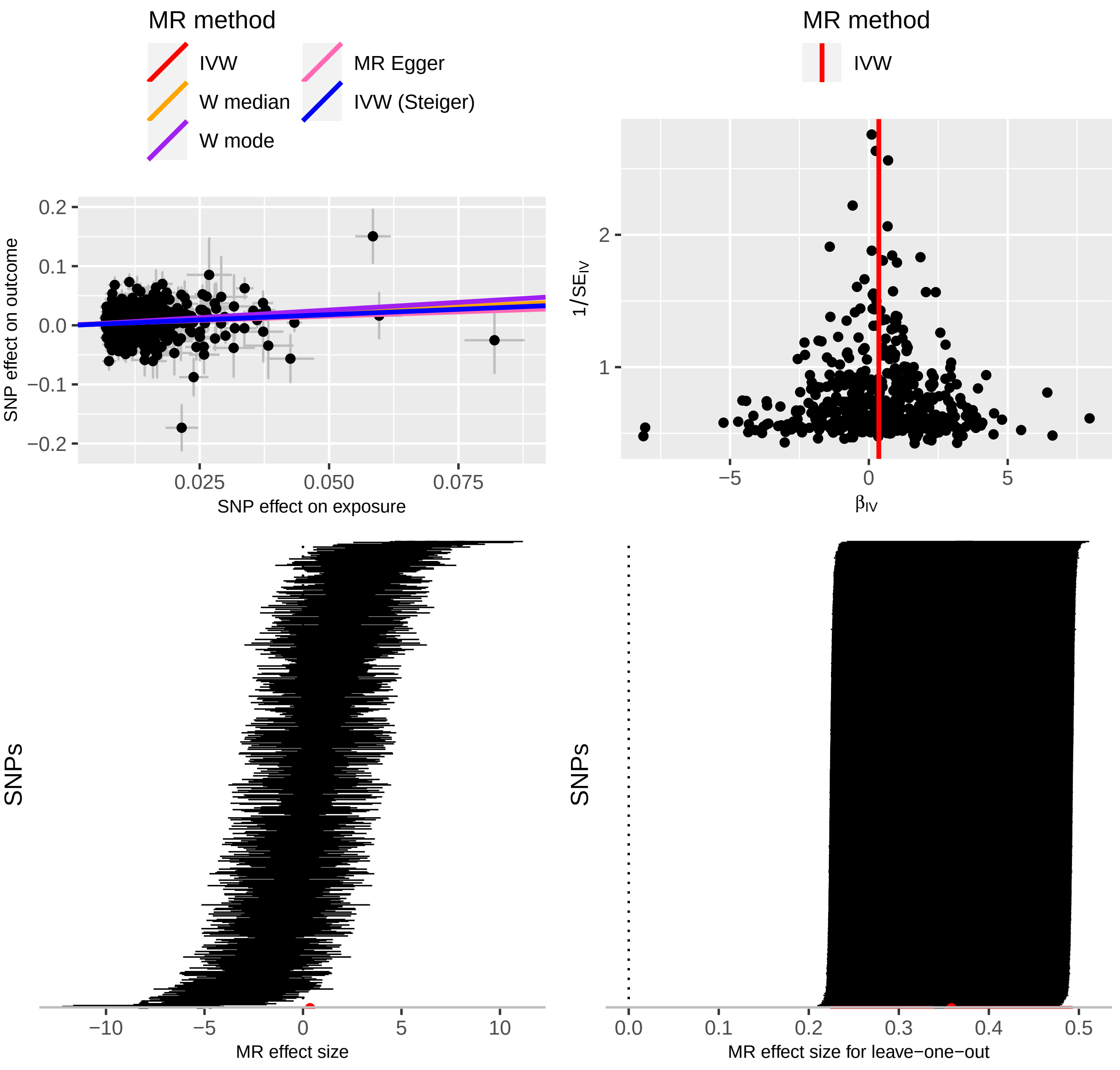

n) Whole body fat mass → ADHD

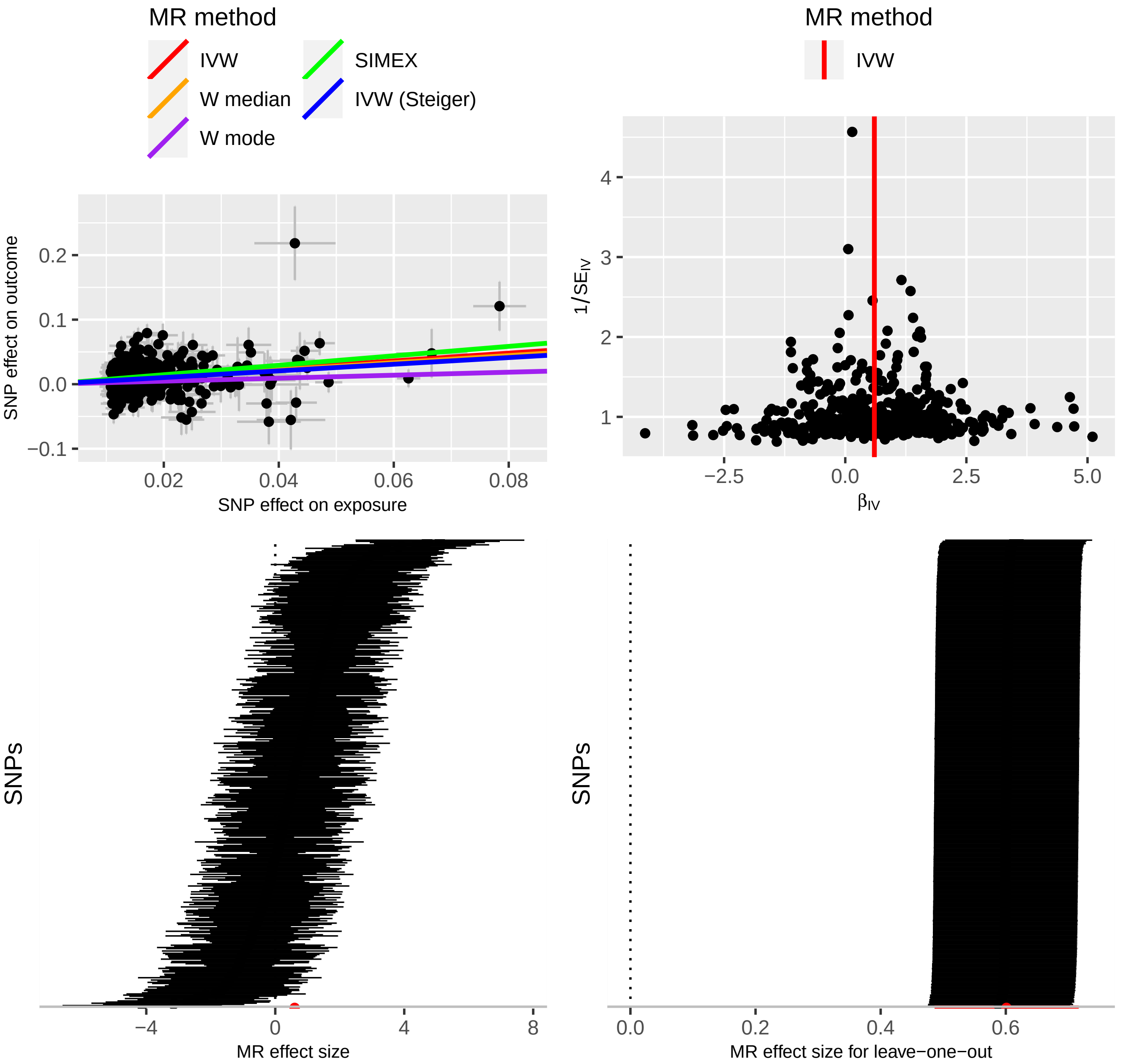

o) Whole body water mass → ADHD

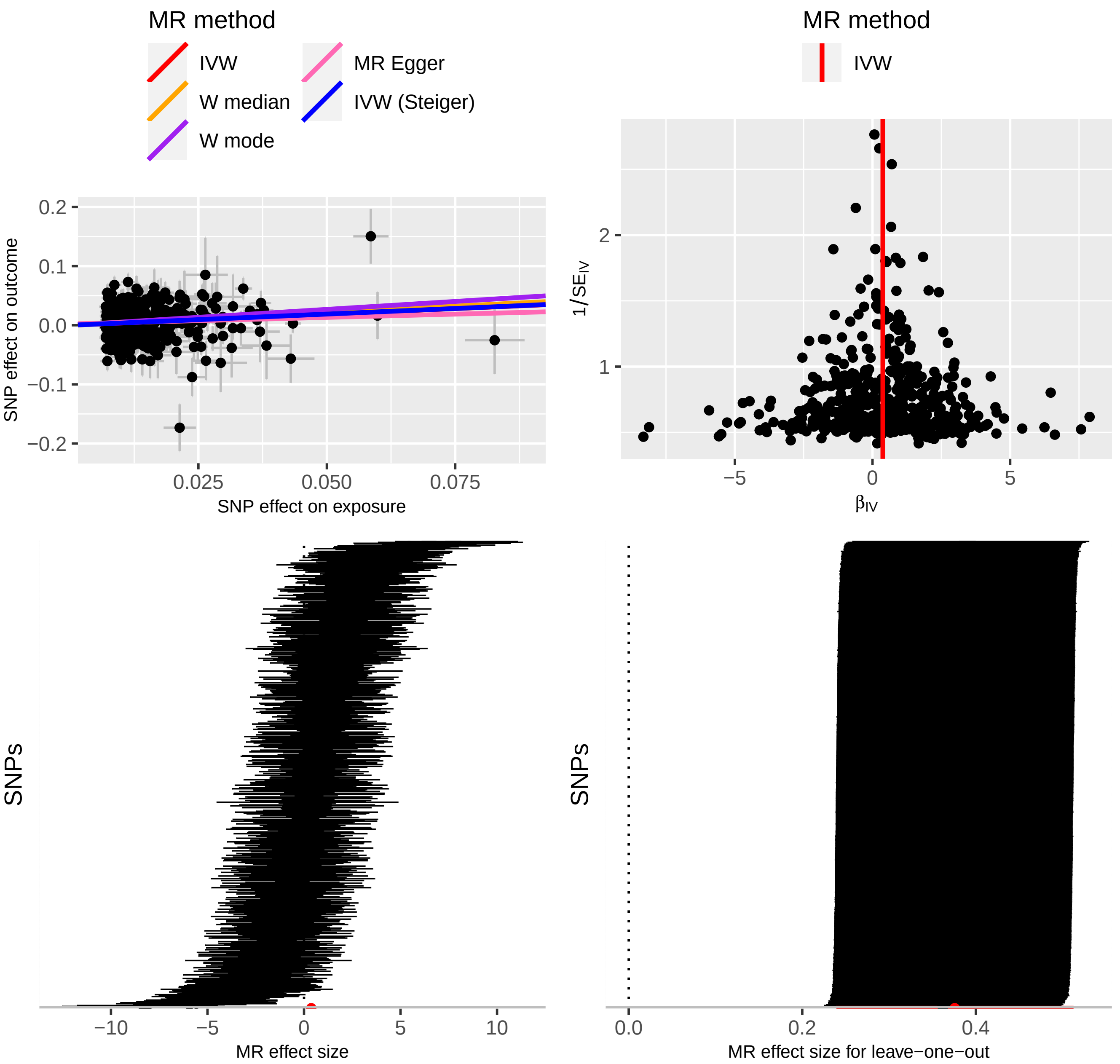

p) Hip circumference → ADHD

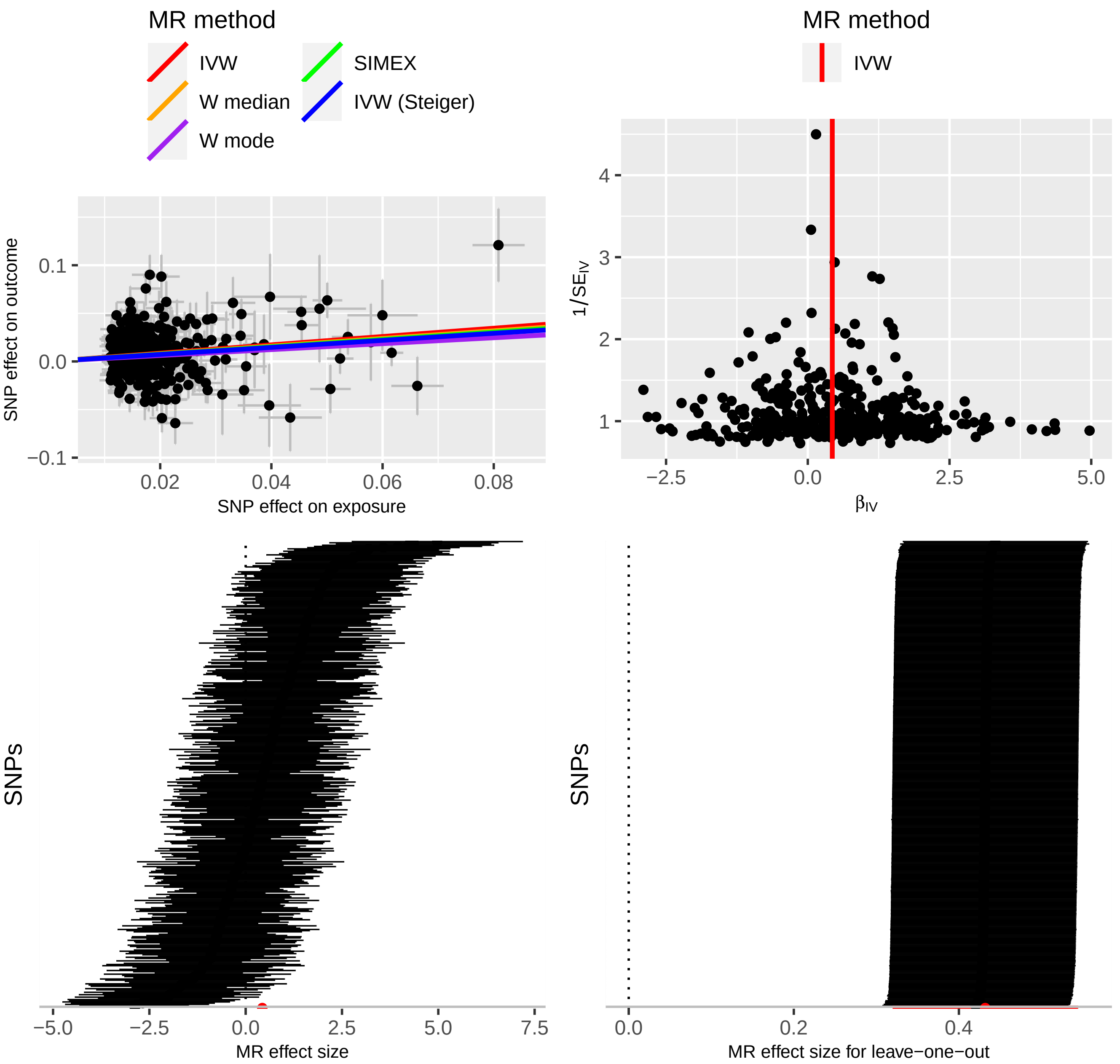

q) Impedance of leg (left) → ADHD

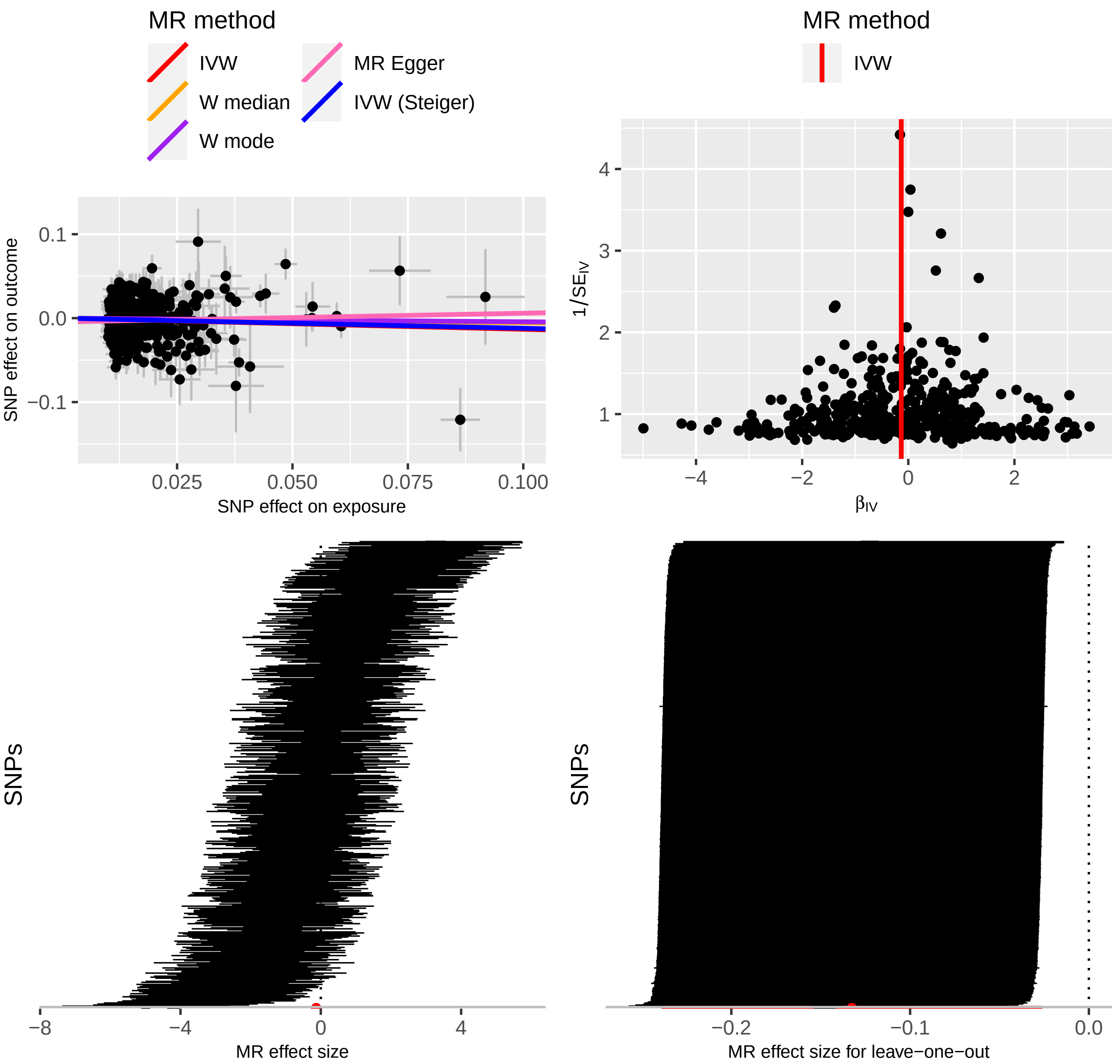

r) Leg fat−free mass (left) → ADHD

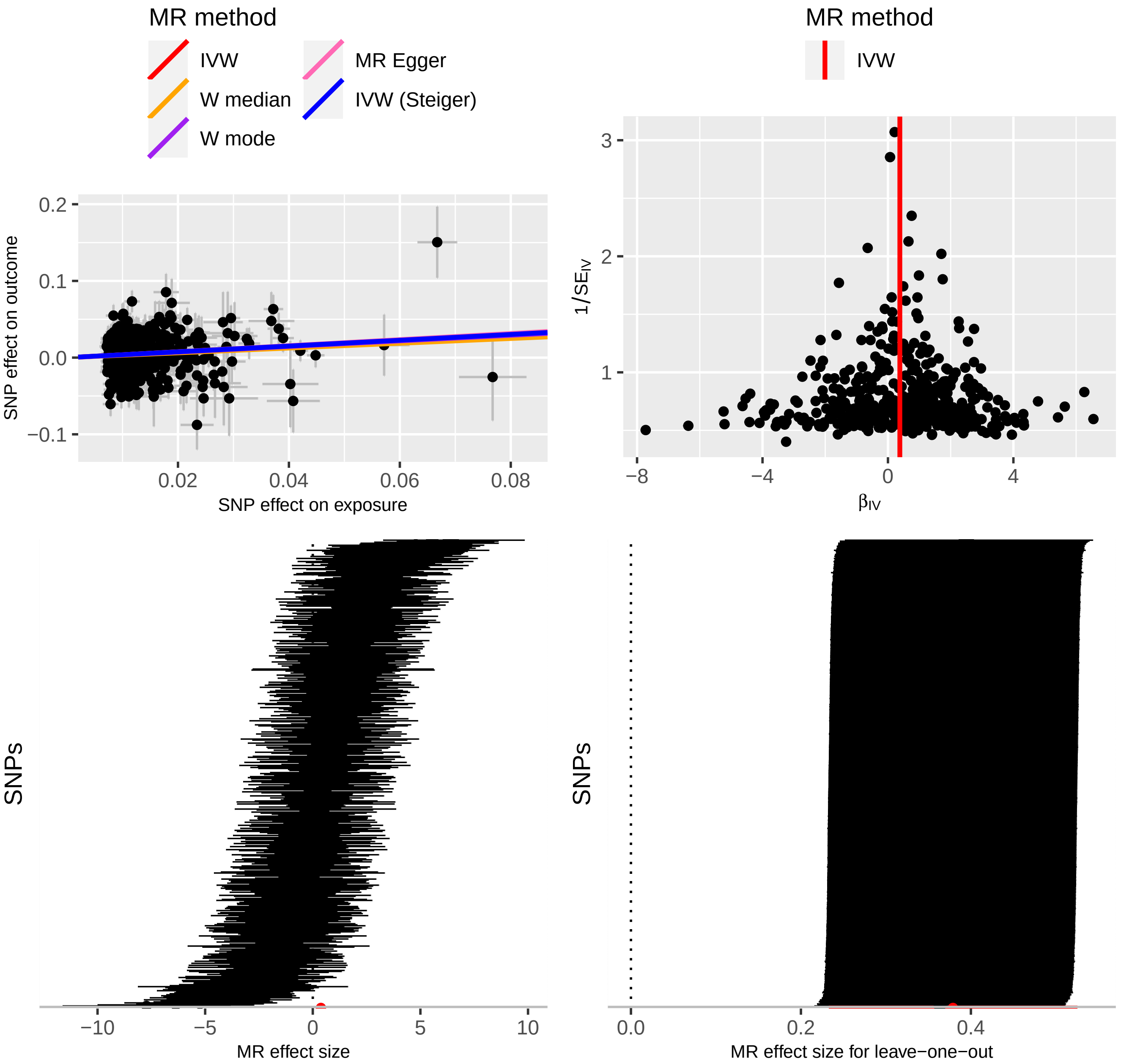

s) ADHD → Leg fat mass (left)

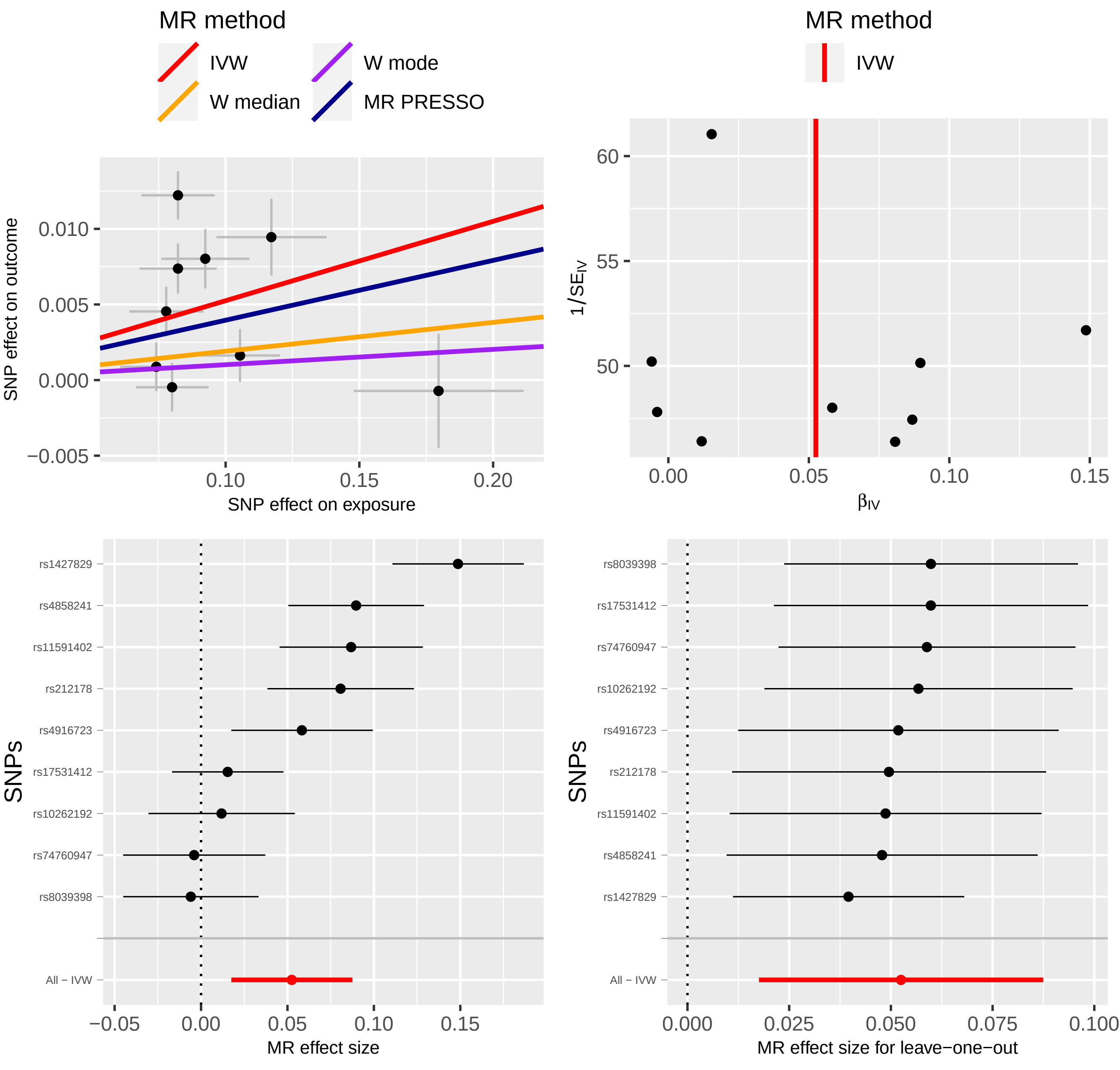

t) Leg fat mass (left) → ADHD

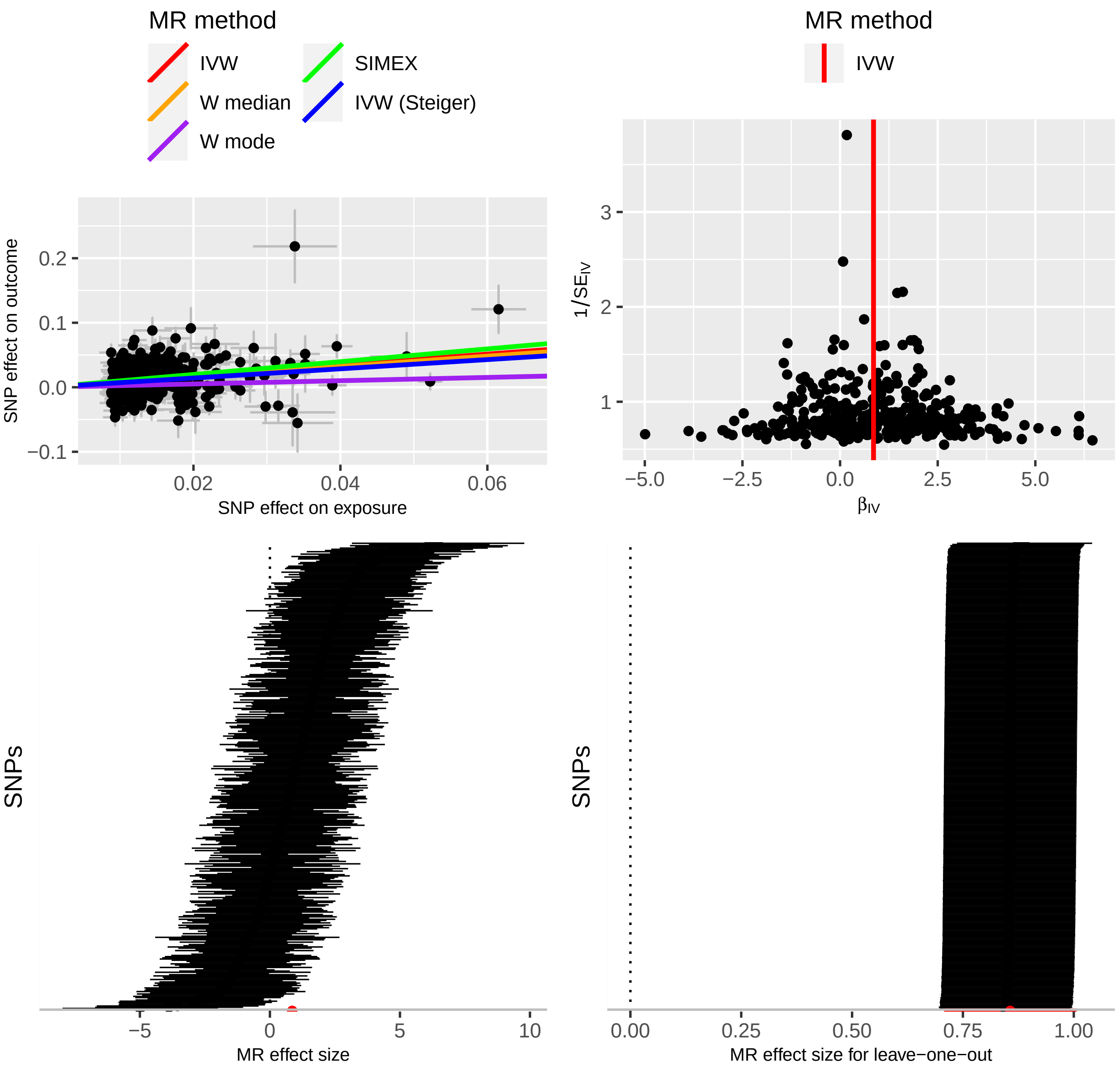

u) Leg fat percentage (left) → ADHD

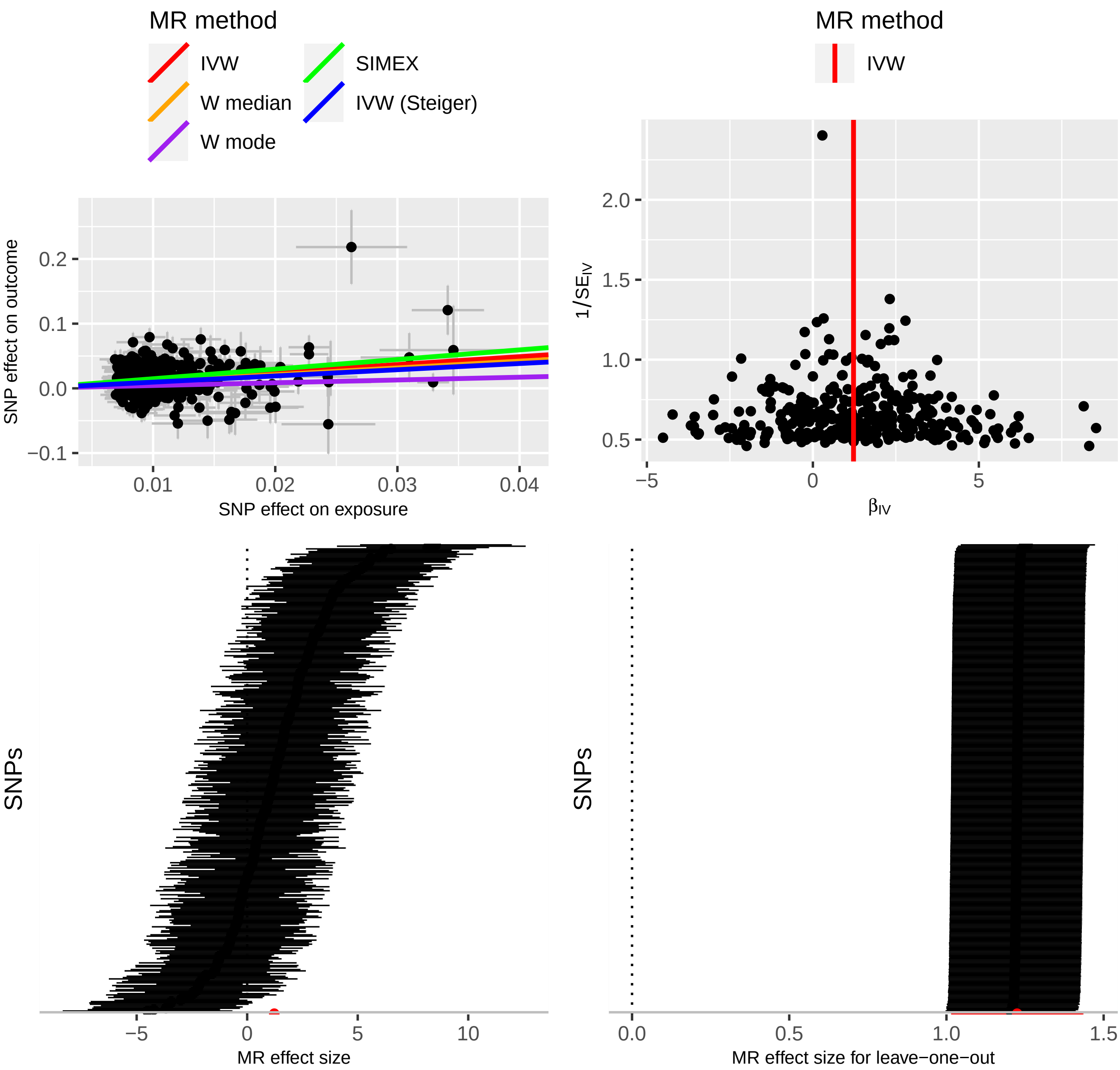

v) ADHD → Leg fat percentage (left)

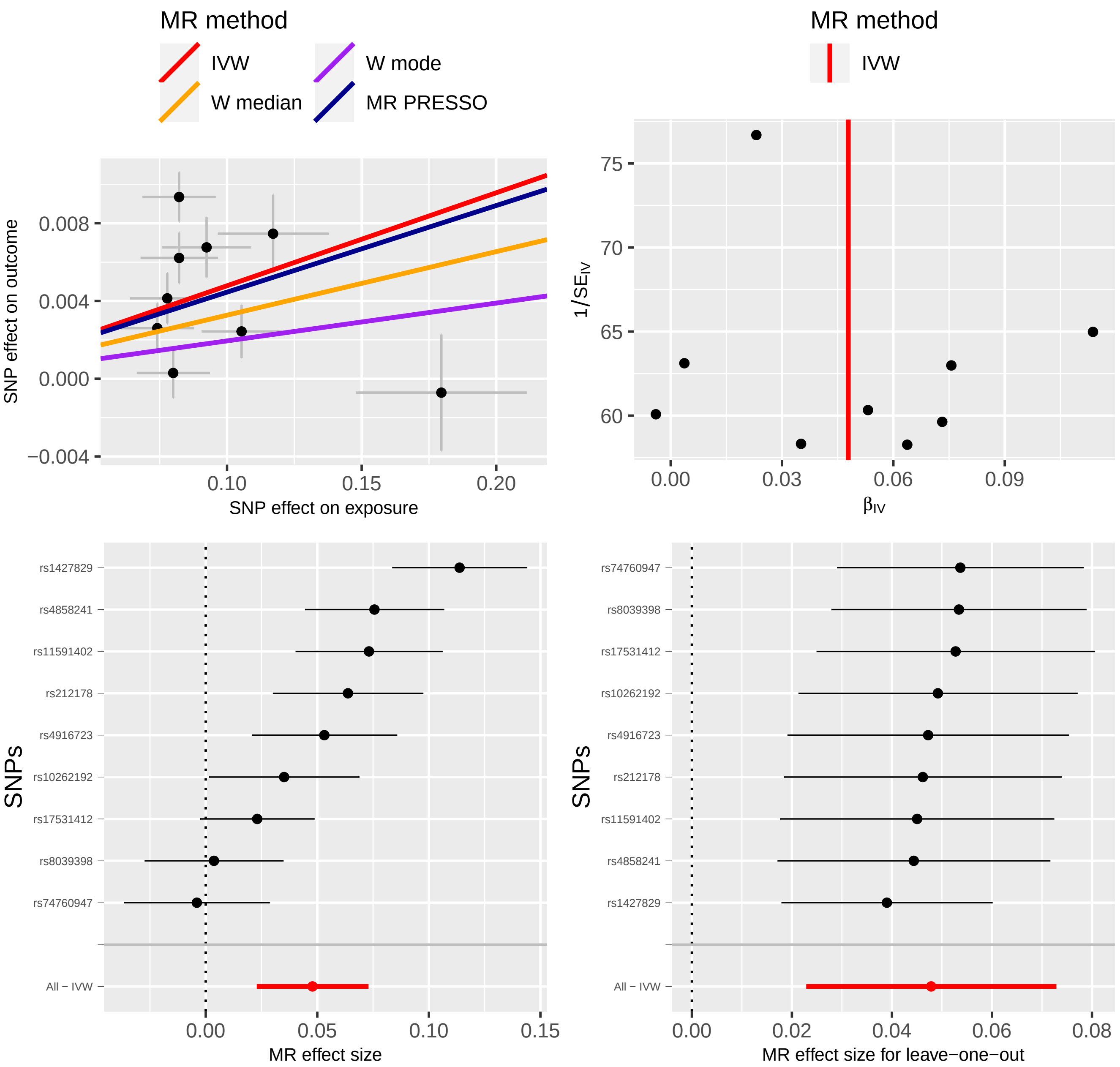

w) Leg predicted mass (left) → ADHD

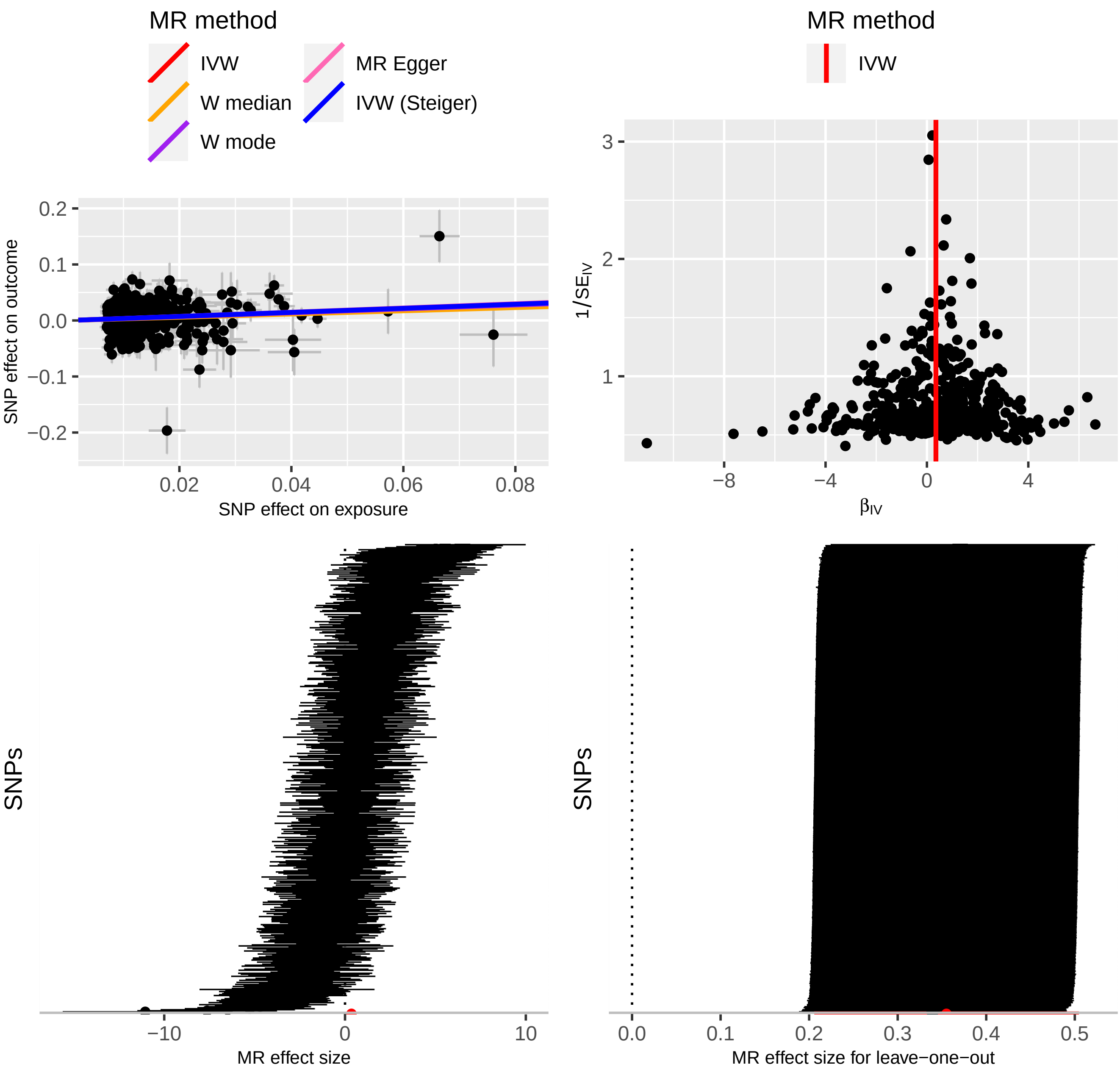

x) Trunk fat−free mass → ADHD

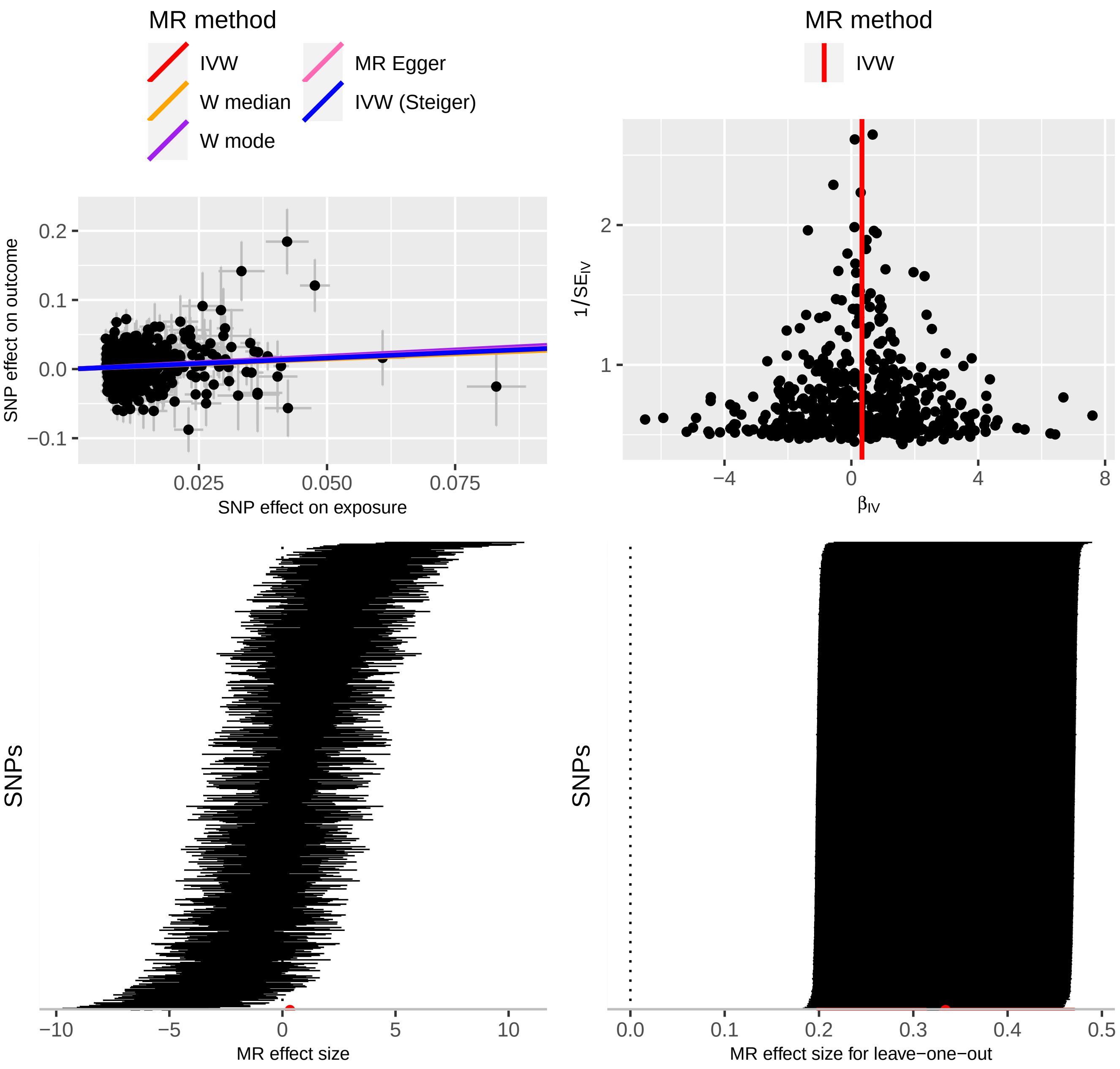

y) Trunk fat mass → ADHD

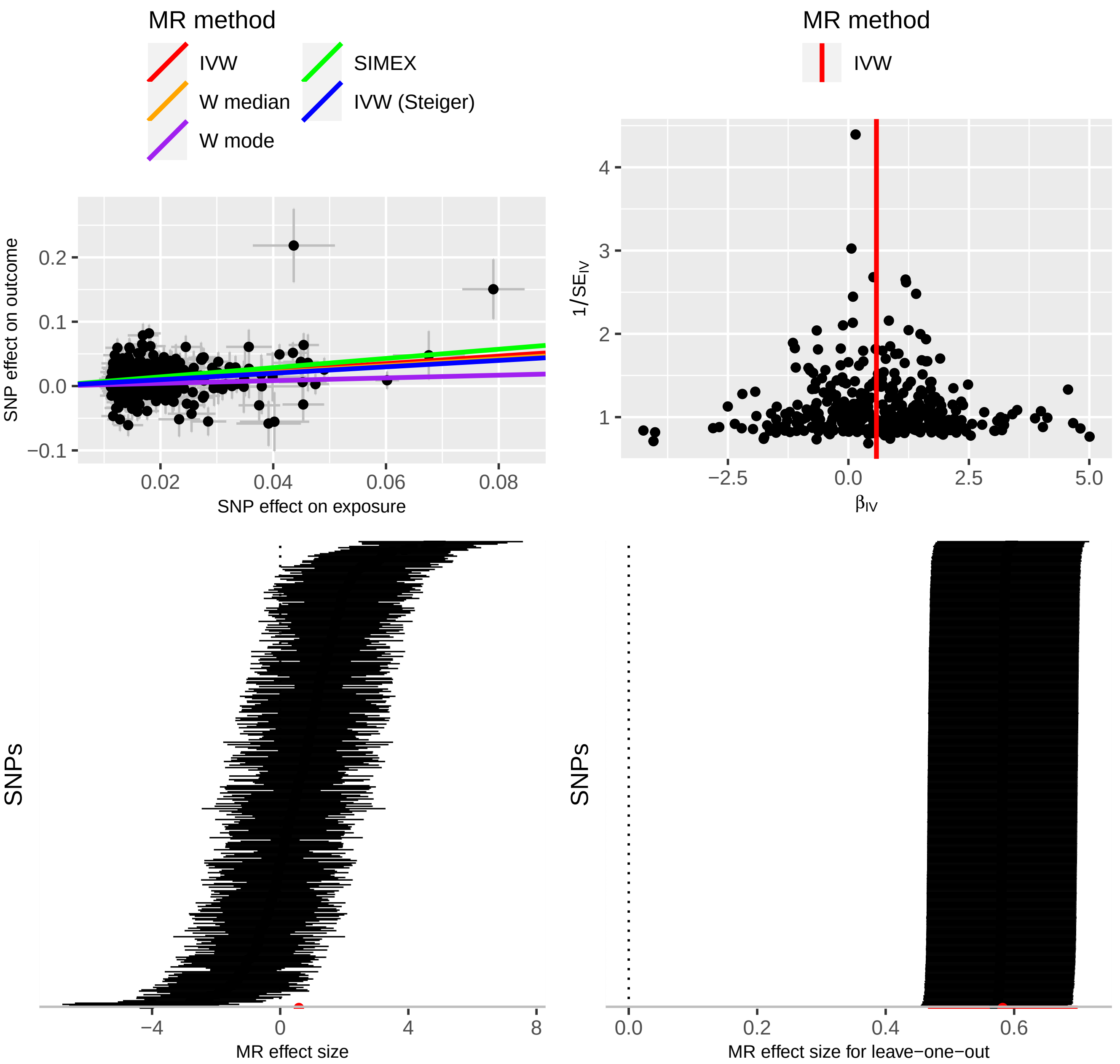

z) Trunk fat percentage → ADHD

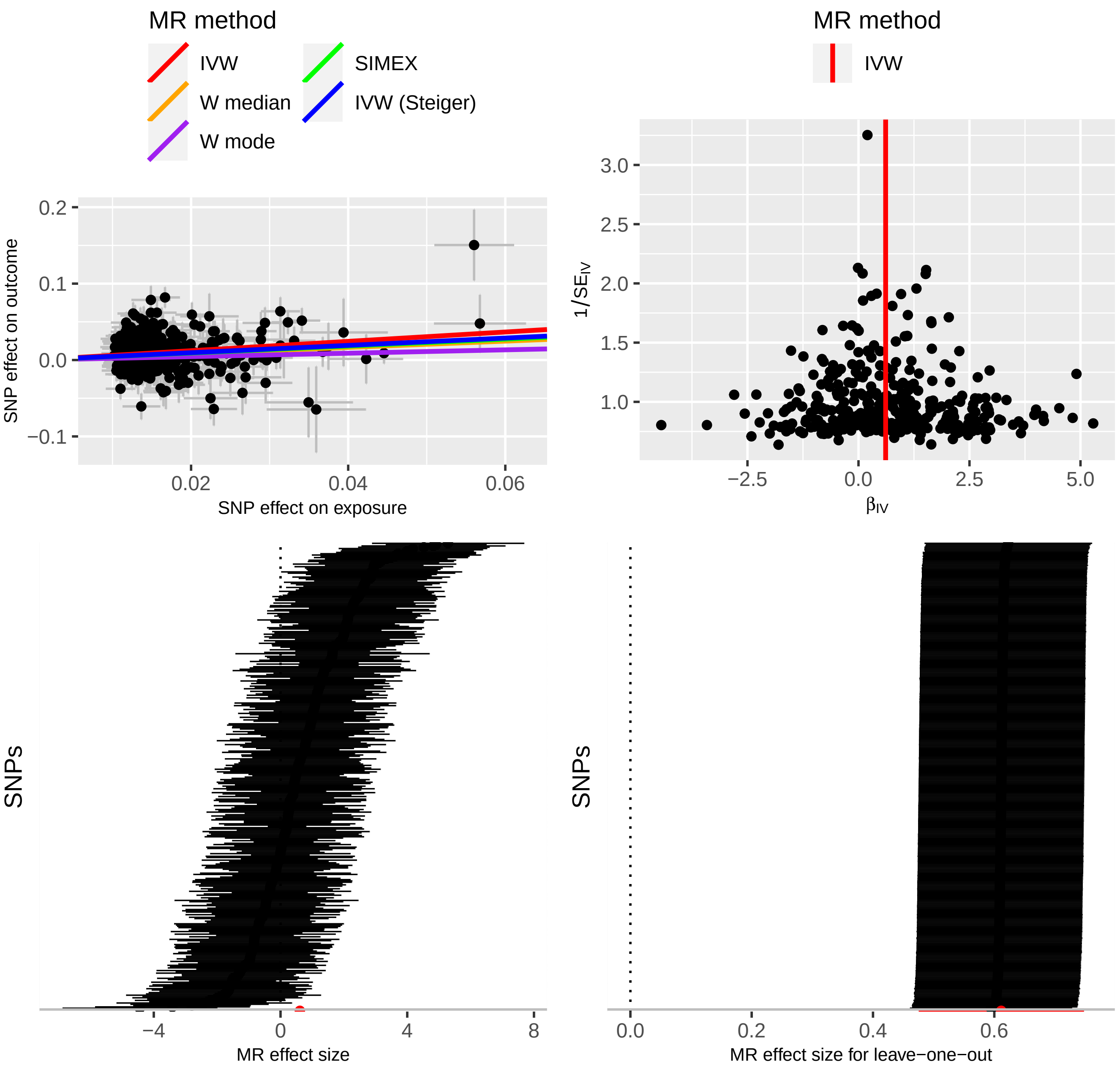

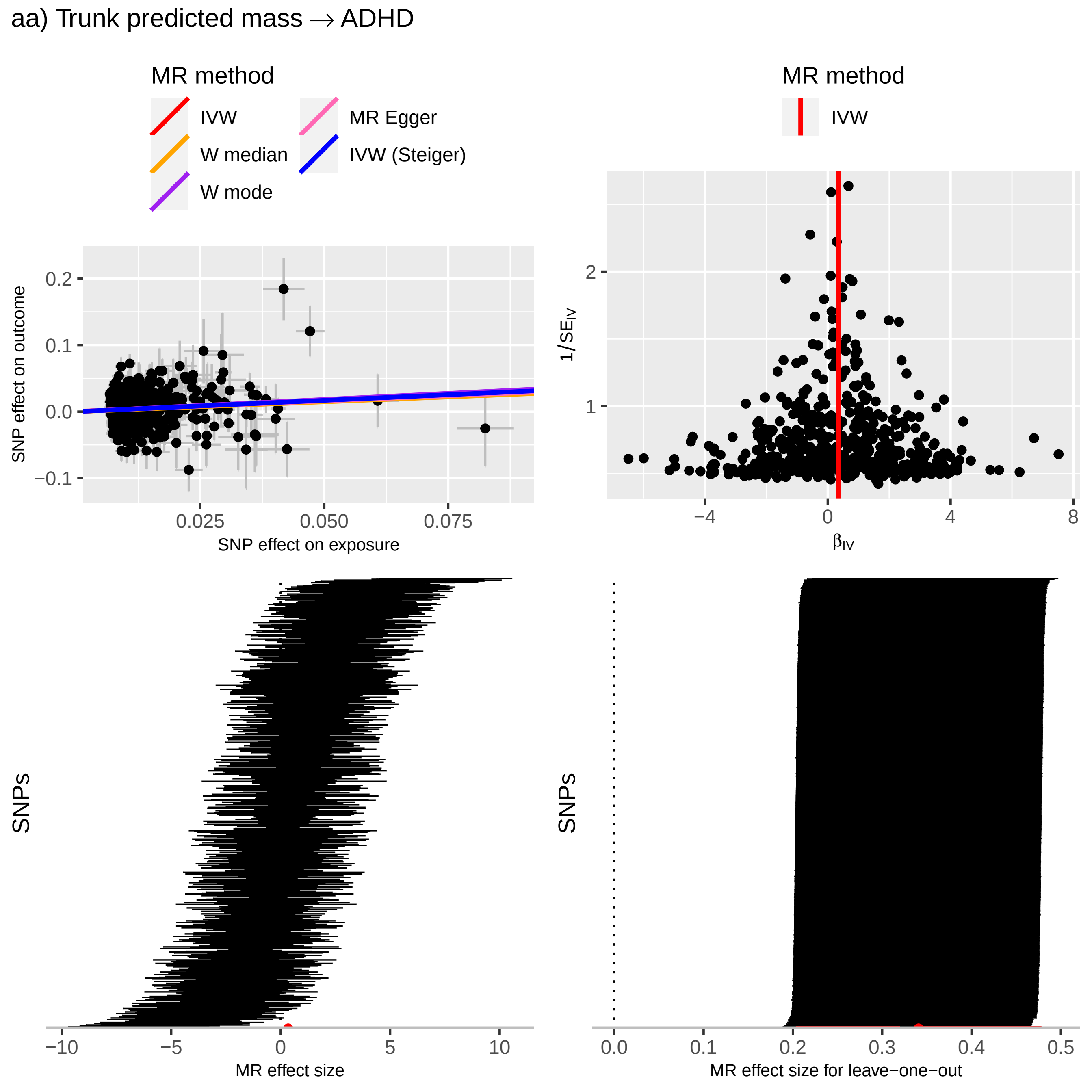

aa) Trunk predicted mass → ADHD
MR method
IVW
W median
W mode
MR Egger
IVW (Steiger)
SNP effect on outcome
SNP effect on exposure
0.2
0.1
0.0
−0.1
0.025
0.050
0.075
MR method
IVW
$1/SE_{IV}$
β_{IV}
2
1
−4
0
4
8
SNPs
MR effect size
−10
−5
0
5
10
SNPs
MR effect size for leave−one−out
0.0
0.1
0.2
0.3
0.4
0.5

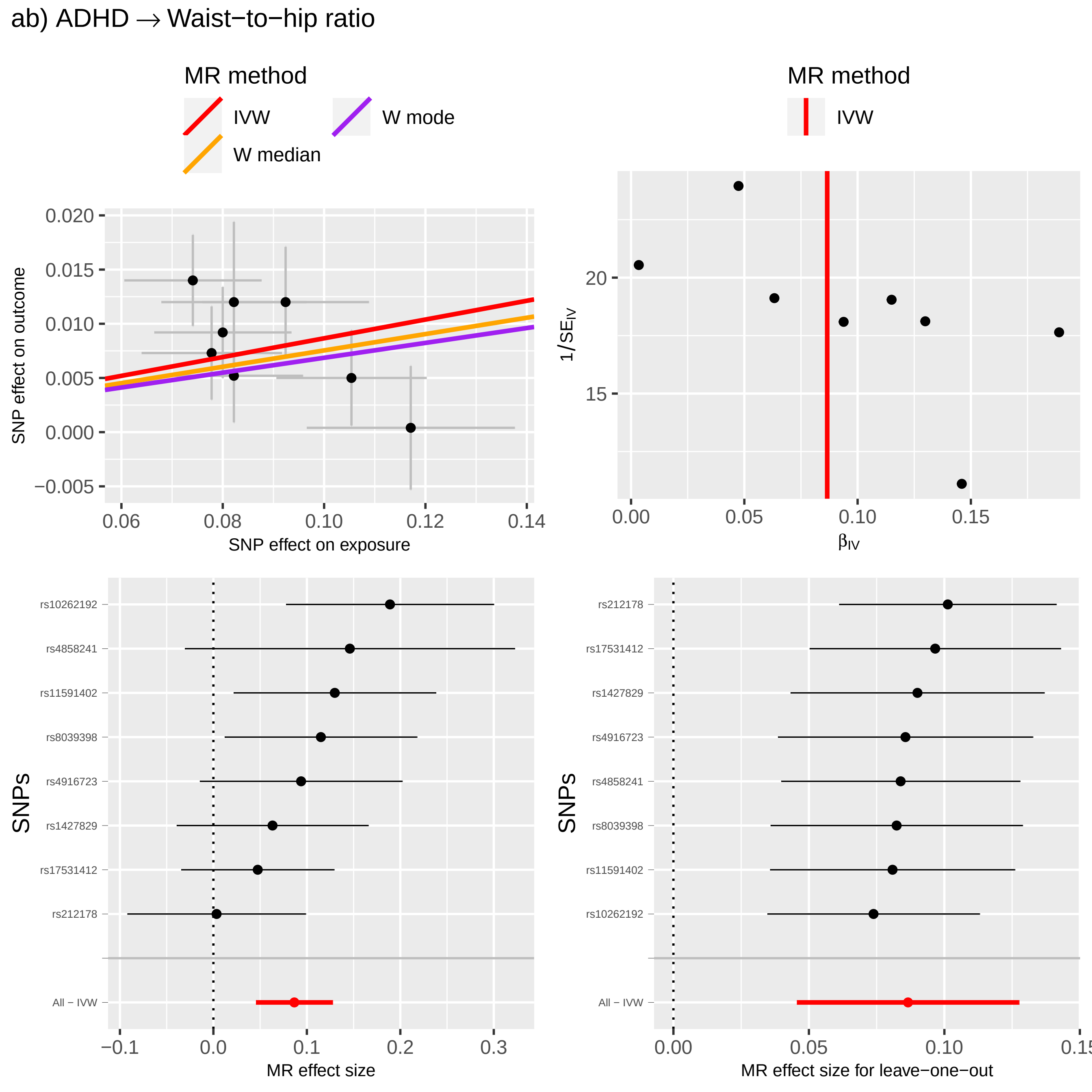

ab) ADHD → Waist−to−hip ratio
MR method
IVW
W mode
W median
0.020
0.015
0.010
0.005
0.000
−0.005
SNP effect on outcome
0.06
0.08
0.10
0.12
0.14
SNP effect on exposure
MR method
IVW
20
15
1/SE_IV
0.00
0.05
0.10
0.15
β_IV
rs10262192
rs4858241
rs11591402
rs8039398
rs4916723
rs1427829
rs17531412
rs212178
All − IVW
SNPs
−0.1
0.0
0.1
0.2
0.3
MR effect size
rs212178
rs17531412
rs1427829
rs4916723
rs4858241
rs8039398
rs11591402
rs10262192
All − IVW
SNPs
0.00
0.05
0.10
MR effect size for leave−one−out

ac) ADHD → Waist circumference

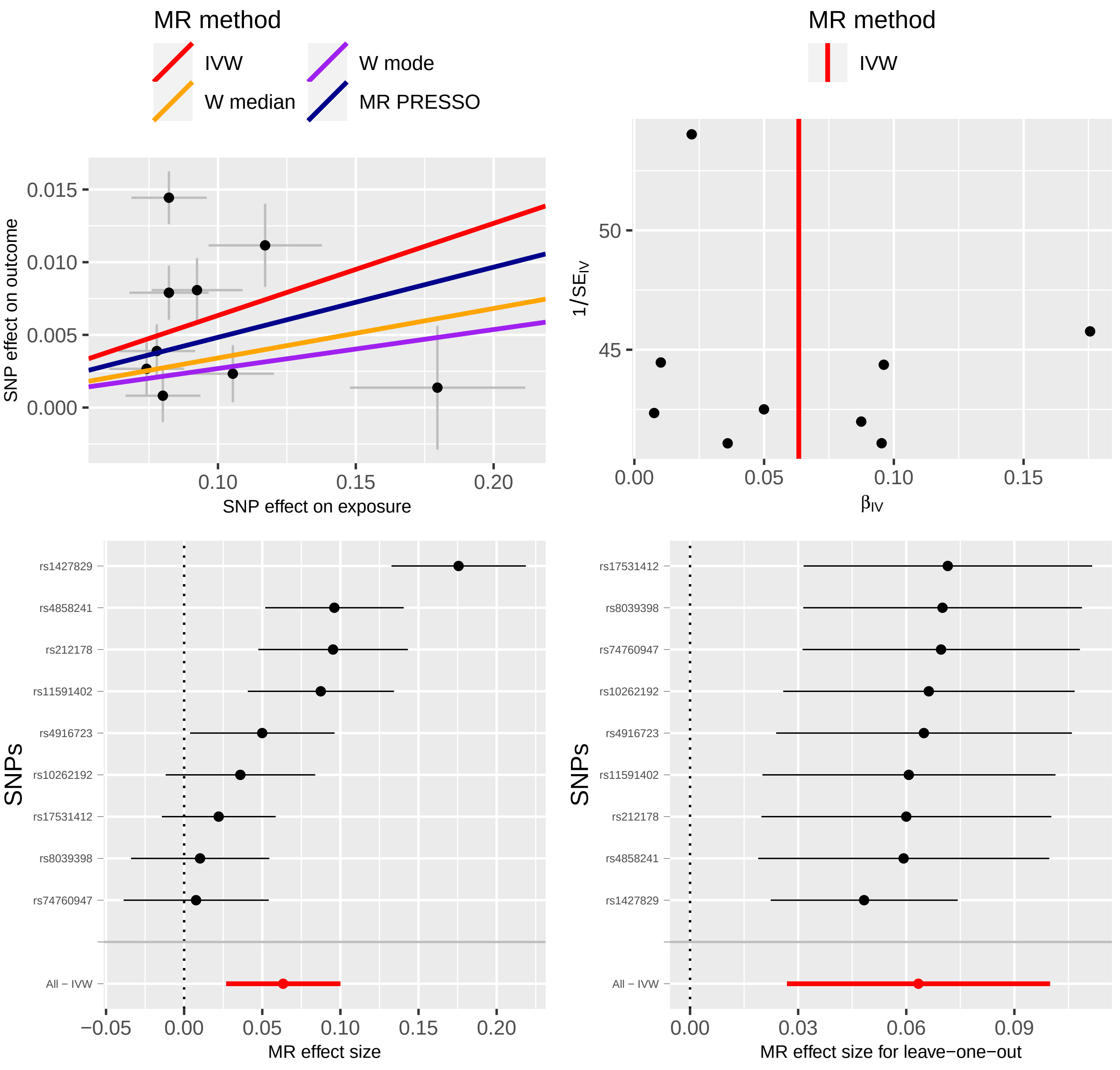

ad) Waist circumference → ADHD

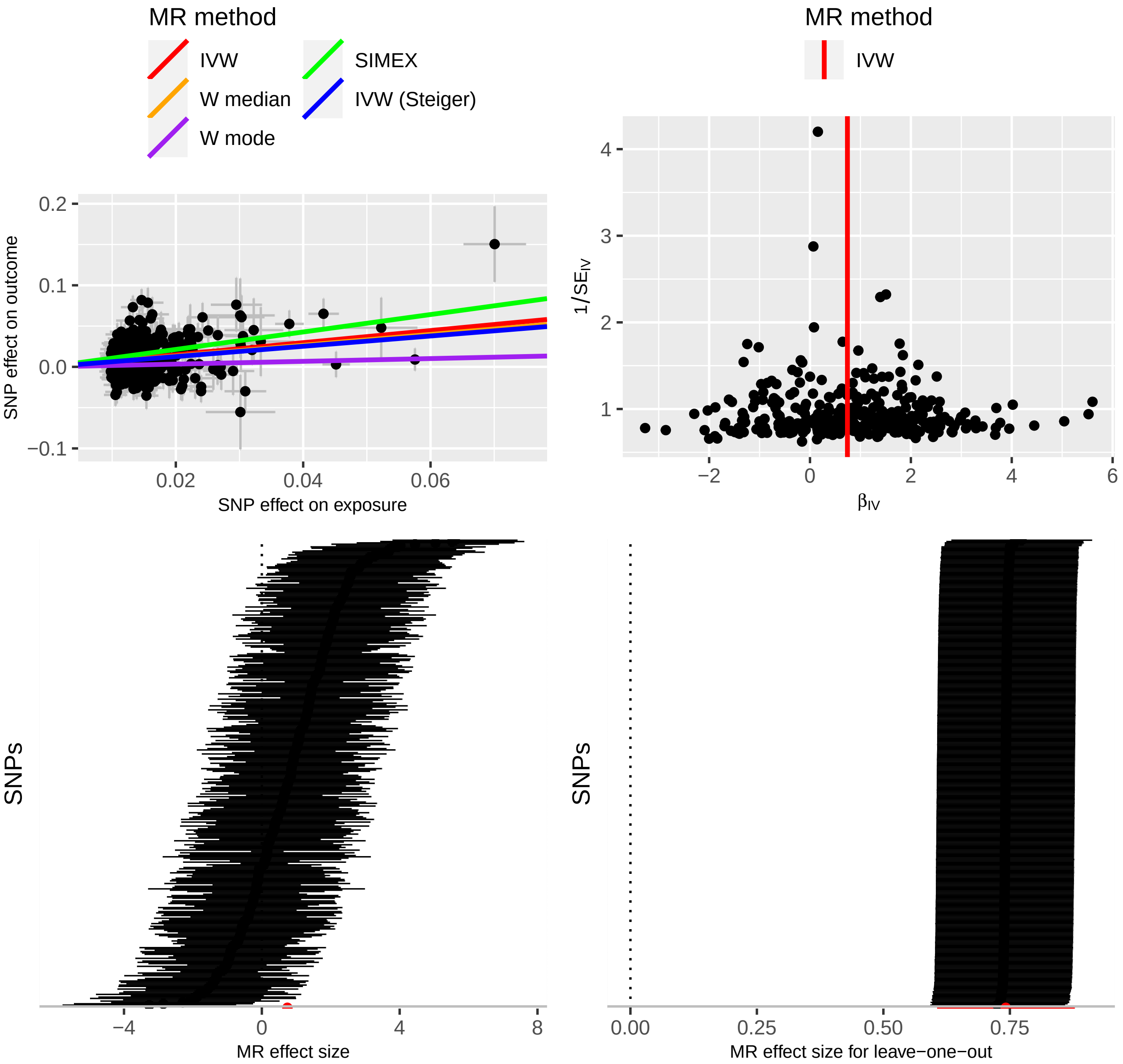

ae) Overweight → ADHD

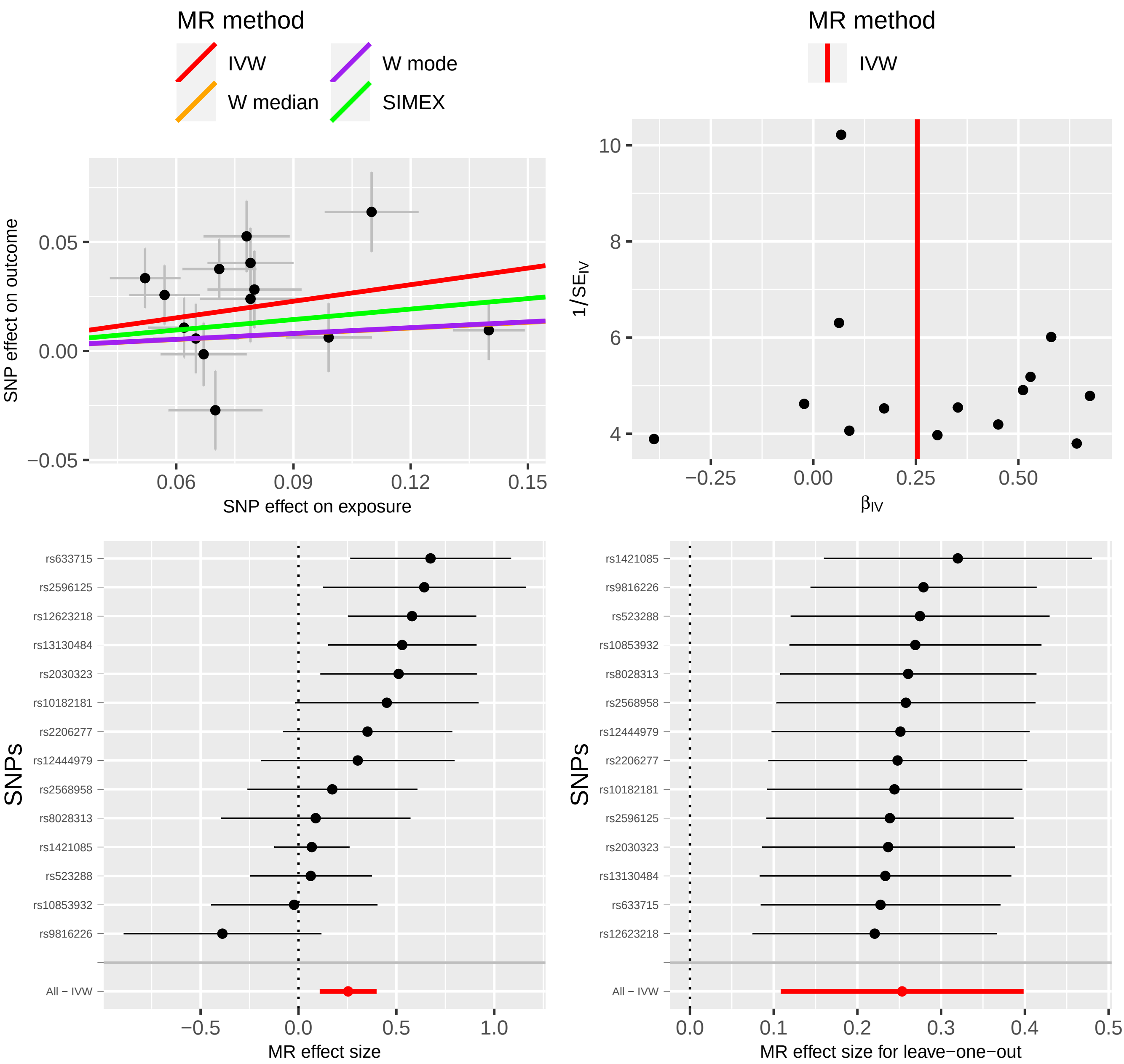

af) Weight → ADHD

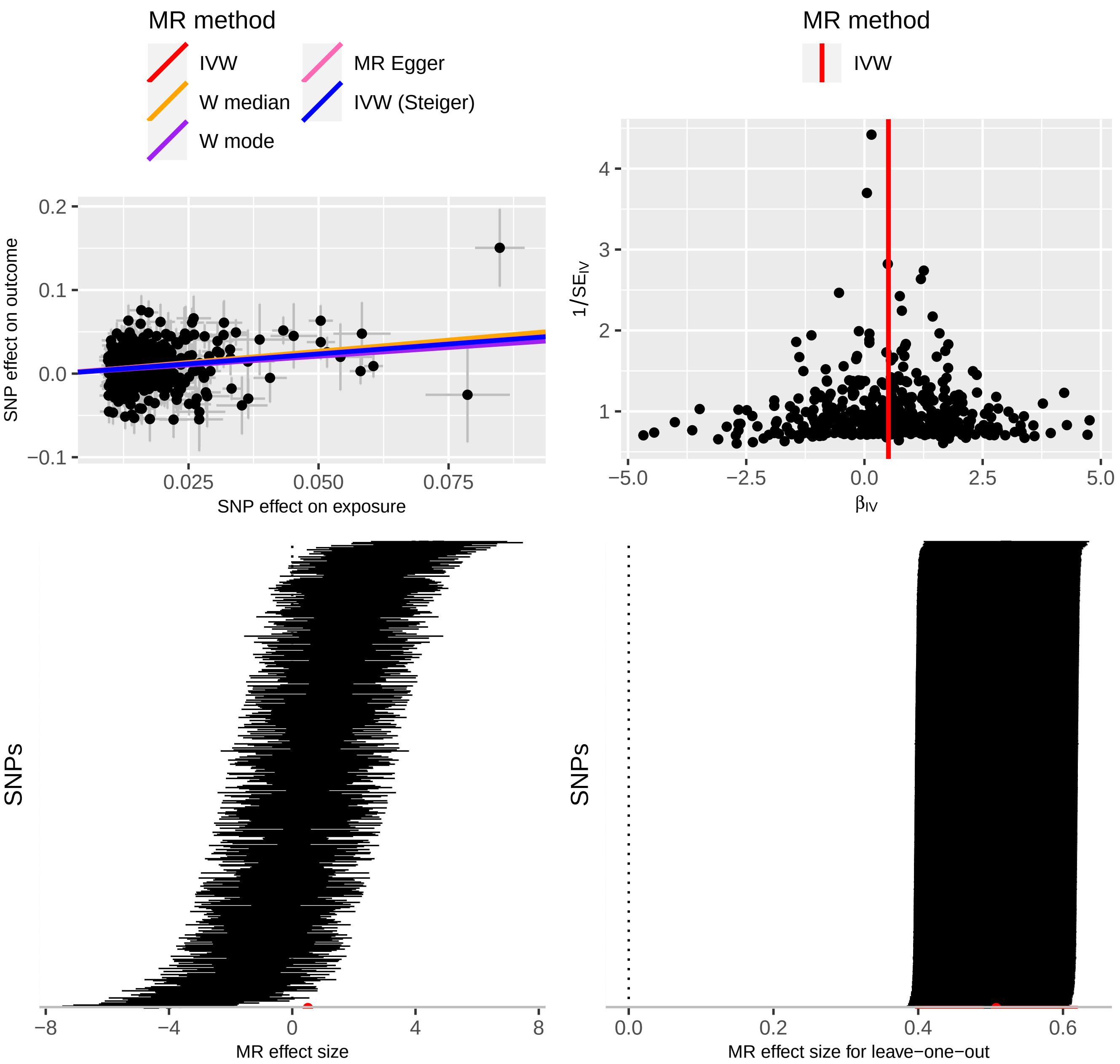

ag) Cognitive performance → ADHD

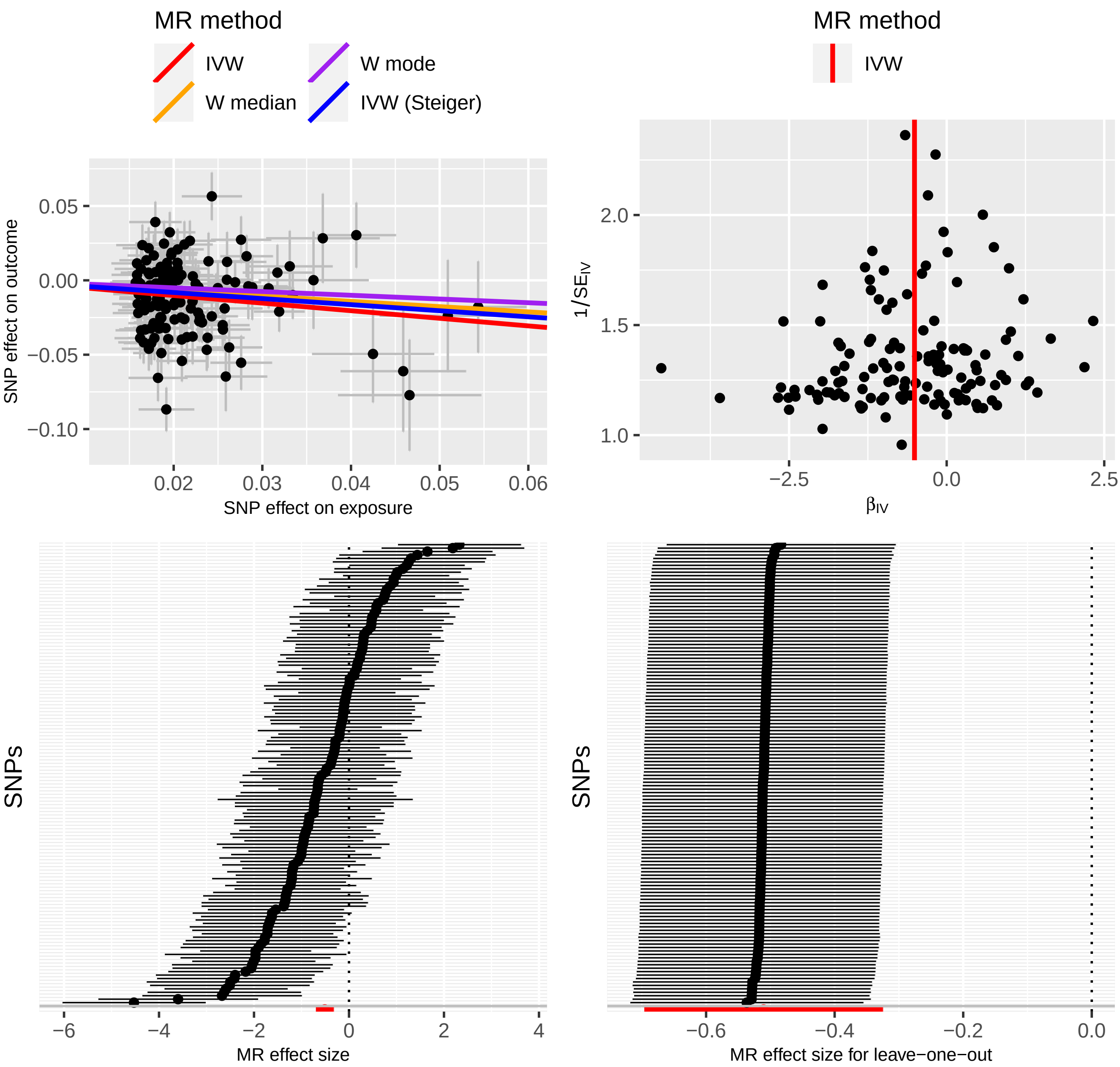

ah) ADHD → Cognitive performance

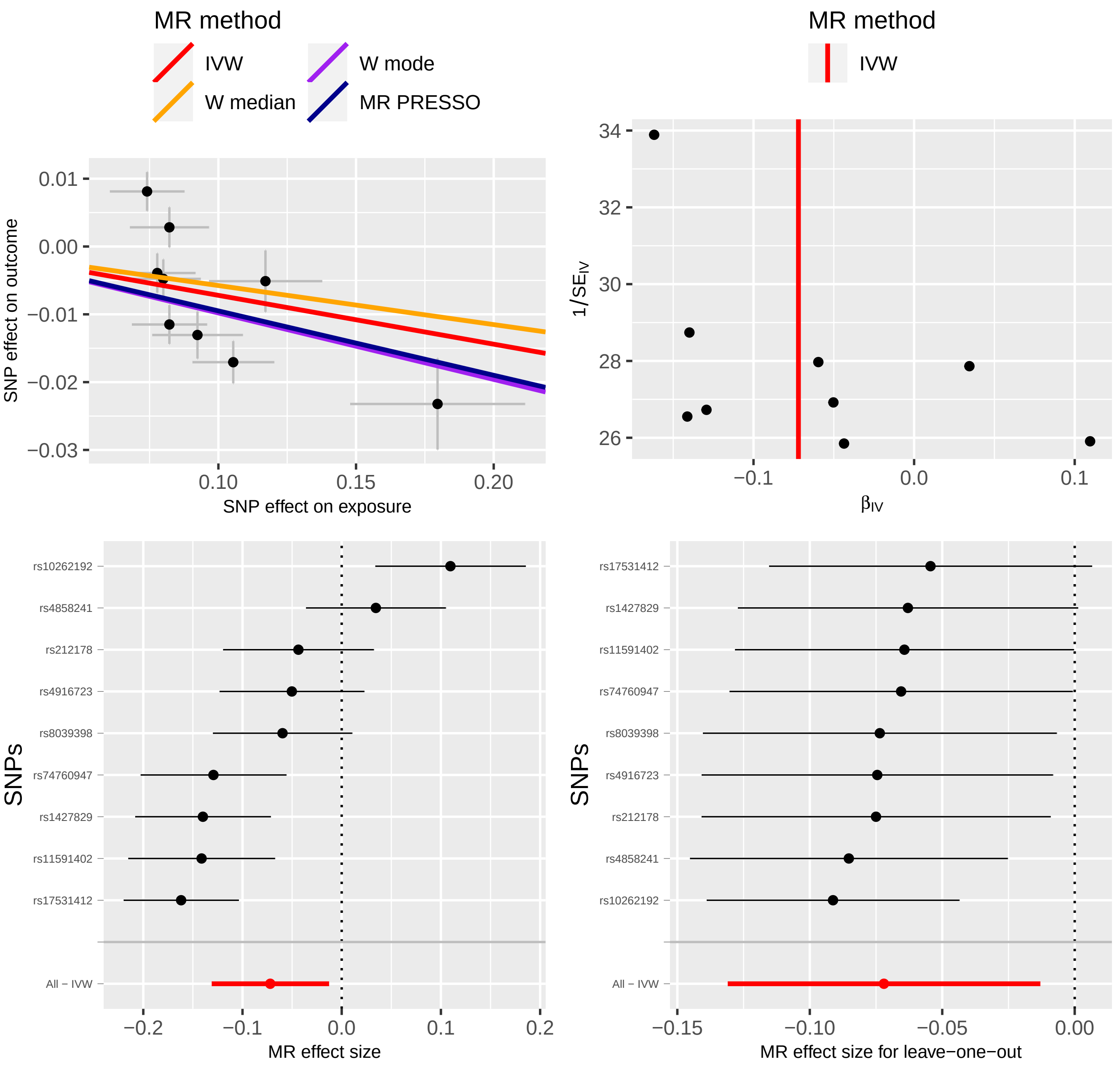

ai) ADHD → Fluid intelligence score

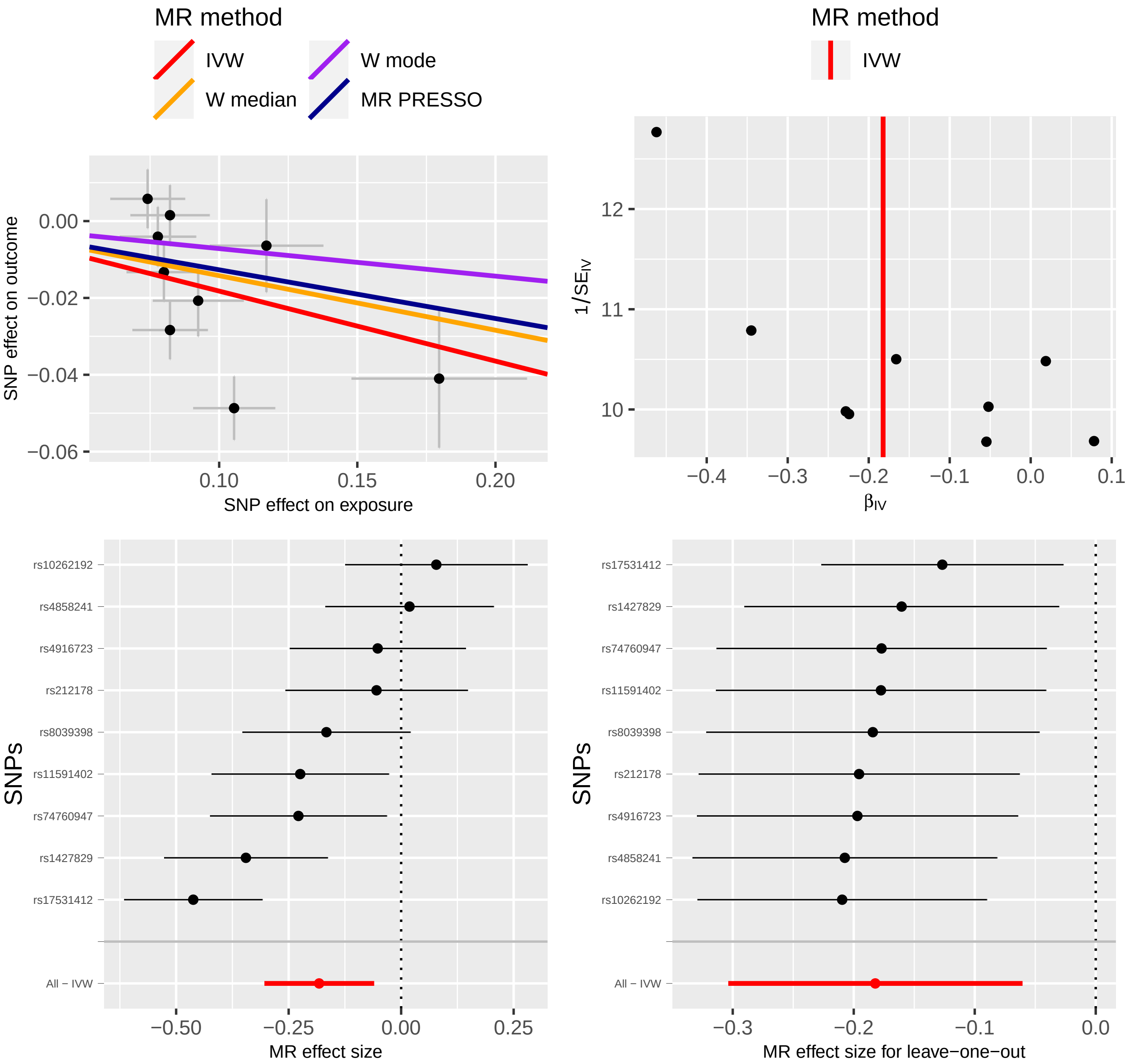

aj) Fluid intelligence score → ADHD

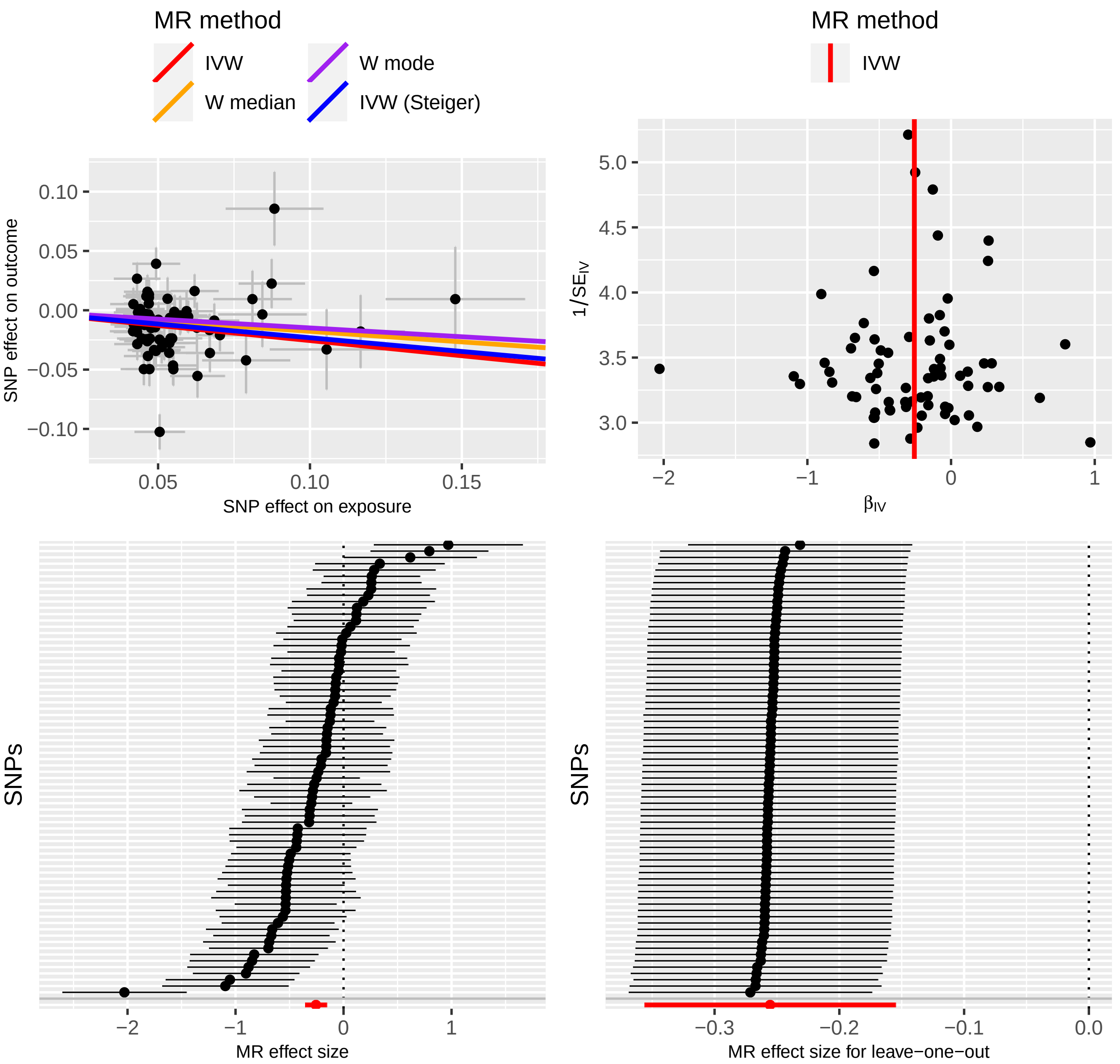

ak) Intelligence → ADHD

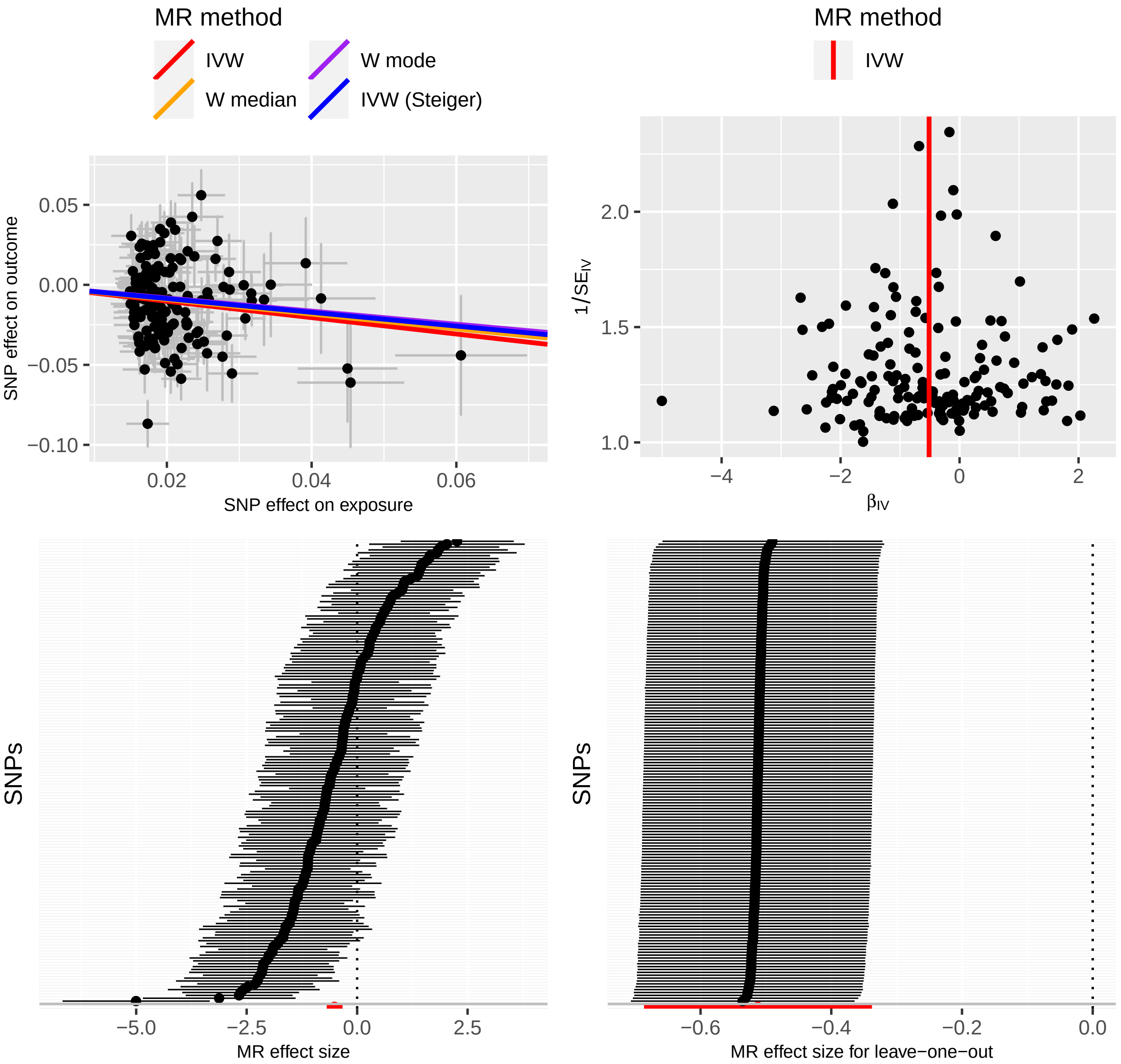

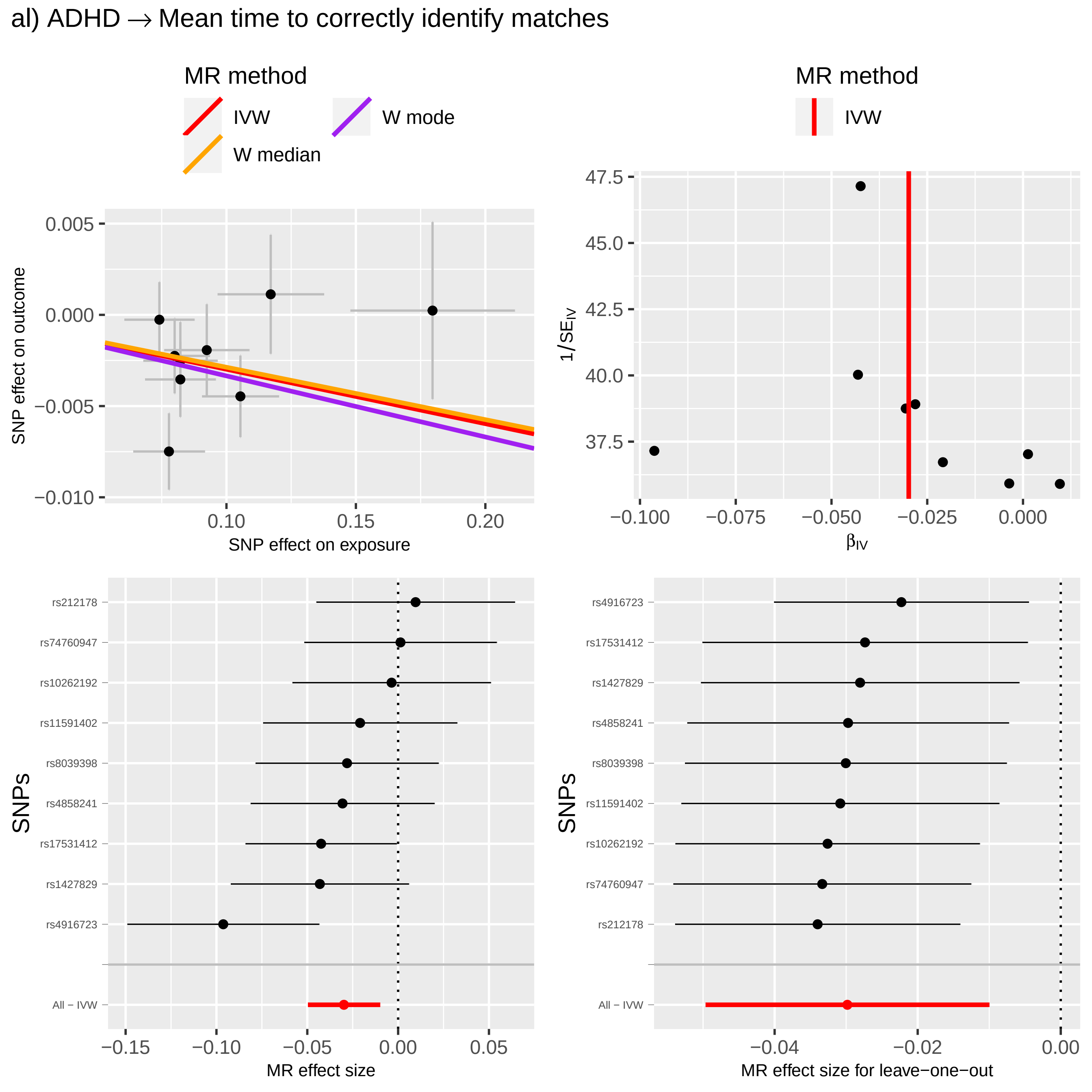
al) ADHD → Mean time to correctly identify matches
MR method
IVW
W median
W mode
SNP effect on outcome
SNP effect on exposure
0.005
0.000
−0.005
−0.010
0.10
0.15
0.20
MR method
IVW
1/SE$_{IV}$
β_{IV}
47.5
45.0
42.5
40.0
37.5
−0.100
−0.075
−0.050
−0.025
0.000
SNPs
rs212178
rs74760947
rs10262192
rs11591402
rs8039398
rs4858241
rs17531412
rs1427829
rs4916723
All − IVW
−0.15
−0.10
−0.05
0.00
0.05
MR effect size
SNPs
rs4916723
rs17531412
rs1427829
rs4858241
rs8039398
rs11591402
rs10262192
rs74760947
rs212178
All − IVW
−0.04
−0.02
0.00
MR effect size for leave−one−out

am) ADHD → Maternal smoking around birth

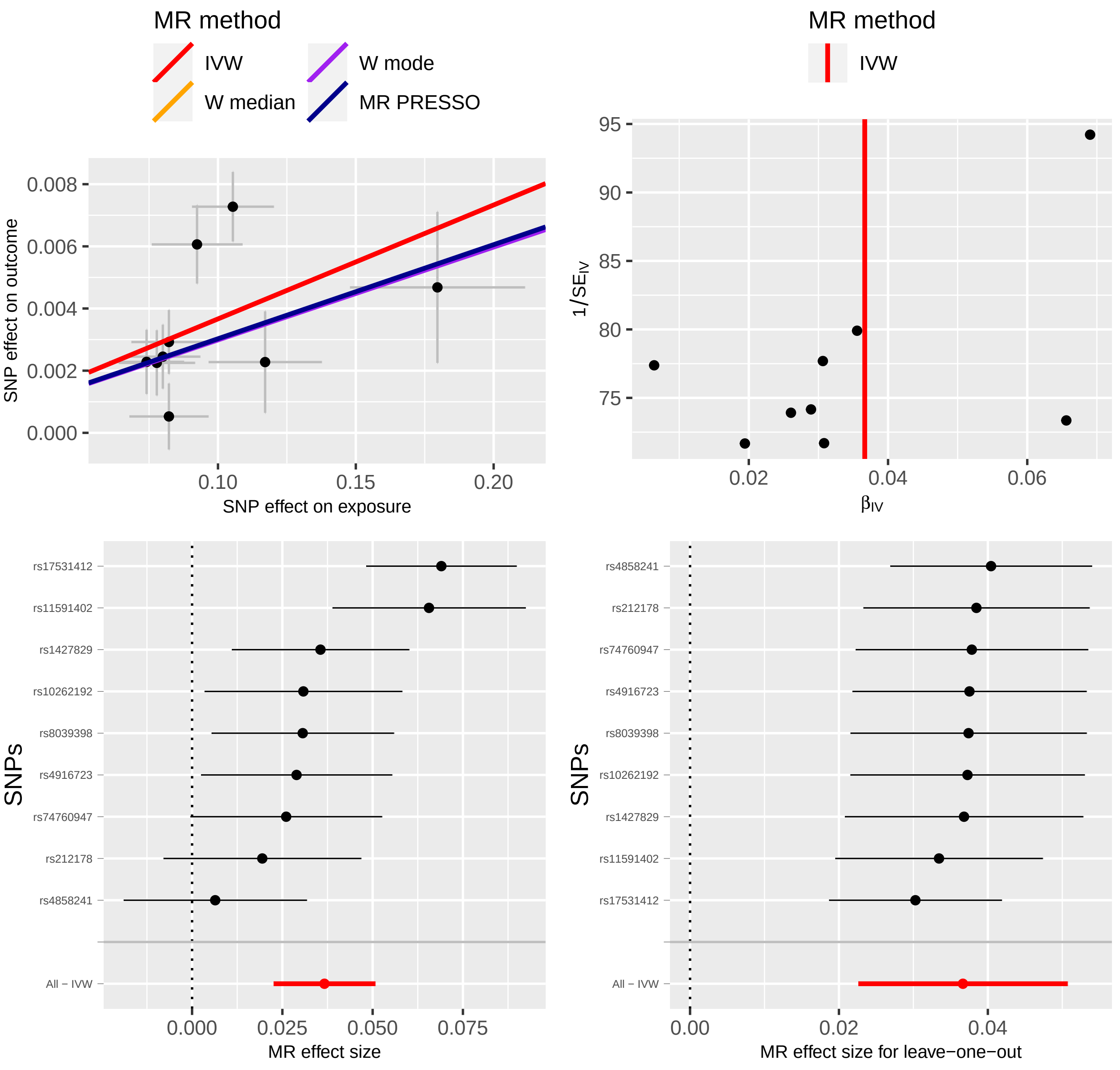

an) Maternal smoking around birth → ADHD

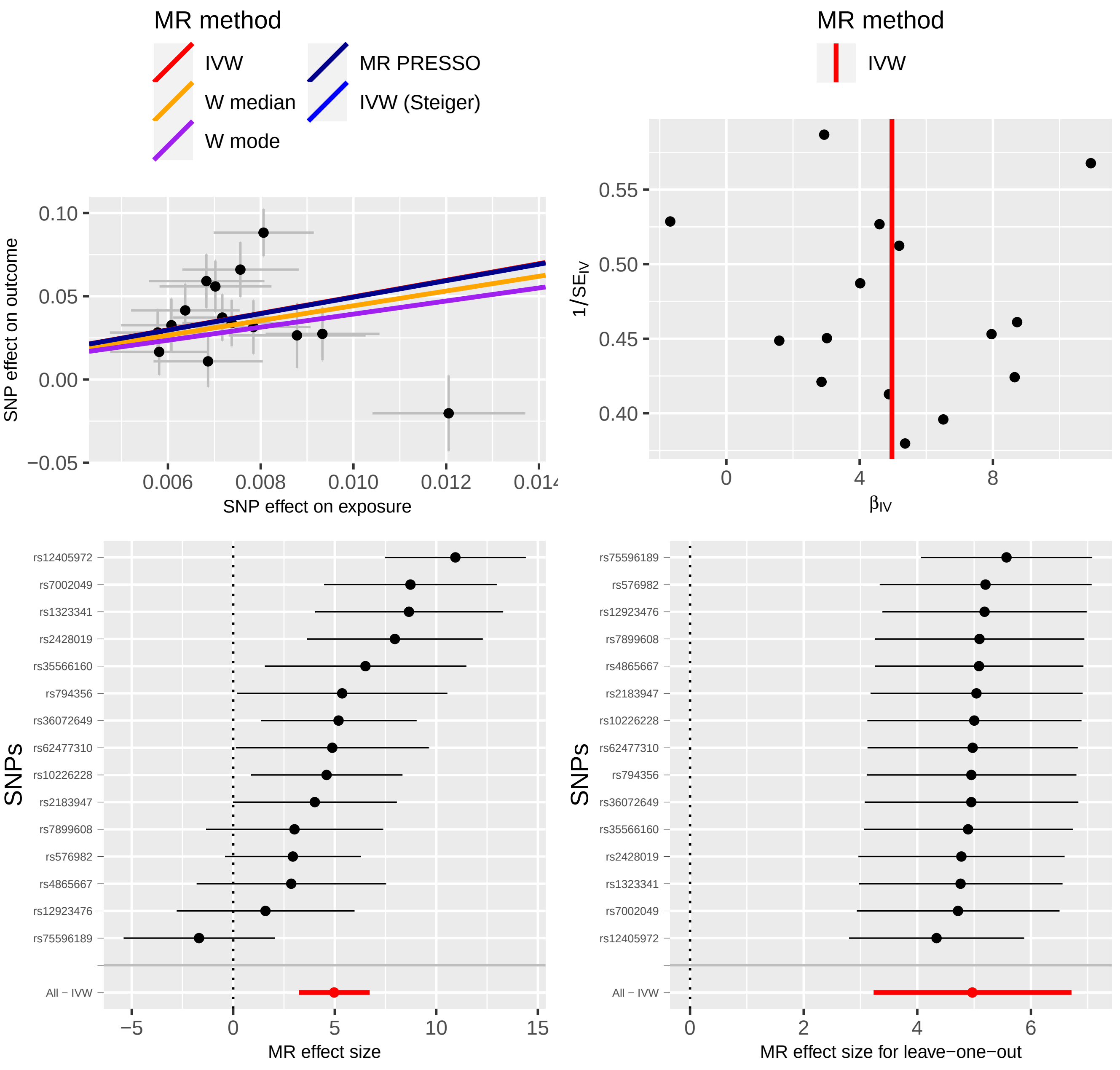

ao) Age completed full time education → ADHD

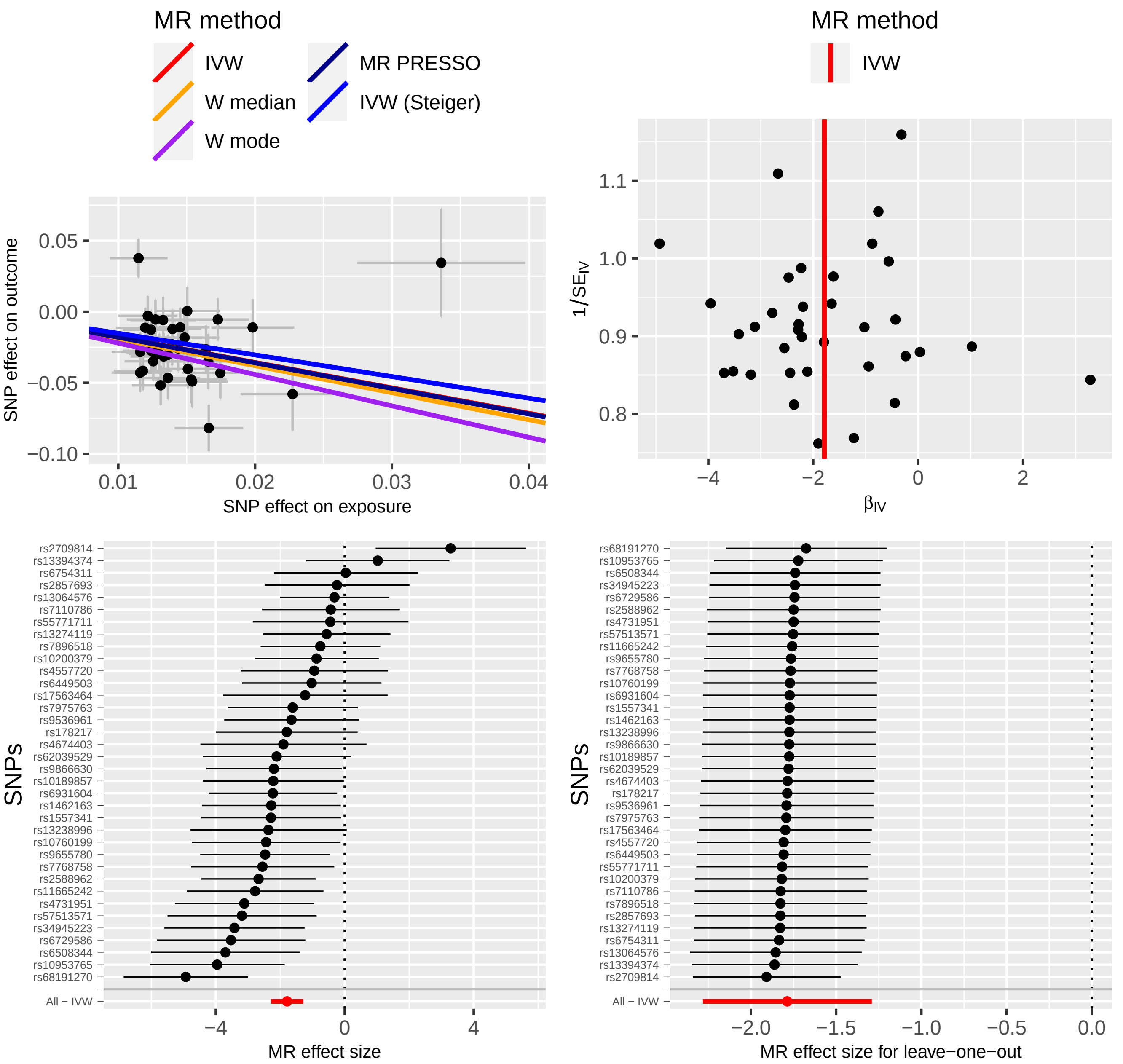

ap) ADHD → Age completed full time education

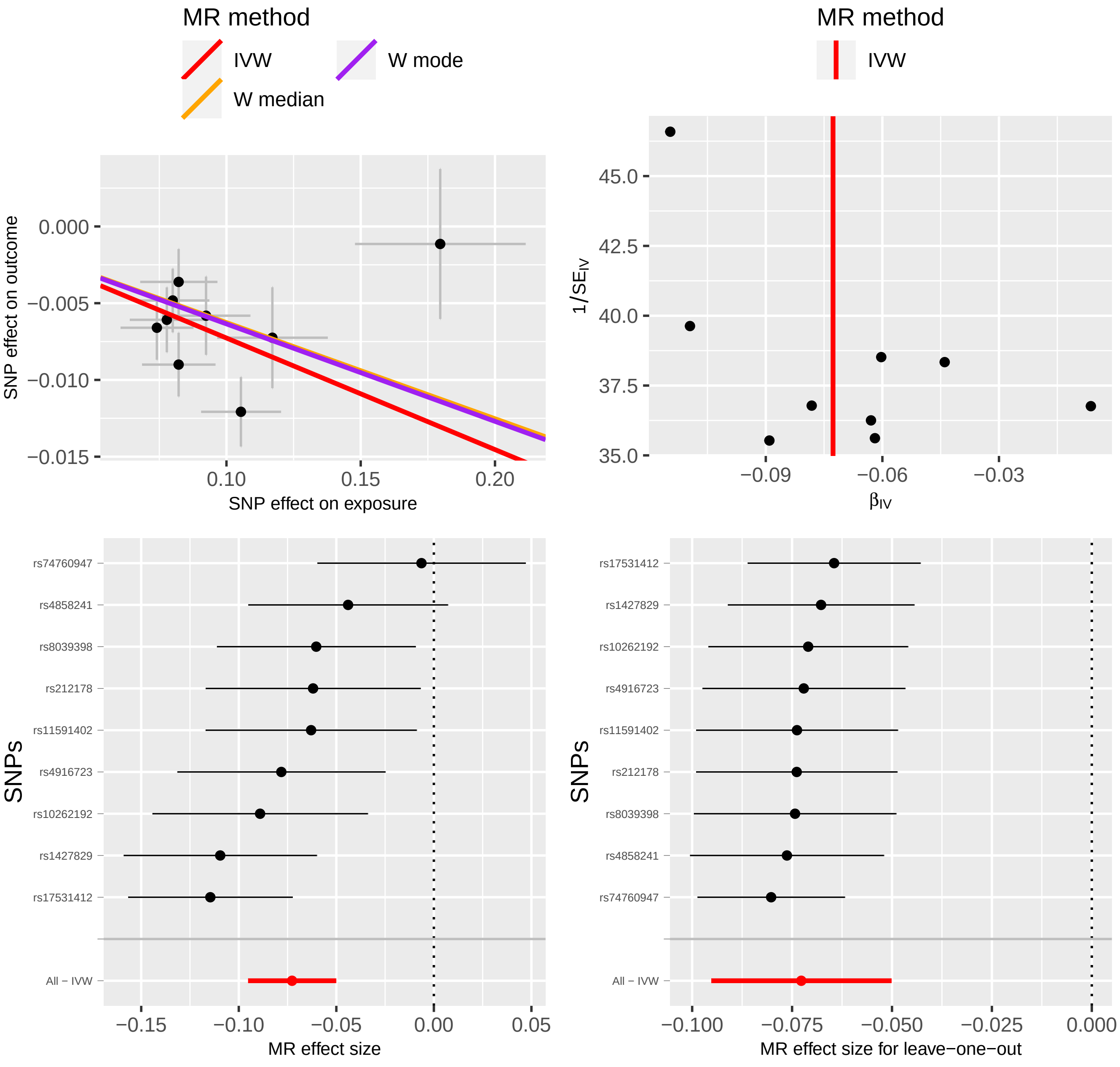

aq) ADHD → Job involves heavy manual or physical work

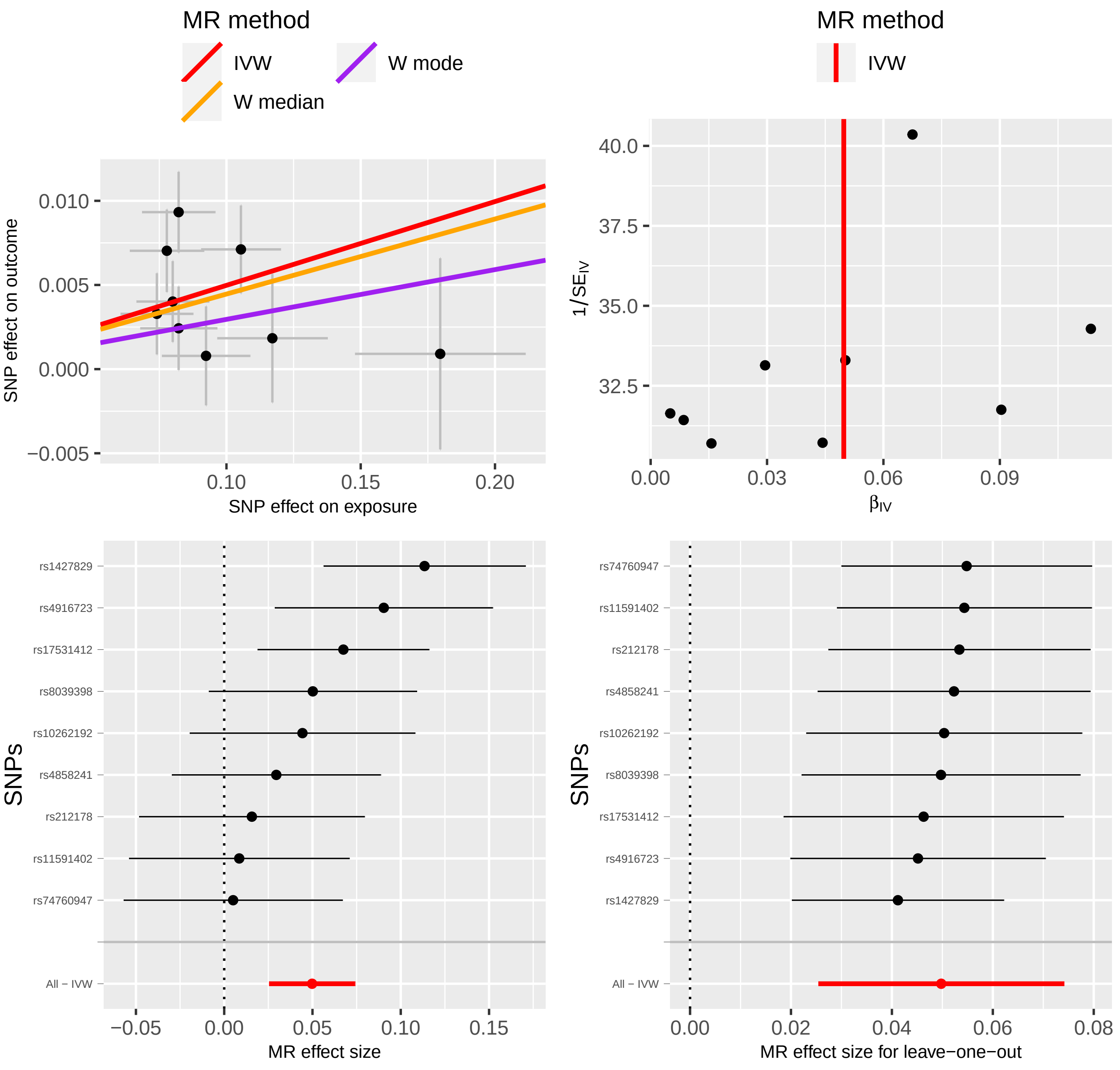

ar) Job involves heavy manual or physical work → ADHD

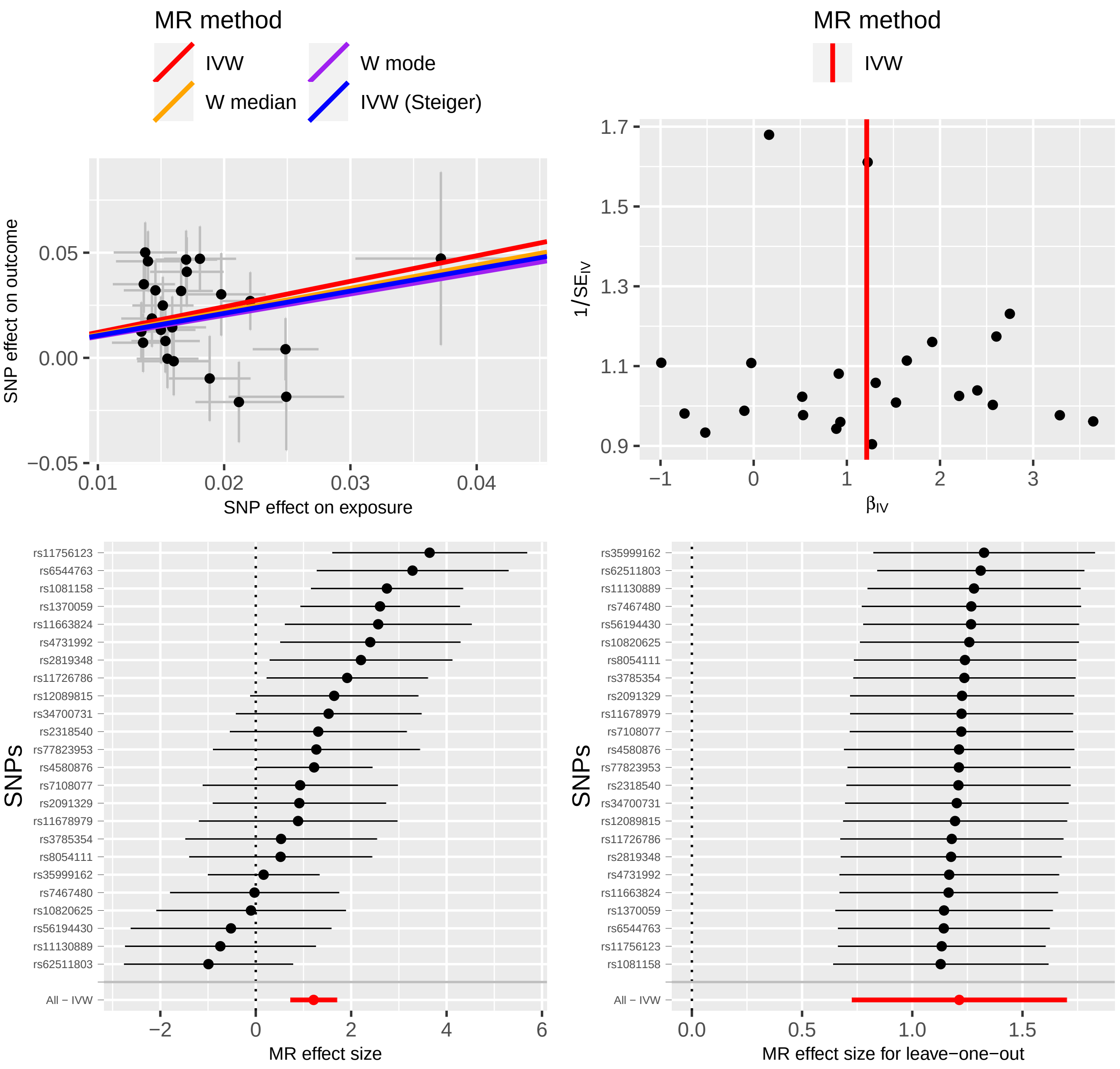

as) Job involves mainly walking or standing → ADHD

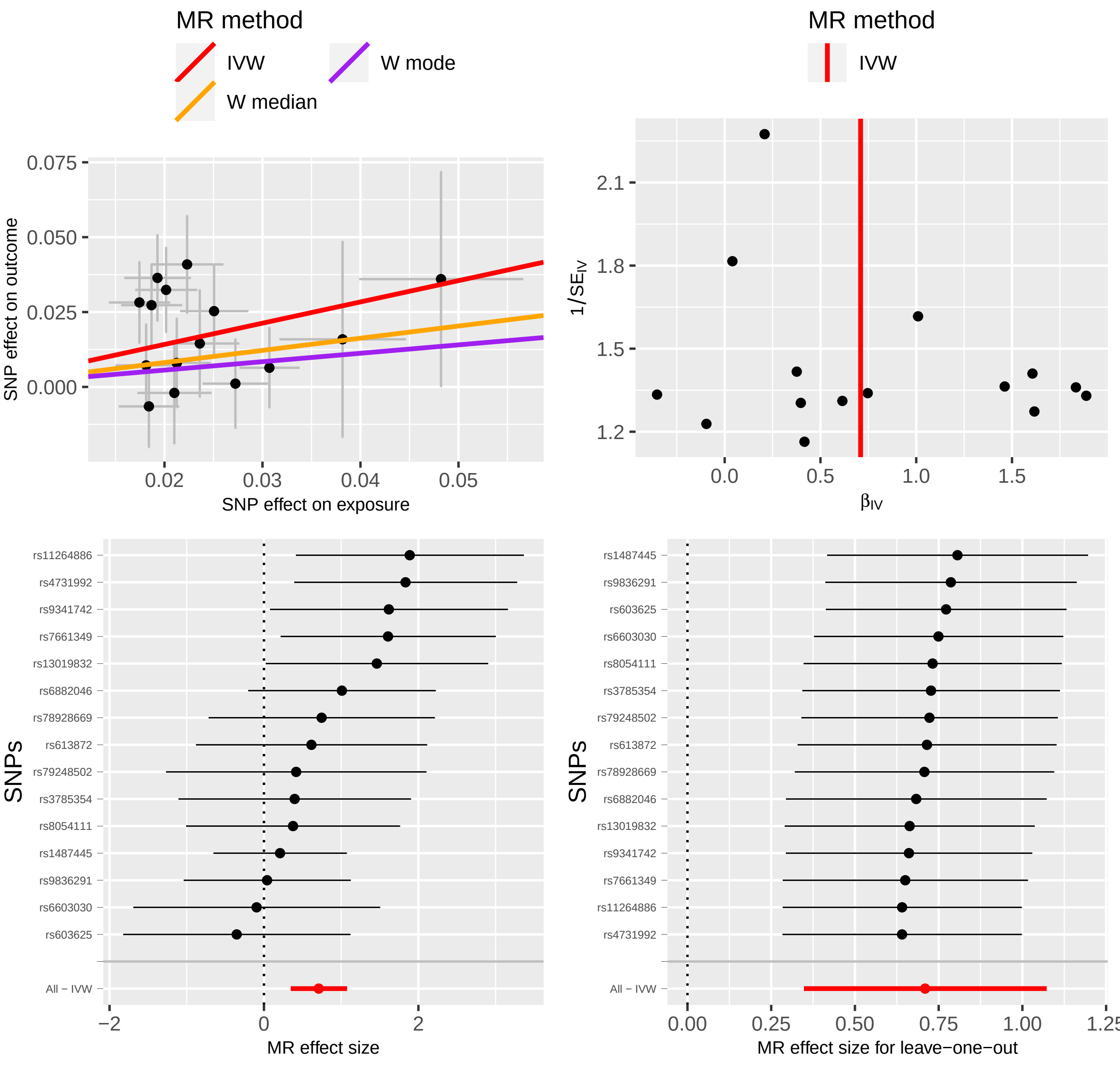

at) ADHD → Job involves mainly walking or standing

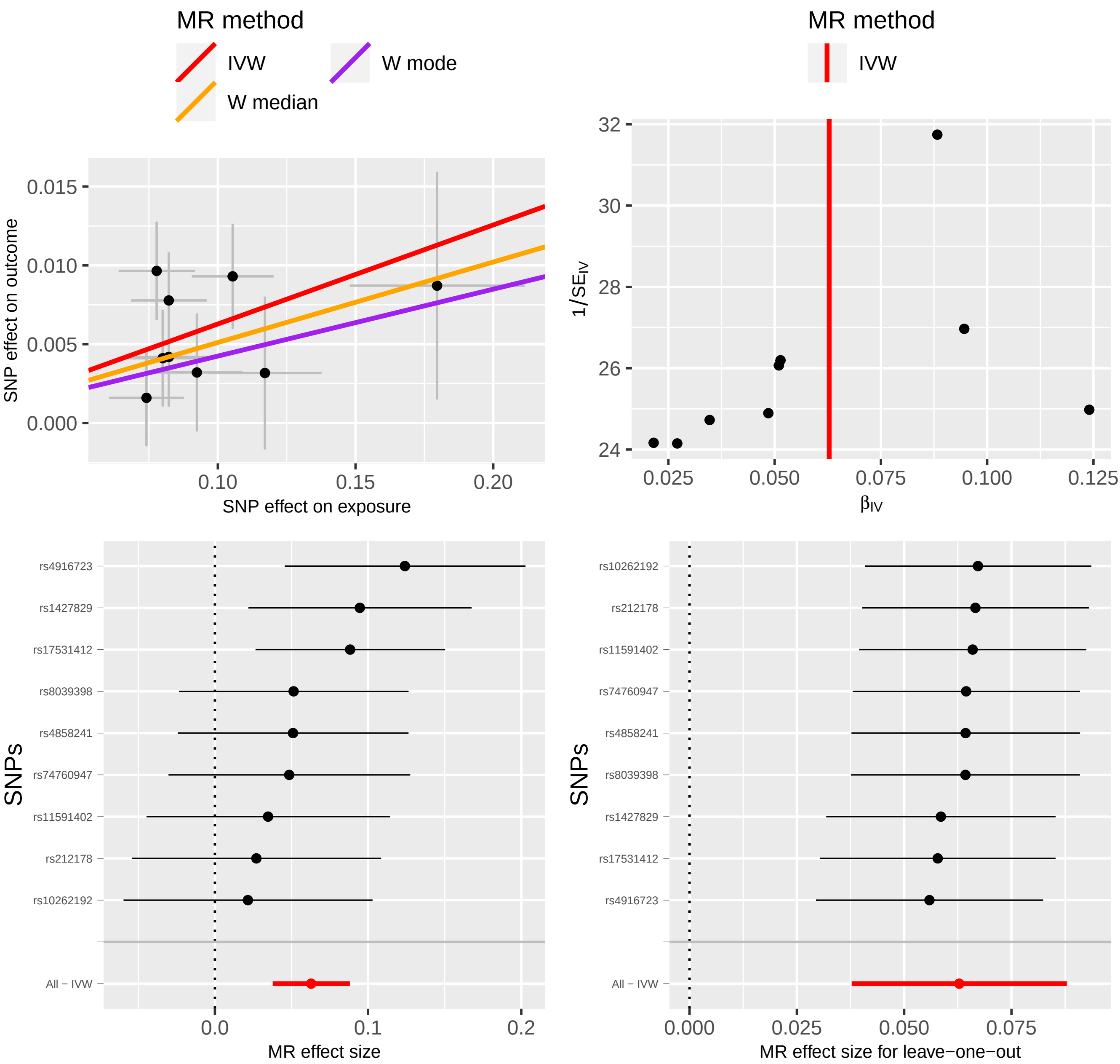

au) ADHD → Years of schooling

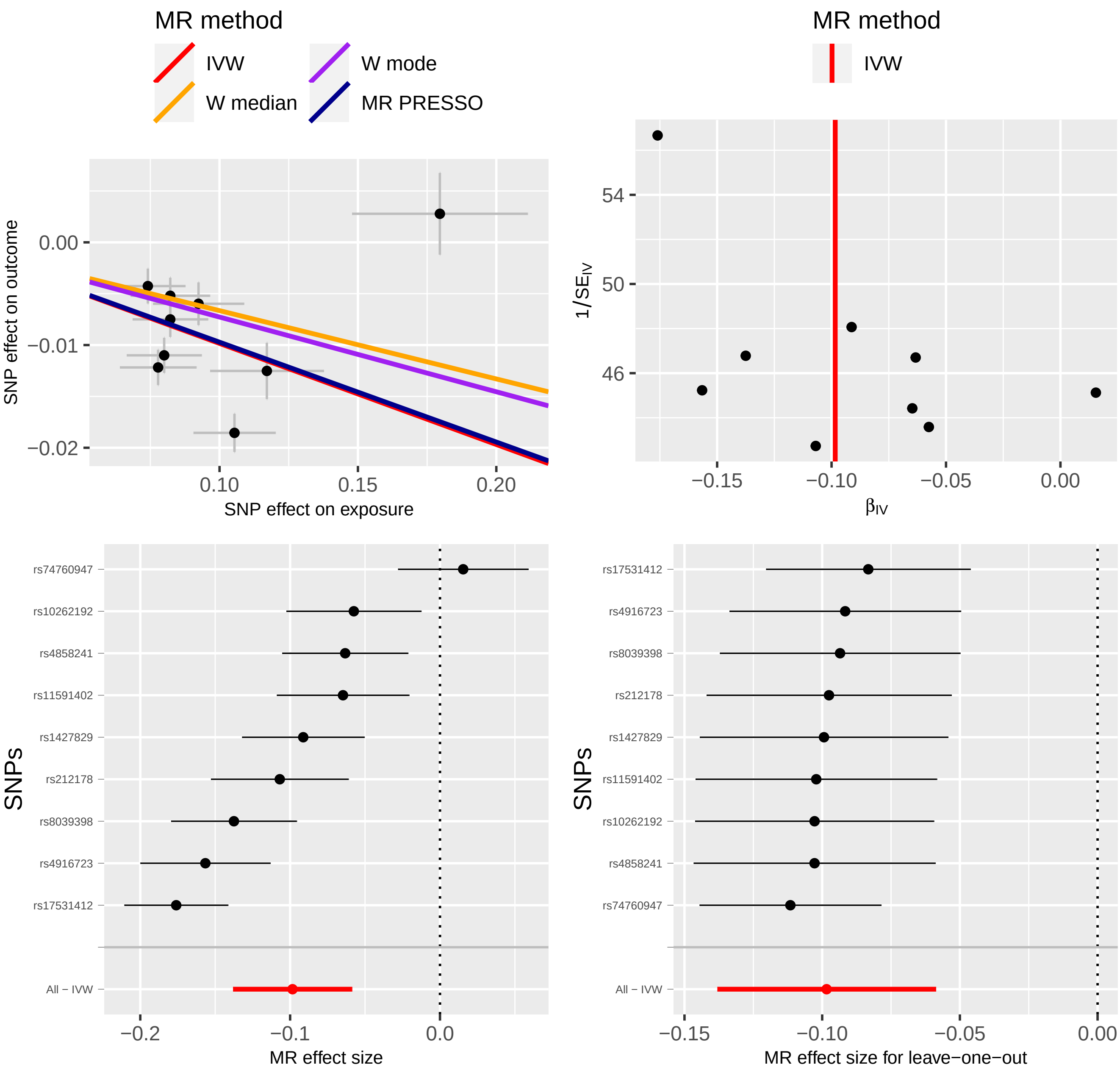

av) Years of schooling → ADHD

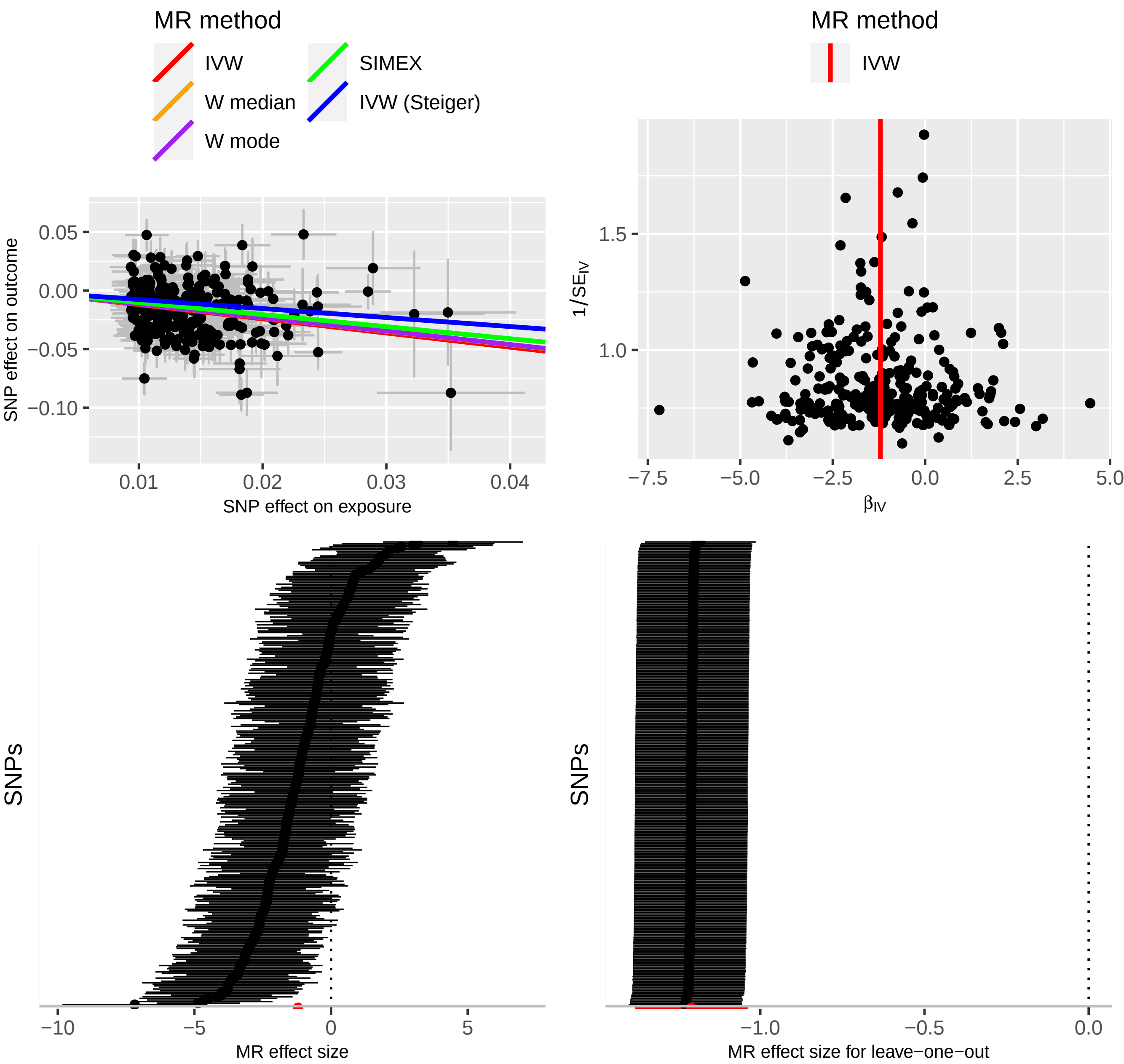

aw) Alcohol intake frequency. → ADHD

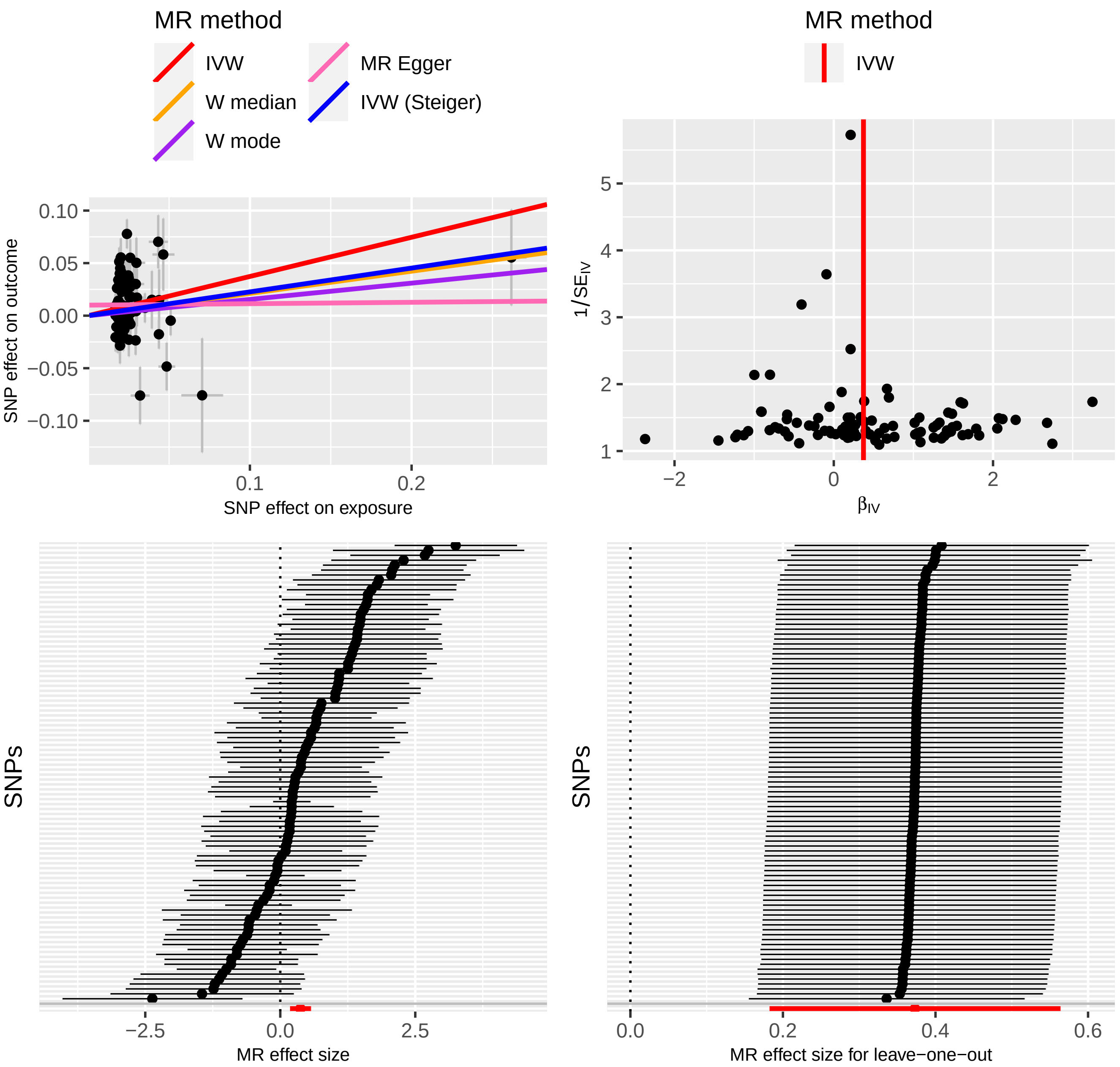

ax) ADHD → Alcohol intake frequency.

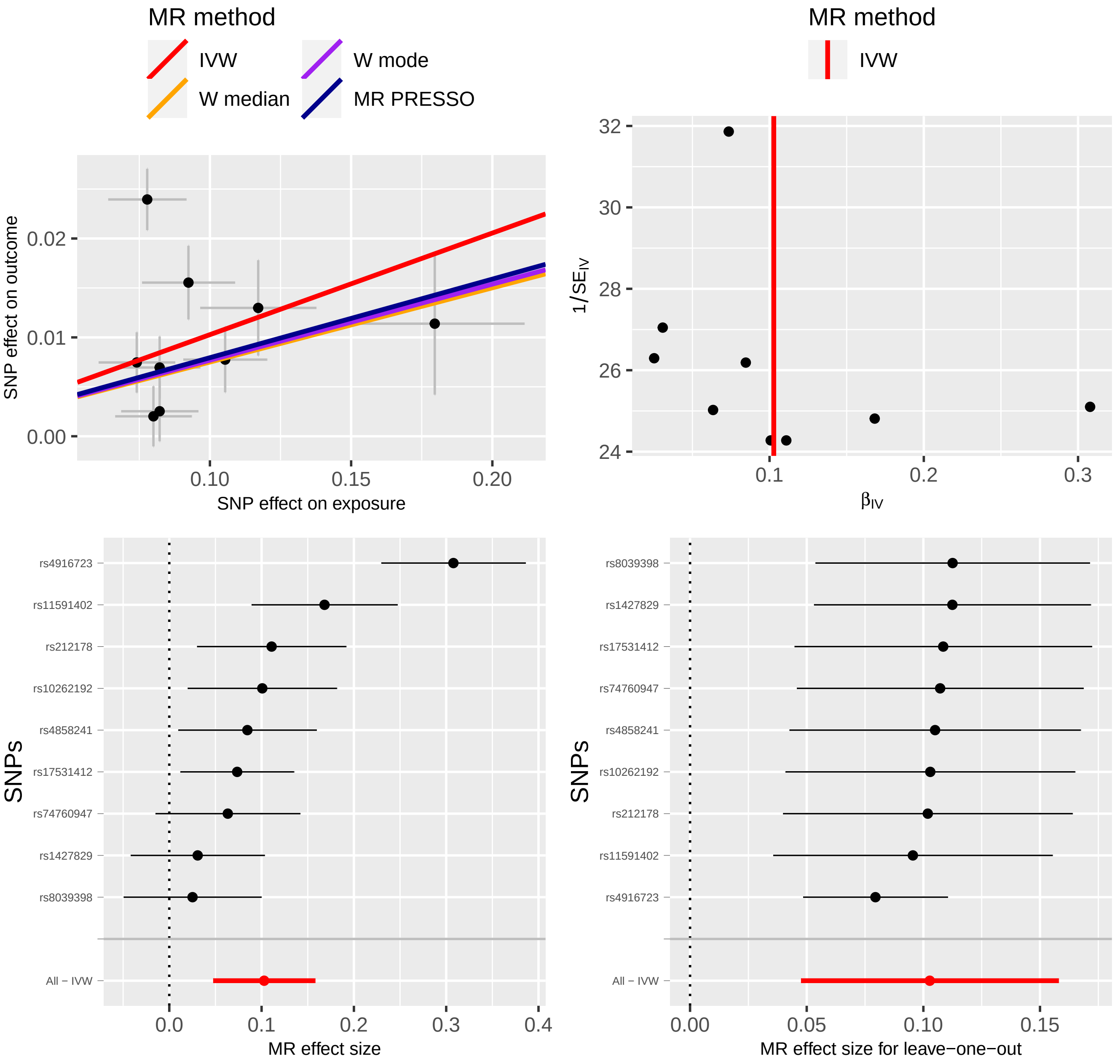

ay) Alcohol intake versus 10 years previously → ADHD

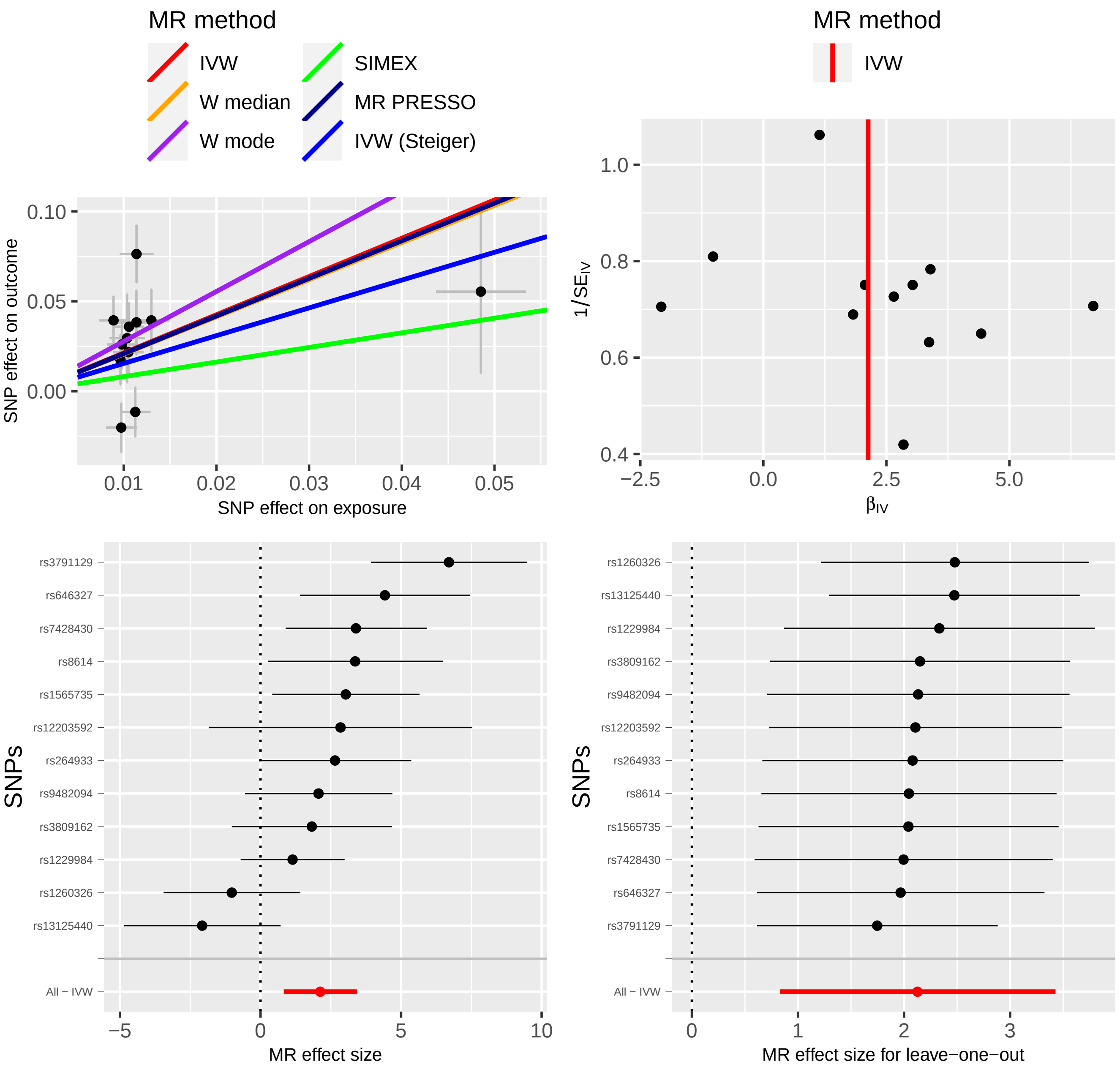

az) ADHD → Alcohol intake versus 10 years previously

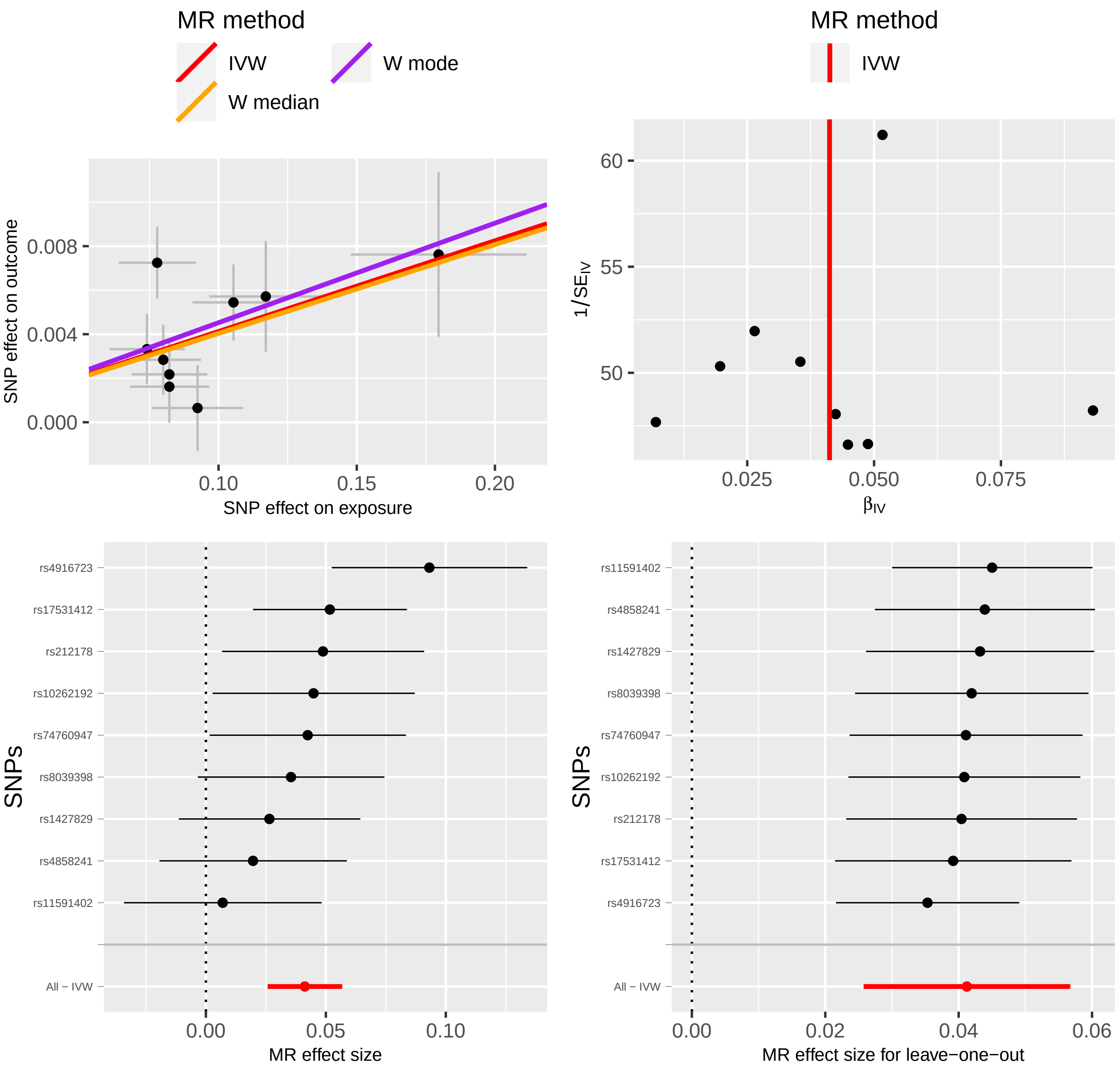

ba) Alcohol usually taken with meals → ADHD

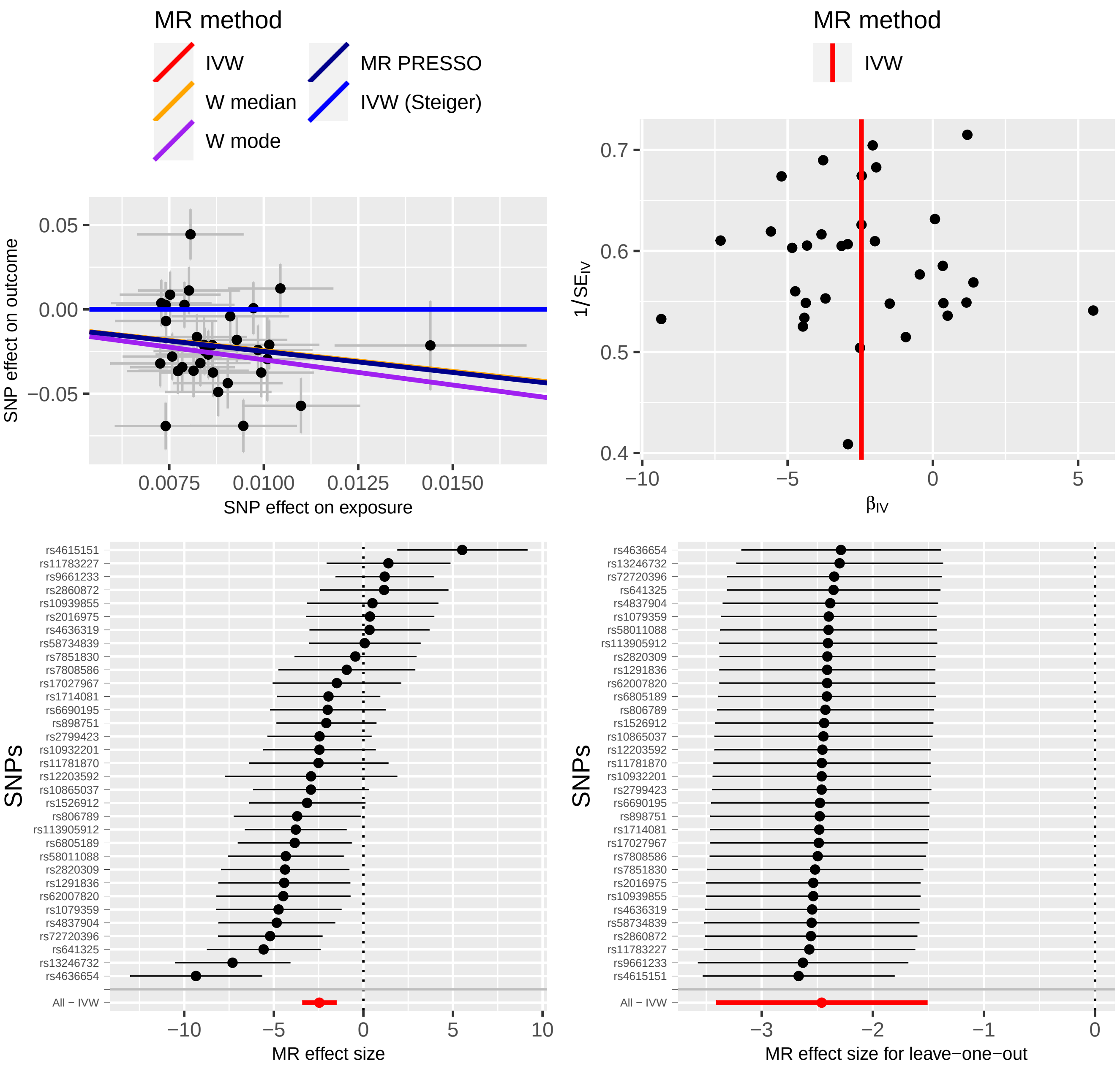

bb) ADHD → Alcohol usually taken with meals

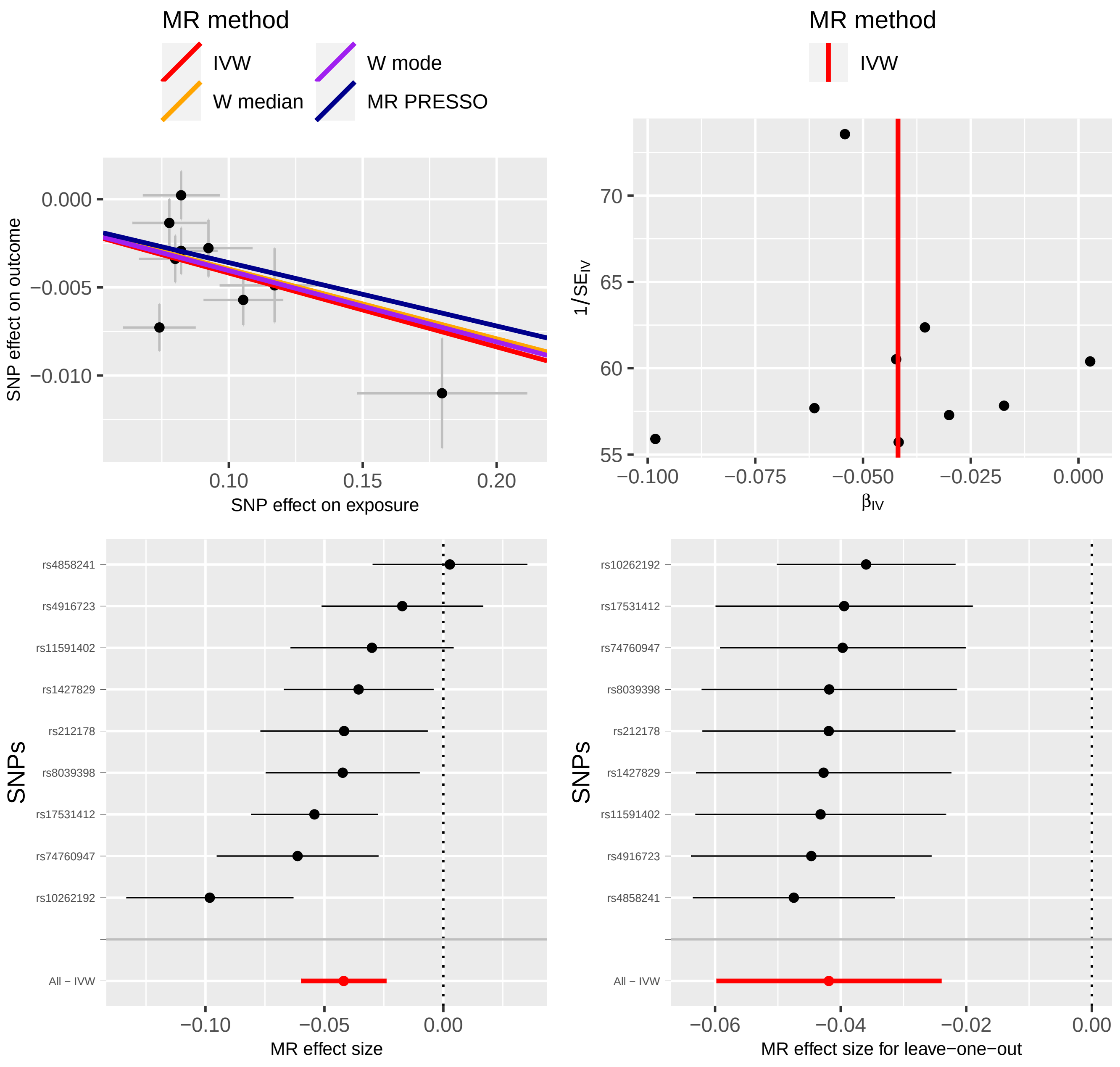

bc) Average weekly beer plus cider intake → ADHD

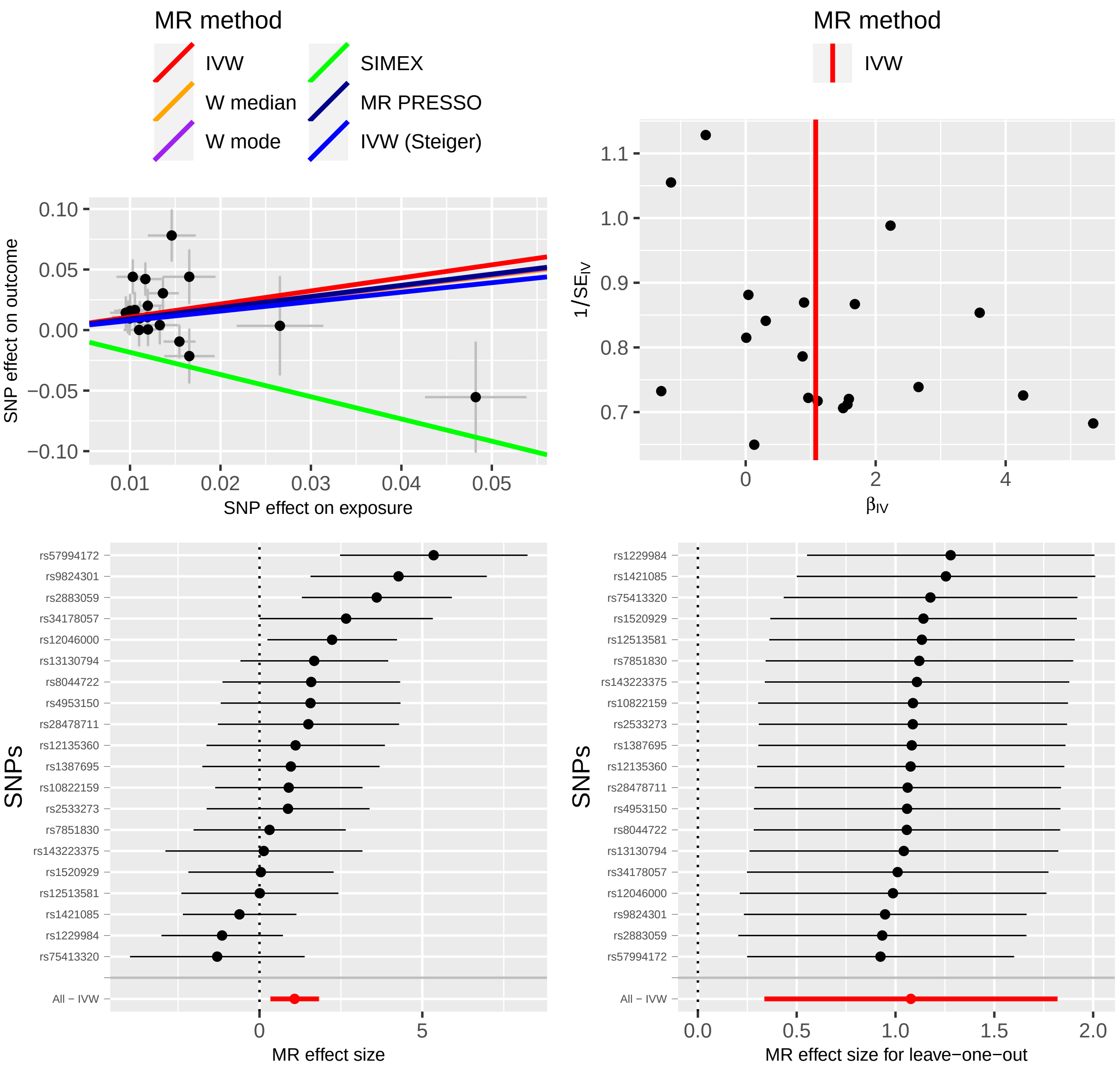

bd) ADHD → Average weekly beer plus cider intake

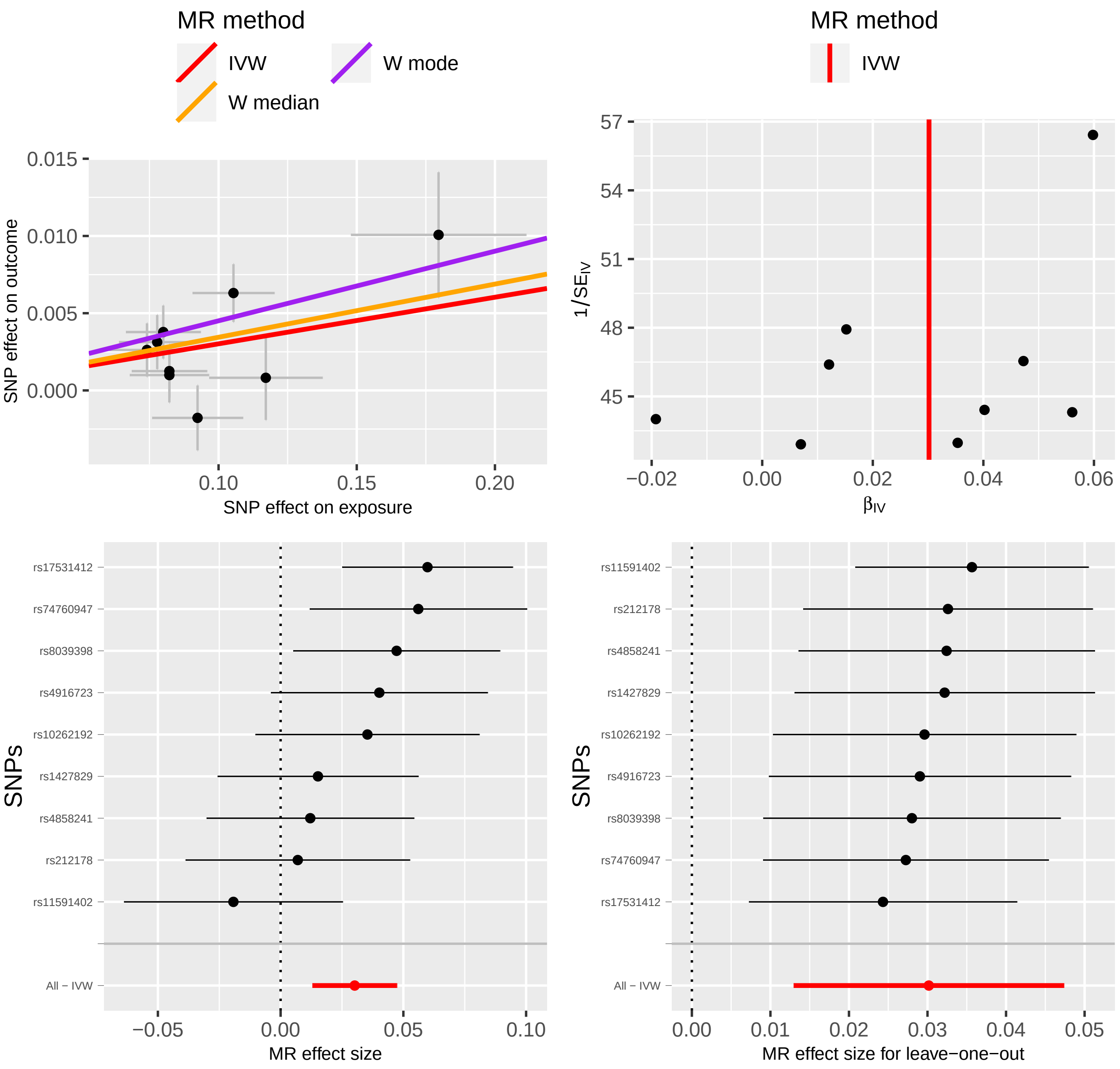

be) Average weekly champagne plus white wine intake → ADHD

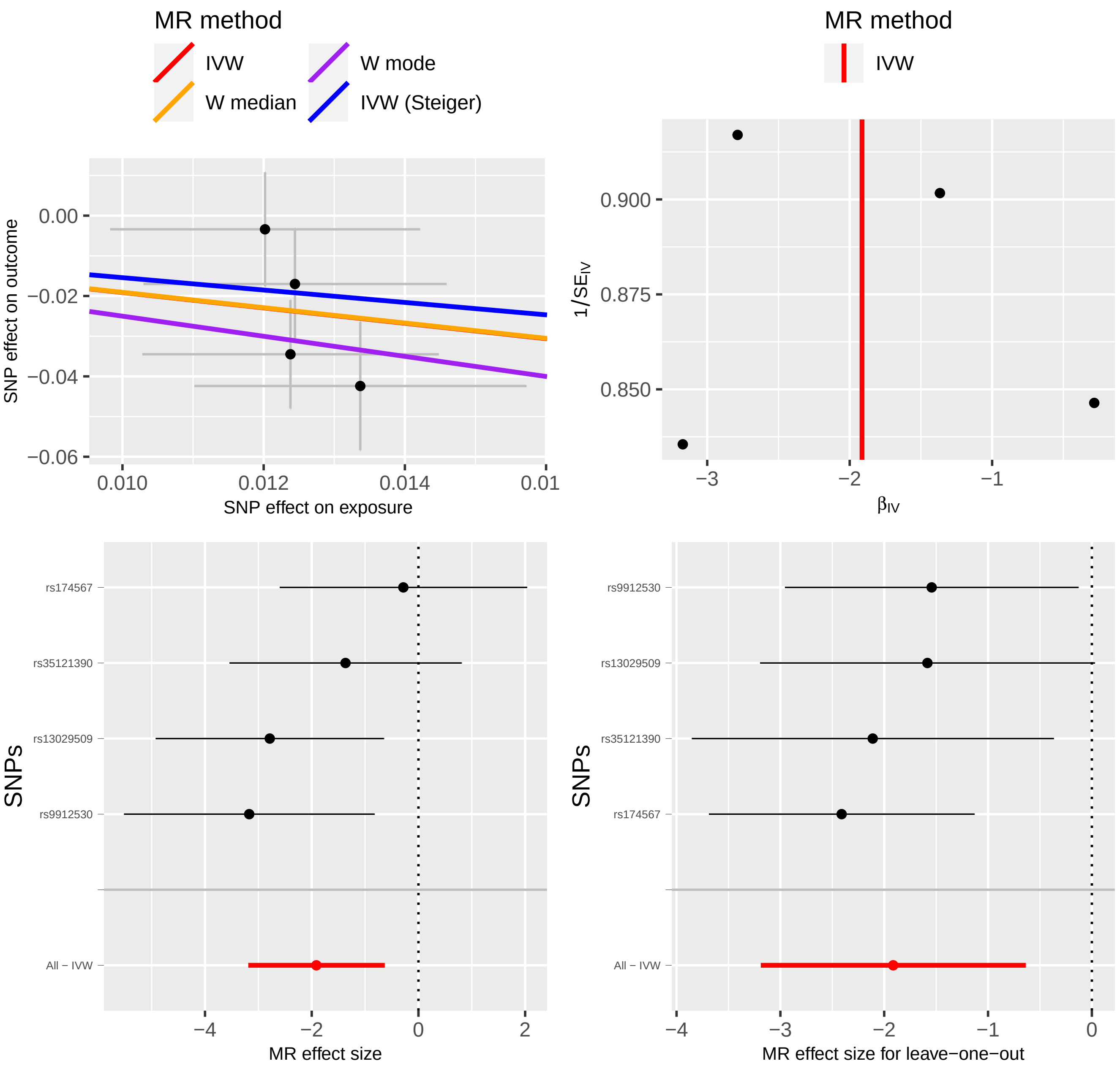

bf) ADHD → Average weekly champagne plus white wine intake

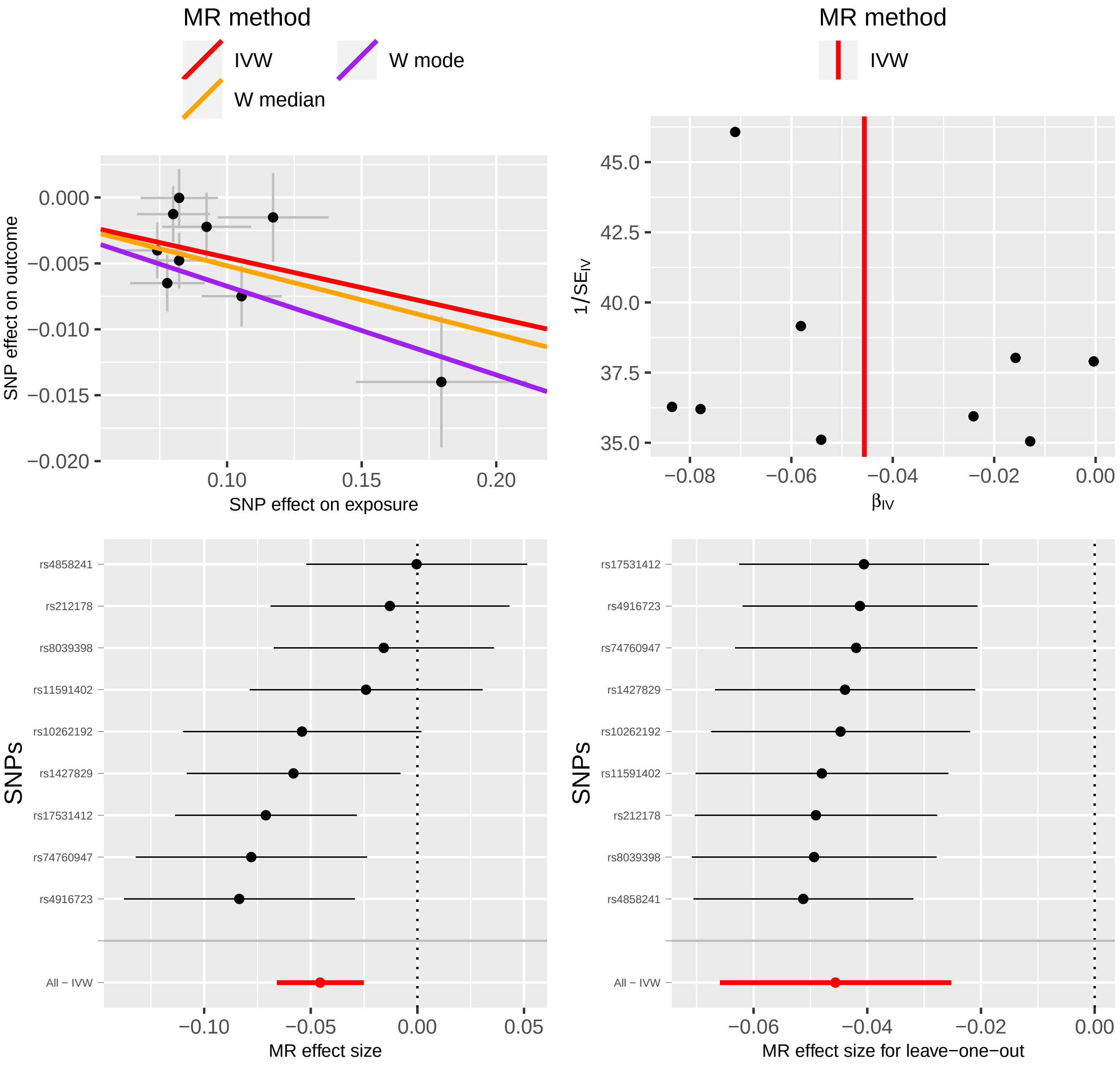

bg) ADHD → Average weekly red wine intake

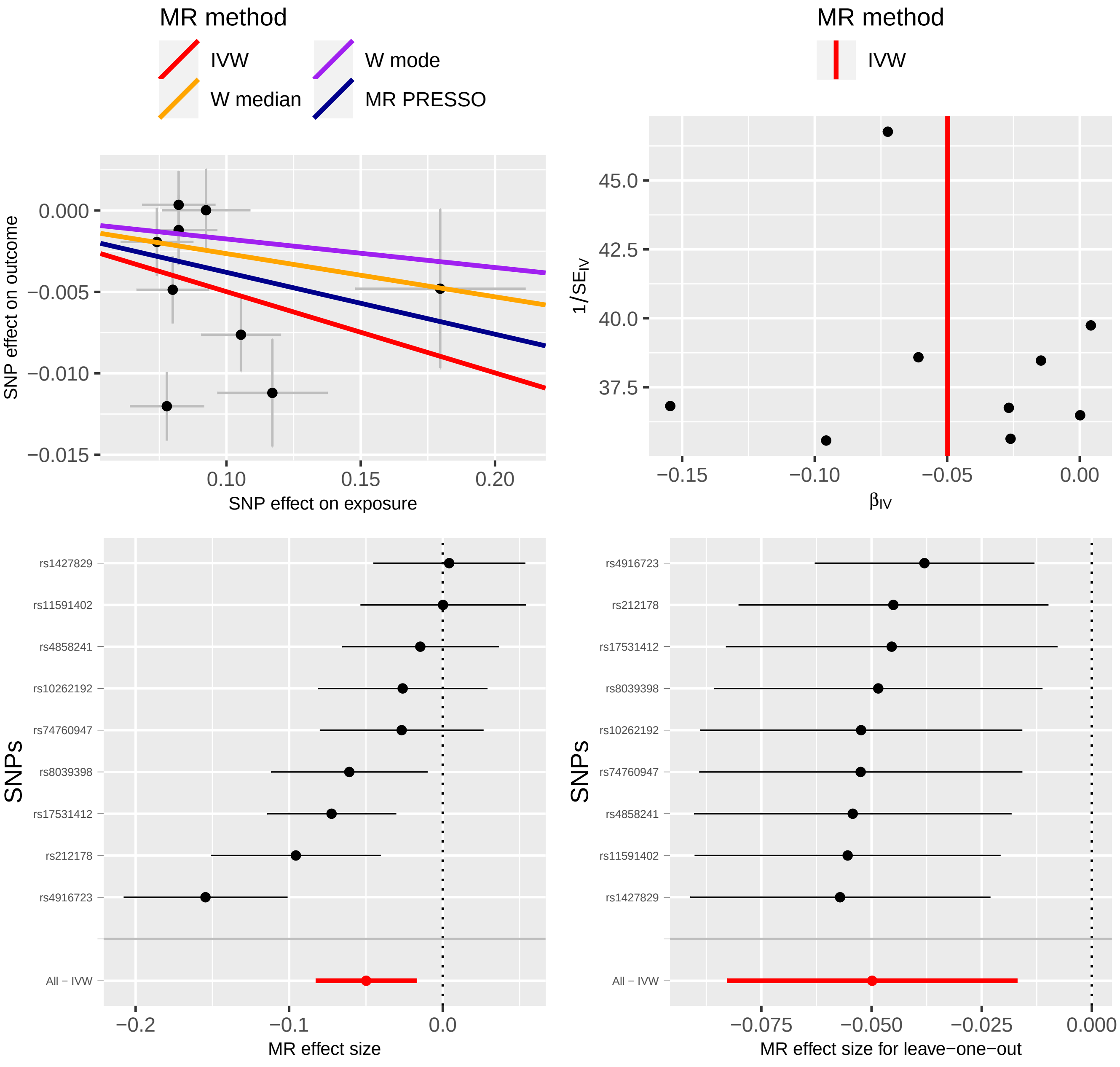

bh) ADHD → Duration of moderate activity

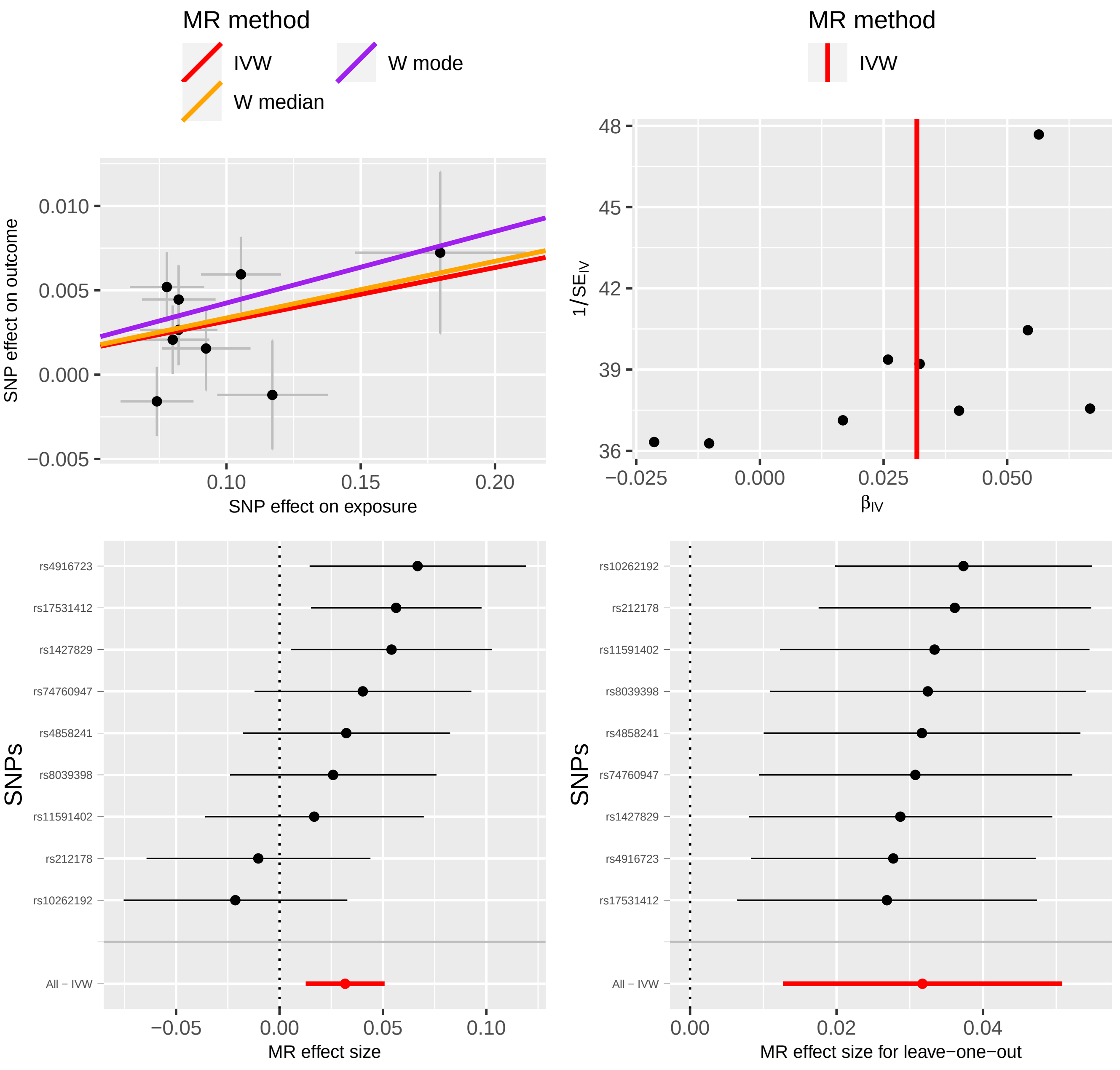

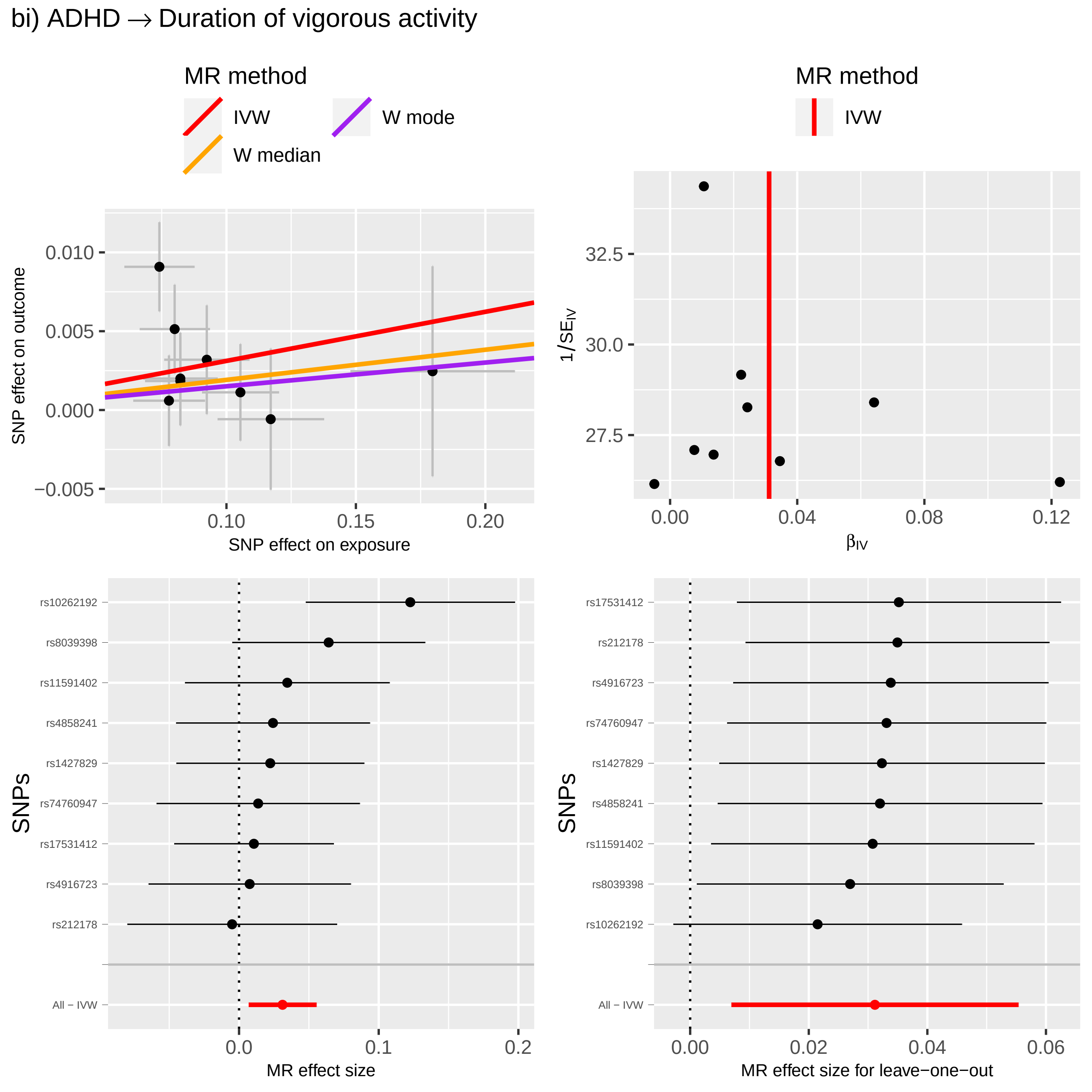

bi) ADHD → Duration of vigorous activity
MR method
IVW
W mode
W median
SNP effect on outcome
SNP effect on exposure
1/SE_IV
β_IV
SNPs
rs10262192
rs8039398
rs11591402
rs4858241
rs1427829
rs74760947
rs17531412
rs4916723
rs212178
All − IVW
MR effect size
rs17531412
rs212178
rs4916723
rs74760947
rs1427829
rs4858241
rs11591402
rs8039398
rs10262192
All − IVW
MR effect size for leave−one−out

bj) Frequency of stair climbing in last 4 weeks → ADHD

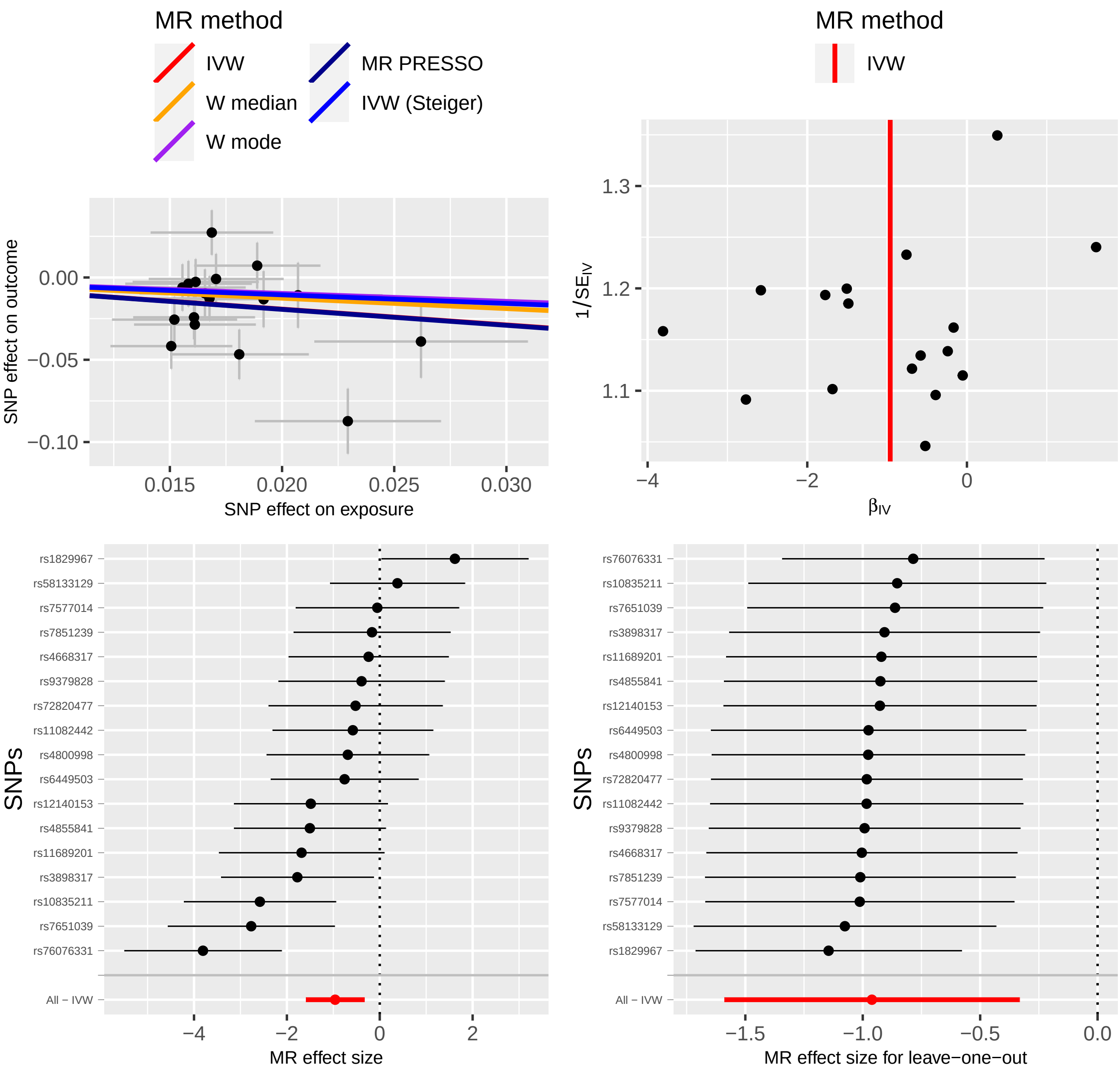

bk) ADHD → Frequency of stair climbing in last 4 weeks

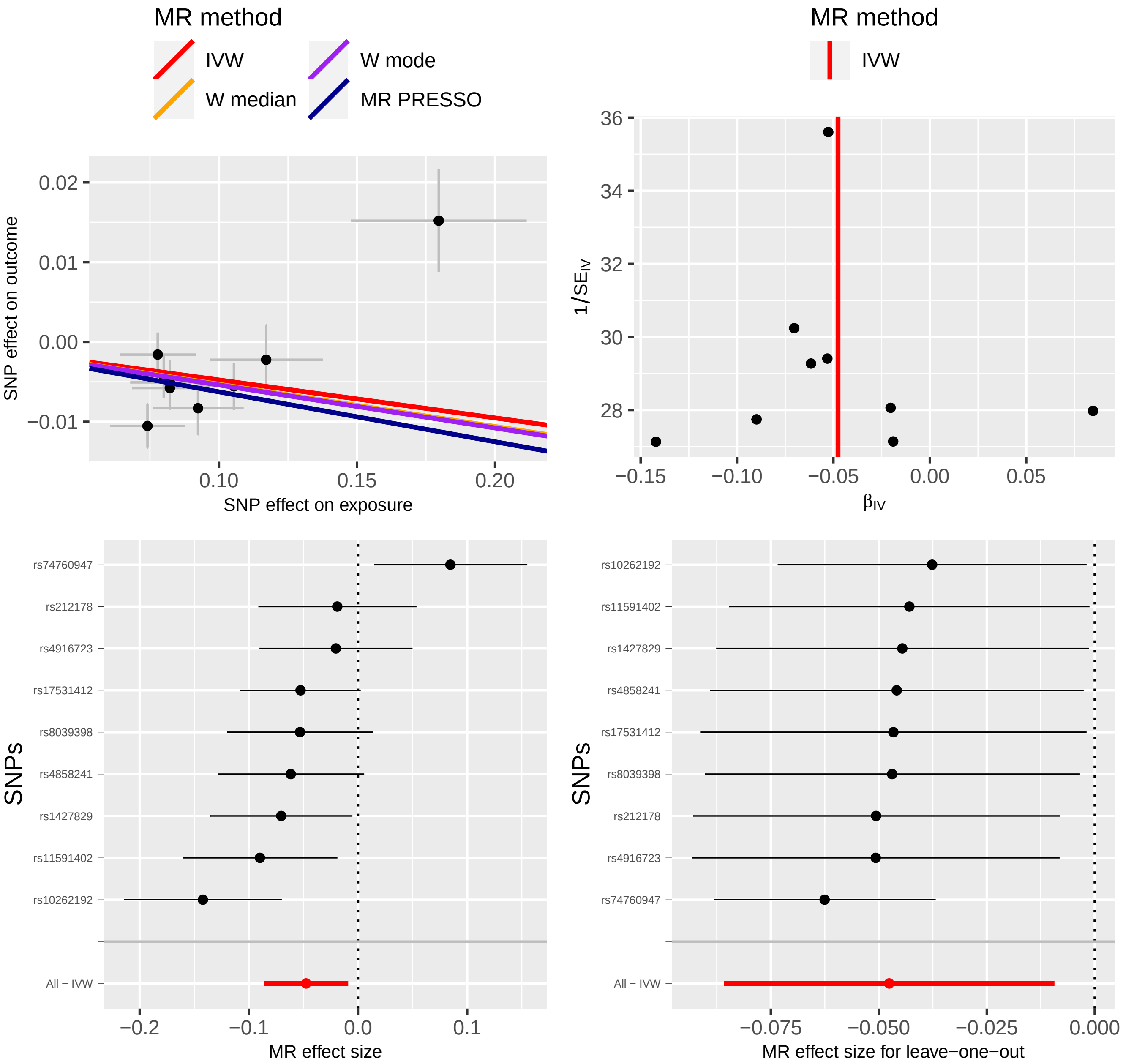

bl) ADHD → Nitrogen dioxide air pollution; 2010

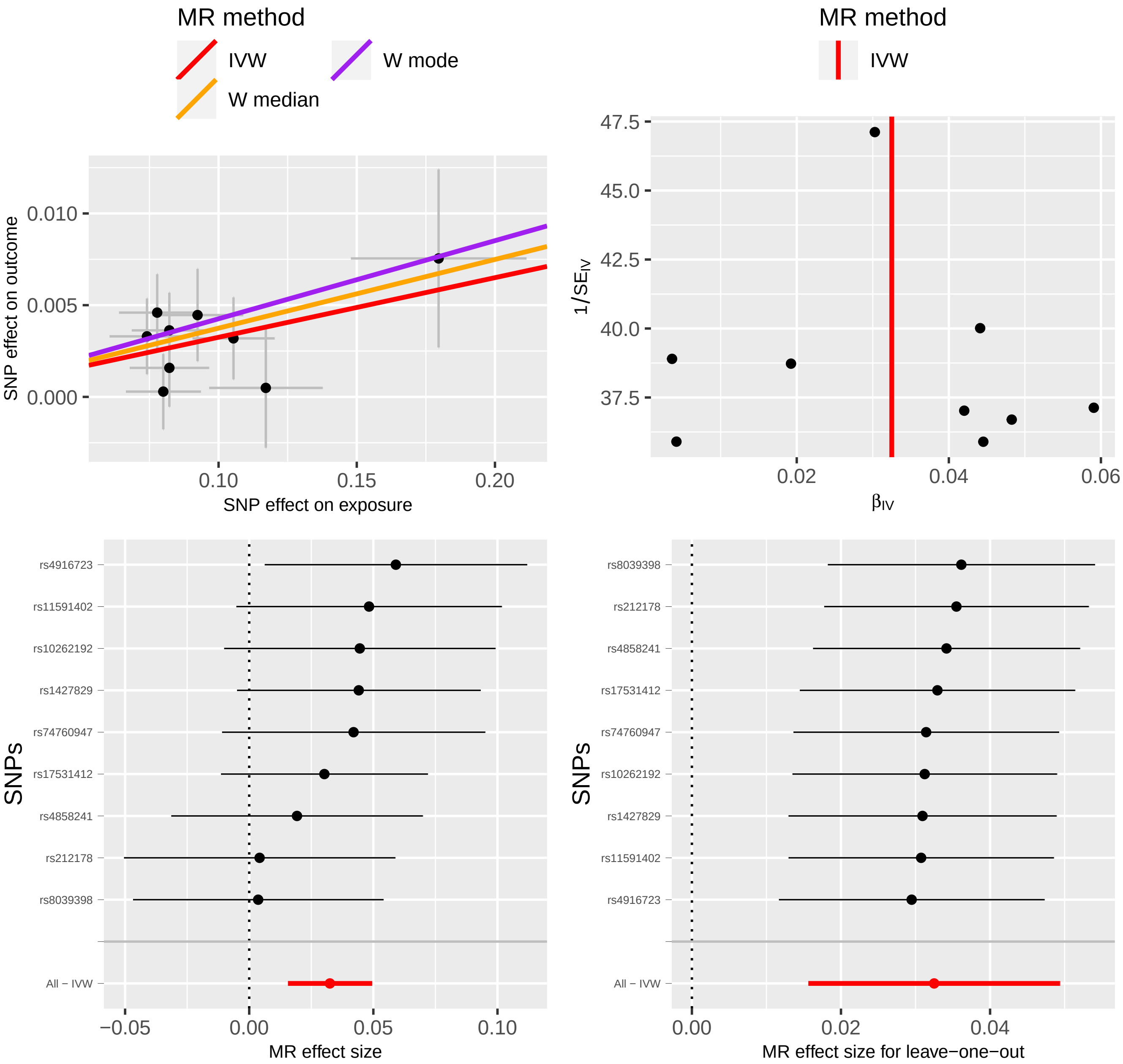

bm) ADHD → Nitrogen oxides air pollution; 2010

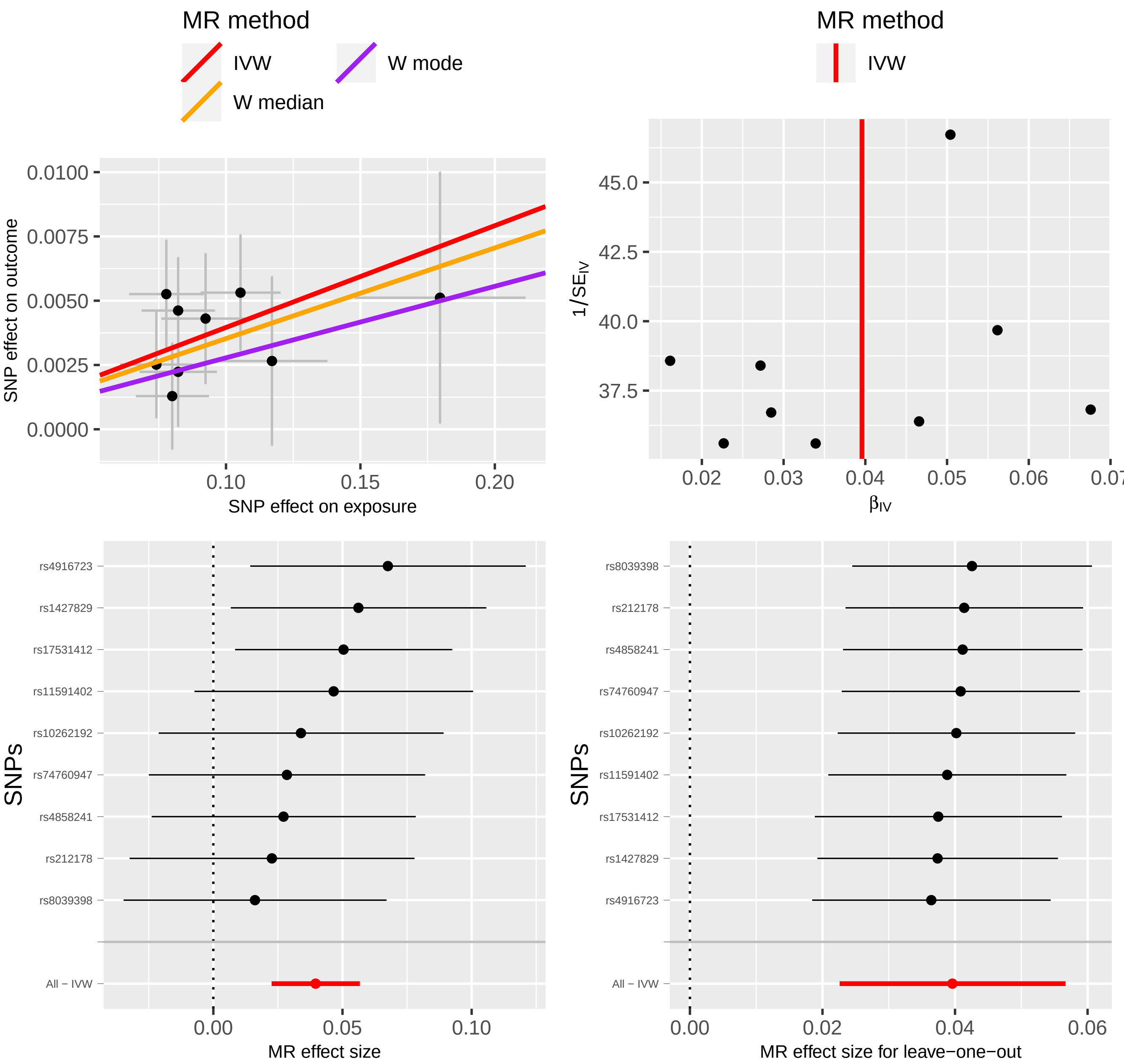

bn) ADHD → Particulate matter air pollution (pm2.5); 2010

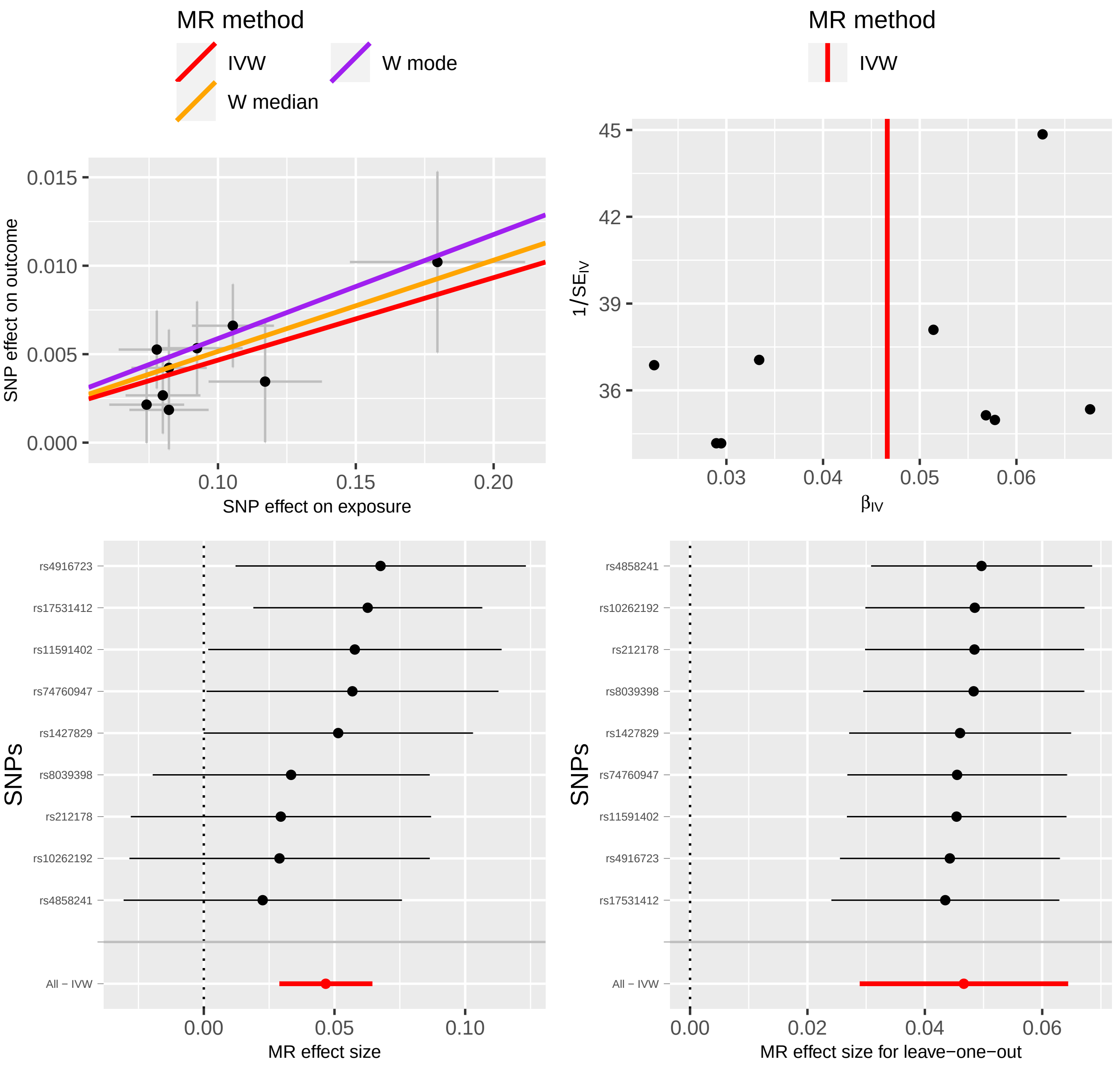

bo) Age first had sexual intercourse → ADHD

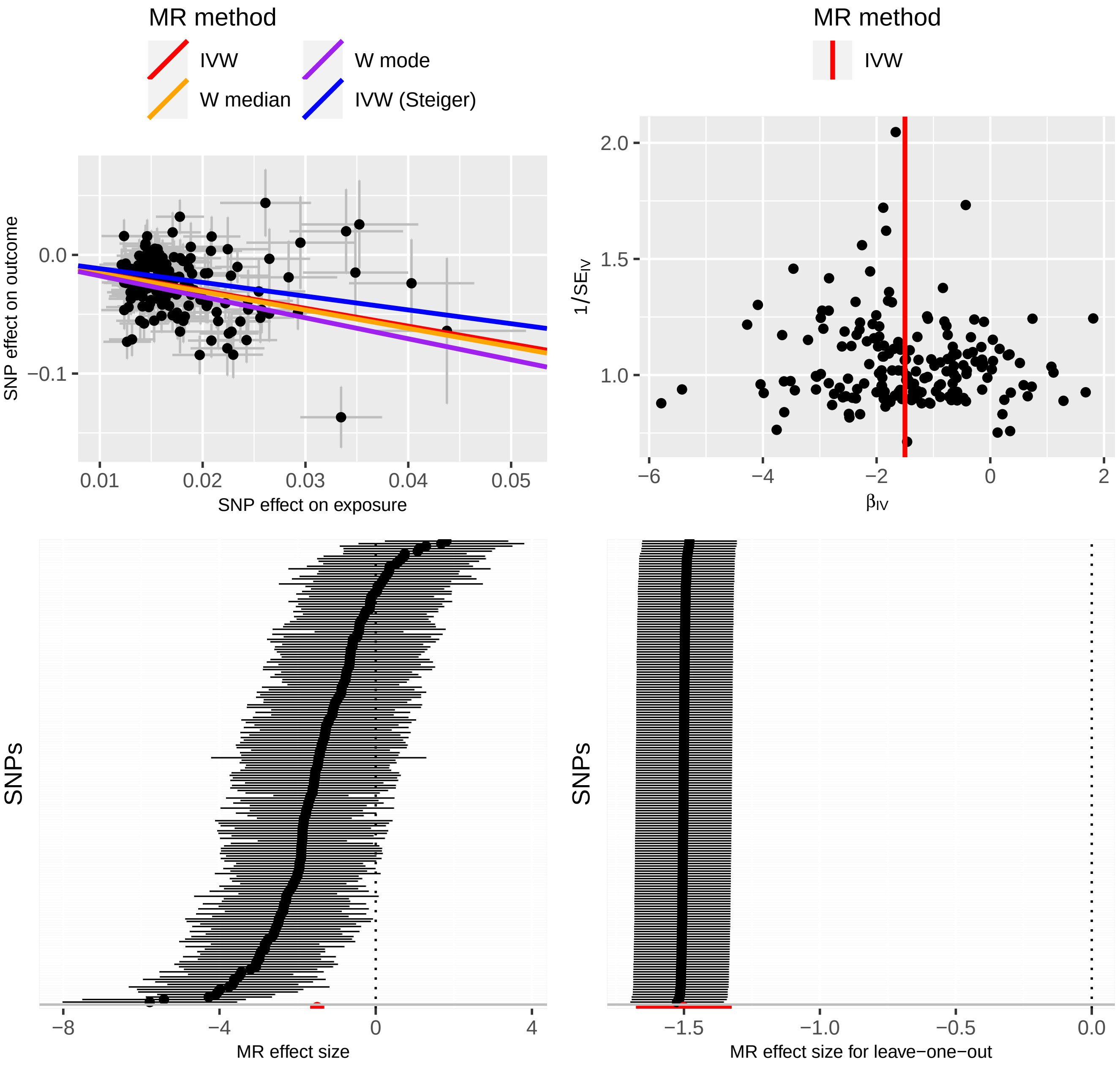

bp) ADHD → Age first had sexual intercourse

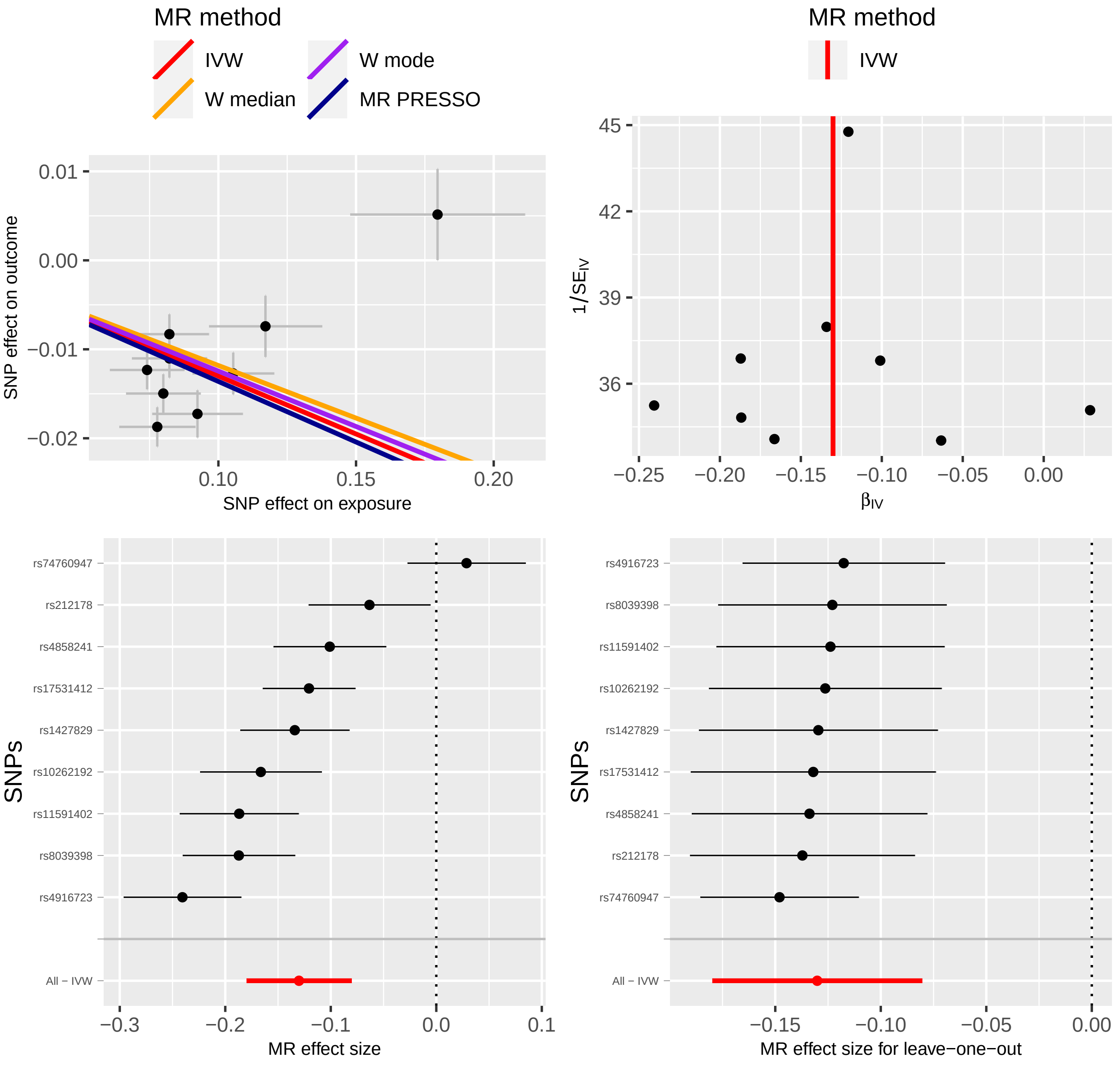

bq) ADHD → Lifetime number of sexual partners

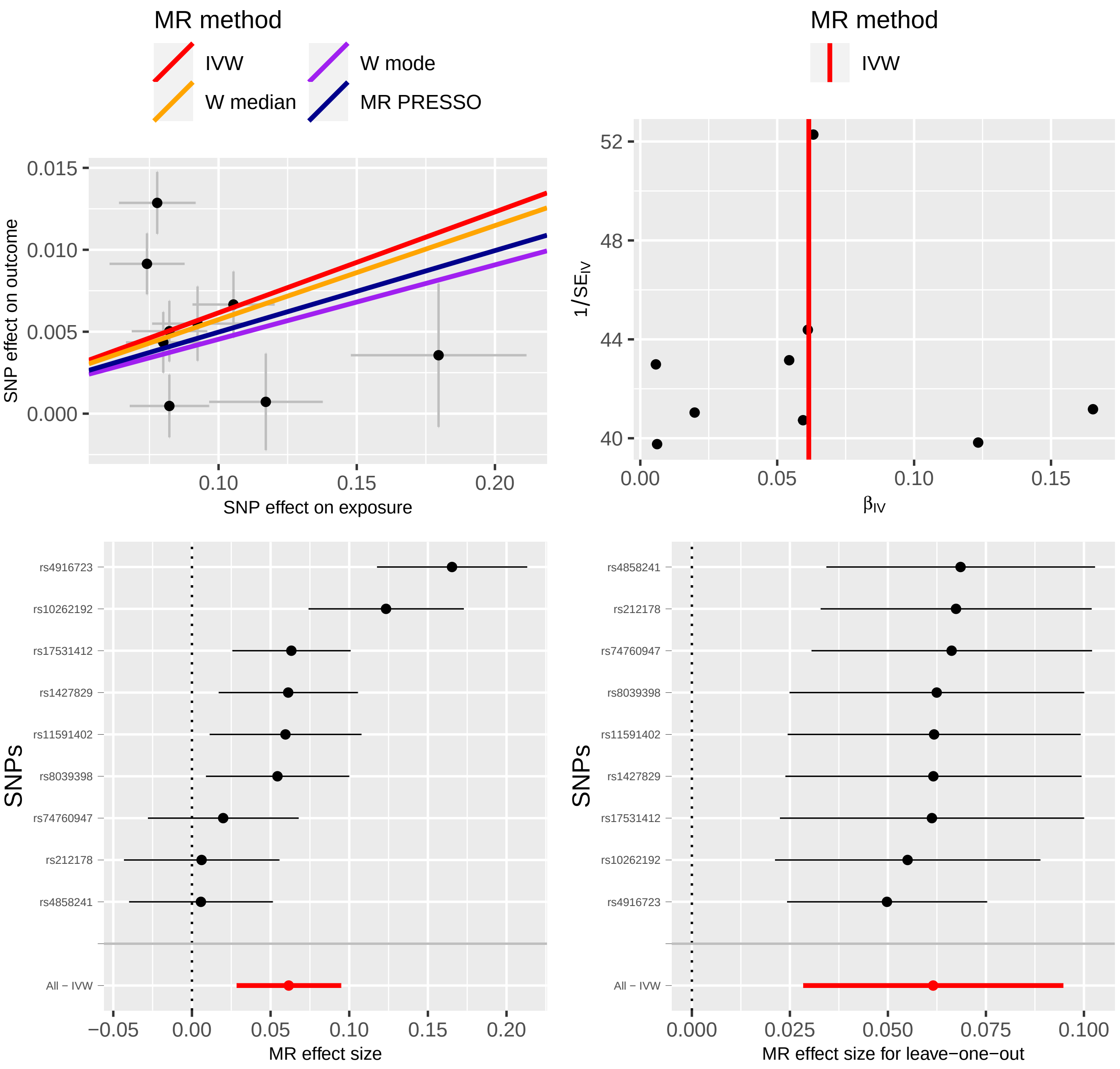

br) Lifetime number of sexual partners → ADHD

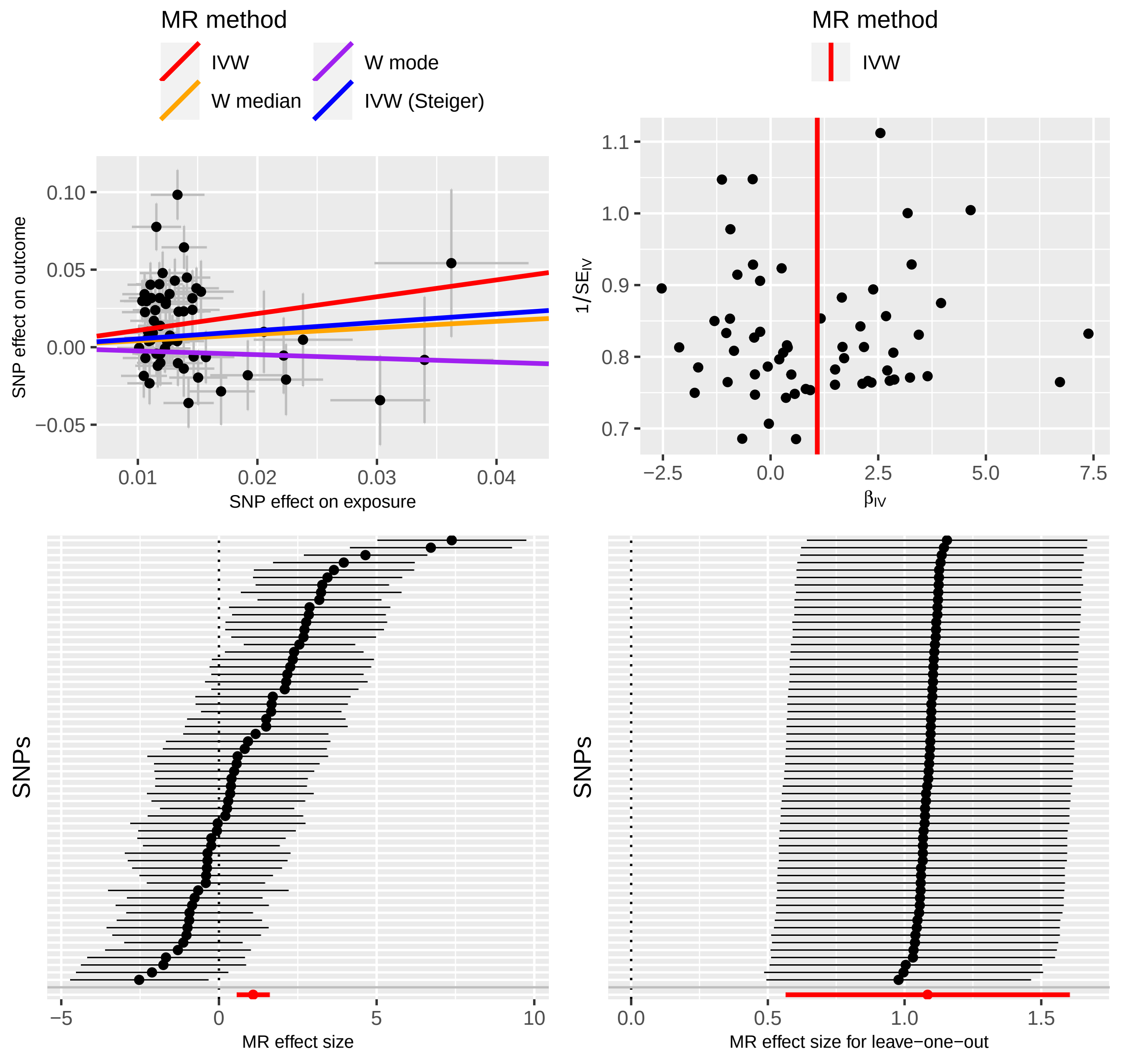

bs) ADHD → Current tobacco smoking

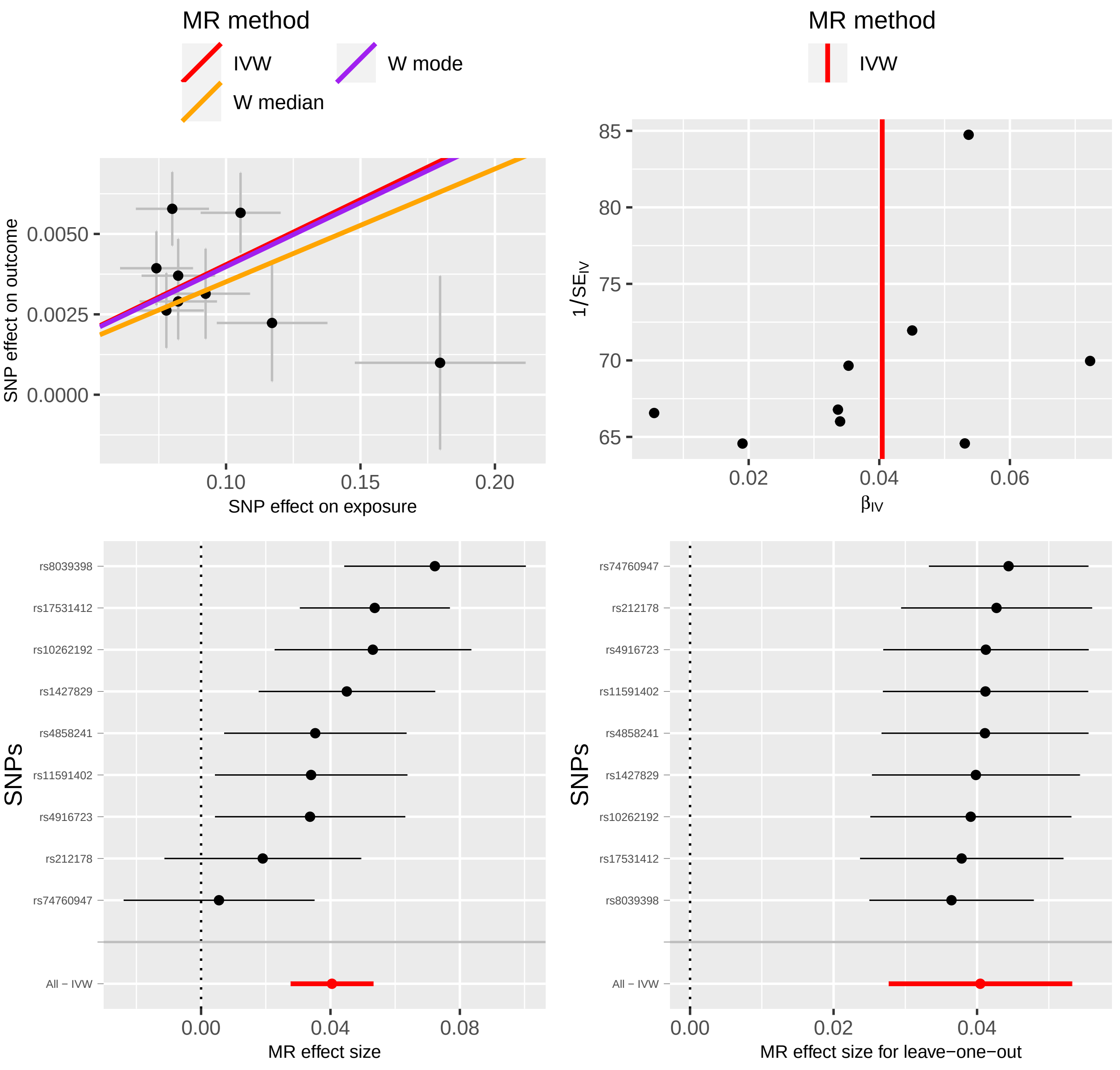

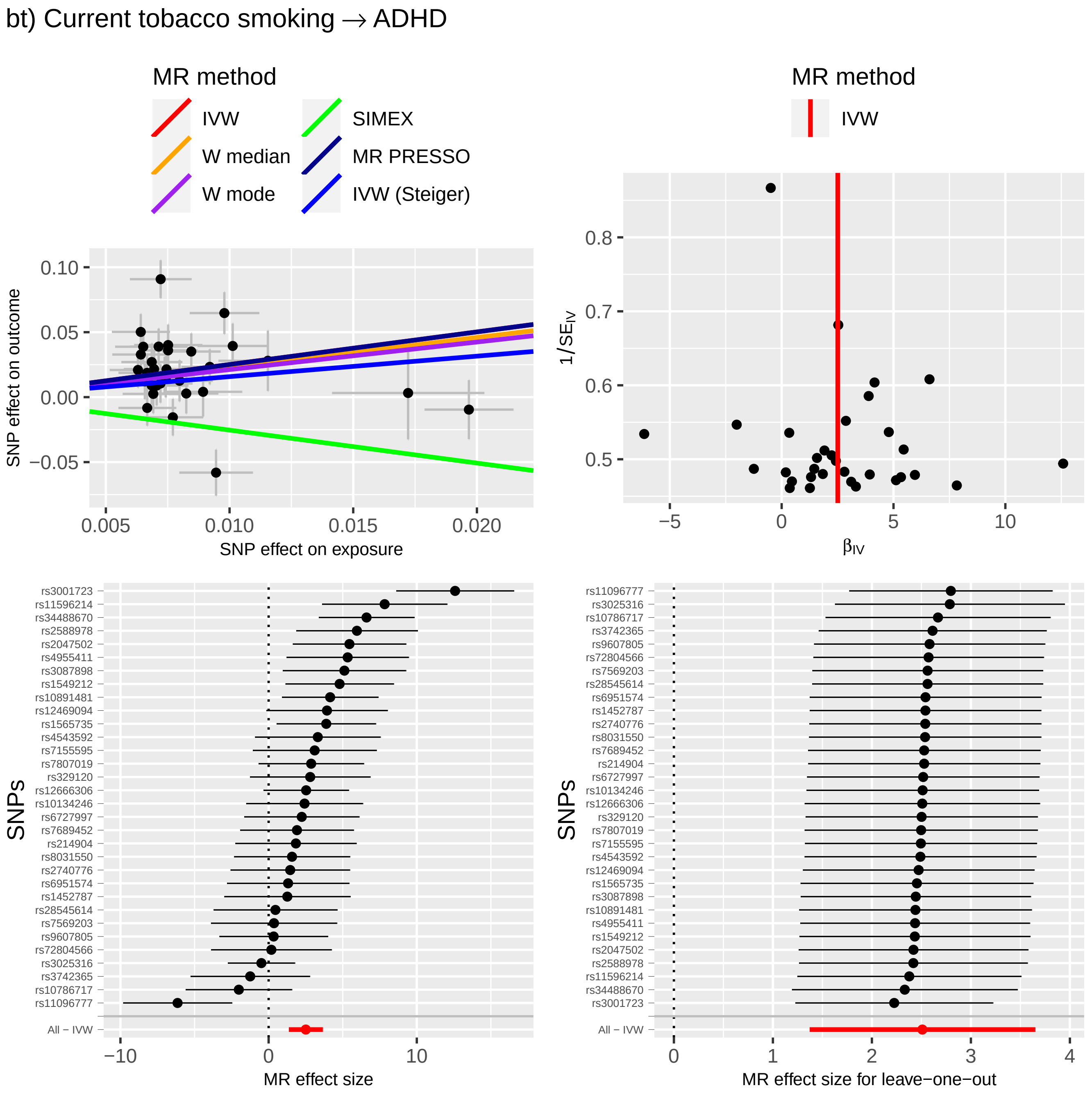

bt) Current tobacco smoking → ADHD
MR method
IVW
W median
W mode
SIMEX
MR PRESSO
IVW (Steiger)
SNP effect on outcome
SNP effect on exposure
0.10
0.05
0.00
−0.05
0.005
0.010
0.015
0.020
MR method
IVW
1/SE_IV
β_IV
0.8
0.7
0.6
0.5
−5
0
5
10
SNPs
rs3001723
rs11596214
rs34488670
rs2588978
rs2047502
rs4955411
rs3087898
rs1549212
rs10891481
rs12469094
rs1565735
rs4543592
rs7155595
rs7807019
rs329120
rs12666306
rs10134246
rs6727997
rs7689452
rs214904
rs8031550
rs2740776
rs6951574
rs1452787
rs28545614
rs7569203
rs9607805
rs72804566
rs3025316
rs3742365
rs10786717
rs11096777
All − IVW
−10
0
10
MR effect size
SNPs
rs11096777
rs3025316
rs10786717
rs3742365
rs9607805
rs72804566
rs7569203
rs28545614
rs6951574
rs1452787
rs2740776
rs8031550
rs7689452
rs214904
rs6727997
rs10134246
rs12666306
rs329120
rs7807019
rs7155595
rs4543592
rs12469094
rs1565735
rs3087898
rs10891481
rs4955411
rs1549212
rs2047502
rs2588978
rs11596214
rs34488670
rs3001723
All − IVW
0
1
2
3
4
MR effect size for leave−one−out

bu) Ever smoked → ADHD

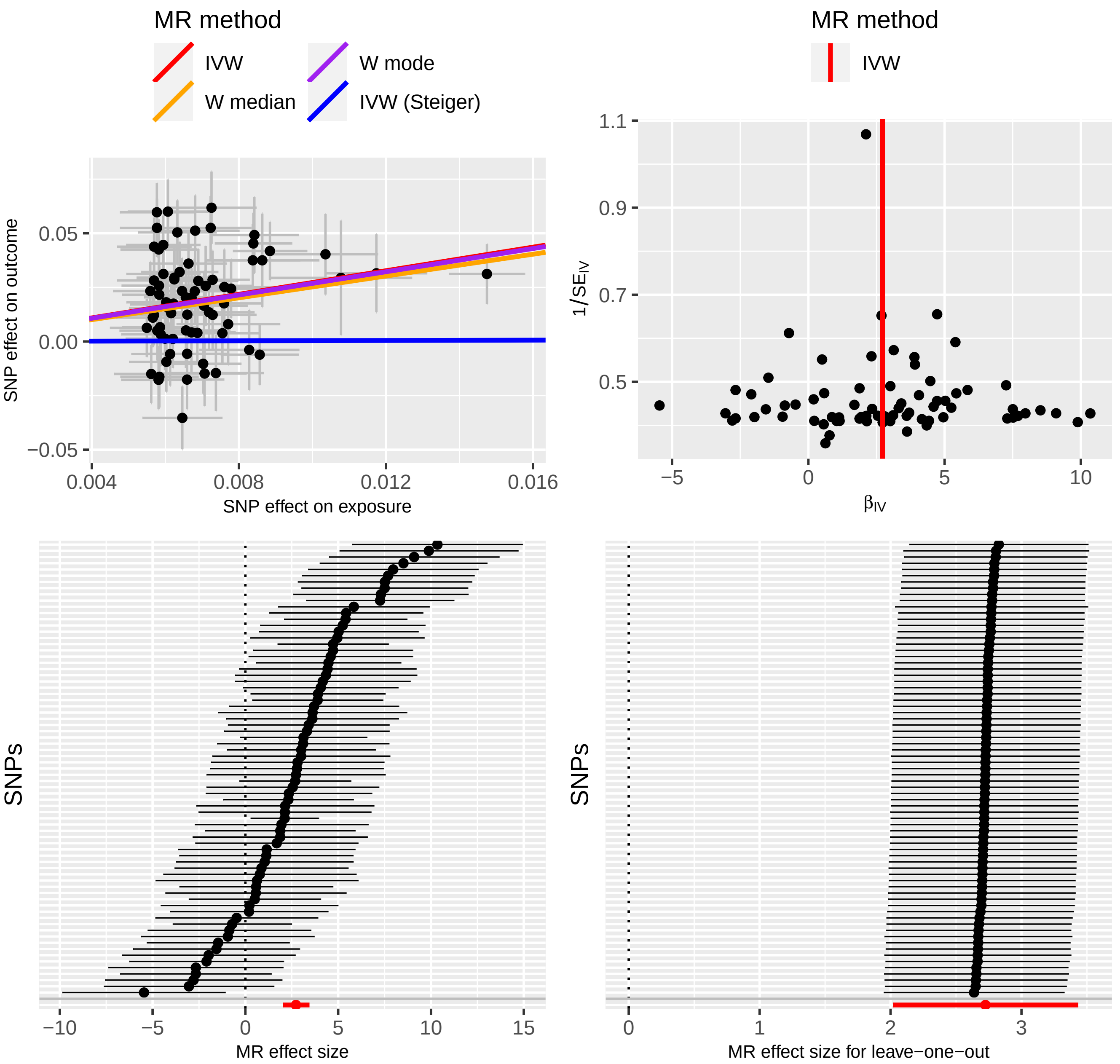

bv) ADHD → Ever smoked

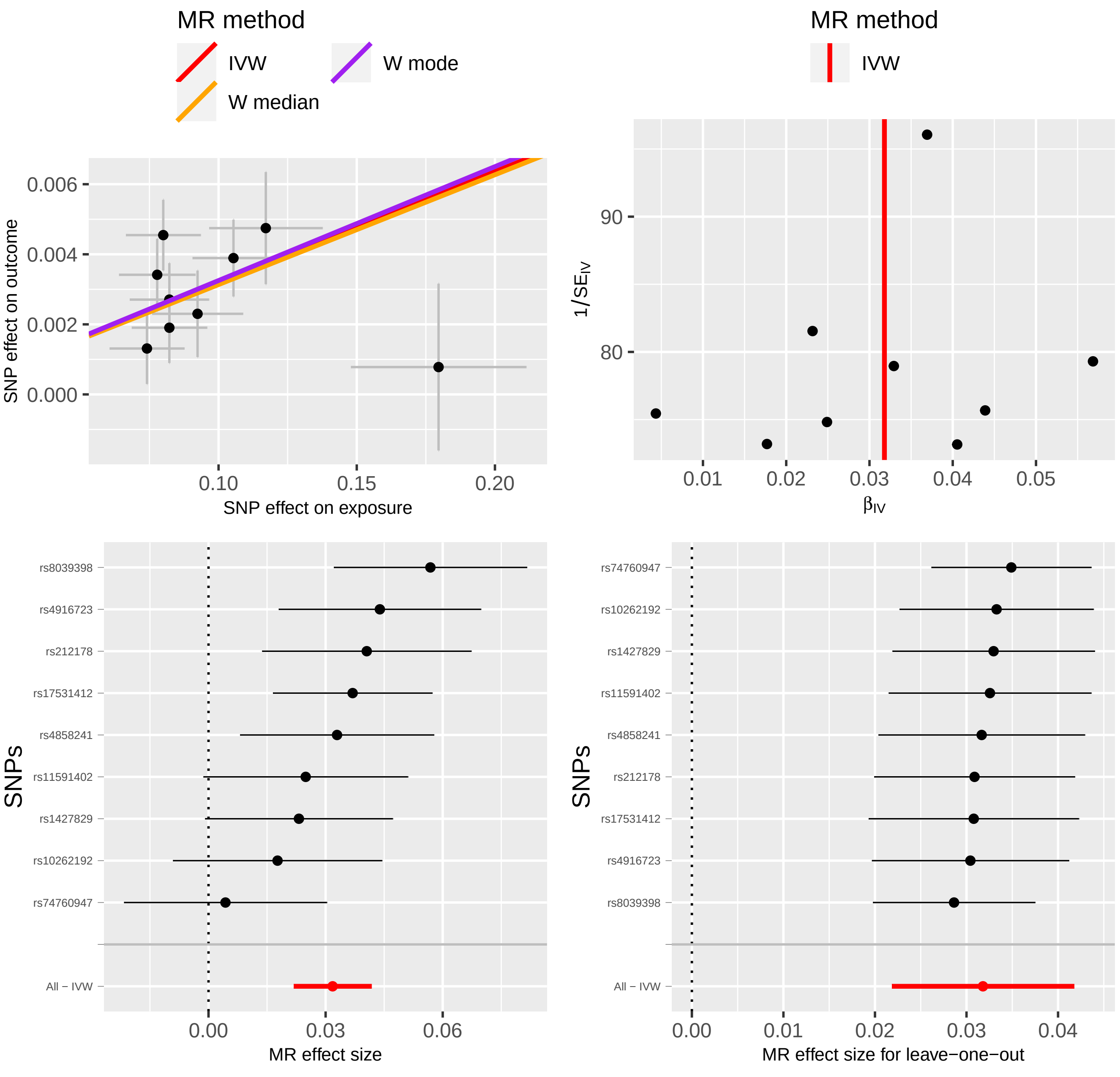

bw) ADHD → Exposure to tobacco smoke outside home

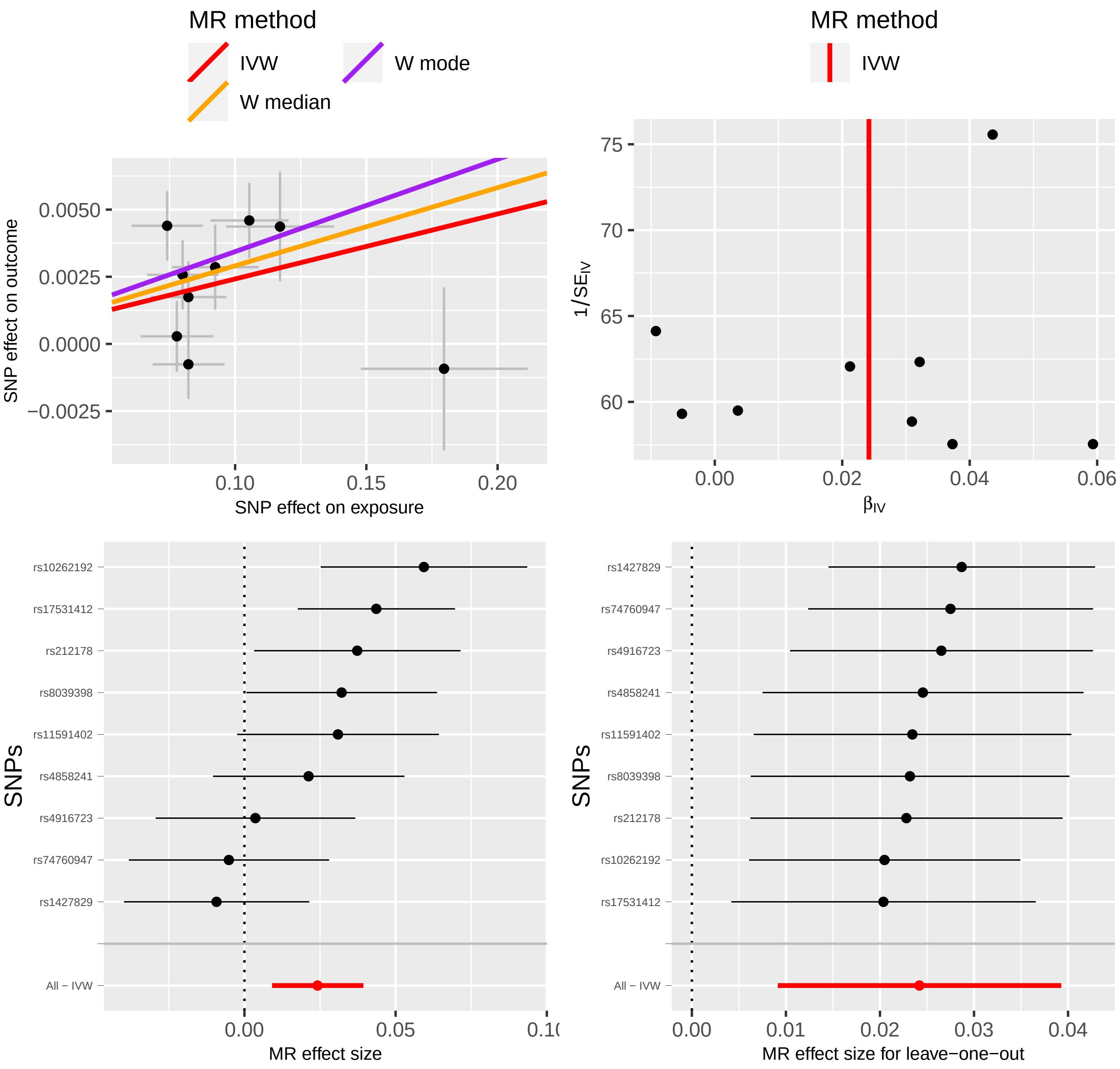

bx) ADHD → Light smokers at least 100 smokes in lifetime

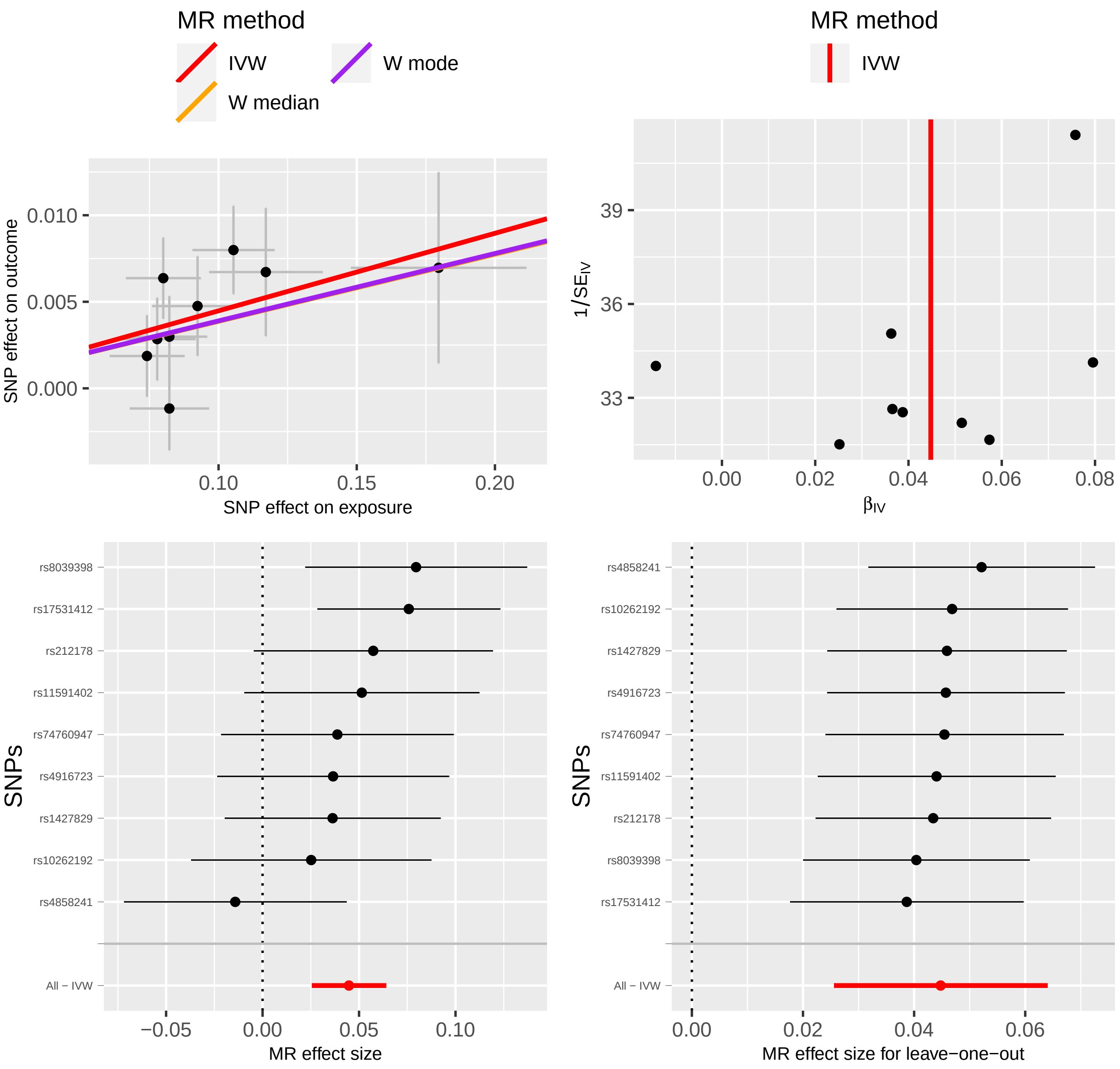

by) ADHD → Pack years adult smoking as proportion of life span exposed to smoking

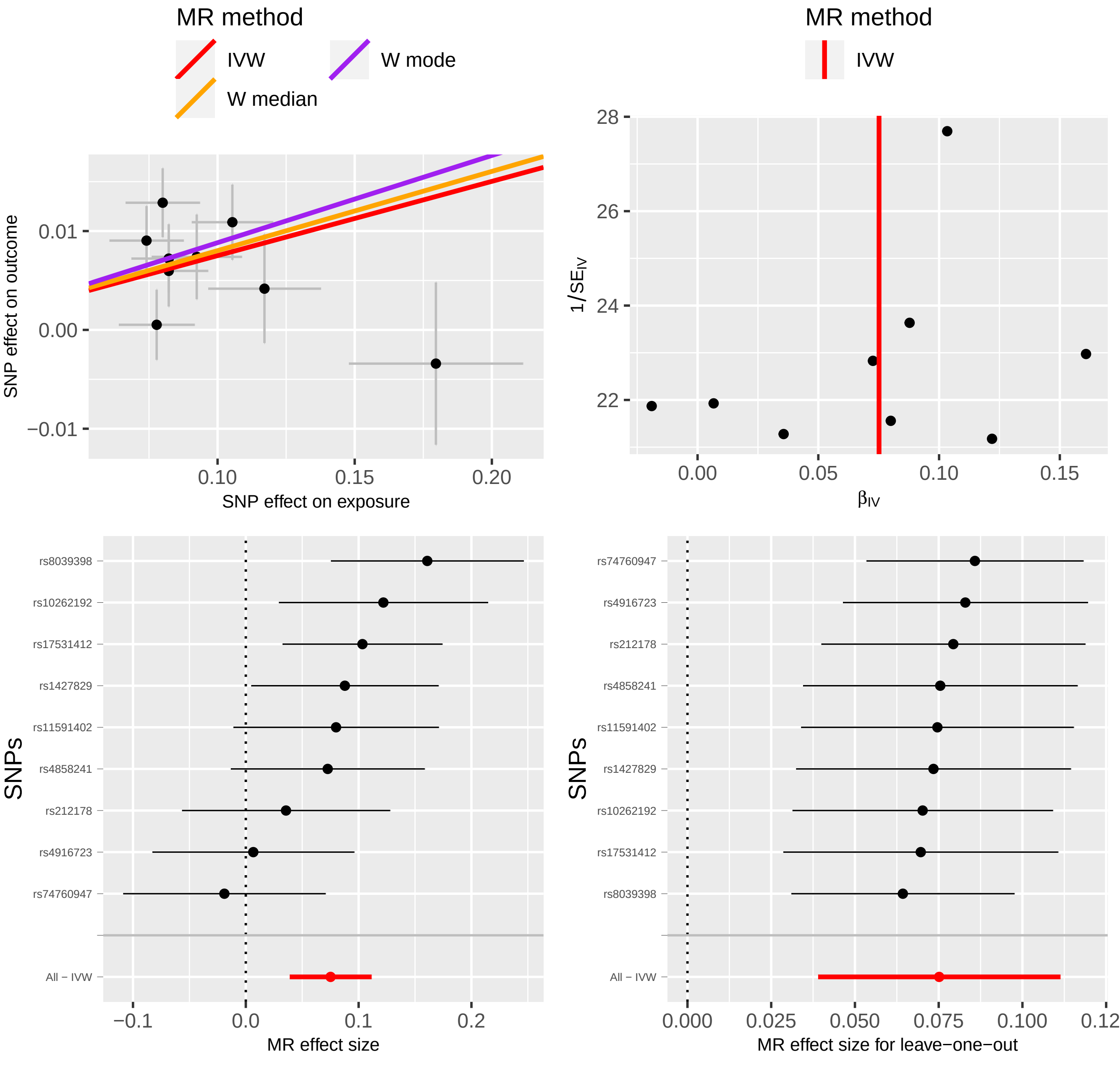

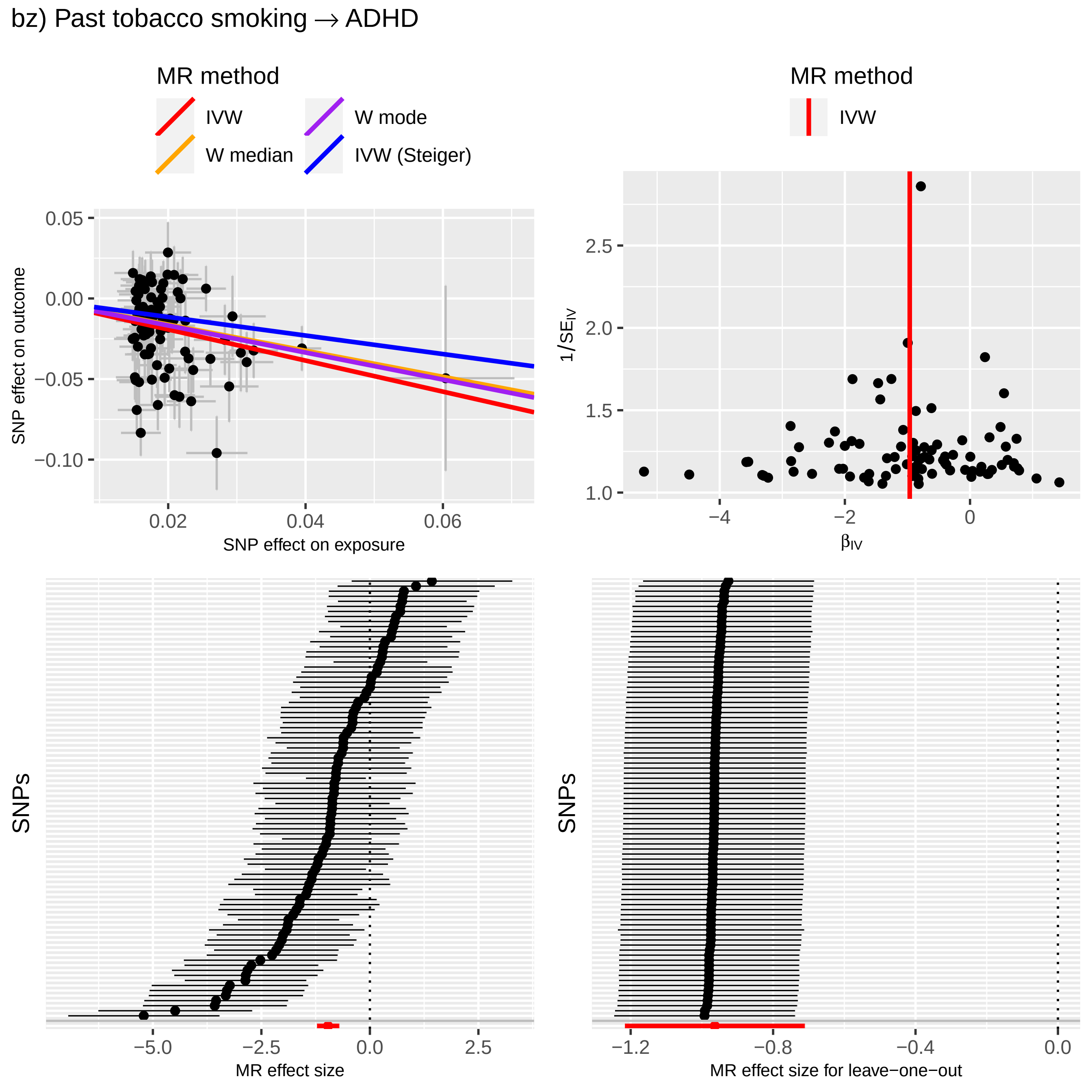

bz) Past tobacco smoking → ADHD
MR method
IVW
W median
W mode
IVW (Steiger)
SNP effect on outcome
SNP effect on exposure
0.05
0.00
−0.05
−0.10
0.02
0.04
0.06
MR method
IVW
1/SE_IV
β_IV
2.5
2.0
1.5
1.0
−4
−2
0
SNPs
MR effect size
−5.0
−2.5
0.0
2.5
SNPs
MR effect size for leave−one−out
−1.2
−0.8
−0.4
0.0

ca) ADHD → Past tobacco smoking

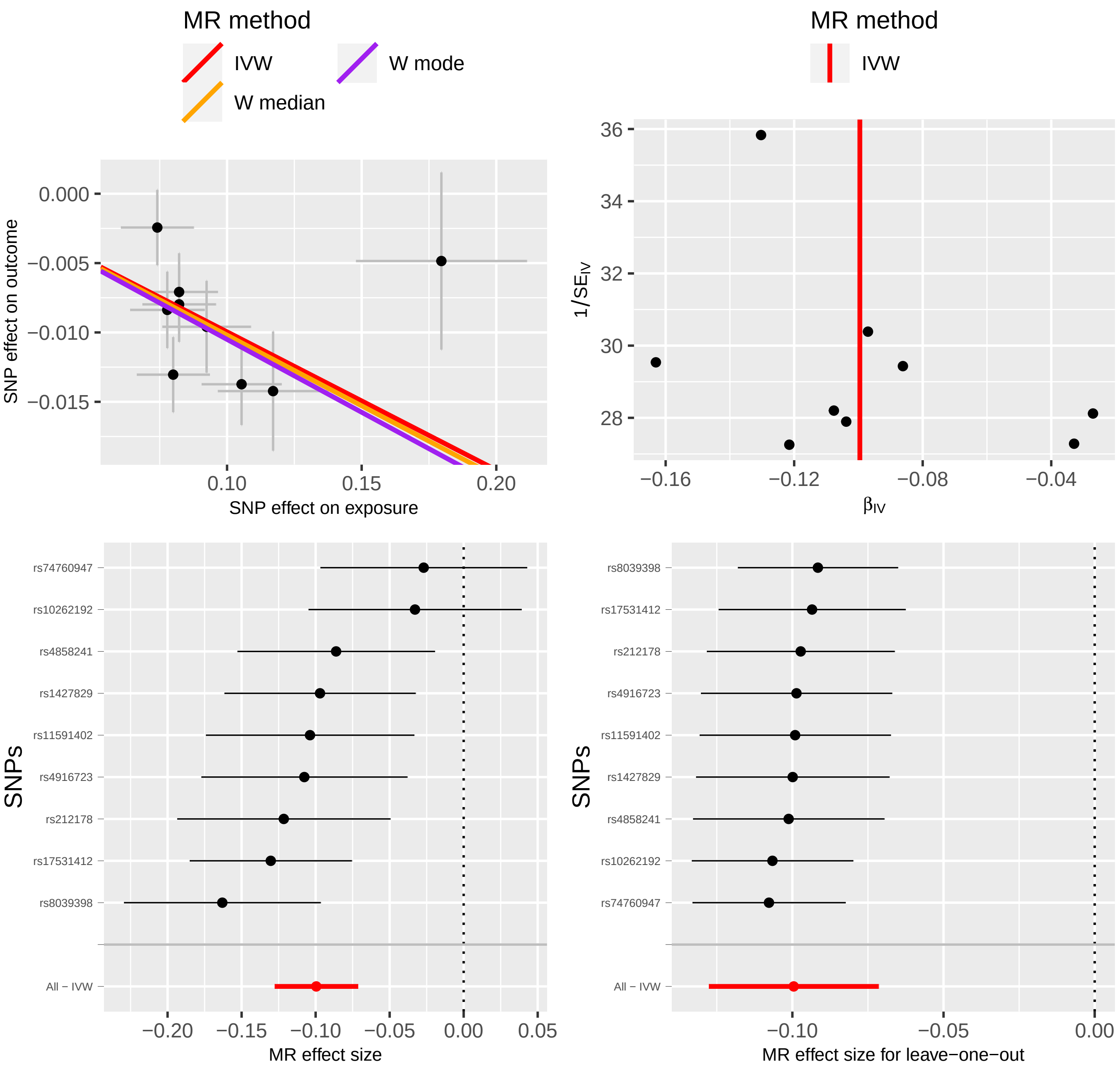

cb) ADHD → Average total household income before tax

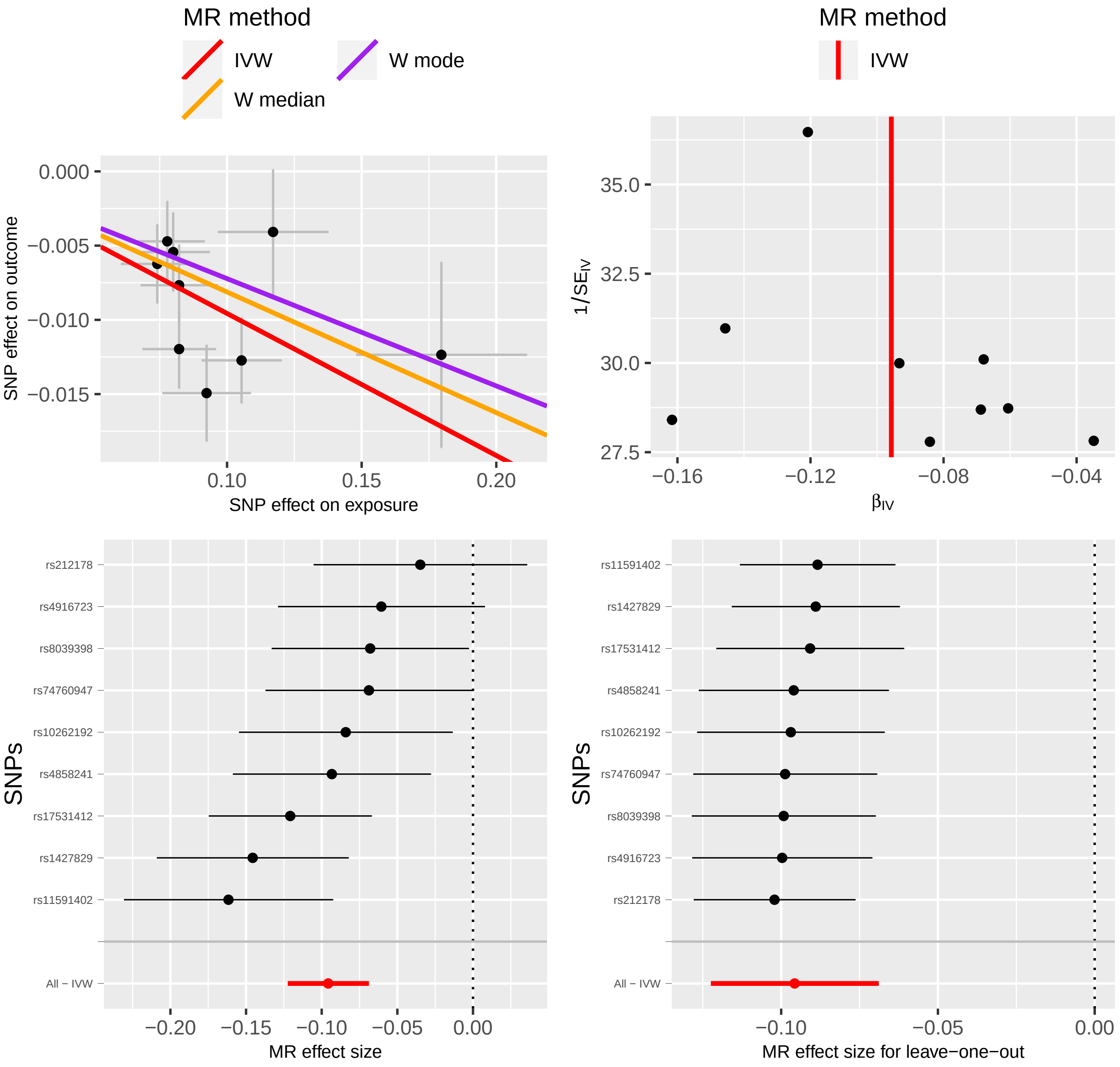

cc) Average total household income before tax → ADHD

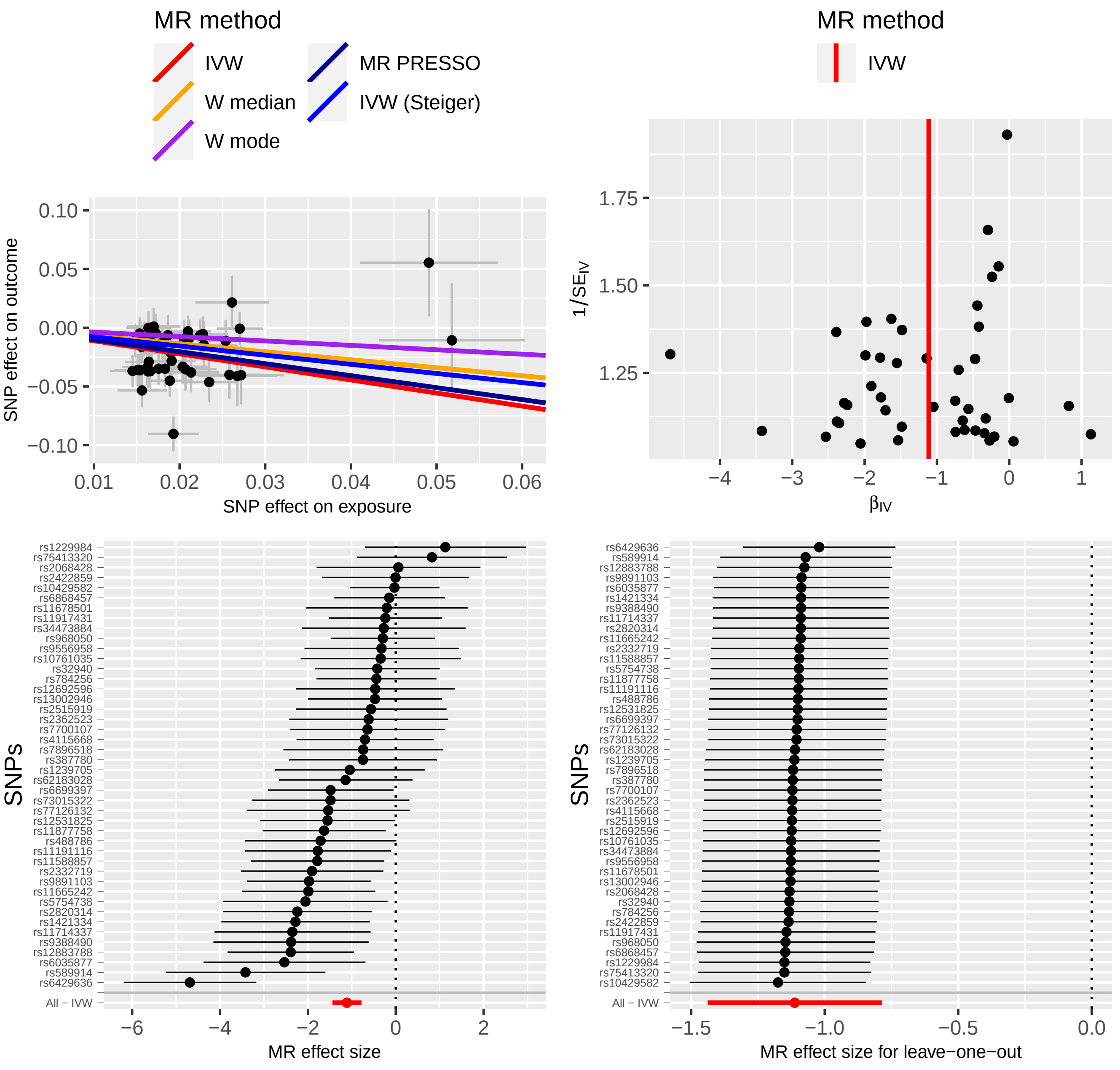

cd) Number of full brothers → ADHD

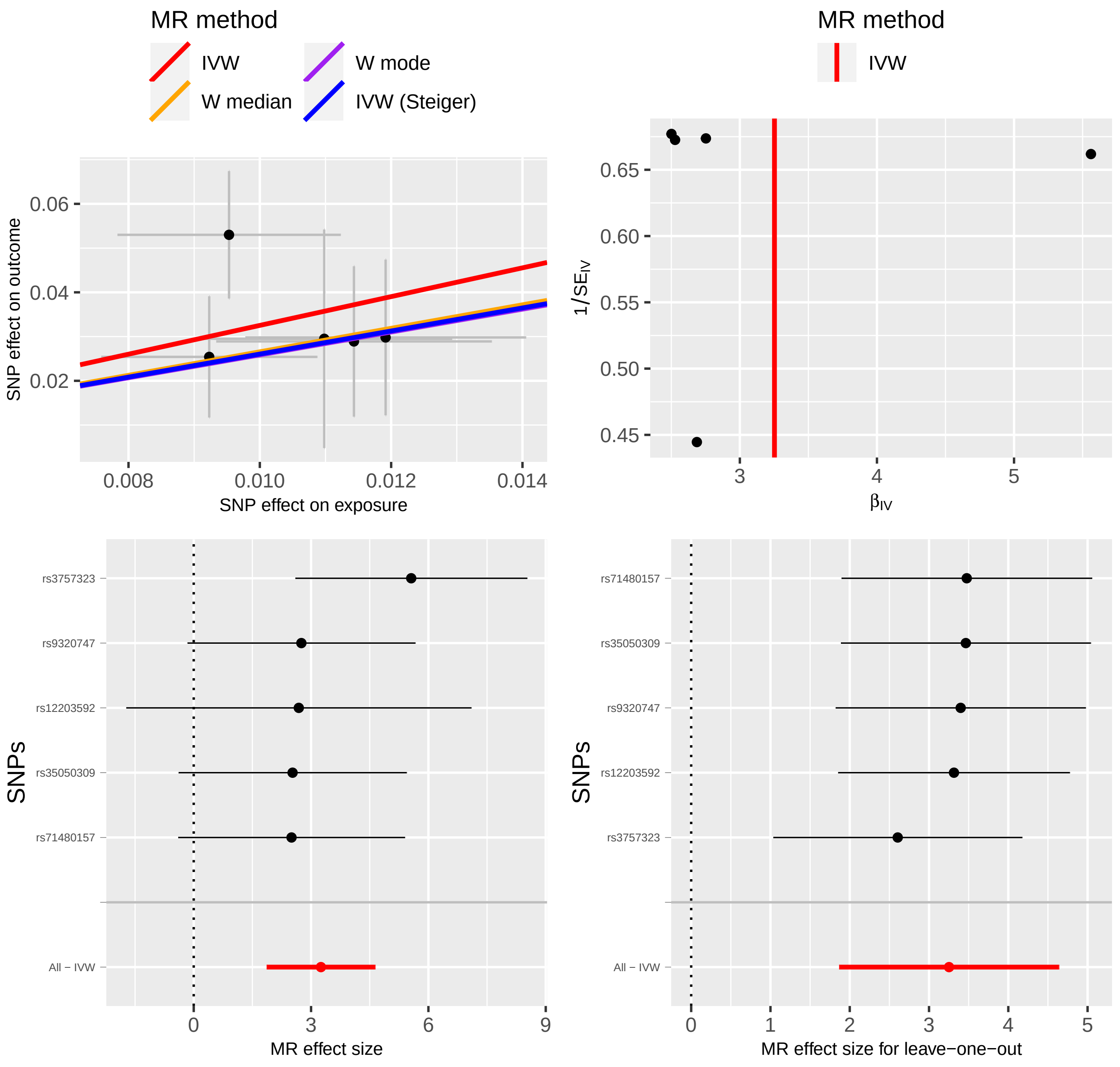

ce) ADHD → Number of full brothers

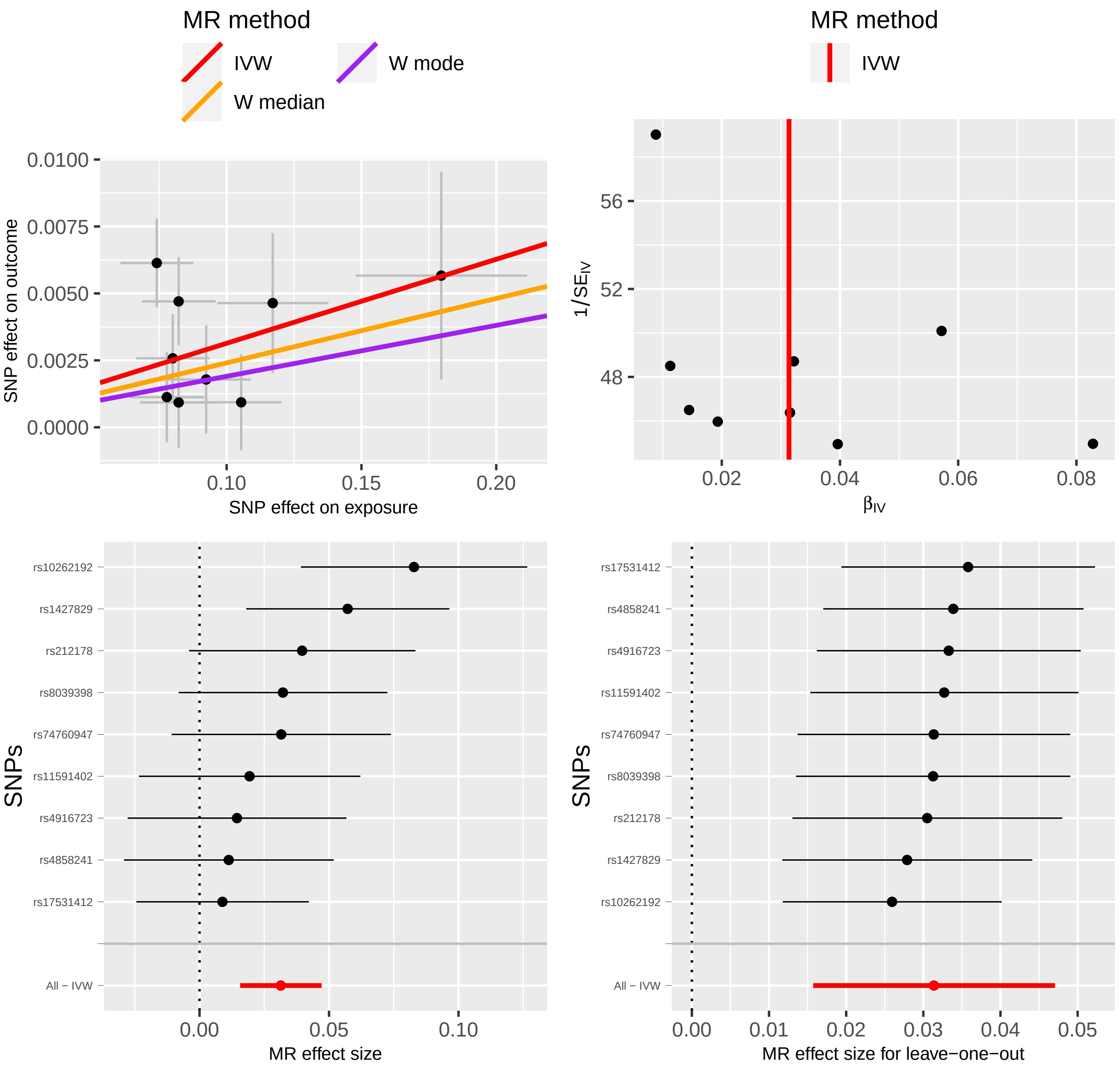

cf) ADHD → Number of full sisters

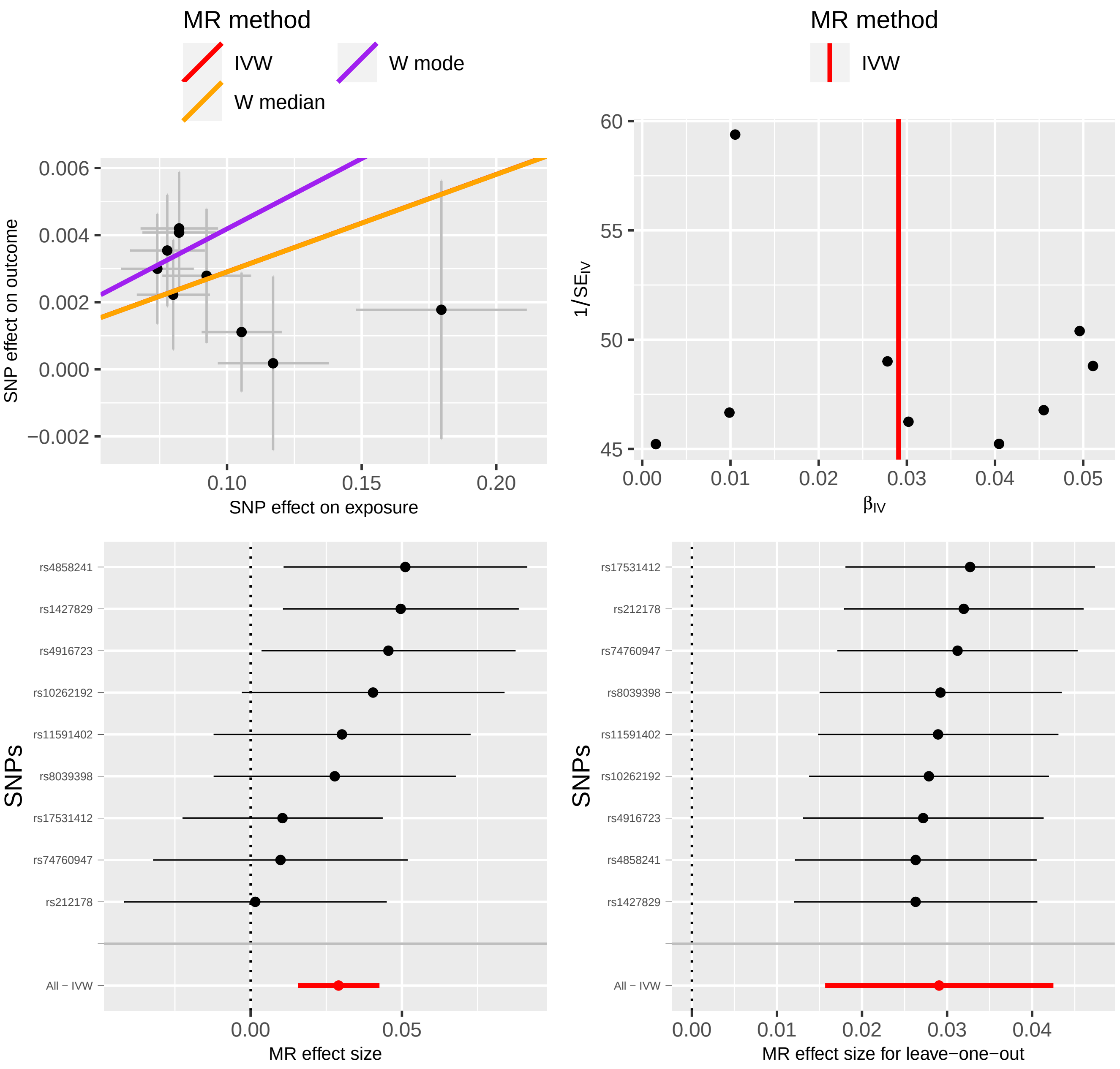

cg) ADHD → Number of vehicles in household

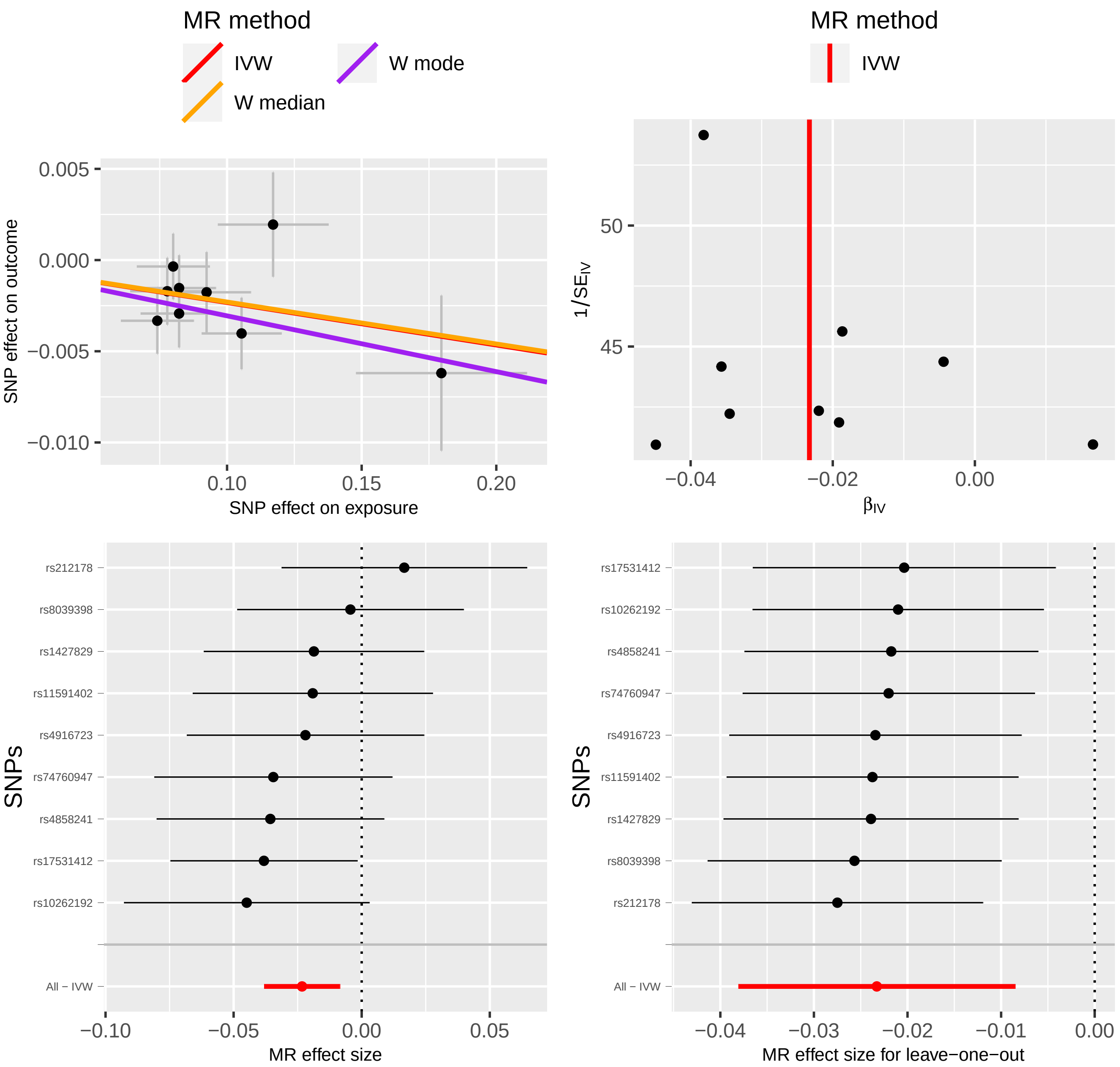

ch) ADHD → Townsend deprivation index at recruitment

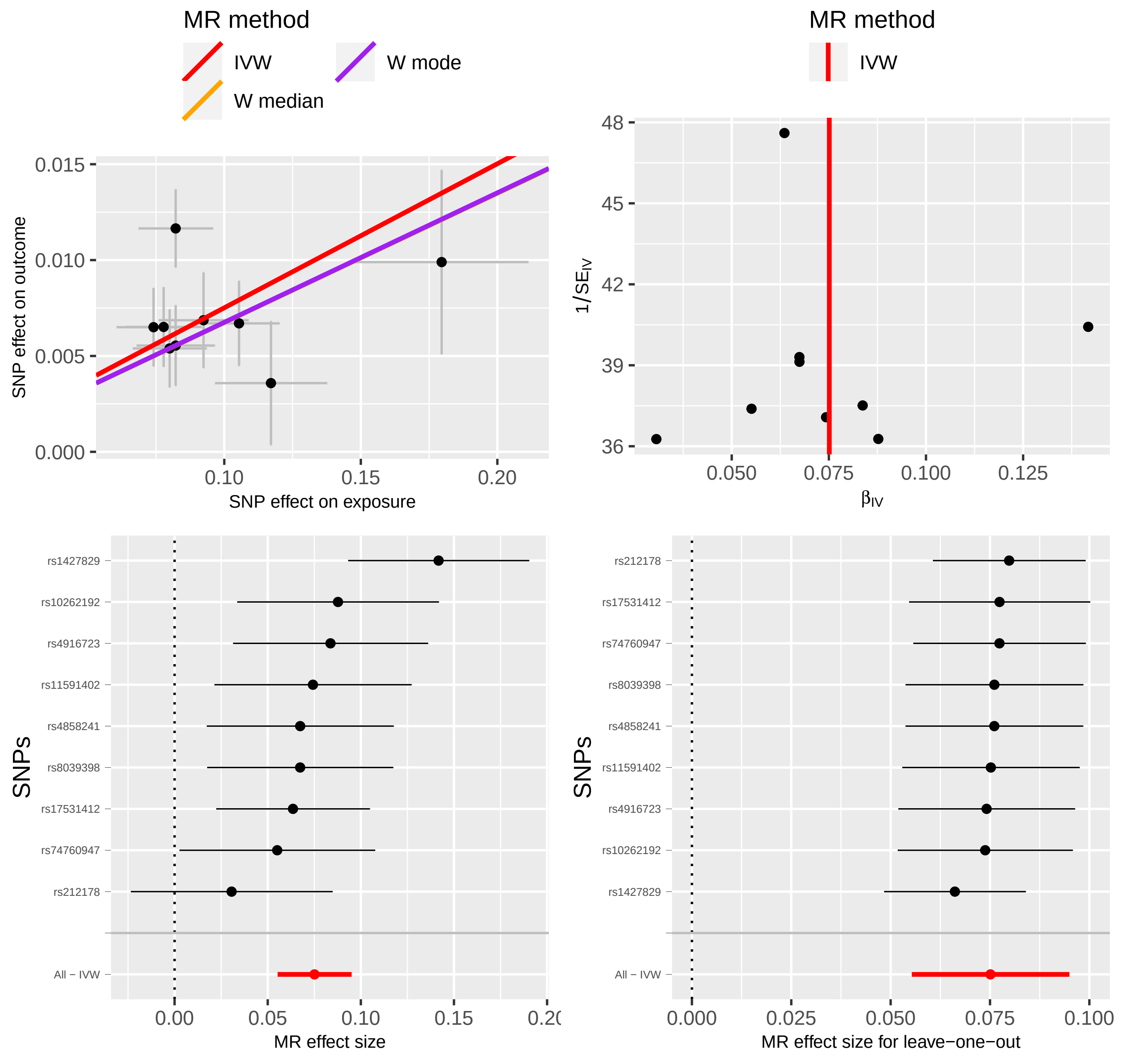

ci) Townsend deprivation index at recruitment → ADHD

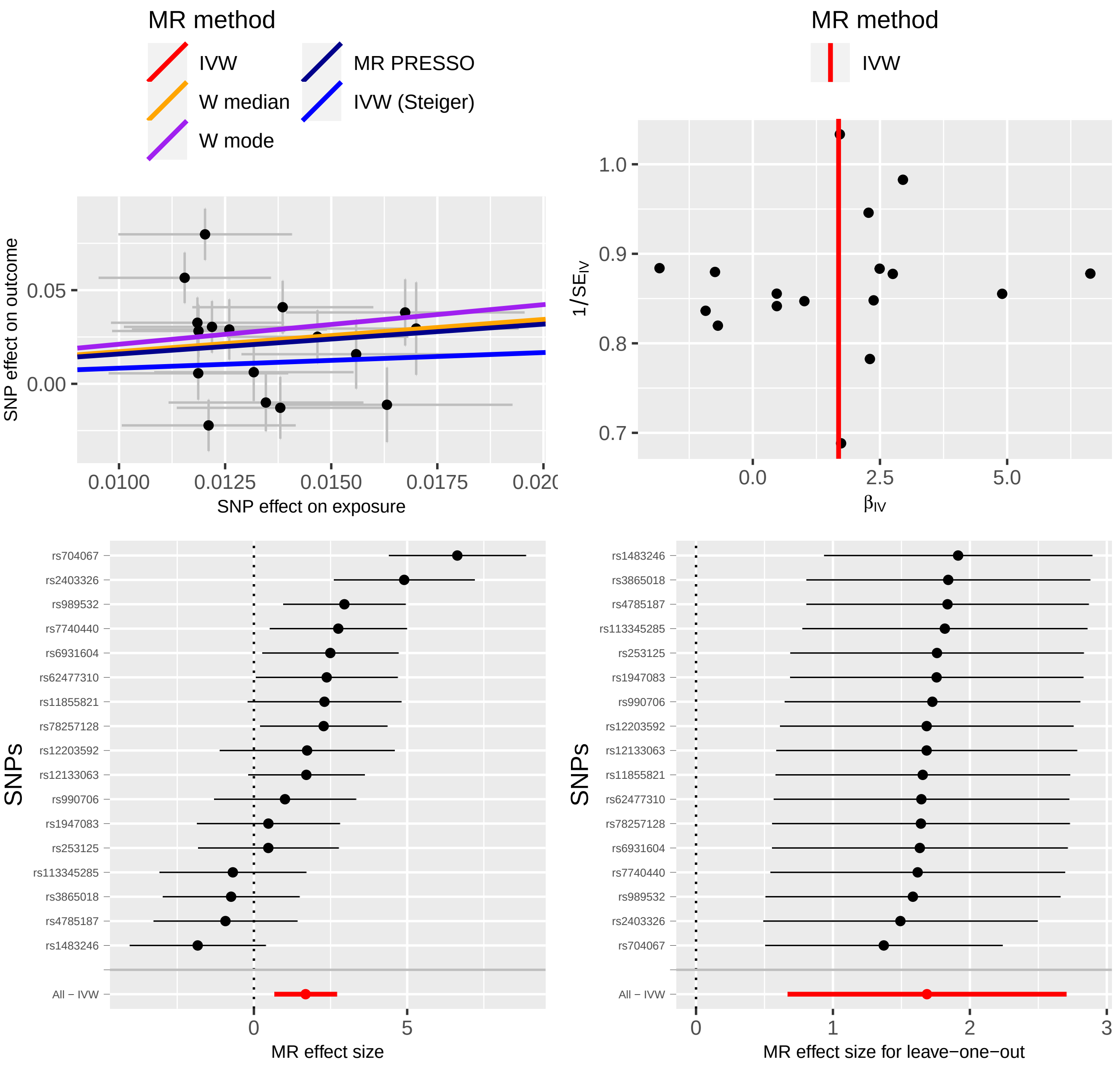

cj) Time spend outdoors in summer → ADHD

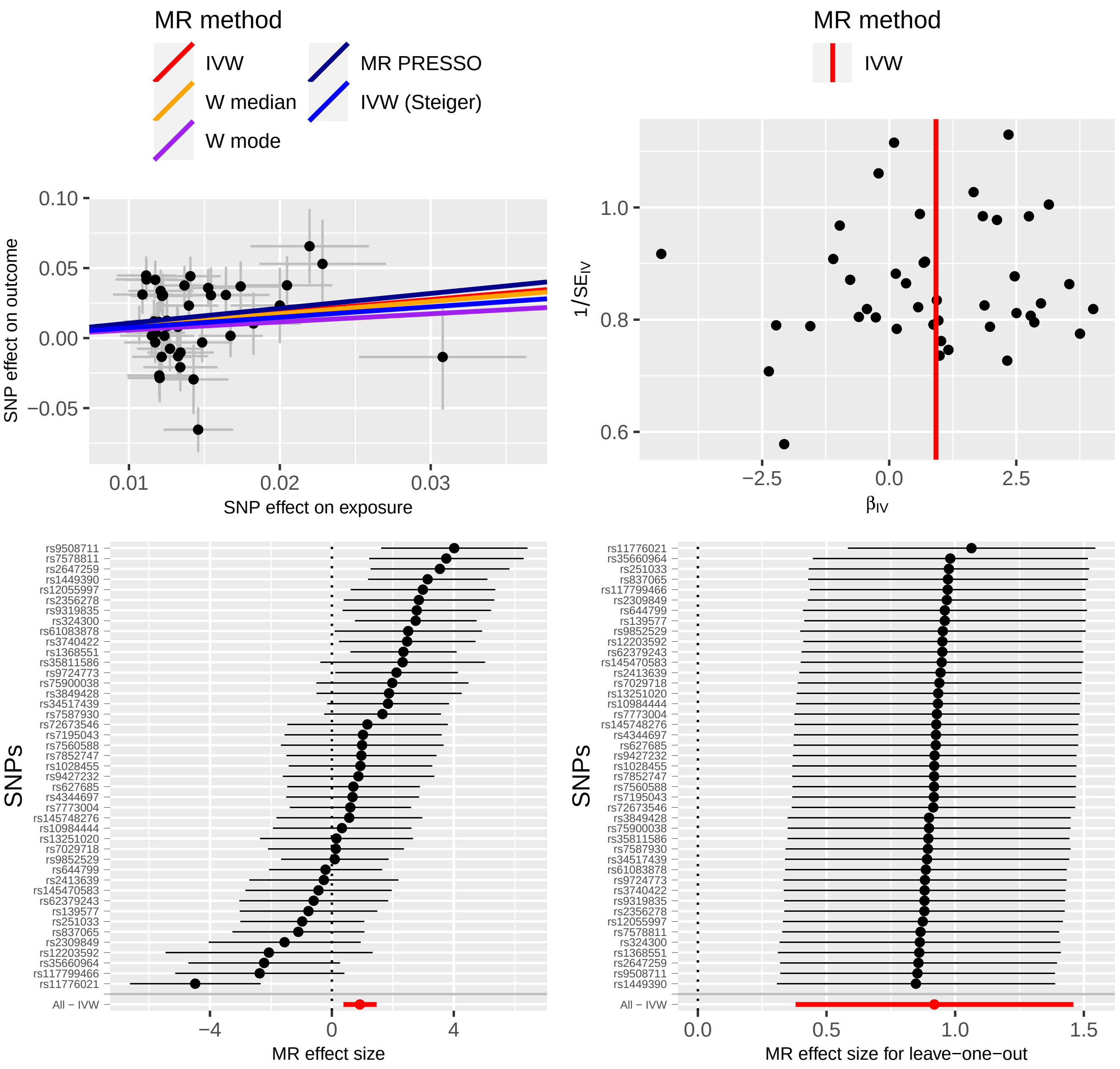

ck) ADHD → Time spend outdoors in summer

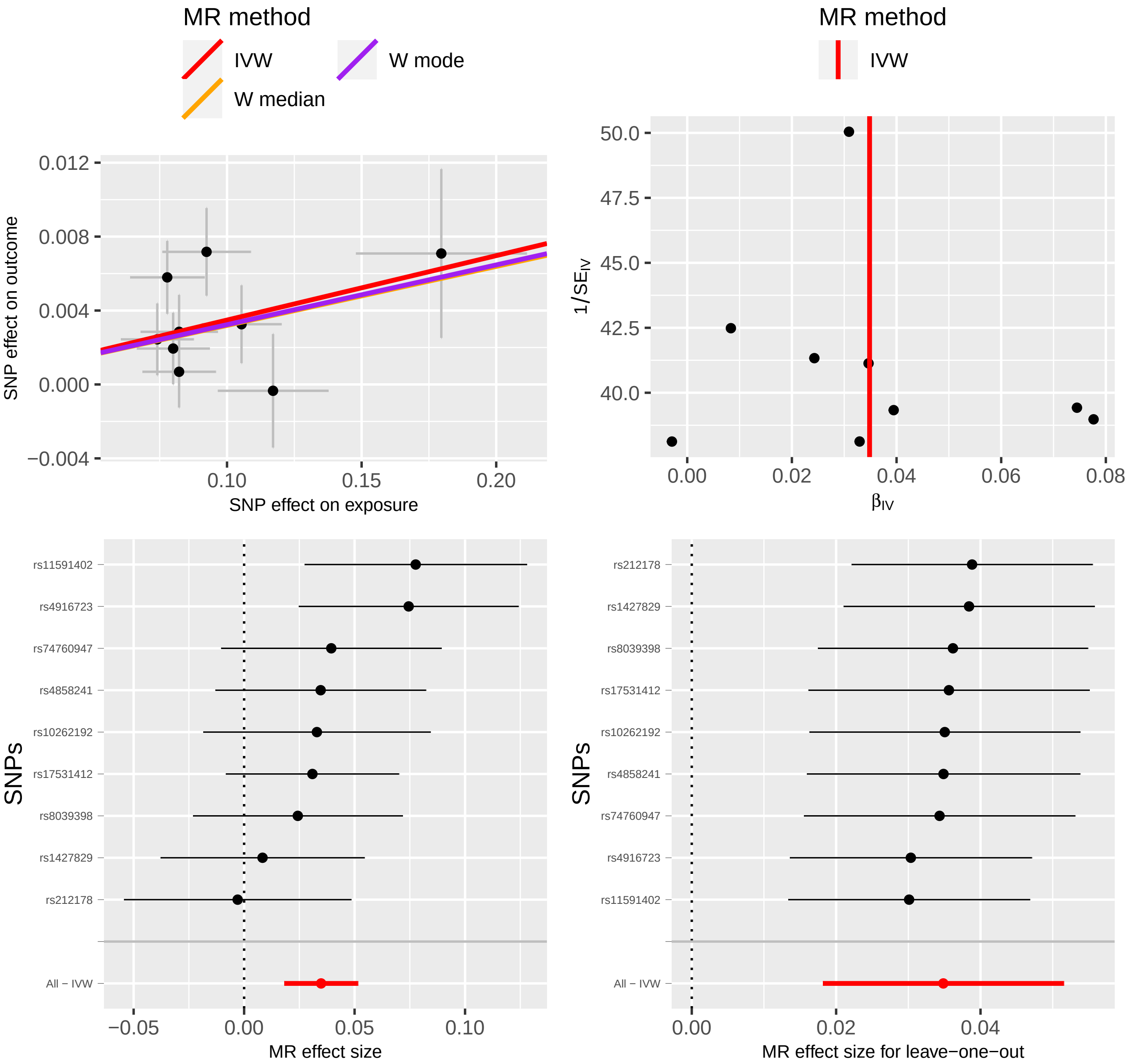

cl) Time spent outdoors in winter → ADHD

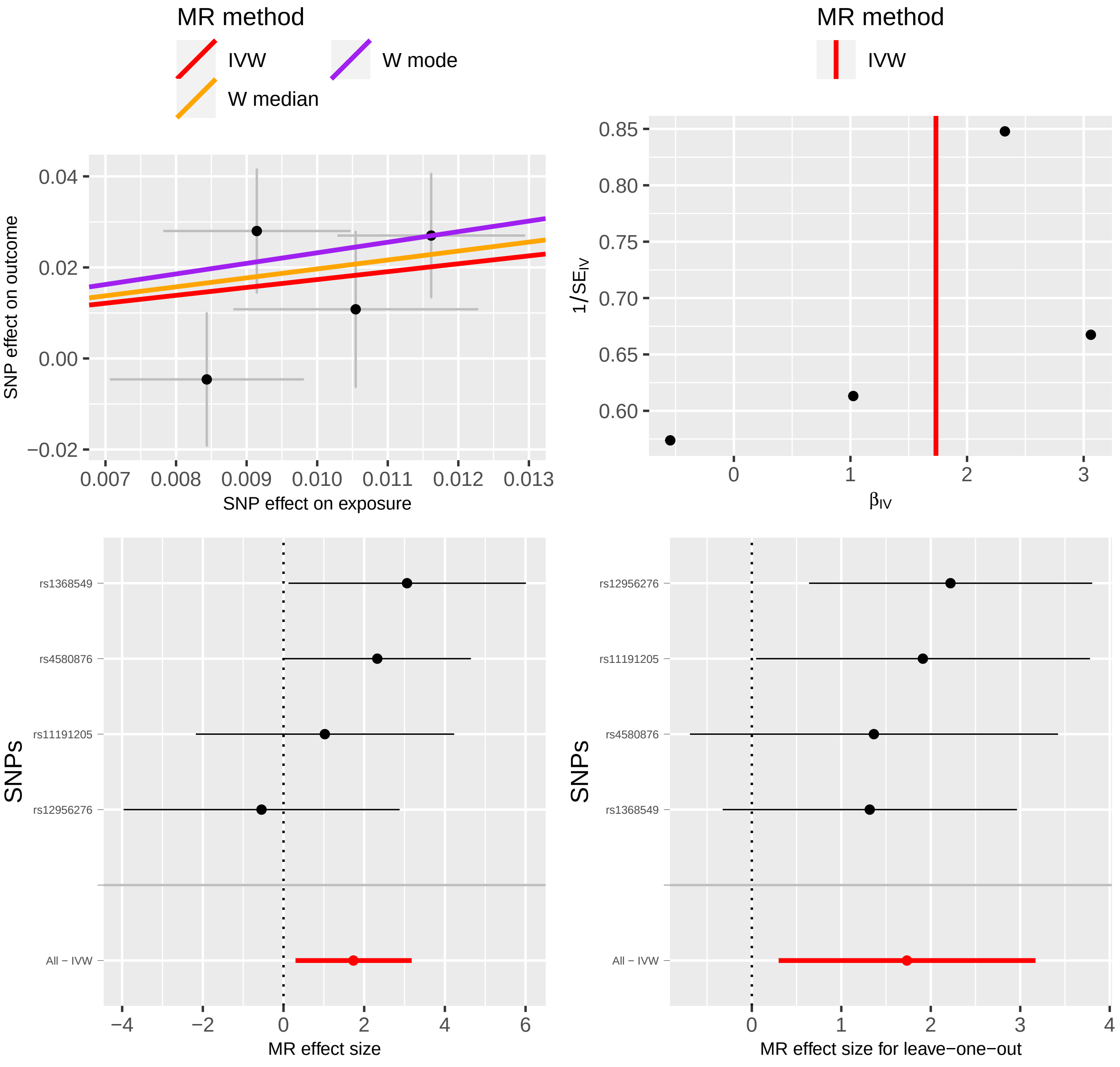

cm) ADHD → Time spent outdoors in winter

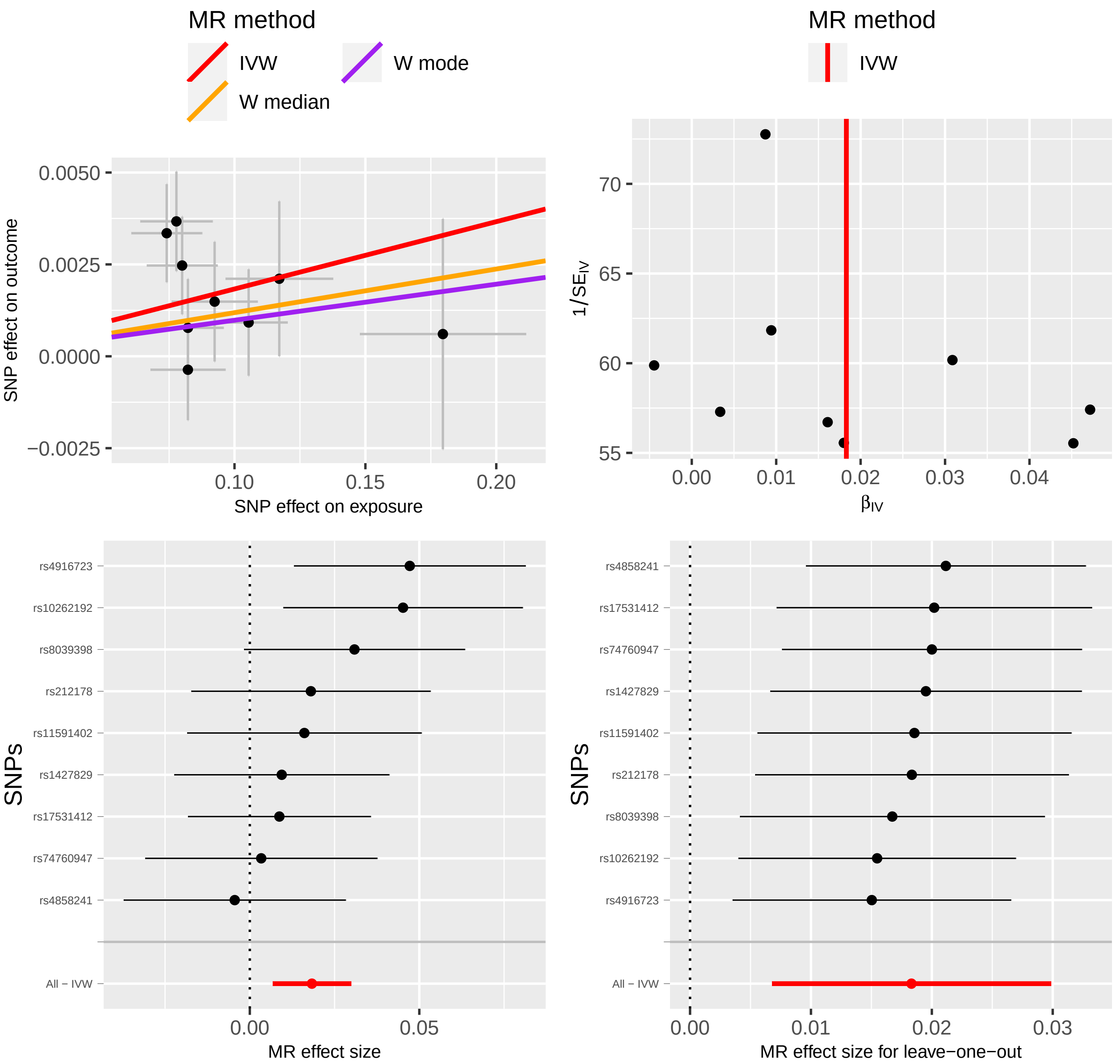

cn) Length of mobile phone use → ADHD

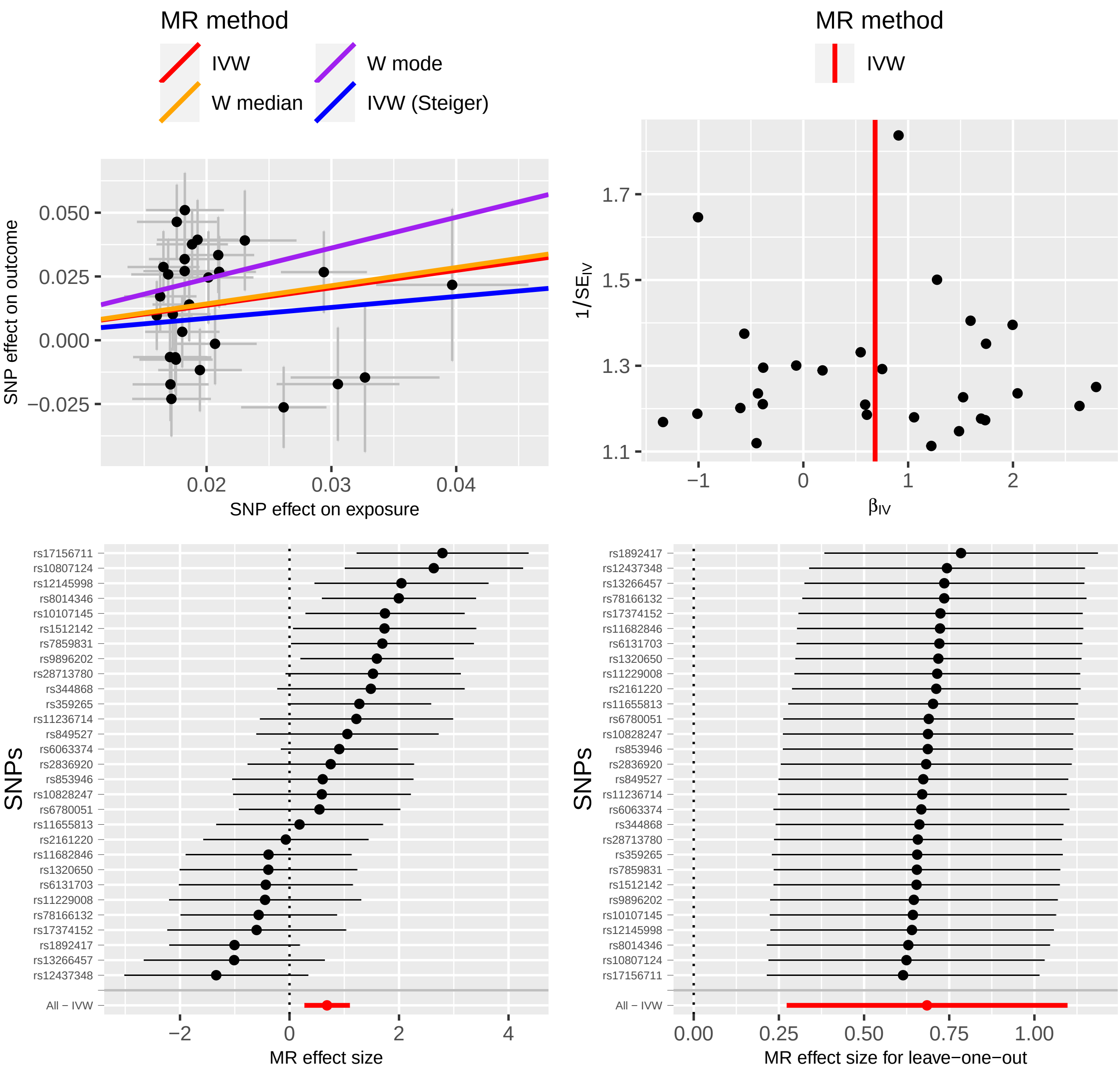

co) ADHD → Plays computer games

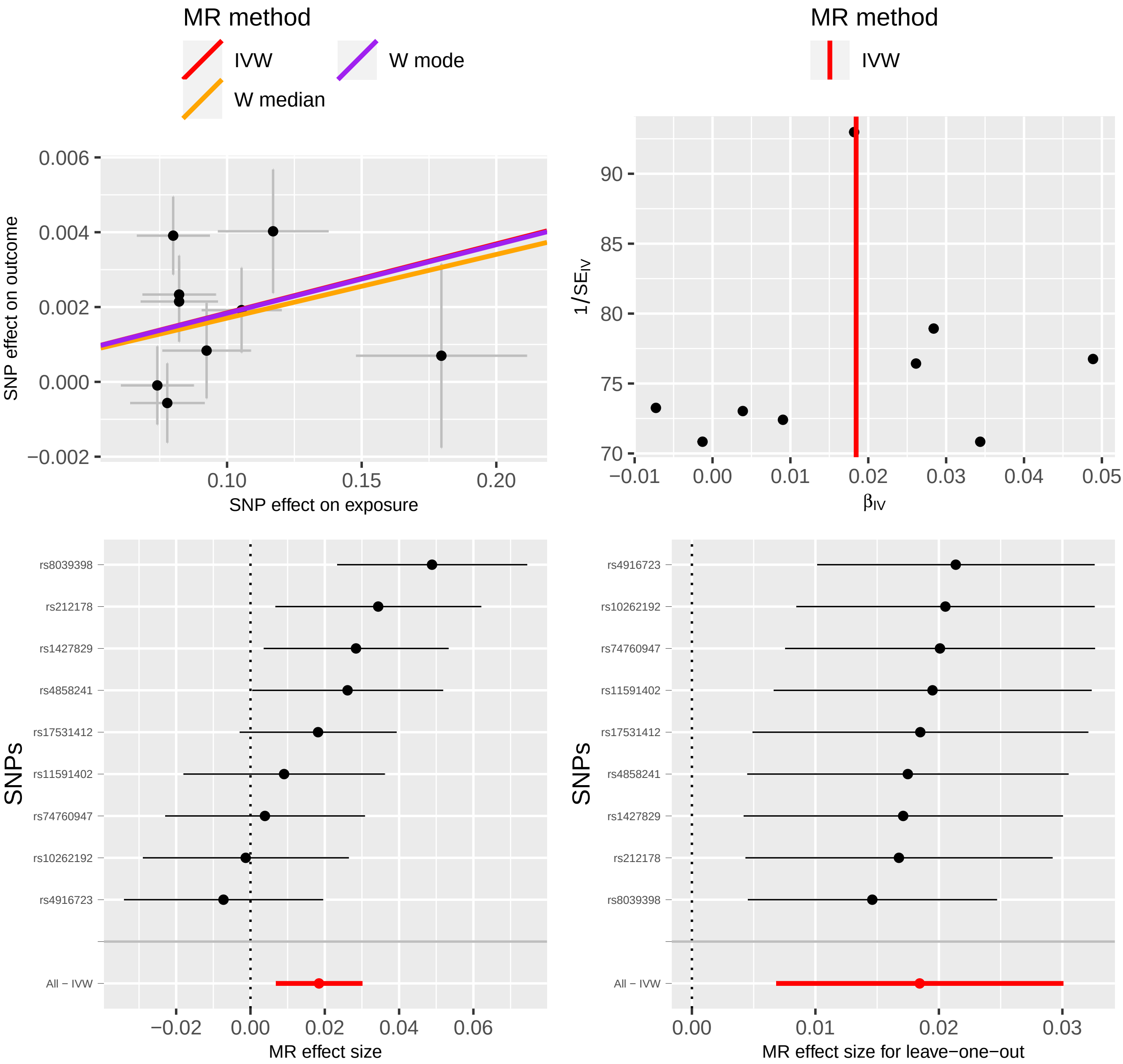

cp) Time spent watching television (TV) → ADHD

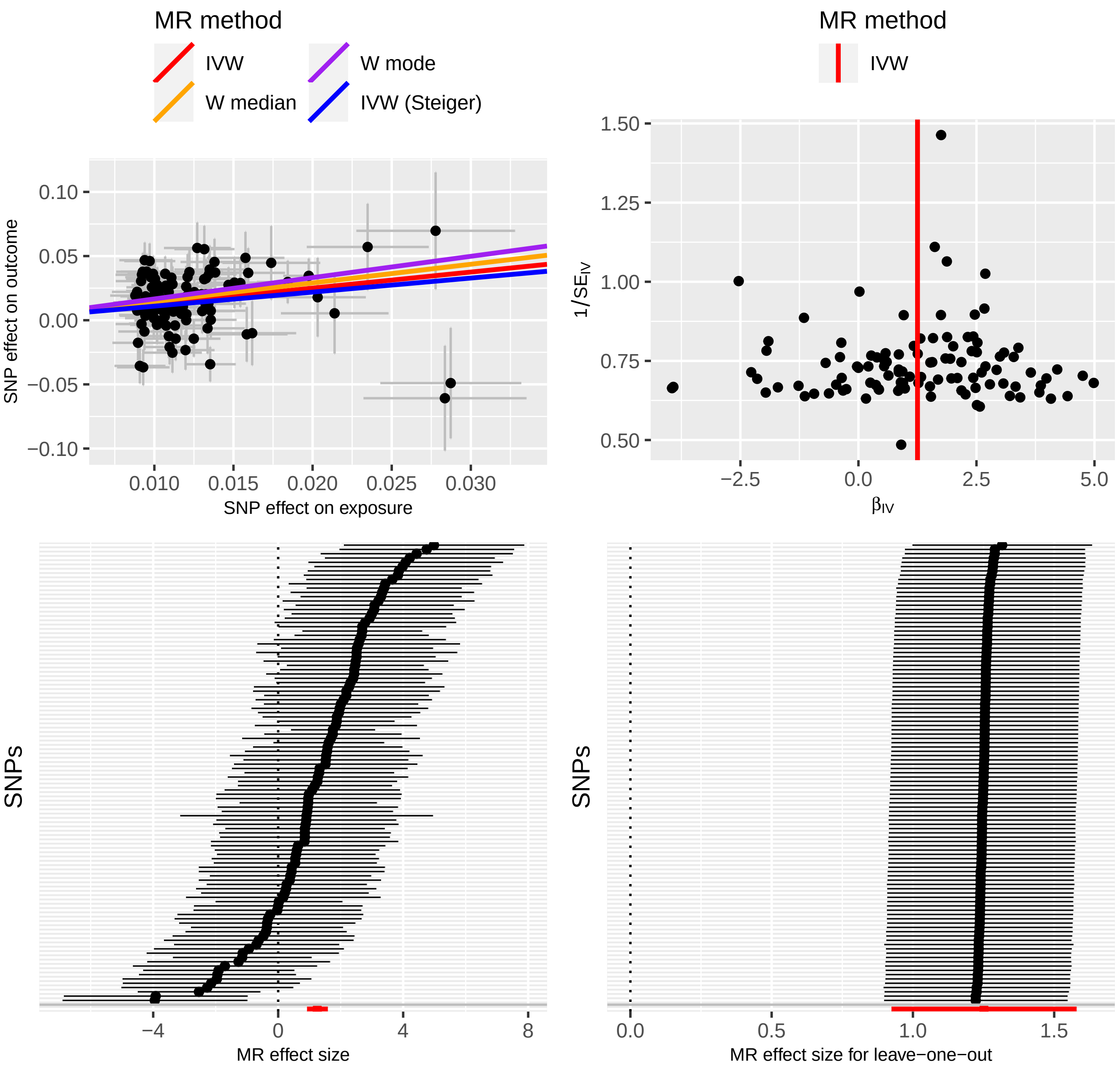

cq) Weekly usage of mobile phone in last 3 months → ADHD

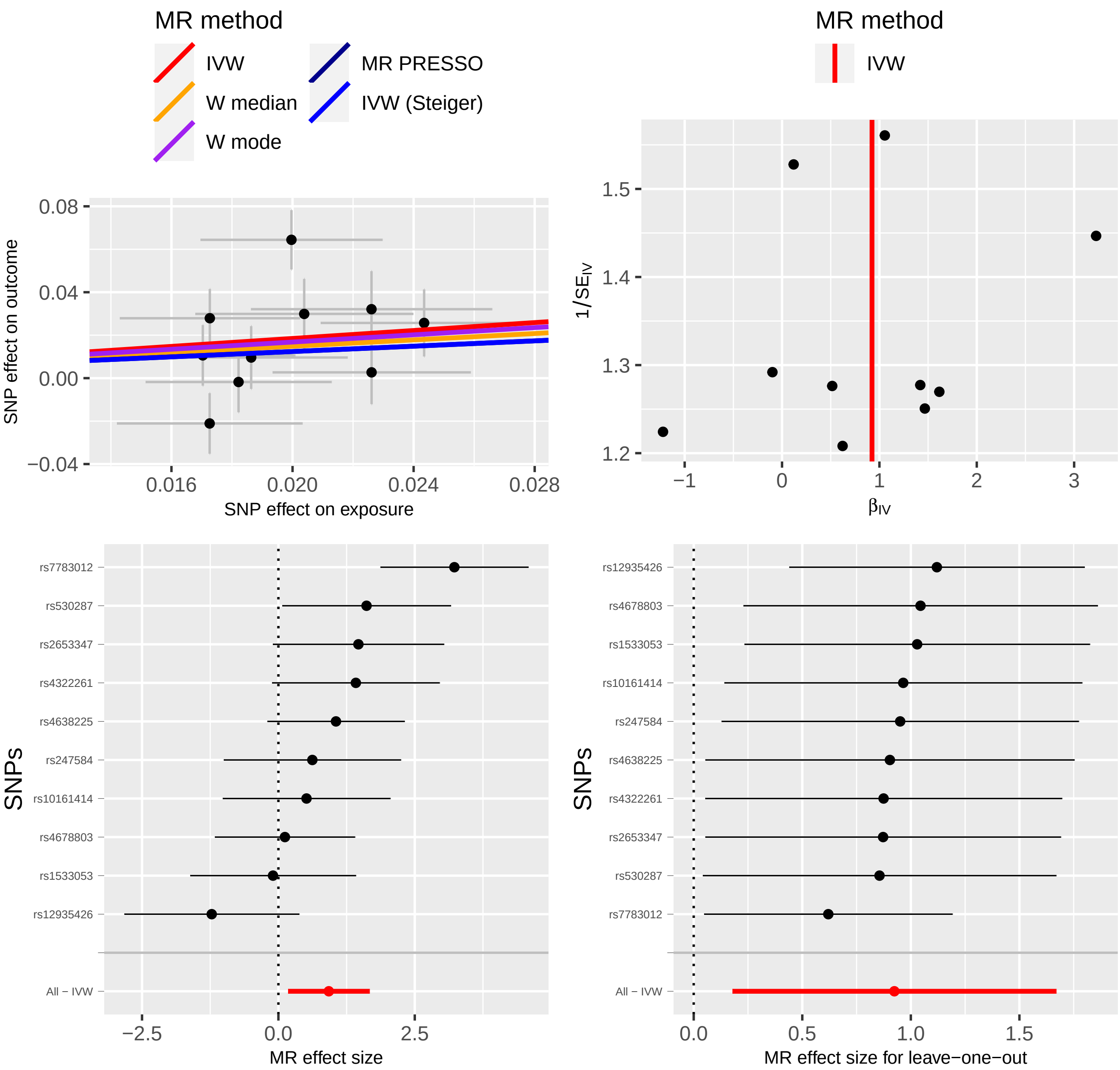

cr) ADHD → Weekly usage of mobile phone in last 3 months

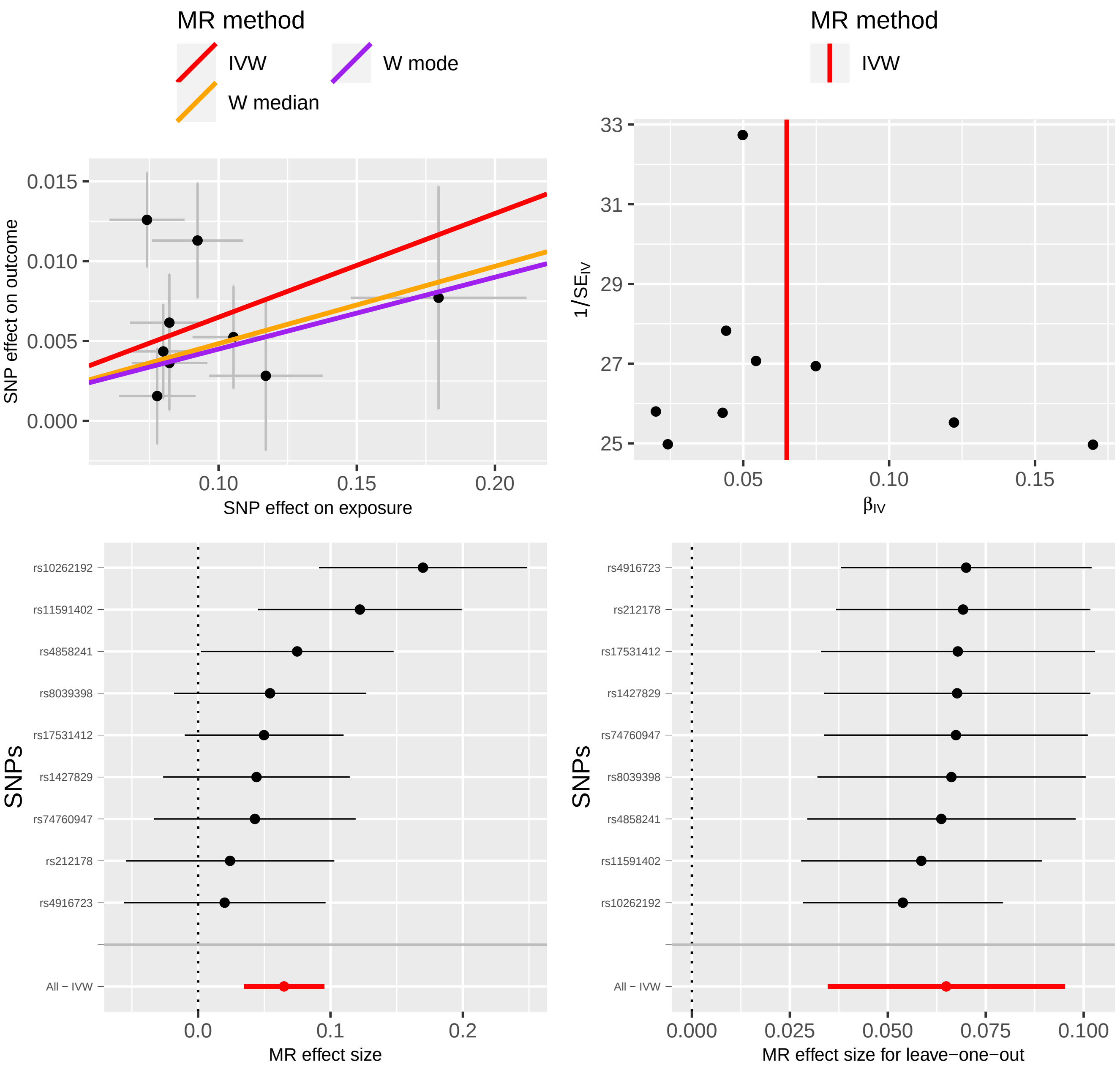

cs) Frequency of friend/family visits → ADHD

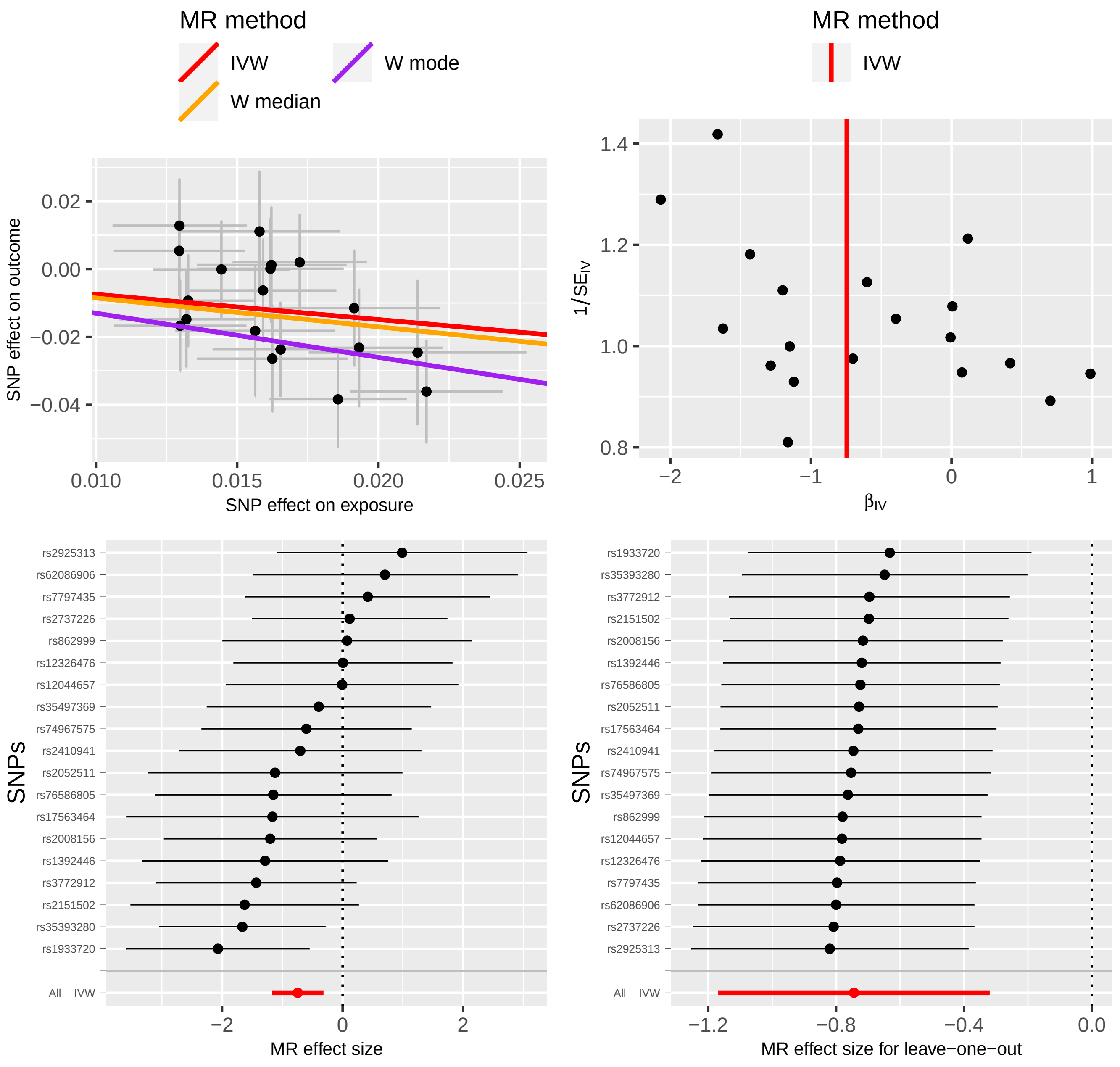

ct) ADHD → Usual walking pace

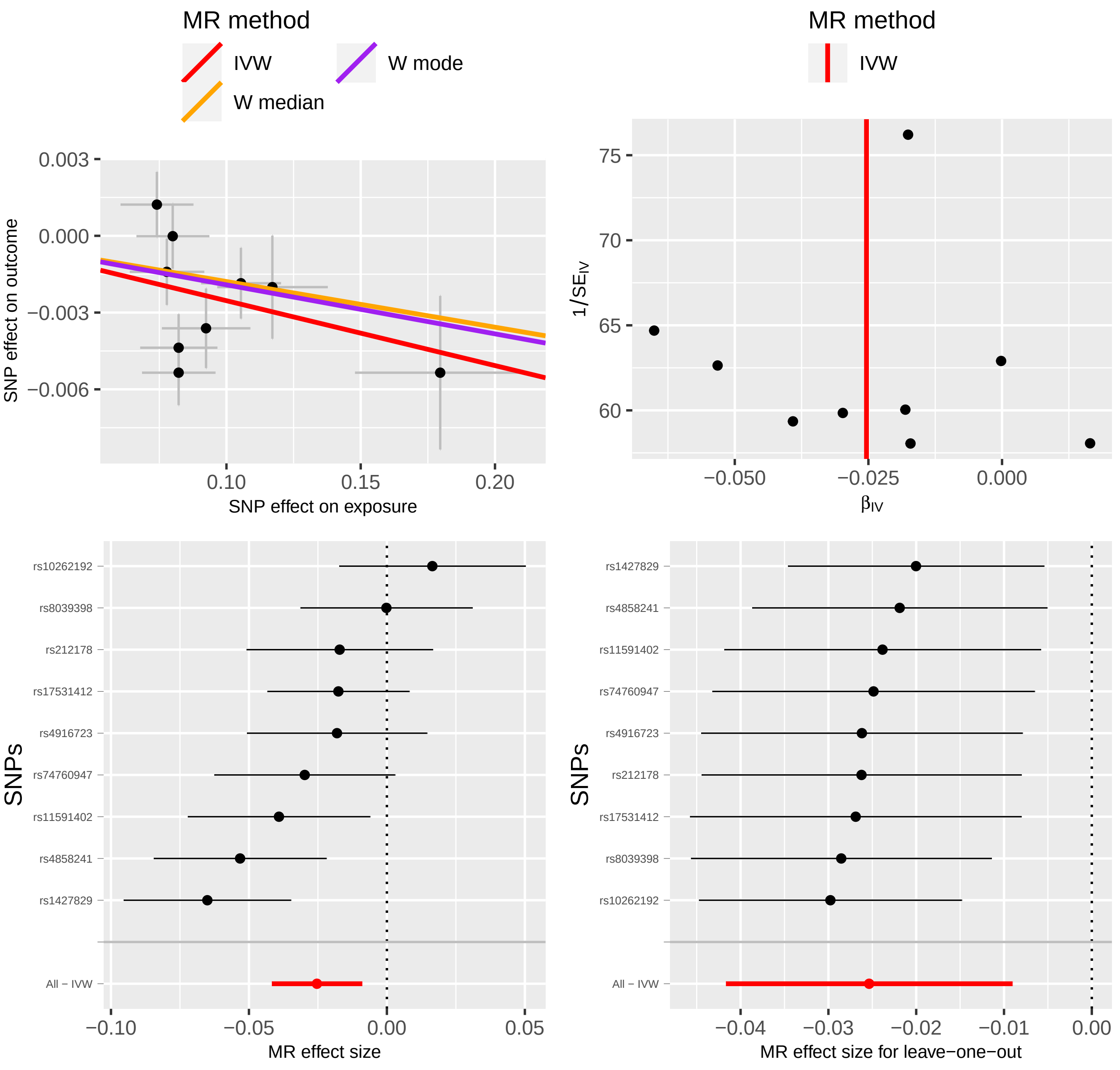

cu) Usual walking pace → ADHD

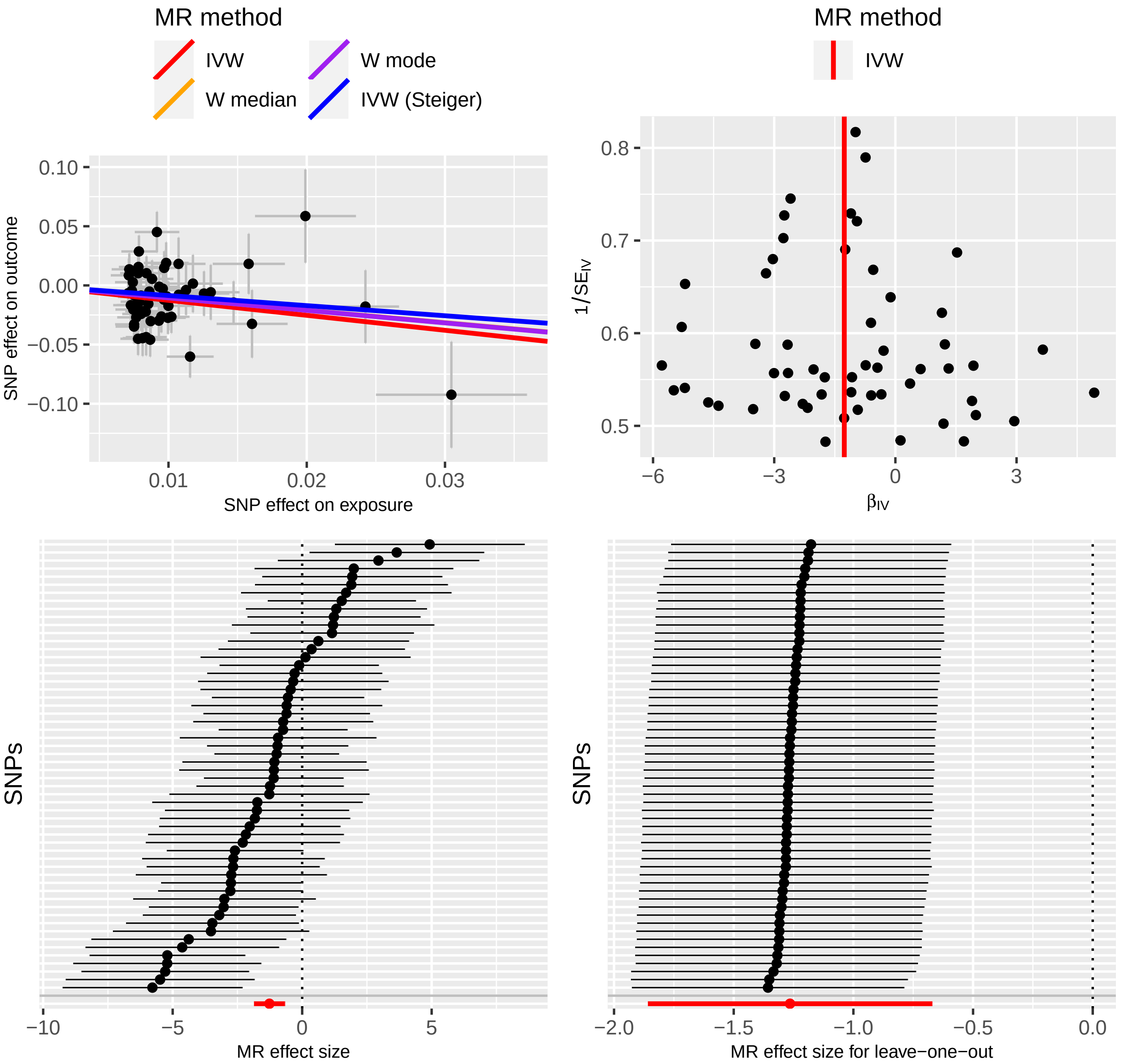

cv) ADHD → Parental longevity (both parents in top 10%)

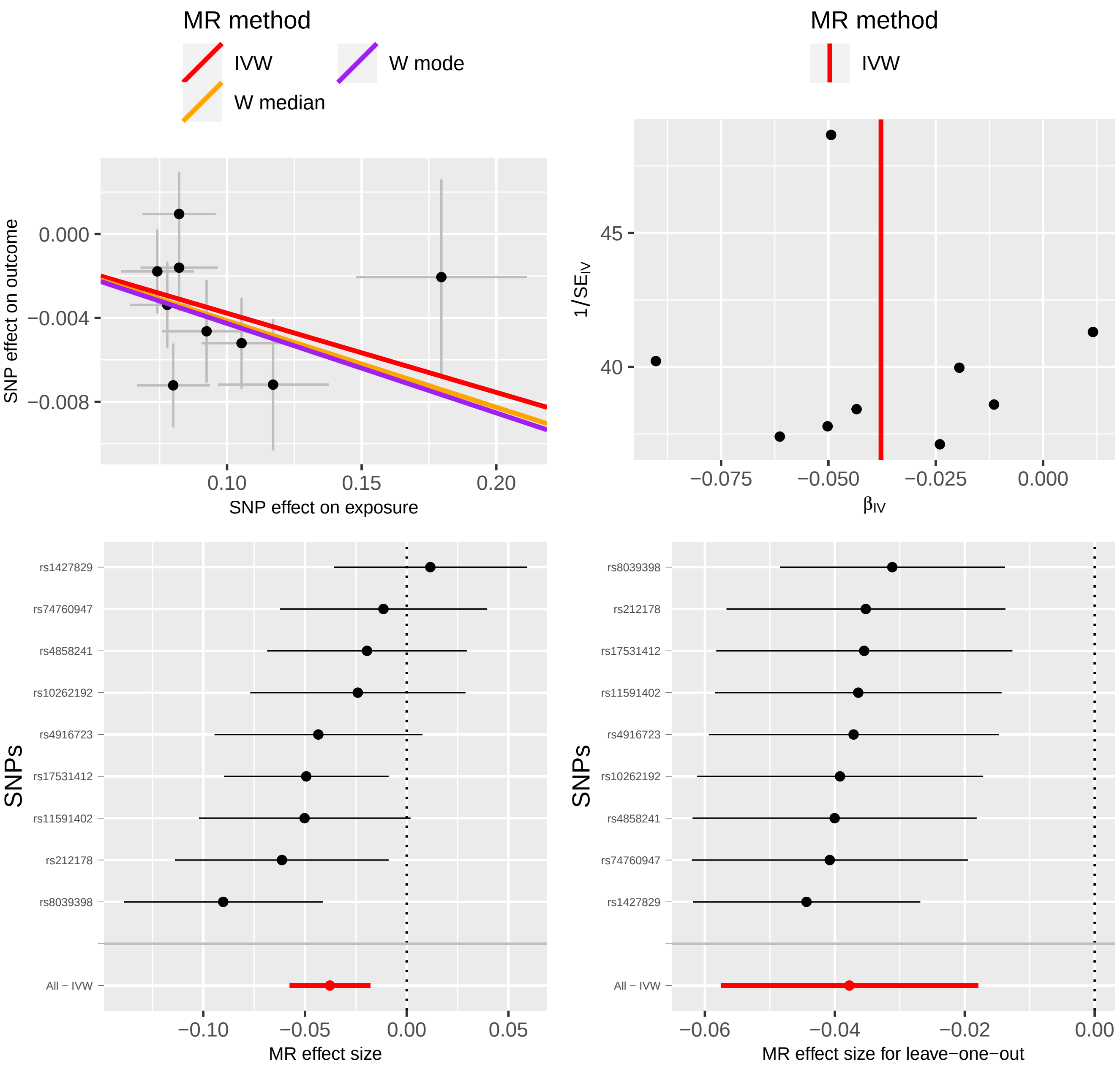

cw) ADHD → Parental longevity (combined parental age at death)

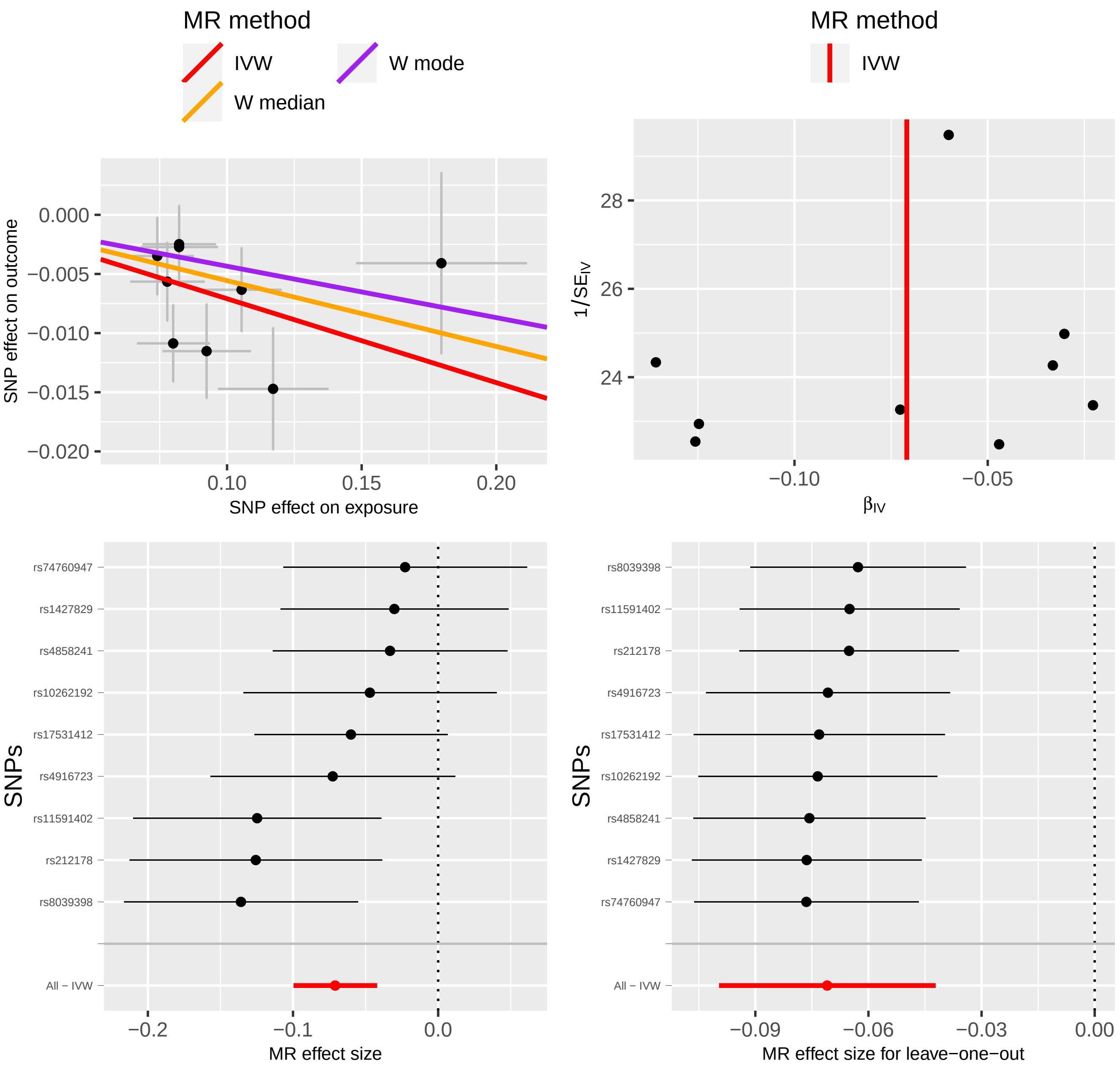

cx) ADHD → Parental longevity (combined parental attained age, Martingale residuals)

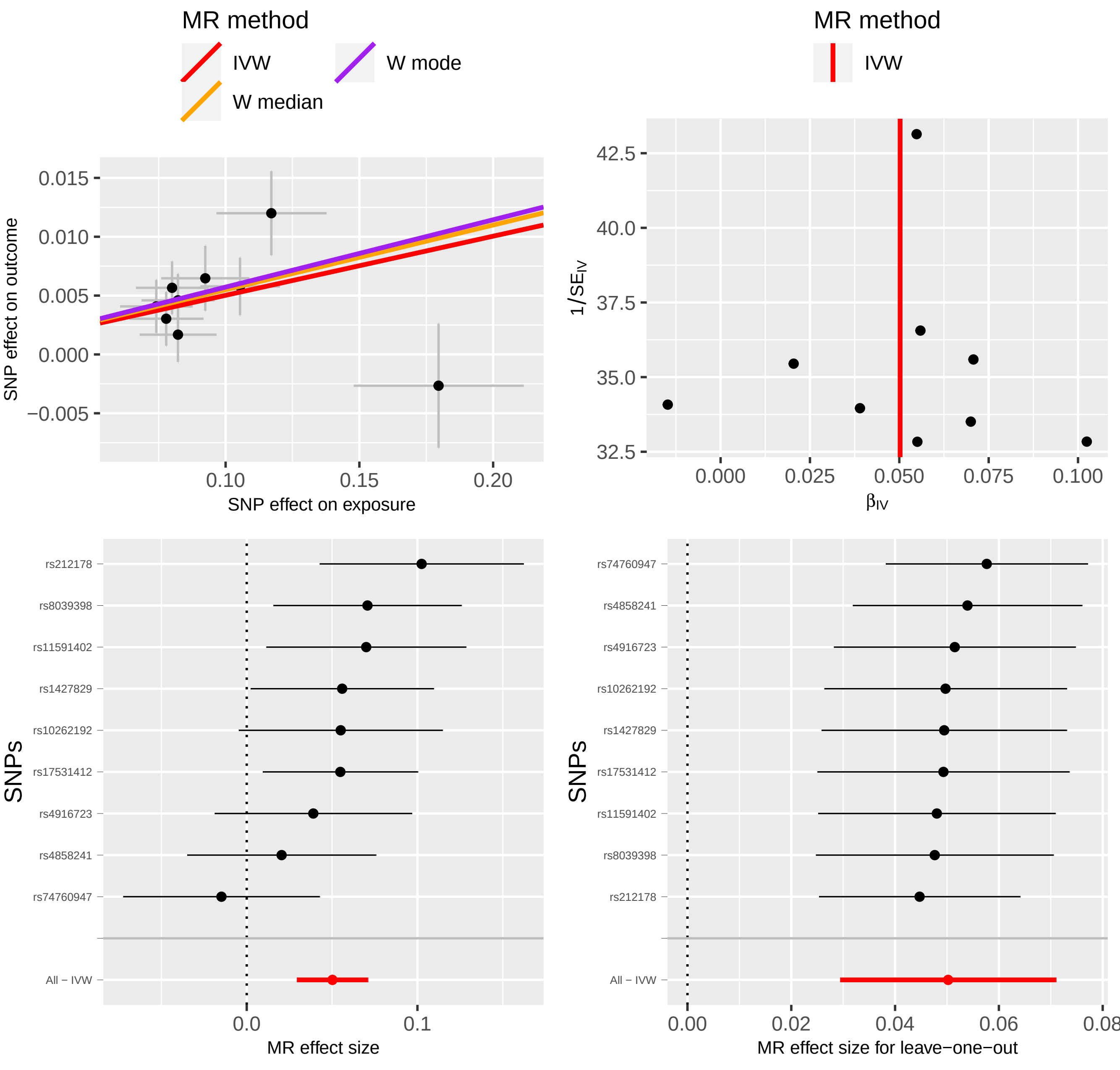

cy) ADHD → Parental longevity (father's age at death)

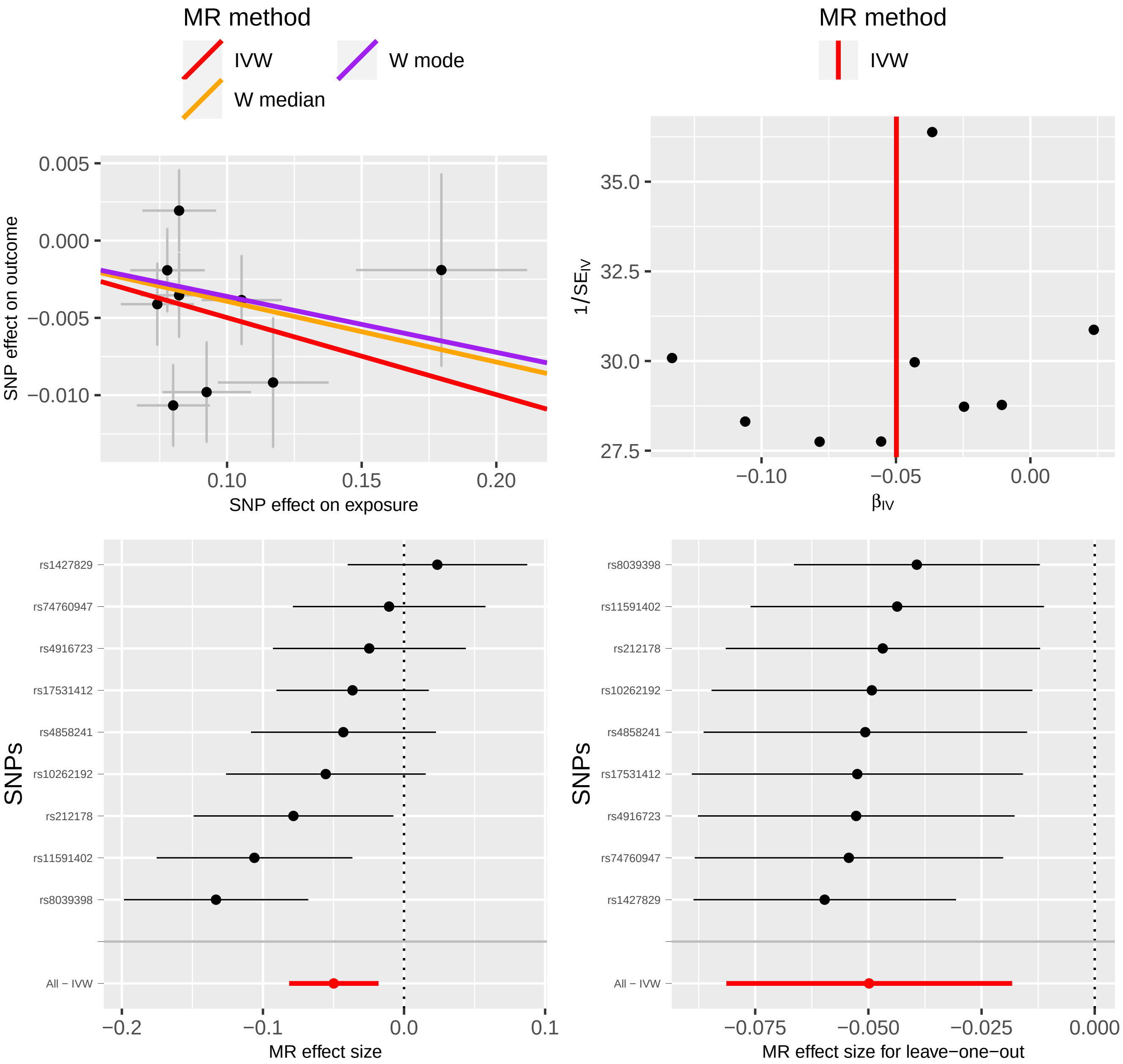

cz) ADHD → Parental longevity (father's attained age)

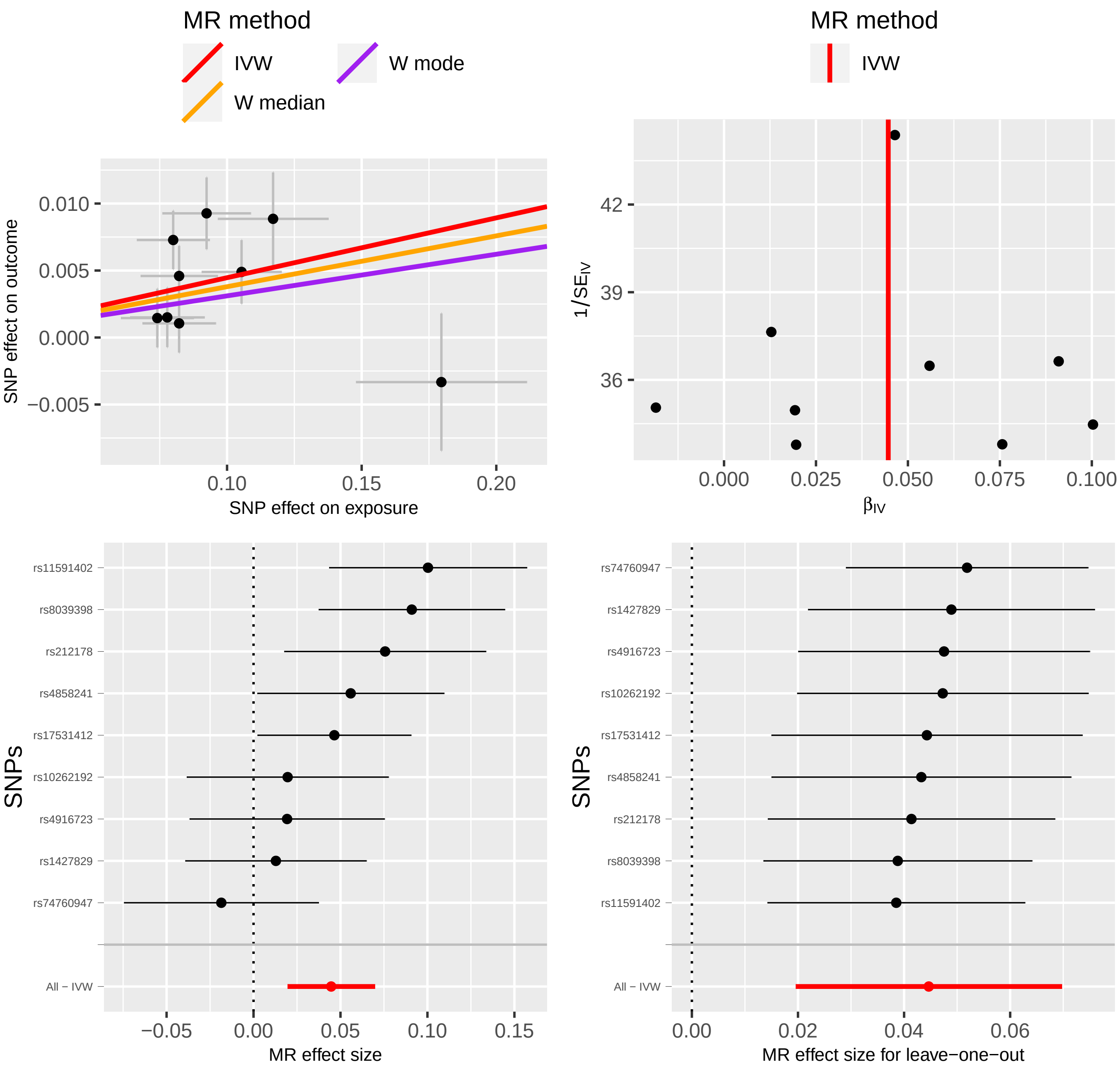

da) ADHD → Parental longevity (mother's attained age)

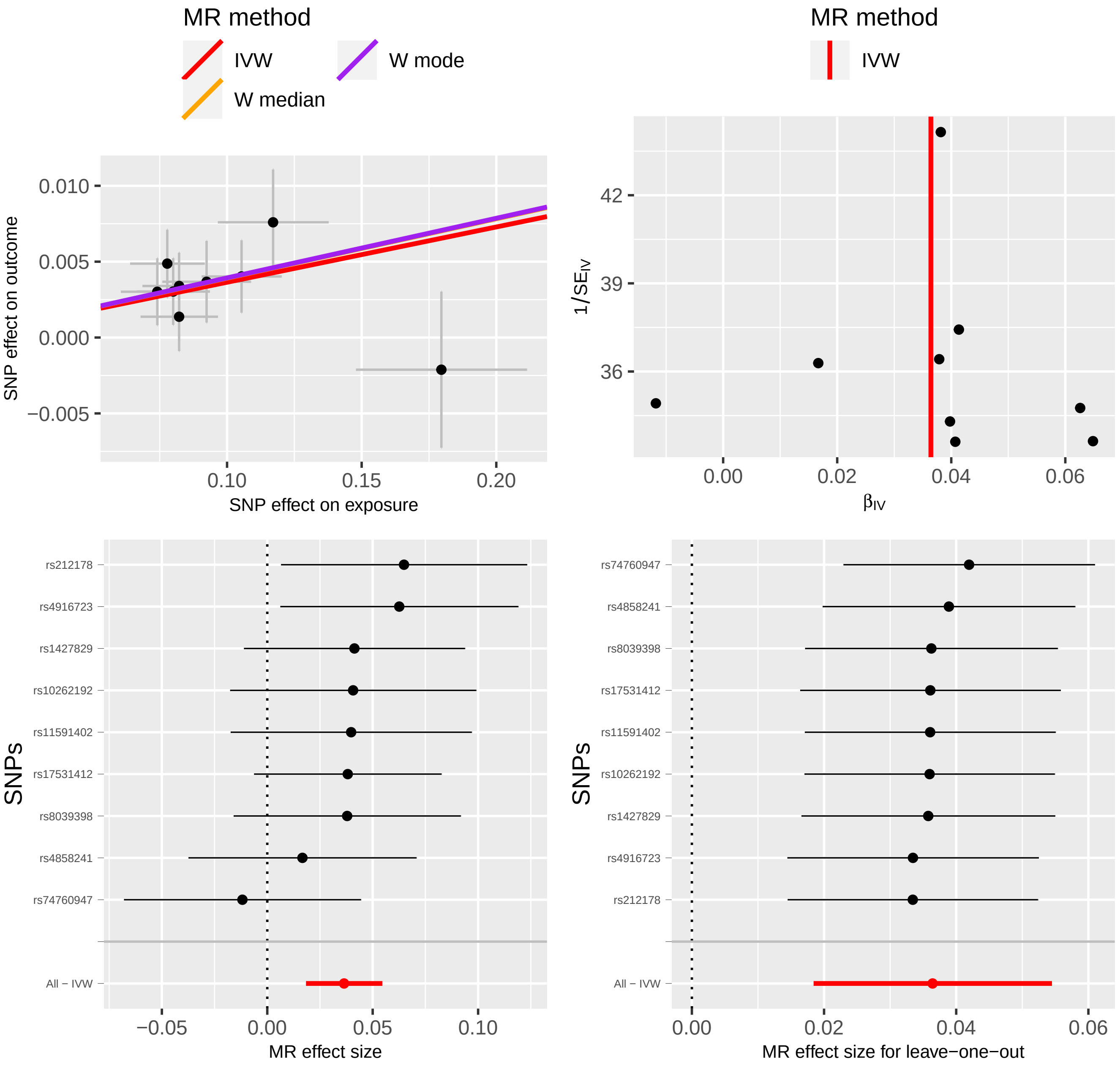

db) ADHD → Major Depressive Disorder

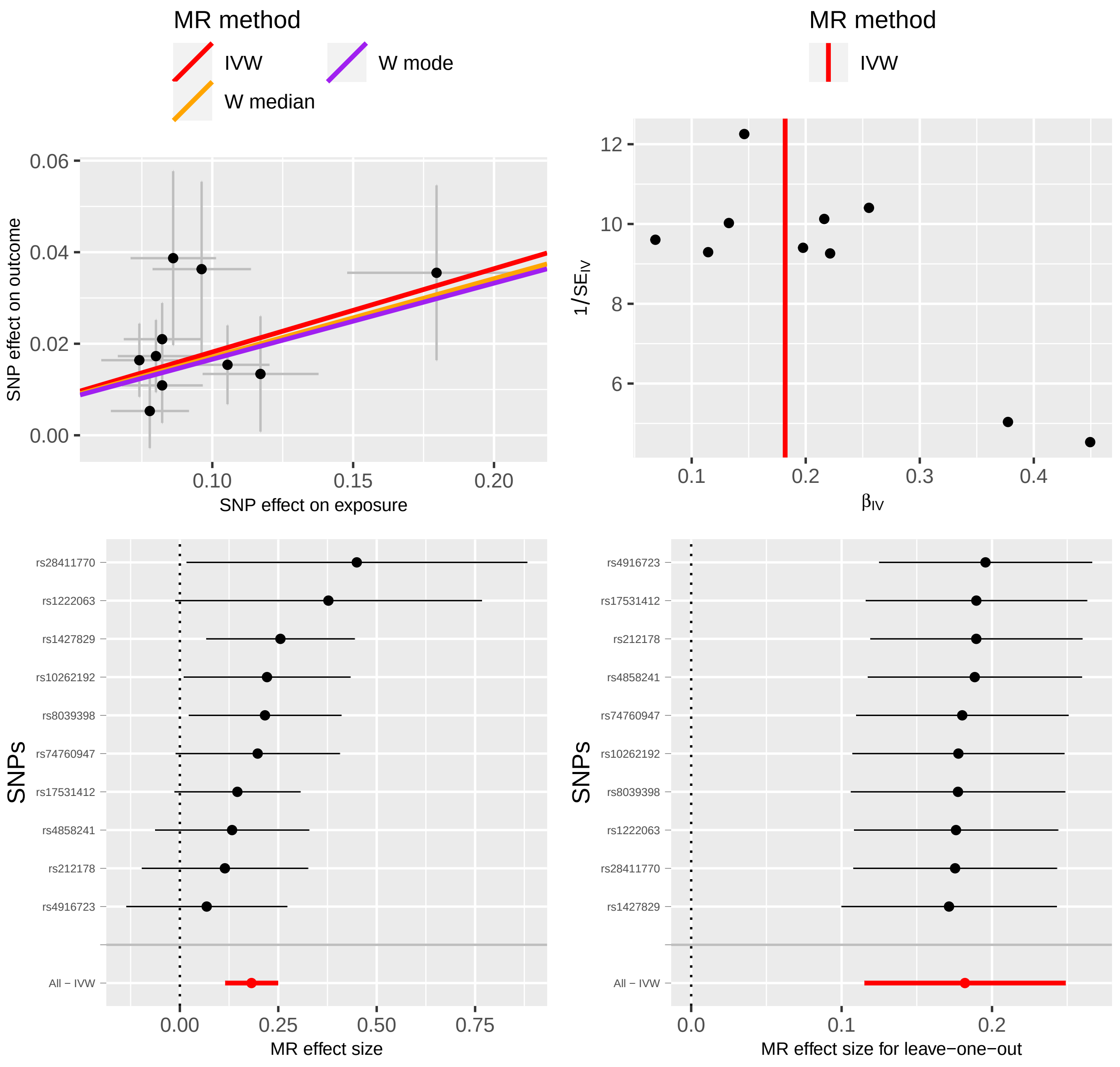

dc) Able to confide → ADHD

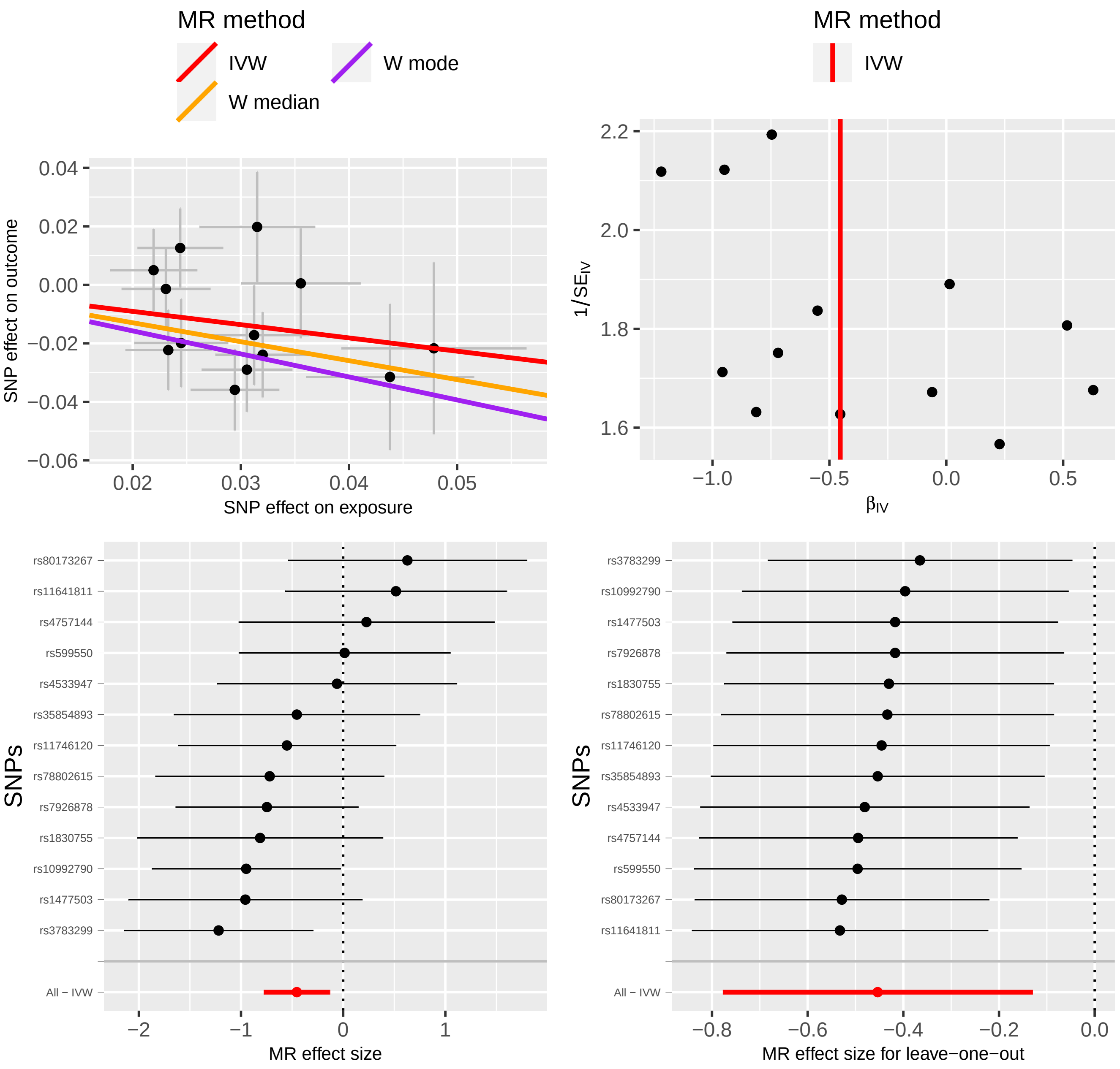

dd) ADHD → Able to confide

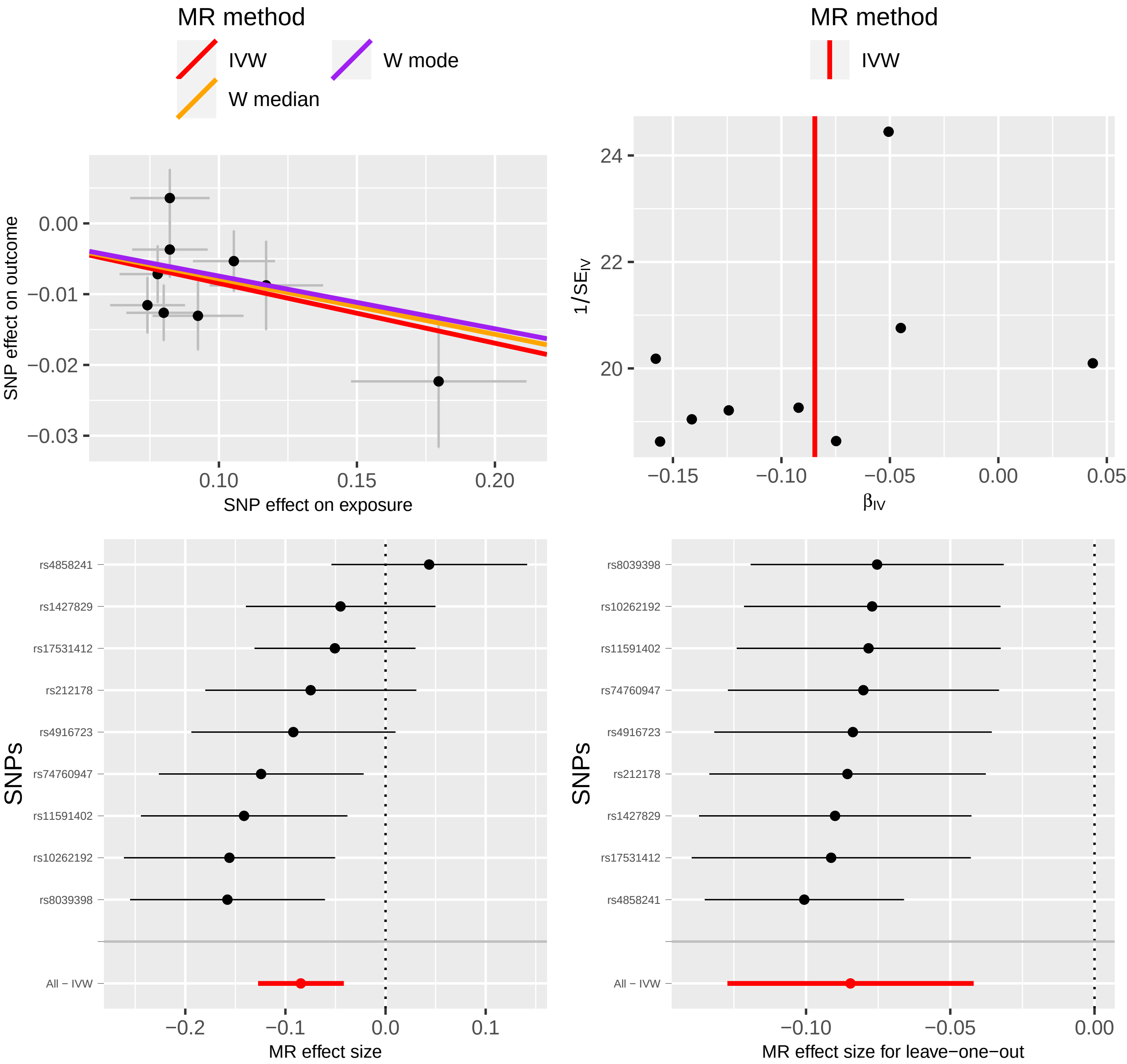

de) Experiencing mood swings → ADHD

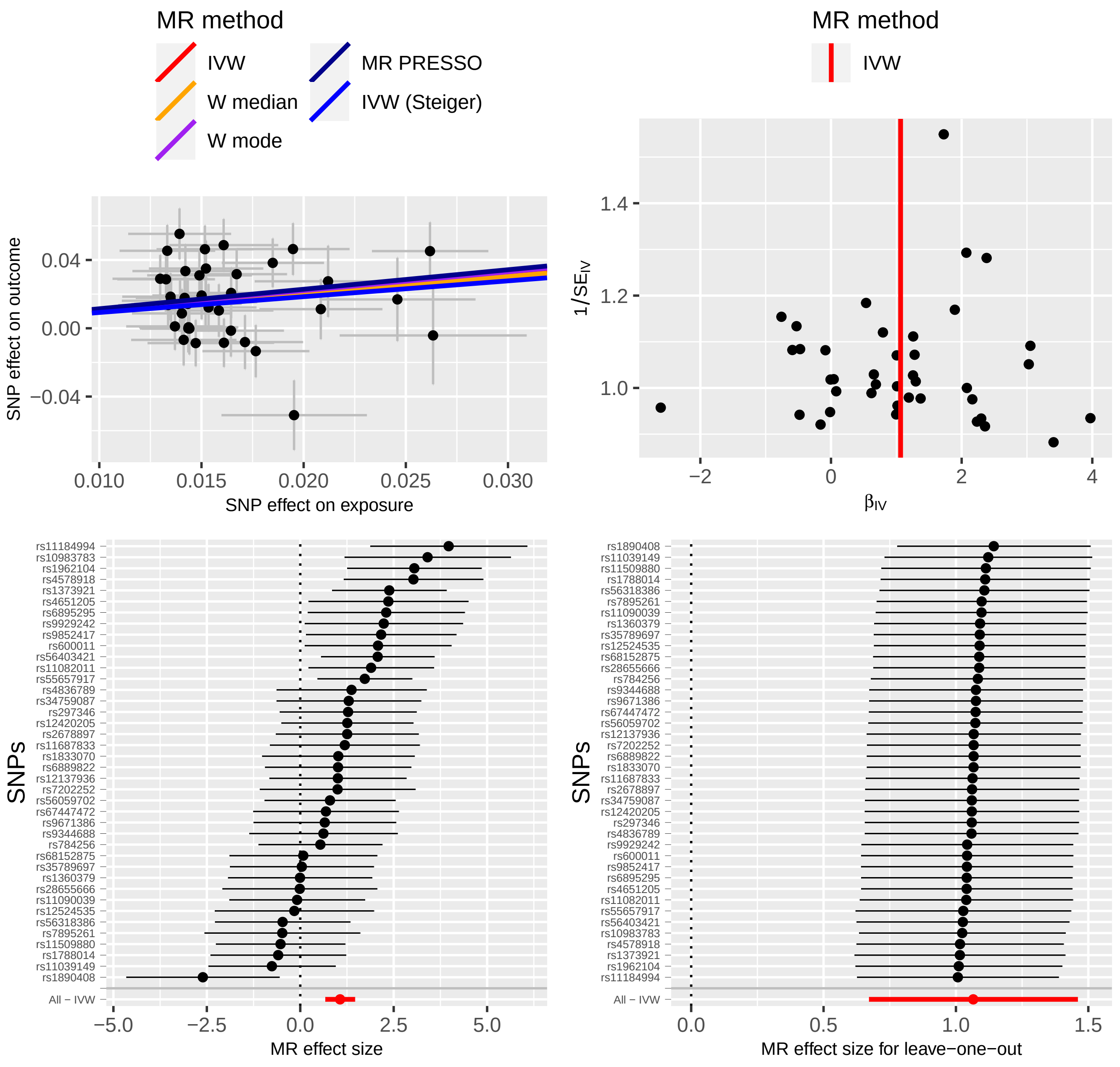

df) Feeling fed−up → ADHD

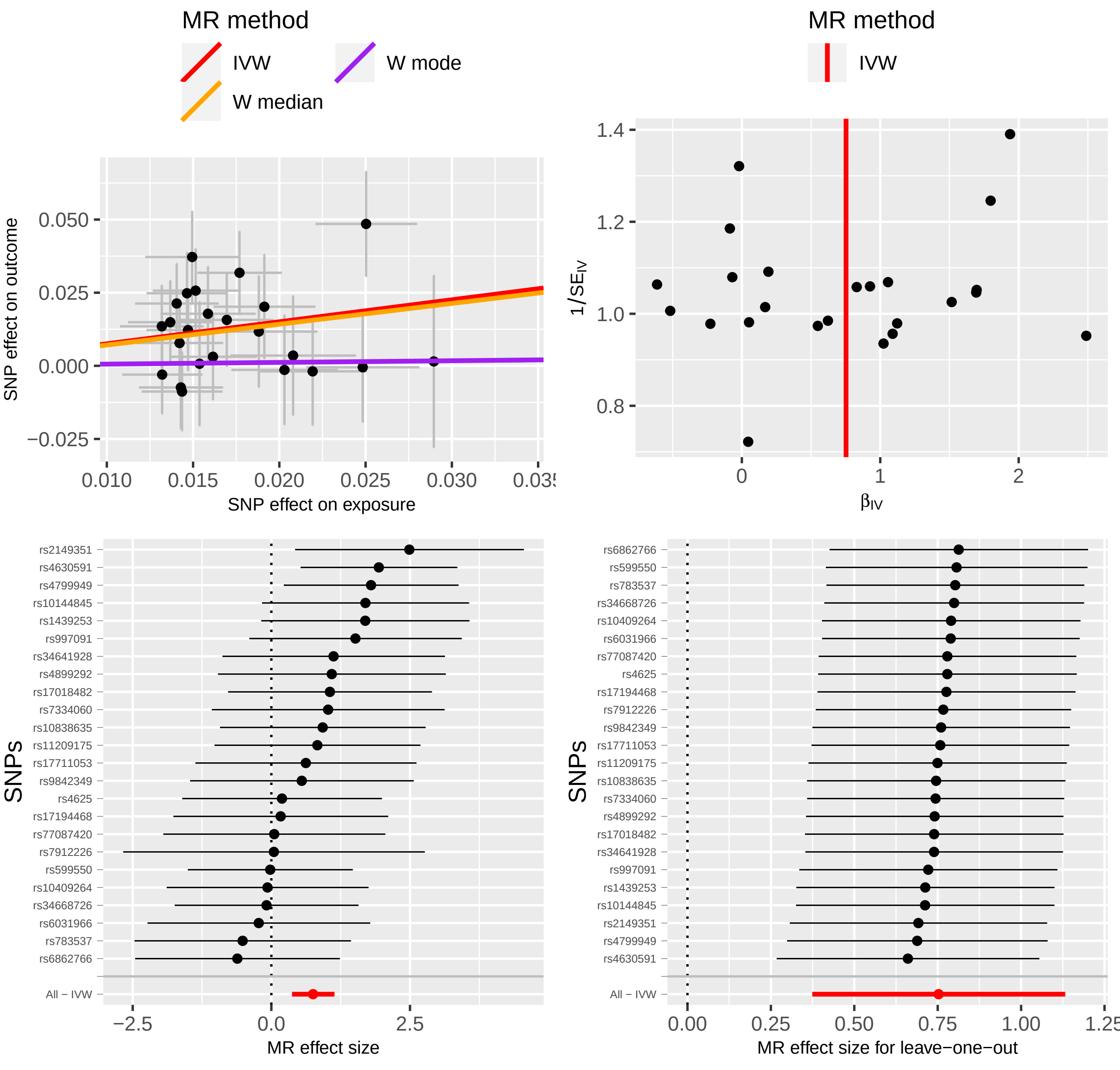

dg) Feeling guilty → ADHD

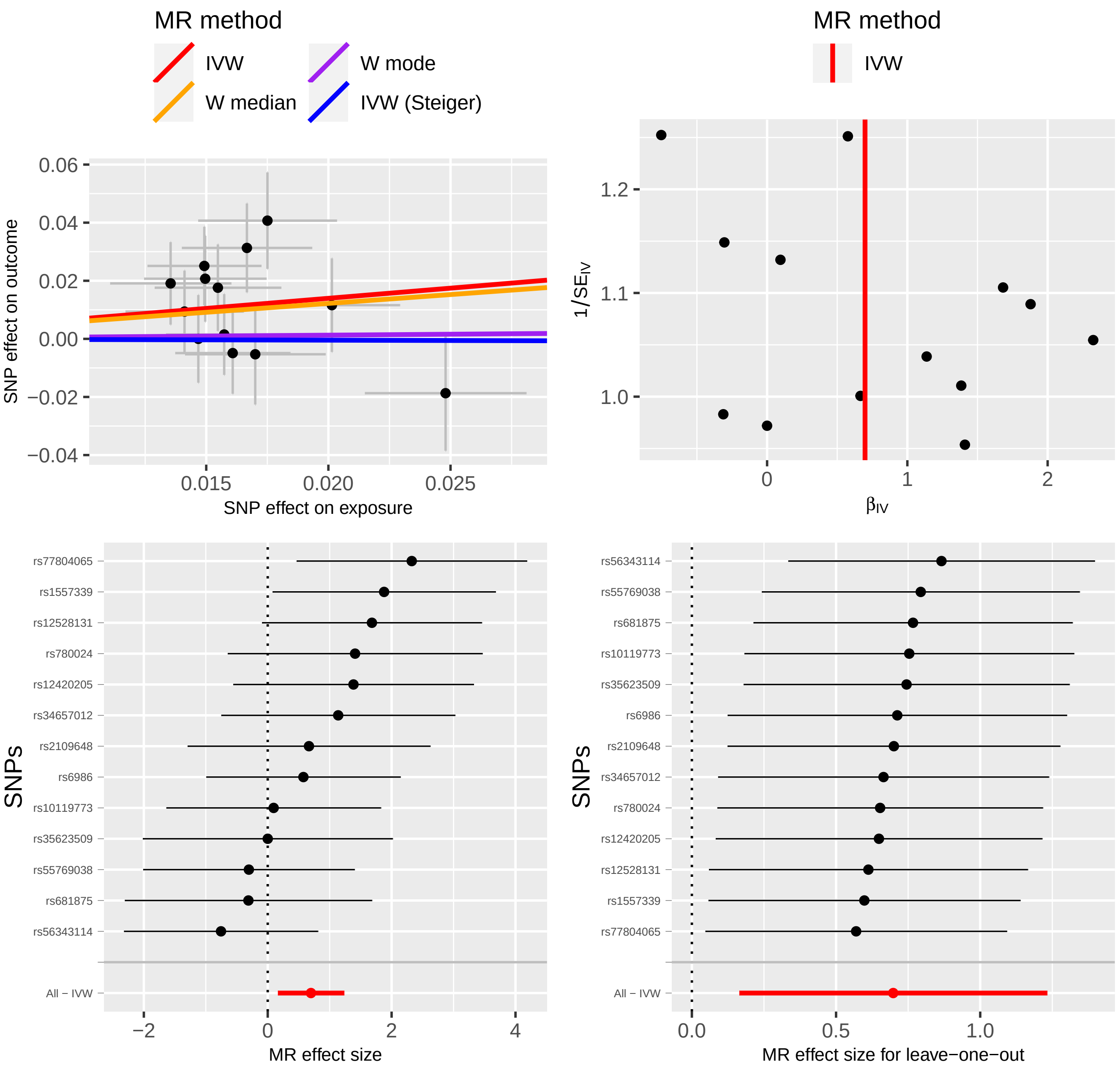

dh) Feeling hurt → ADHD

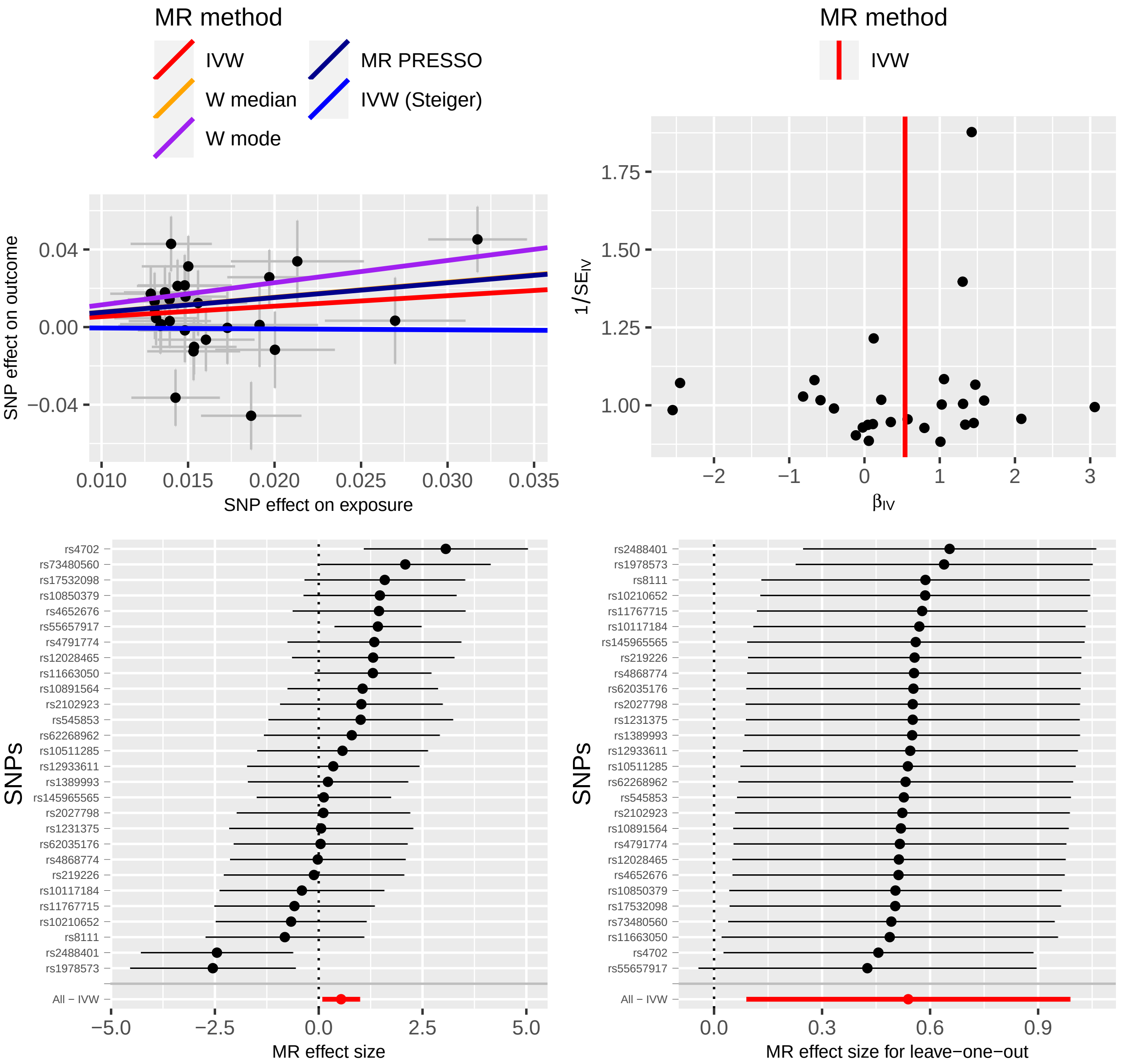

di) ADHD → Feeling lonely

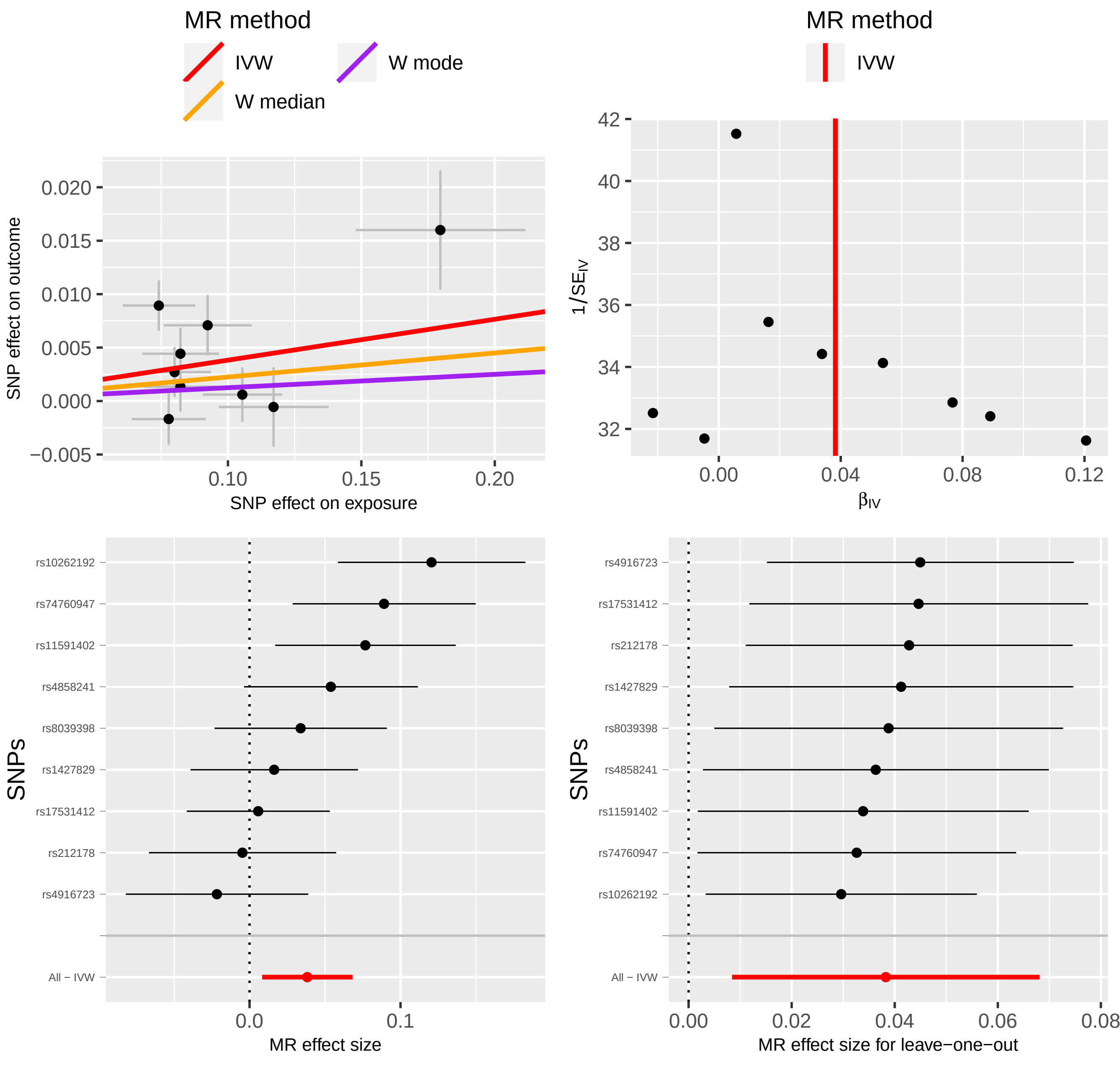

dj) Feeling miserable → ADHD

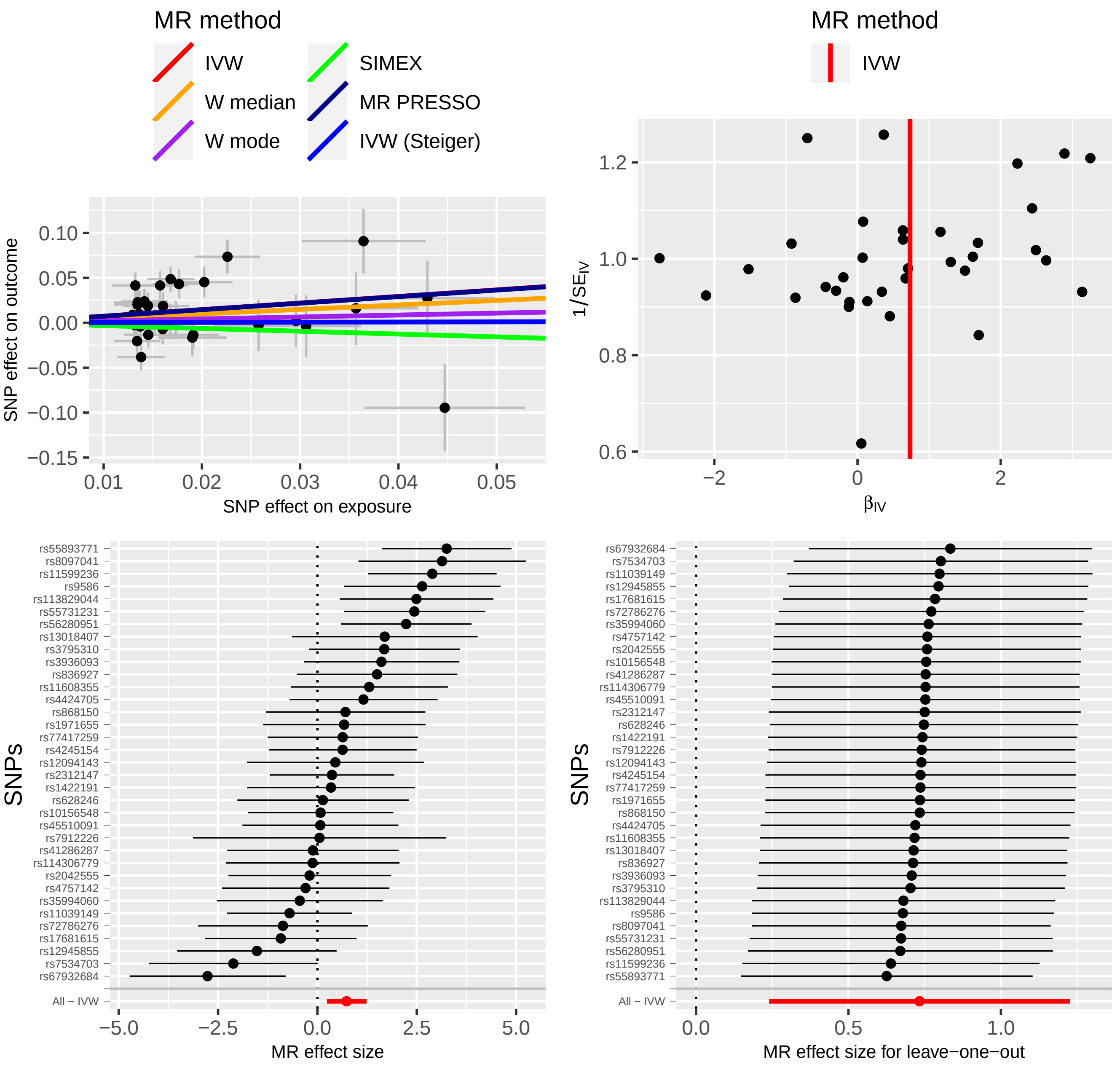

dk) Frequency of depressed mood in last 2 weeks → ADHD

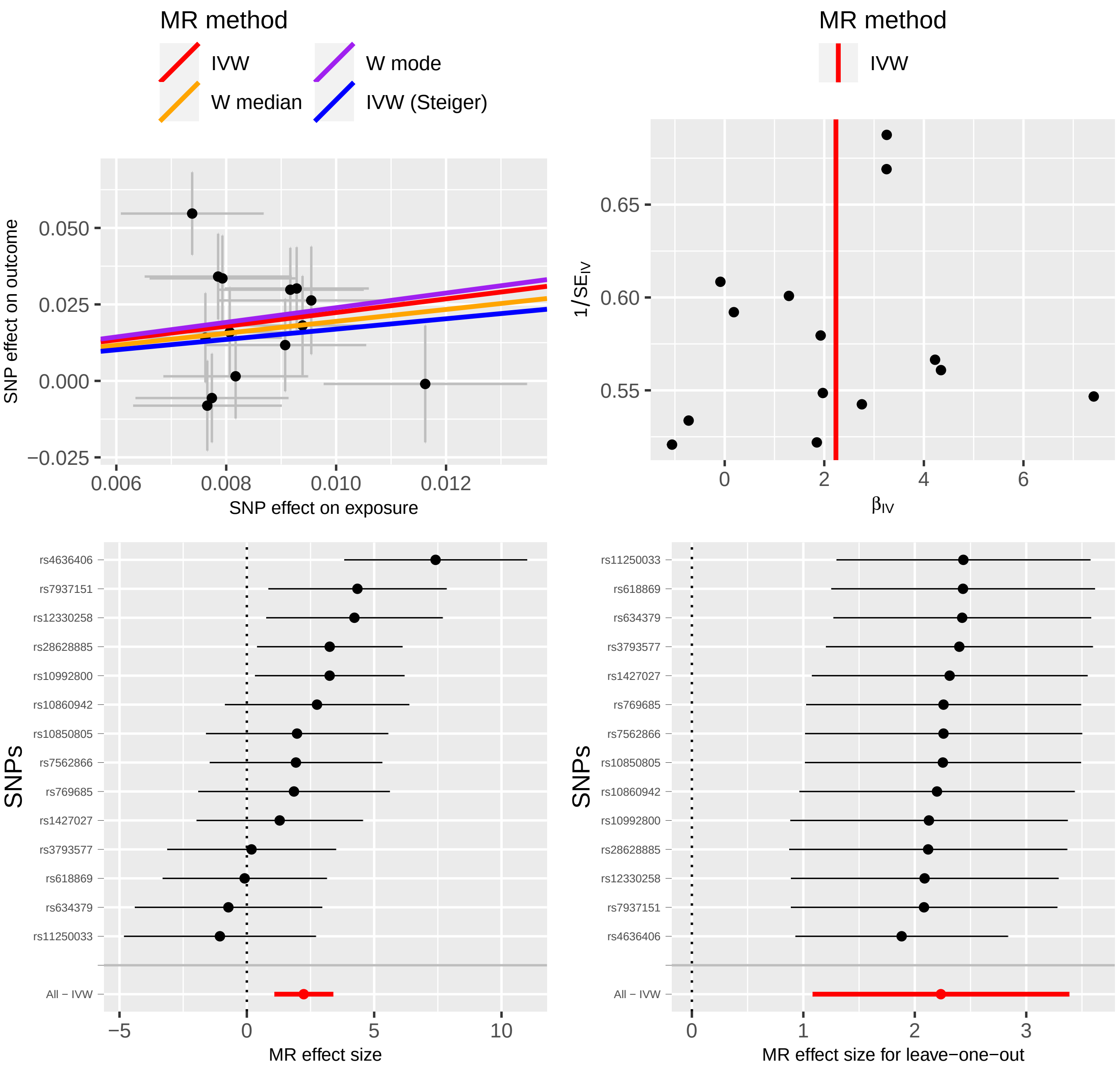

dl) ADHD → Frequency of depressed mood in last 2 weeks

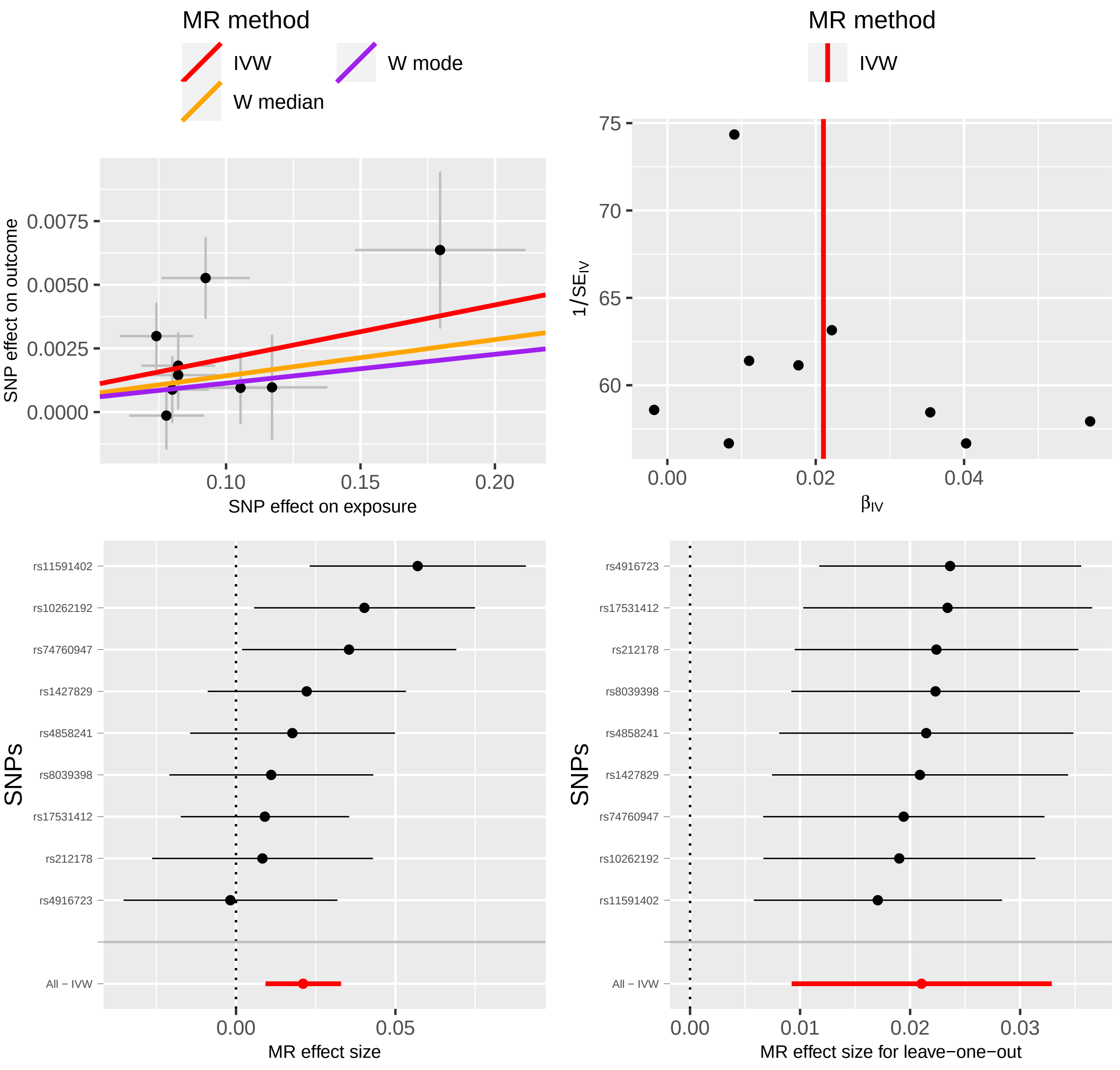

dm) Frequency of tiredness / lethargy in last 2 weeks → ADHD

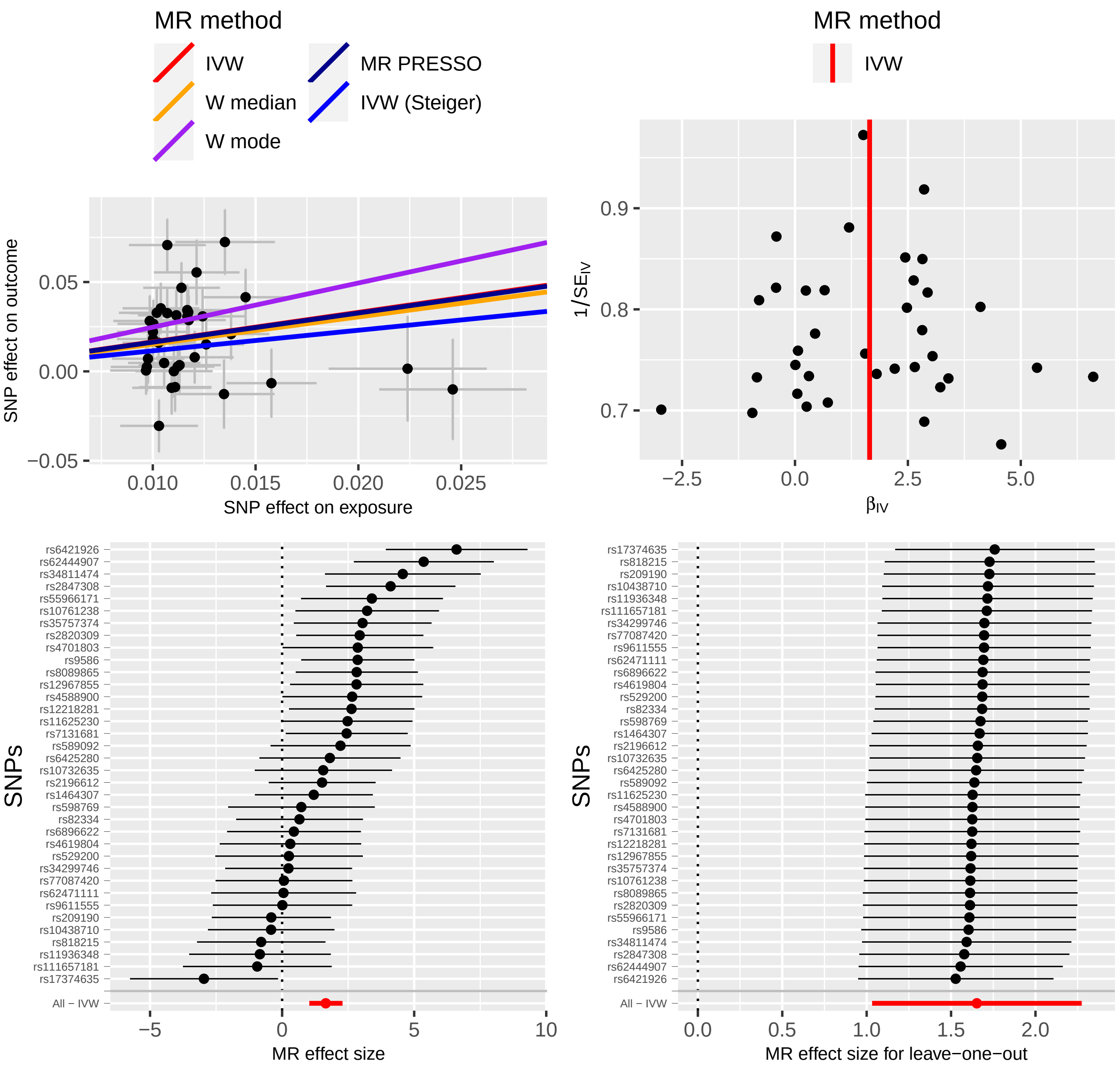

dn) Frequency of unenthusiasm / disinterest in last 2 weeks → ADHD

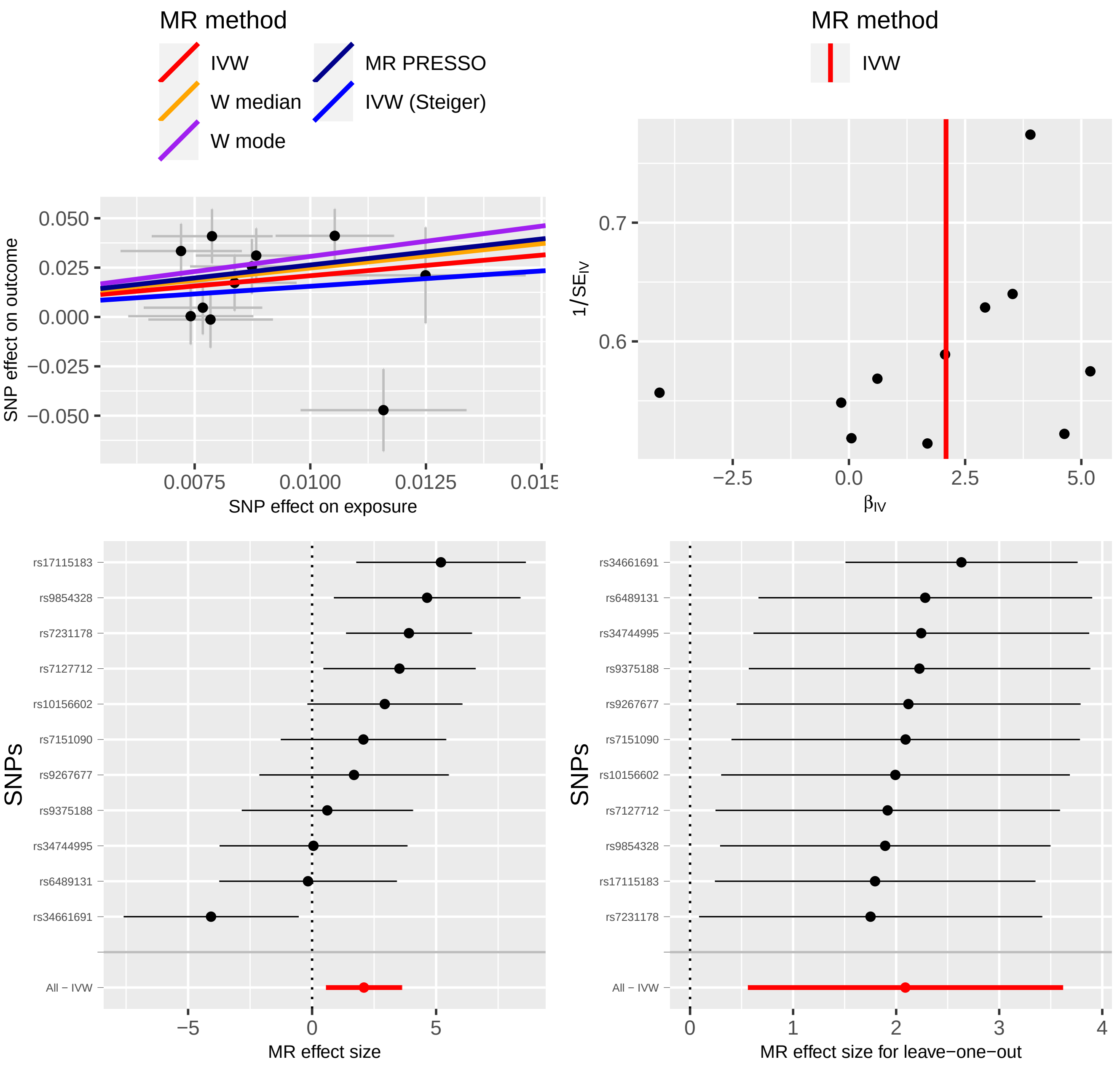

do) ADHD → Frequency of unenthusiasm / disinterest in last 2 weeks

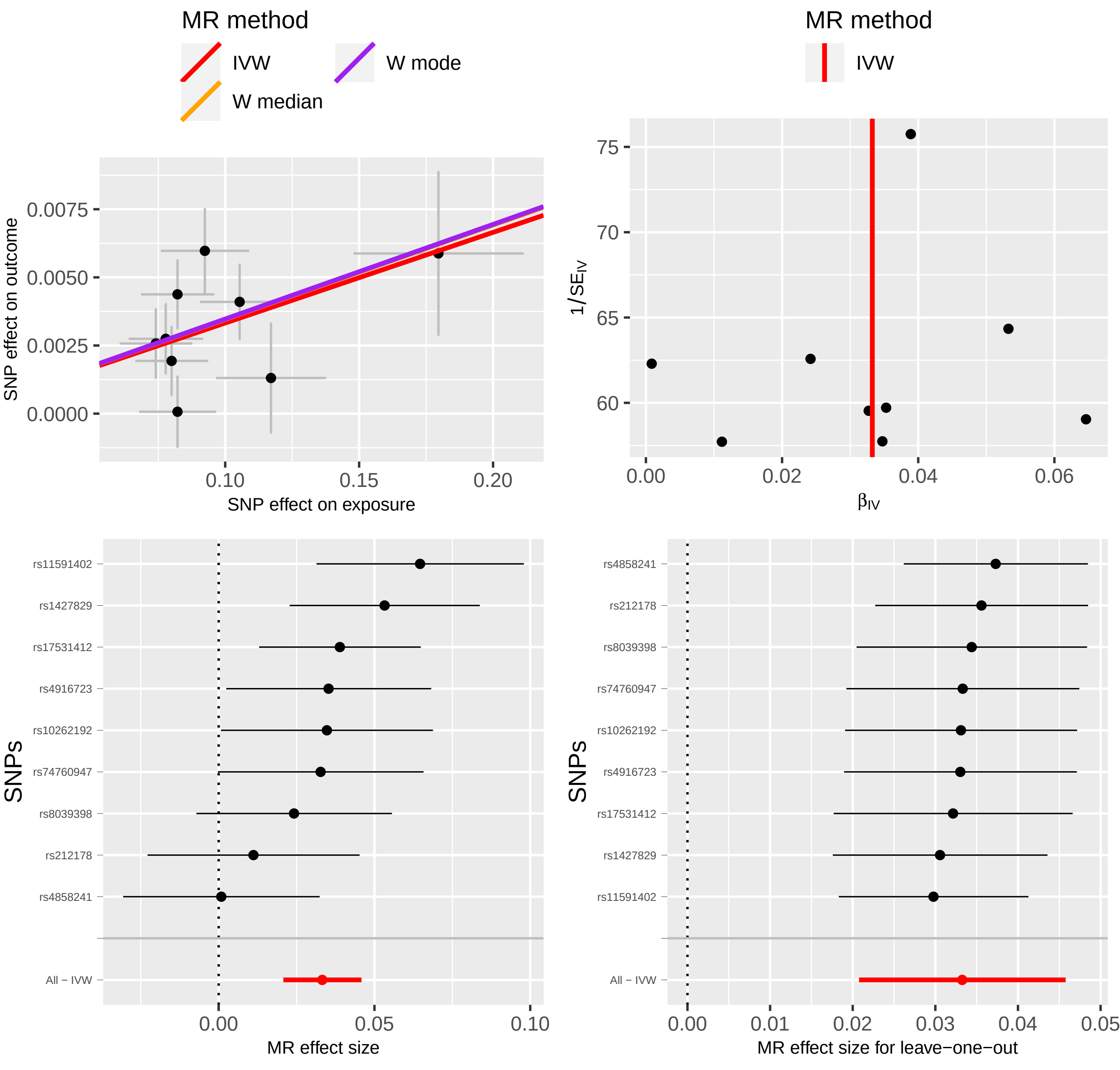

dp) Irritable mood → ADHD

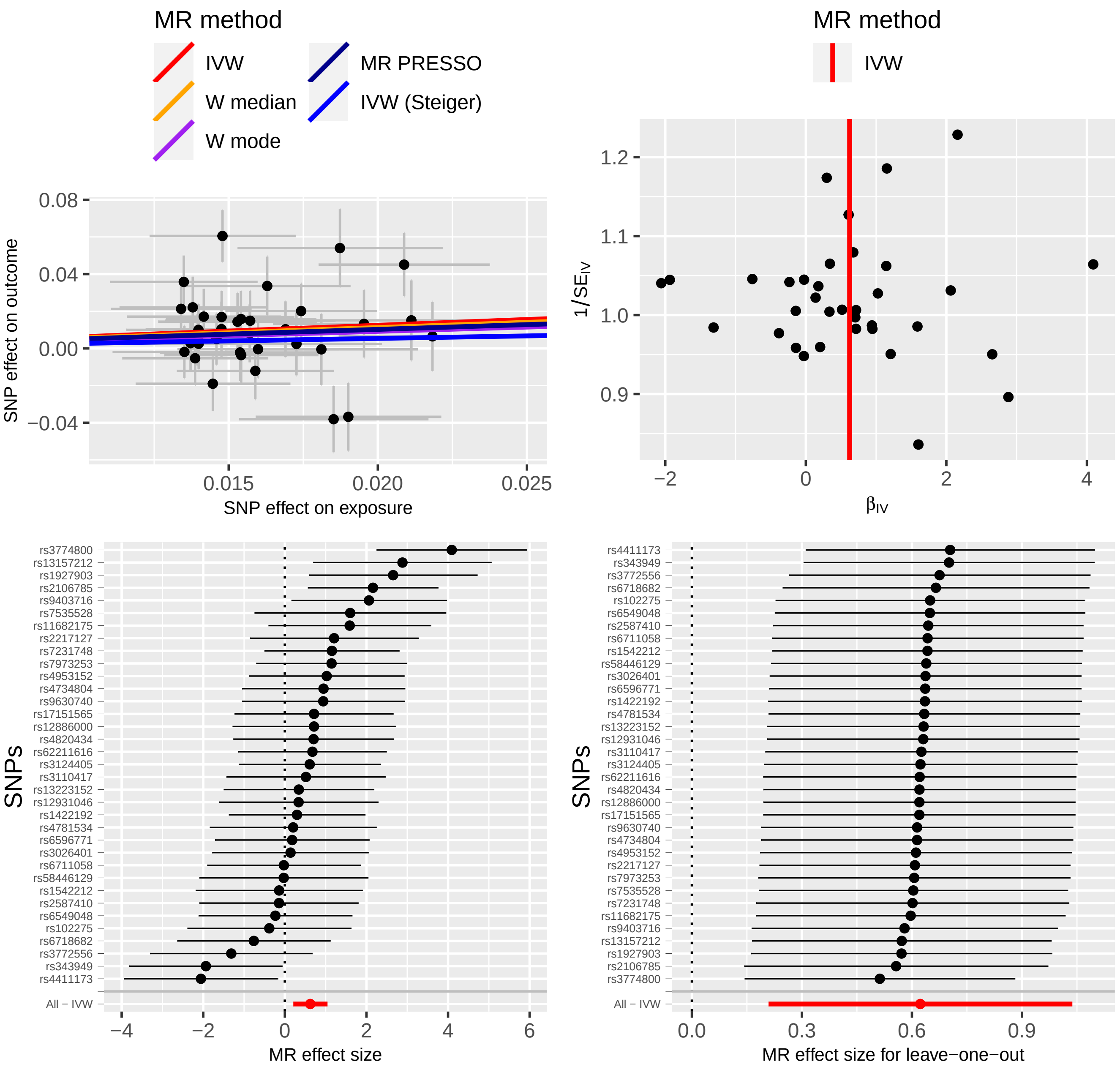

dq) Neurociticism → ADHD

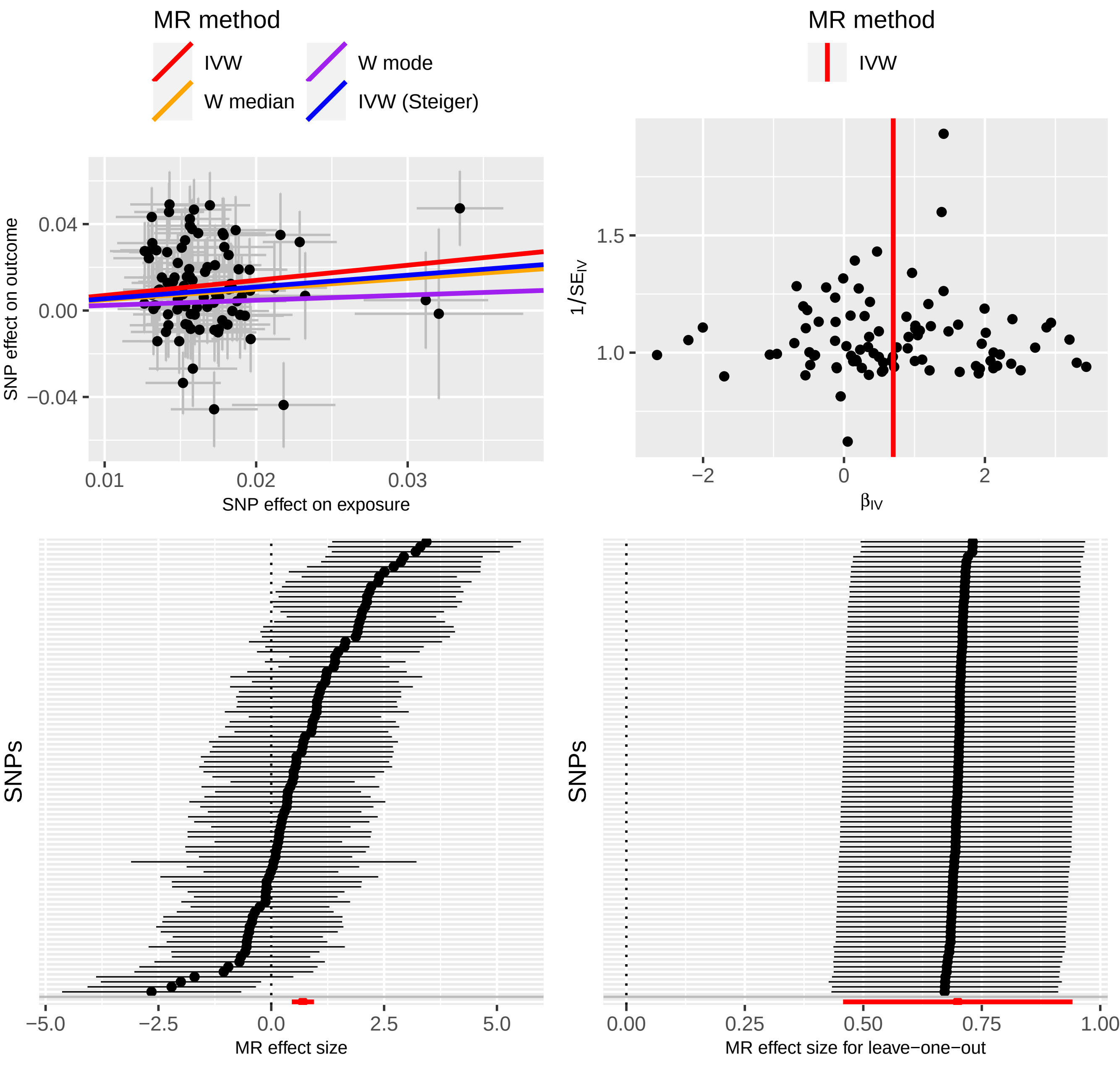

dr) Seen doctor (GP) for nerves, anxiety, tension or depression → ADHD

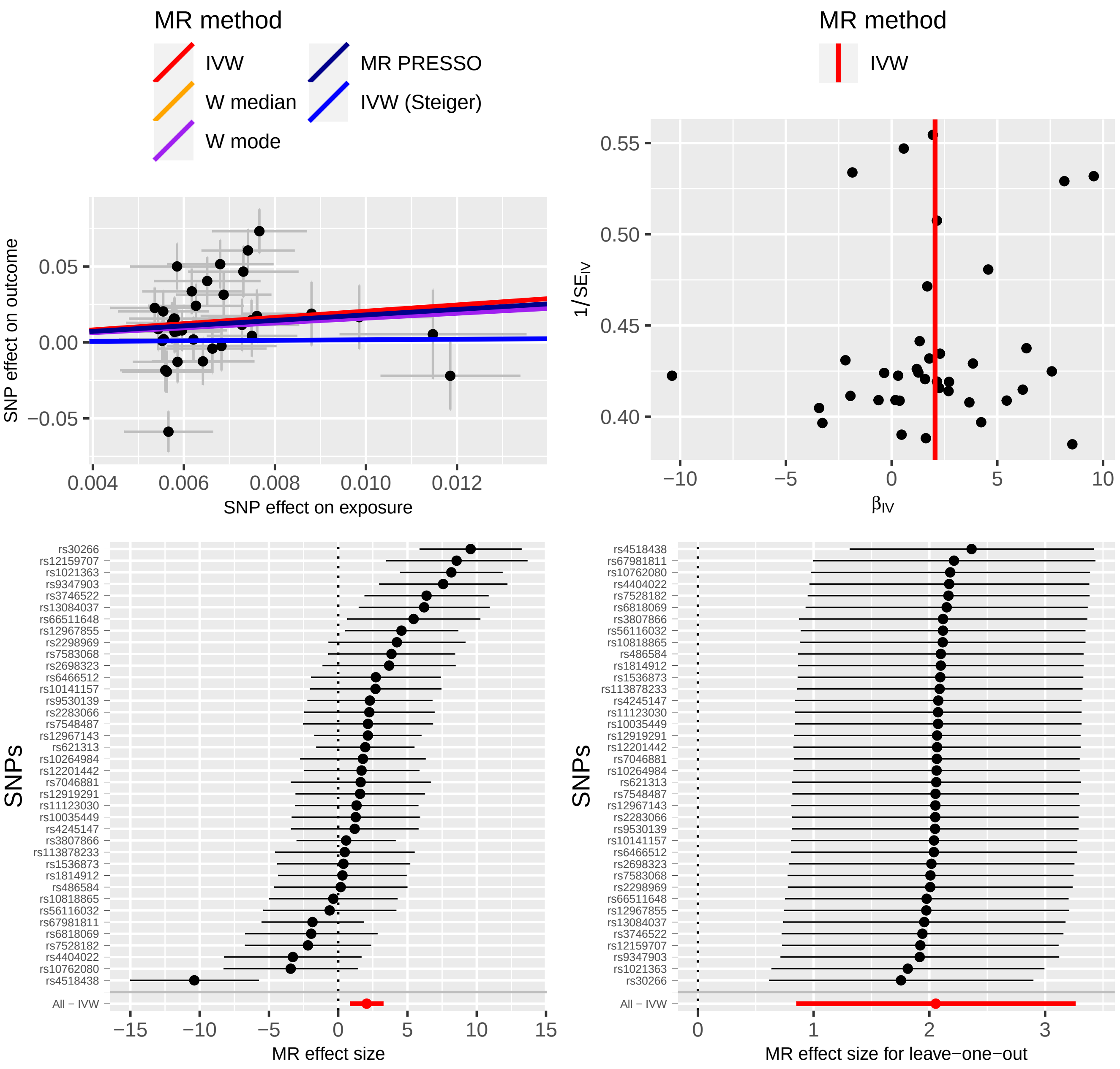

Supplement: dyac128_Supplementary_Data [file dyac128_supplementary_data.zip › dyac128_Supplementary_Data/ije-2021-03-0537-File008.pdf]
