# Supplementary material for: Mendelian randomization analysis for attention deficit/hyperactivity disorder: studying a broad range of exposures and outcomes
Source: Int J Epidemiol. 2022 Jun 12;52(2):386–402. doi: 10.1093/ije/dyac128 (PMC10114062; doi:10.1093/ije/dyac128)
Supplement: dyac128_Supplementary_Data [file dyac128_supplementary_data.zip › dyac128_Supplementary_Data/ije-2021-03-0537-File009.pdf]

Supplementary Figure 2 Forest plot for maternal smoking multivariable Mendelian randomization analysis

The total effect of ADHD genetic liability on maternal smoking is presented (“Total”), together with its direct effect when accounting for each potential mediator separately and together (“All”). Effect sizes and 95% confidence intervals are presented as maternal smoking OR per unit increase in ADHD log(OR).

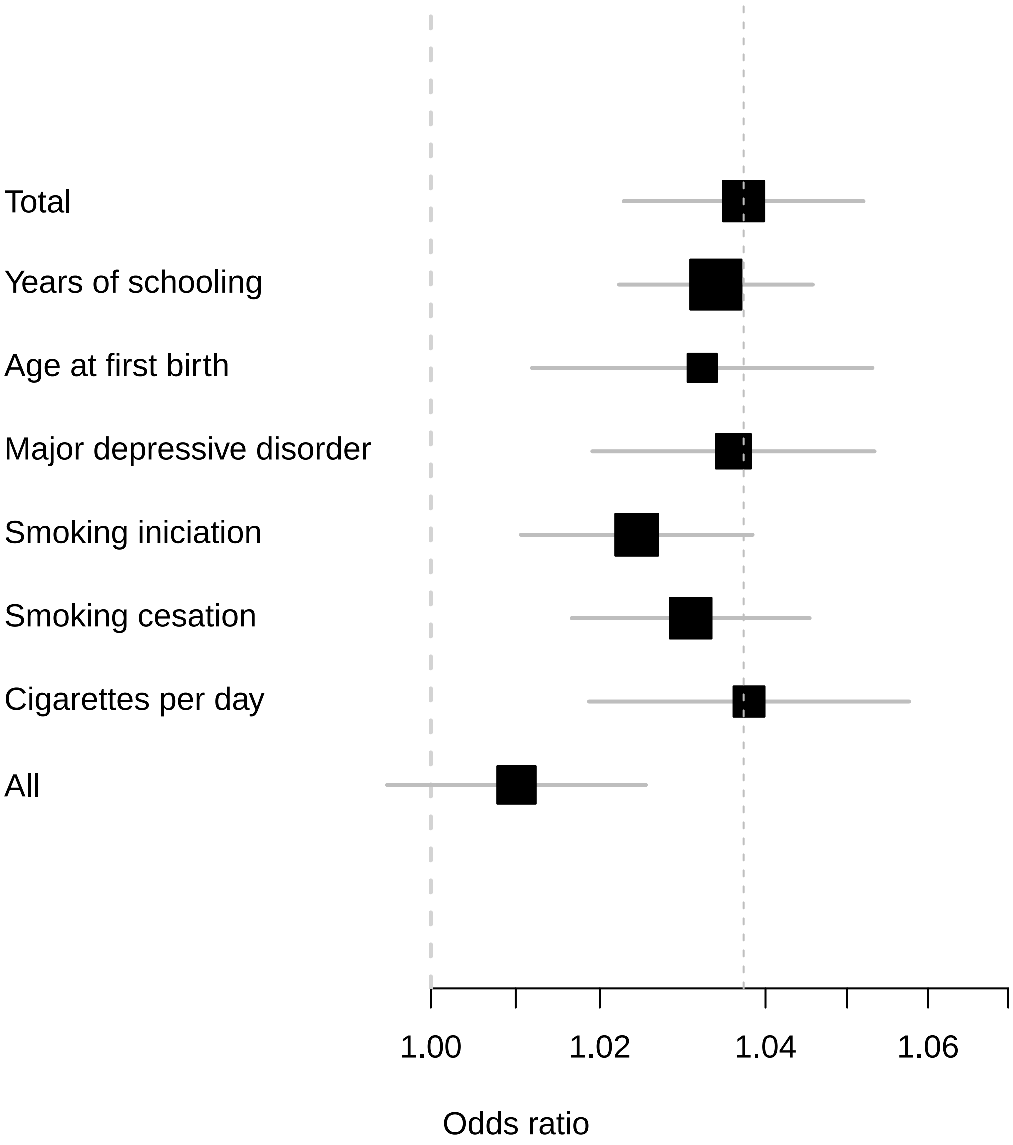

Total
Years of schooling
Age at first birth
Major depressive disorder
Smoking iniciation
Smoking cesation
Cigarettes per day
All
1.00
1.02
1.04
1.06
Odds ratio
